# Both isoforms of Drosophila ApoLpp (ApoB) cross the blood–brain barrier in adults

**DOI:** 10.1093/genetics/iyaf224

**Published:** 2025-10-16

**Authors:** Michael J Stinchfield, Sudhindra R Gadagkar, Michael B O’Connor, Stuart J Newfeld

**Affiliations:** School of Life Sciences, Arizona State University, Tempe, AZ 85287-4501, United States; Biomedical Sciences Program, Midwestern University, Glendale, AZ 85308, United States; Department Genetics, Cell Biology and Development, University Minnesota, Minneapolis, MN 55455, United States; School of Life Sciences, Arizona State University, Tempe, AZ 85287-4501, United States

**Keywords:** cortex, glia, medulla, morphotrap, myoglianin, phylogenetics

## Abstract

Human ApolipoproteinB (ApoB) exists in two isoforms that are packaged into low density lipoprotein particles and are major contributors to atherosclerosis. Alternatively, Drosophila Apolipoprotein Lipophorin (ApoLpp) also exists in two isoforms packaged into lipoprotein particles that cross the blood–brain barrier (BBB) in second instar larvae where they deliver lipids to neuroblasts. To extend our understanding of ApoLpp function to adult brains and suggest new hypotheses for human ApoB, we document evolutionary conservation between the two N-terminal isoforms human ApoB48 and fly ApoLppII. Then our tissue-specific analyses including rescue of *apolpp* lethality and *apolpp* RNAi showed that *apolpp* expression in the fat body is both necessary and sufficient for survival to adulthood. Our imaging studies of ApoLpp in the adult brain employed endogenous isoform-specific tagged proteins generated by the Fourth Chromosome Resource Project. Images revealed that both ApoLpp isoforms are present in the adult brain with ApoLppII accumulation prominent near glia. Nanobody morphotrap experiments that blocked tagged ApoLpp at the BBB demonstrated that ApoLpp detected inside the adult brain is exogenous. An N- and C-terminal tagged ApoLpp transgene expressed solely in the fat body facilitated tracking of each isoform from fat body secretion to the BBB and then inside the adult brain. Overall, our data suggest that the known role of ApoLpp in lipid delivery to larval brains likely continues in adults. Strong conservation between ApoLppII and ApoB48 supports the hypothesis that ApoB48 may have a role in the brain outside the circulatory system.

## Introduction

The Apolipoprotein (Apo) family, in humans and other species is not a traditional protein family where all members share sequence similarity derived from a common ancestor. Instead, Apo is a family that investigators defined based on the ability to bind lipids. As organisms utilize lipids in a myriad of ways, this function originated multiple times during the course of life on earth. Thus, except for recent gene duplications, Apo proteins in a single species are only tenuously connected to each other in intraspecific trees. However, a homologous relationship that is actively maintained by selection for a specific function should be visible in interspecific trees.

The APOB gene is unique among human Apo family members. Its 14 kb mRNA encodes the largest human Apo family member by an order of magnitude, a 4,563 amino acid isoform called ApoB100. Another novel feature of APOB is that its mRNA undergoes editing to convert cytosine to uracil at nucleotide 6,538. Editing converts a normally translated CAA codon to a stop codon, creating an N-terminal 2,180 amino acid isoform called ApoB48. Editing occurs in the liver where ApoB48 is the exclusive form and in the gut where ApoB100 and ApoB48 are generated (Higuchi et al. 1988). Both ApoB isoforms are secreted into the bloodstream where ApoB100 is the primary protein in very low density lipoproteins and ApoB48 the primary in ultralow density lipoproteins. In colloquial terms, ApoB isoforms contribute to two types of “bad cholesterol.” Heterozygosity for pathogenic mutations in APOB is the cause of 5% to –10% of cases of familial hypercholesterolemia (Beheshti et al. 2020). No roles for ApoB outside the circulatory system are known.

However, evidence of potential roles for human ApoB outside the circulatory system is accumulating. In humans, mutations in APOB and circulating levels of ApoB have been associated with cognitive impairment: (i) loss of function mutations in APOB are among the causes of familial Alzheimer's disease (Aumont-Rodrigue et al. 2024), (ii) high serum ApoB levels are connected to greater risk of dementia (Gong et al. 2022), and (iii) a high ratio of ApoB/ApoA1 in circulation predicts the extent of diminished mental capacity in stroke patients (Lee et al. 2023). In rodents, studies of adult rat brains showed ApoB in a complex with the Low Density Lipoprotein (LDL) receptor on glia (Jung-Testas et al. 1992) and in an in vitro model the mouse LDL receptor was shown to transcytose the blood–brain barrier (BBB) (Dehouck et al. 1997). These studies suggest the hypothesis that ApoB transits the BBB to perform an unknown role in brain. Upstream of APOB, the brain has a role with glucagon like peptide-1 (GLP-1) signals from the hypothalamus regulating ApoB liver and gut expression and secretion (Farr et al. 2015).

The possibility that studies of Apolipoprotein Lipophorin (ApoLpp) can be employed to suggest hypotheses for ApoB is based on known similarities between the two. Among these are that ApoLpp is also large (3,350 amino acid) and subdivided, in this case by furin cleavage, to create N-terminal (ApoLppII) and C-terminal (ApoLppI) isoforms. ApoB48 and ApoLppII N-terminal isoforms both contain a Vitellogenin domain (pfam09172). In humans and flies the N-terminal isoform and full length proteins are incorporated into circulating lipoprotein particles. However, to date only in fly second instar larvae do particles containing ApoLpp cross the BBB to deliver lipids to neuroblasts (Brankatschk and Eaton 2010). Particles containing the ApoLpp sibling Apolipoprotein Lipid Transfer Particle (ApoLTP) also cross the BBB during second instar to deliver lipids to insulin producing neurons (Brankatschk et al. 2014).

To extend our understanding of ApoLpp brain function to adults and perhaps provide evidence for a hypothesis that human ApoB has a role in the brain, we established an evolutionary connection between the N-terminal isoforms ApoB48 and ApoLppII. Then our rescue studies showed that ApoLpp from the fat body, an organ functionally analogous to the ApoB expressing liver (Ugur et al. 2016; Meschi and Delanoue 2021), is necessary and sufficient for flies to survive beyond the *apolpp* mutant embryonic lethal phenotype. Imaging studies of ApoLpp indicated that both N- and C-terminal isoforms are present in the adult brain. Morphotrap studies with tagged ApoLpp blocked at the BBB demonstrated that the ApoLpp in the adult brain is exogenous. Utilizing an N- and C-terminal tagged version of ApoLpp expressed solely in the fat body, we tracked both isoforms from fat body secretion to the BBB and into the adult brain. Overall, the fly data suggest that the previously known role of ApoLpp in second instar lipid delivery to the brain persists through third instar to adulthood. Our phylogenetic data connecting ApoLppII and ApoB48, supports the hypothesis that ApoB can transit the BBB to perform a function in the brain.

## Materials and methods

### Phylogenetics of human, fly and nematode Apolipoproteins

#### Human sequences

Twenty-three human Apo proteins were identified by name in Uniprot (uniprot.org). Reference sequences were downloaded (ncbi.nlm.nih.gov/refseq) and their structural domains retrieved (pfam-legacy.xfam.org). The ApoB sequence was divided and analyzed separately as ApoB48 (Higuchi et al. 1988) and the remainder of ApoB100 that we named ApoB52. Microsomal Triglyceride Transfer Protein (MTTP) was chosen as the outgroup since it has a similar function but not the Apo name. Splitting ApoB plus the outgroup yielded 25 human sequences.

#### Fly sequences

Eight fly Apo proteins were identified via the DIOPT function in Flybase, the longest reference sequence downloaded and structural domains retrieved. The ApoLpp and ApoLTP sequences were divided and analyzed separately as isoforms generated by furin cleavage. Isoform naming followed Brankatschk and Eaton (2010) with N-terminal isoforms ApoLppII and ApoLTPII, and C-terminal isoforms ApoLppI and ApoLTPI. Fly Microsomal triacylglycerol transfer protein (Mtp) was recovered by BLASTp as the homolog of human MTTP and employed as the outgroup. Splitting ApoLpp and ApoLTP plus the outgroup yielded 11 fly sequences.

#### Nematode sequences

Nine nematode Apo proteins were identified via structural domains in WormBase, the longest reference sequence downloaded and structural domains retrieved. Each of the Vit-1 through Vit-6 sequences was divided as predicted by a furin consensus cleavage site conserved in ApoLpp and ApoLTP. The naming convention for ApoLpp was applied. For example, for Vit-1 the N-terminal region was named Vit-1II and the C-terminal region was named Vit-1I. The outgroup was Microsomal triacylglycerol transfer protein (Dsc-4) due to its similar function, but it is not the homolog of either the human or fly outgroup by reciprocal BLASTp. Splitting of Vit-1 through Vit-6 plus the outgroup yielded 16 nematode sequences.

#### Phylogenetics

Analyses were conducted in MEGAX (Kumar et al. 2018). Protein sequences were aligned with default settings using MUSCLE (Edgar 2004). Complete Deletion, the most conservative method for handling gaps was employed with the alignments for each species and the two species pairs (human-fly and fly-nematode) for best-fit model selection (lowest Bayesian Information Criterion) then Maximum Likelihood tree building. With Complete Deletion, a position is considered noninformative and removed from consideration if even one sequence in the alignment has a gap at that position. For the alignment of all three species, the Partial Deletion method was employed with an 80% cutoff for best-fit model selection (lowest Bayesian Information Criterion) and Maximum Likelihood tree building. With Partial Deletion, a position that has a gap in up to 19% of the aligned sequences was still considered informative and retained. In all trees, nonparametric bootstrapping was applied with 500 replicates to assess the relative strength of each node.

We took into account the fact that the absence of common ancestry violates two equality assumptions of the maximum likelihood algorithm (equal rates of substitution in all sequences and equal constraints on branch length; Dhar and Minin 2016). Note these are not the core independence assumptions of the algorithm (individual substitutions in a single sequence are independent of all other substitutions in that sequence and substitutions in one sequence are independent of substitutions in all other sequences; Felsenstein 2004). Violations of the independence assumptions would completely undermine the algorithm. The impact of equality assumption violations is less severe and we counterbalanced the impact by consistently applying independent node validation. Validation methods such as reciprocal BLASTp and identifying shared structural domains were routine.

### Stocks, mutagenesis, tissue-specific rescue of *apolpp* lethality and RNAi

#### Stocks

Three UAS *apolpp* cDNA clones were obtained from Marko Brankatschk (Biotec TU-Dresden; Brankatschk and Eaton 2010) then injected by BestGene (www.thebestgene.com). Transformant lines were obtained and mapped to a chromosome. Stocks designated as UAS. ApoLpp.wt contain a wild type *apolpp* cDNA. UAS.5′-HA-ApoLpp-Myc-3′ stocks contain a double tagged *apolpp* cDNA. UAS.5′-HA-ApoLpp-Myc furin-resistant stocks contain the doubled tagged *apolpp* cDNA with a mutation in the furin cleavage site. Five GAL4 stocks were utilized in rescue crosses: Repo.GAL4 glia (BL#7415), CG.GAL4 fatbody/hemocytes (Asha et al. 2003, BL#7011), Mhc82.GAL4 muscle (Schuster et al. 1996), NPC1b.GAL4 gut (BL#95257) and R4.GAL4 fat body specific (Lee and Park 2004, BL#33832). *apolpp^102^* is a 29  base pair (bp) deletion that removes the initiator methionine (Tanaka et al. 2021). UAS.*apolpp*.RNAi (BL#33388) was validated with ubiquitous expression employing Da.GAL4 (Wodarz et al. 1995, BL#55851) to recreate the *apolpp* loss of function lethal phenotype. Elav.GAL4 with neuronal specific expression (Luo et al. 1994, BL#458) was crossed to *apolpp*.RNAi as a control for off target effects that showed there were none. Three fourth chromosome balancers were: adult eye like Glazed from a lethal insertion in *Gat* (*TI{GMR-HMS04515}Gat^eya^*), fourth longitudinal wing vein truncation from a lethal inversion in *In(4)ci^D^* (both in BL#90852) and a lethal neural GFP enhancer trap (*P{w+; ActGFP}unc-13^G^;* BL#4759).

#### Mutagenesis

For generation of new *apolpp* mutants, two gRNAs roughly 2.3 kb apart on opposite sides of the furin cleavage site were selected using CRISPR Optimal Target Finder (https://flycrispr.org/target-finder) and cloned into pU6-BbsI (Gratz et al. 2013). Plasmids expressing these gRNAs were injected into a *vasa*.Cas9-expressing stock (BL#5491) by GenetiVision (genetivision.com). Fifteen G^0^ males were crossed to *P{w+; ActGFP}unc-13^G^/In(4)ci^D^*. Ten F^1^ males of the genotype mutant?/*In(4)ci^D^* from each G^0^ male were backcrossed to *P{w+; ActGFP}unc-13^G^/In(4)ci^D^* flies. After mating, each F^1^ male was subject to PCR with primers covering the gRNA targets. Sequencing identified 2 new mutant alleles.

gRNA 1 (C-terminal): 5′-CTTCAATGGGCTGACGAAACCACG-3′

gRNA 2 (N-terminal): 5′-CTTCGGGAATAGAAGCGGAACATG-3′

PCR *apolpp* 1 (C-terminal) forward: 5′-CTTCAATGGGCTGACGAAACCACG-3′

PCR *apolpp* 1 reverse: 5′-AAACCGTGGTTTCGTCAGCCCATT-3′

PCR *apolpp* 2 (N-terminal) forward: 5′-CTTCGGGAATAGAAGCGGAACATG-3′

PCR *apolpp* 2 reverse: 5′-AAACCATGTTCCGCTTCTATTCCC-3

#### Lethal phase analysis

To determine the lethal phase of *apolpp* transheterozygous loss of function mutants, *apolpp^70A^*/*P{w+; ActGFP}unc-13^G^* females were crossed to *apolpp^32B^*/*P{w+; ActGFP}unc-13^G^* males and eggs collected on apple juice agar plates for 6 h. Fifty GFP-negative eggs were picked and placed in rows on another agar plate. Twenty-five of these eggs (*apolpp^70A^/apolpp^32B^*) hatched into first instar larvae but died shortly thereafter without an increase in size. Of the 25 that did not hatch, five were subsequently noted as GFP-positive and thus *P{w+; ActGFP}unc-13^G^* heterozygotes that were mispicked. The remaining 20 *apolpp^70A^/apolpp^32B^* eggs appeared normal but failed to hatch.

#### Tissue specific rescue of lethality

For the rescue experiment, males were employed with one of the five GAL4 transgenes listed above on chromosome two over *CyO P{w; ActGFP}JMR1* or on chromosome three over *TM3 P{w[+mC] =ActGFP}JMR2 Ser* and with *apolpp^102^* on chromosome four over *P{w+; ActGFP}unc-13^G^.* These were crossed to females with one of the three UAS.ApoLpp transgenes on chromosome three over *TM6B P{w+; Ubi-GFP.S65T}PAD2 Tb* with *apolpp^70A^* on chromosome four over *P{w+; ActGFP}unc-13^G^.* Three days after mating, eggs were collected on apple juice agar plates for six hours. Two hundred eggs were picked and placed on a gridded plate, then the eggs and agar grid were transferred to a culture bottle with media. This was done twice more for each GAL4/UAS combination. Adults were scored over a 15-day period and GFP-negative individuals considered rescued with a genotype of GAL4 on chromosome two or three, UAS on chromosome three and *apolpp^102^*/*apolpp^70A^* on chromosome four.

#### Tissue specific RNAi phenocopy of *apolpp* lethal phase

Twenty females carrying one of four GAL4 transgenes, Da.GAl4, CG.GAL4, R4.GAL4 and Elav.GAL4 were crossed to 10 males with UAS.*apolpp.*RNAi over *TM6B P{w+; Ubi-GFP.S65T}PAD2 Tb.* After three passes in clear glass vials progeny (>100 third instar larvae that became >100 adults) were scored as larvae and again as adults for the presence or absence of the *TM6b Tb* phenotype with surviving UAS.*apolpp.*RNAi individuals expected to show a wild type phenotype at both stages.

### Immunohistochemistry of larval and adult gene and protein expression

#### Stocks *apolpp*

Three stocks with a distinct transgene in the *apolpp* locus were created by the Fourth Chromosome Resource Project (FCRP). The N-terminal tagged ApoLppII N-enhanced Green Fluorescent Protein (eGFP) and transcriptional reporter *apolpp* T2A.GAL4 are DoubleHeader conversions of an *apolpp* second intron CRIMIC (CR70471-BL#9179; Stinchfield et al. 2024). ApoLppII N-eGFP (BL#97744; Kyoto#118965) allows ApoLppII to be visualized. *apolpp* T2A.GAL4 includes an in-frame stop codon that terminates translation of the endogenous *apolpp* mRNA and a downstream internal ribosome entry site that initiates the translation of GAL4 mRNA in the expression pattern of the endogenous *apolpp* mRNA. When crossed to flies carrying UAS.GFP, the progeny express GFP in the pattern of the *apolpp* mRNA. The ApoLppII N-eGFP and *apolpp* T2A.GAL4 transgenes are homozygous lethal and balanced over *Gat* (*TI{GMR-HMS04515}Gat^eya^*). ApoLppI C-eGFP was created by the Gene Disruption Project via a scarless CRISPR exon swap of the last coding exon that leaves behind the endogenous *apolpp* 3′ UTR (gift of Oguz Kanca and Hugo Bellen; Kanca et al. 2022). The ApoLppI C-eGFP transgene is homozygous viable.

#### Stocks *myo*

Two stocks with a distinct transgene in the *myoglianin* (*myo)* locus were created. Myo eGFP (BL#600215; Kyoto#118960) and *myo* T2A.GAL4 were generated by the FCRP via DoubleHeader conversion of a *myo* second intron CRIMIC (CR02262; BL#92207). The Myo eGFP and *myo* T2A.GAL4 transgenes are homozygous lethal. Additional stocks were 9-137.GAL4 (DeSalvo et al. 2014), UAS.CD8.RFP (BL#27398) and UAS.nls.GFP (BL#4775).

#### Brain expression assays

Brains, fat body and body wall muscle from third instar larvae and adults were dissected in cold phosphate-buffered saline (PBS) buffer, fixed in 4% formaldehyde for 20 min, dehydrated in methanol then stored −20 °C. They were then exposed to primary antibodies to detect eGFP from an ApoLpp or Myo transgene, to detect UAS.GFP driven by either *apolpp* or *myo* T2A.GAL4, or to detect the HA or Myc tagged ApoLpp isoforms. Detection of UAS.RFP driven by 9:137.GAL4 was via direct luminescence without antibodies. Brains were counterstained with antibodies against Repo that identifies all glial nuclei or against Vvl that identifies BBB glial nuclei. Antibody detection for eGFP in expression studies employed a modified TSA reaction (Stinchfield et al. 2024). Antibody detection of eGFP in morphotraps employed only antibodies as described (Goldsmith and Newfeld 2023). In tracking experiments with UAS.5′-HA-ApoLpp-Myc-3′, HA was detected with Roche rat 3F10, Myc detected with DSHB monoclonal mouse 9E−10, Vvl as described (previously known as Drifter; Tran et al. 2018) and DAPI in staining solution at 300 μM in PBS. Brains were imaged on a Leica SP8 confocal microscope with slices acquired every 2 μm and analyzed in FIJI. Scale bars are provided.

### Larval and adult morphotraps

#### Stocks

The primary morphotrap was *M{w[+mC]=lexAop-UAS-morphotrap.ext.mCh}ZH-35B/CyO* (Harmansa et al. 2015, BL#68170). This transgene encodes an UAS controlled fusion of the external portion of the transmembrane protein CD8 to a nanobody against GFP. We call this experimental morphotrap CD8-morpho. This transgene effectively traps nearby GFP-tagged secreted proteins at the outer membrane of any expressing cell. CD8-morpho was driven in the BBB by 9-137.GAL4 and tracked by co-expression of UAS.CD8.RFP, a combination that we call 9-137:RFP. As a negative control, we employed another morphotrap *M{w[+mC]=lexAop-UAS-morphotrap.ext.mCh}ZH-86Fb* (Harmansa et al. 2017, BL#68173). This transgene encodes a nanobody to GFP fused to the external portion of the transmembrane protein Nrv1 that only inserts into the membrane on the basolateral surface of polarized epithelia. We call this control morphotrap Nrv-morpho. This is a negative genotype control since the BBB is a squamous epithelia providing no appropriate location for nanobody insertion.

#### Morphotrap genotypes and brain expression

Stocks homozygous for chromosome two insertions of CD8-morpho with either ApoLppII N-eGFP or ApoLppI C-eGFP or *apolpp^81D^* balanced over *TI{GMR-HMS04515}Gat^eya^* on chromosome four were created. Companion stocks containing chromosome two with 9-137:RFP balanced over *CyO* plus either ApoLppII N-eGFP or ApoLppI C-eGFP or *apolpp^81D^* balanced over *In(4)ci^D^* on chromosome four. Crosses produced progeny that were CD8-morpho transheterozygotes with 9-137:RFP on chromosome two and *apolpp* transheterozygotes on chromosome four. Four distinct CD8-morpho genotypes were generated to allow for morphotrap of either ApoLppII N-eGFP or ApoLppI C-eGFP or both. One genotype was transheterozygous for ApoLppII N-eGFP/*apolpp^81D^*. The second was homozygous for ApoLppI C-eGFP. The third was transheterozygous for ApoLppI C-eGFP/*apolpp^81D^*. The fourth was transheterozygous for ApoLppI C-eGFP/ApoLppII N-eGFP. eGFP and Repo detected as above.

## Results

### Human ApoB48 is conserved in flies and nematodes

Sequence conservation as revealed by phylogenetics has successfully predicted multiple conserved functions and regulatory mechanisms for cell signaling pathways (e.g. Konikoff et al. 2008 predicted Dupont et al. 2009). These studies employed alignments and trees with human, fly and nematode proteins as their evolutionary focus. We employed the same approach. Conservation across these three species reveals the value of a protein for organismal survival such that natural selection maintained it during the 700 million years since these species last common ancestor (Kumar et al. 2022 ).

There are 23 human Apo proteins in the Uniprot database (Supplementary Table 1). They range in size from 99 amino acids (ApoC3) to 4,563 amino acids (ApoB), with all but ApoB below 450 amino acids. Similar variability exists in their subcellular location: 16 are secreted, four are cytoplasmic, three are transmembrane including two in the mitochondrial inner membrane while one remains a prediction. Family members are ApoA through ApoOL and include the multi-member subfamilies ApoA, ApoC, and ApoL. Note that ApoP is an apoptotic protein and excluded. Four defined structural domains are found in multiple Apo proteins: (i) pfam04711 in ApoA2 and ApoD, (ii) pfam01442 in ApoA1/A4/A5 and ApoE, (iii) pfam09769 in ApoO and ApoOL, and (iv) pfam05461 in ApoL1-6. The ApoL proteins reside contiguously on chromosome 22q11.2. Two other Apo chromosomal contigs contain proteins from multiple groups. These are: (i) ApoE, ApoC1, ApoC4, ApoC2 on 19q13.32, and (ii) ApoA1, ApoC3, ApoA4, ApoA5 on 11q23.3.

**Fig. 1.**
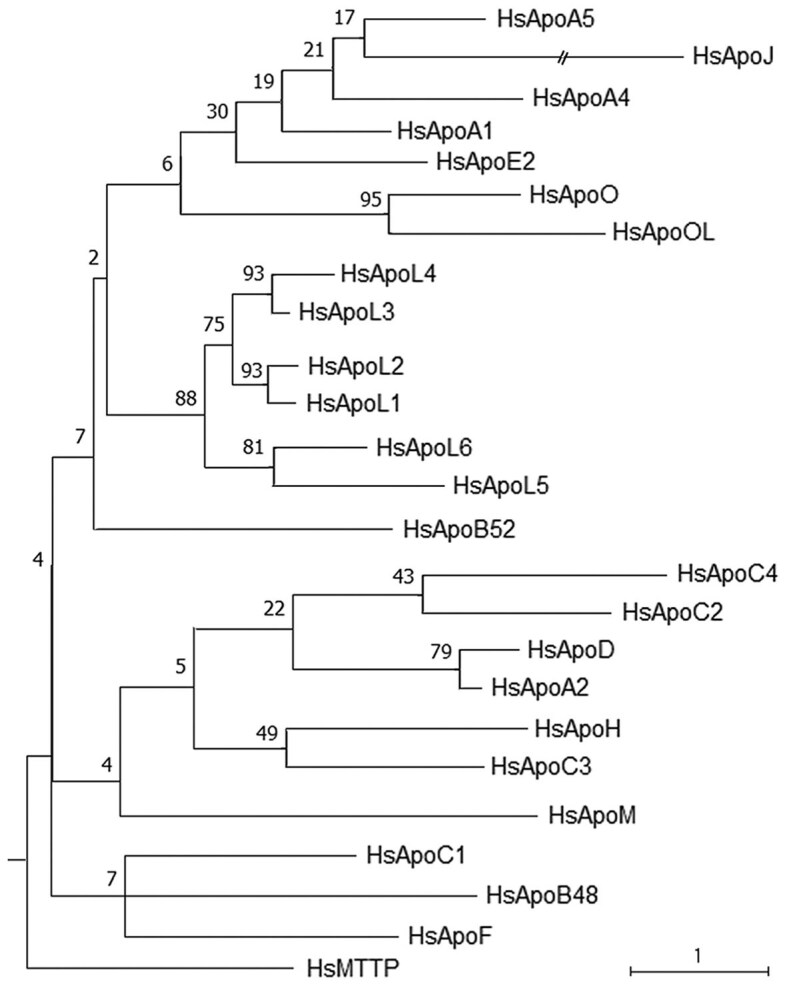
Tree of 25 human Apolipoproteins. Maximum likelihood tree. The percentage of bootstrap replicates (out of 500) in which the associated node appeared is shown. Node values are for internal comparison only as they do not measure statistical confidence. Branch lengths are to scale and measured in the number of substitutions per site (a scale bar is provided). ApoB48 and ApoB52 (C-terminal sequence of ApoB100) are analyzed as separate sequences plus an outgroup. There are 171 informative positions in the underlying MUSCLE alignment (Supplementary Fig. 8). Among the named groups only ApoL and ApoO cluster unambiguously while ApoA and ApoC are dispersed.

We examined paralogous relationships via an alignment (Supplementary Fig. 8; note all alignments are at the end of supplementary material) and an intraspecific tree (Fig. 1). Since strong protein clusters suggest a level of sequence similarity that implies similar ancestry, we examined the tree for informative clusters. The tree shows that only ApoL proteins resemble an ancestry-based subfamily (pfam05461). All members cluster together and exclude other Apo proteins with three recent duplications evident. This fits well with the chromosomal contiguity of ApoL proteins. For ApoC proteins, only ApoC2 and ApoC4 are a recent duplication with ApoC3 and ApoC1 divergent. This is distinct from ApoC1 contiguity on the chromosome with ApoC2 and ApoC4, suggesting divergent selection on the older ApoC1. For ApoA proteins, ApoA2 is divergent in the tree and does not cluster with ApoA1, ApoA4 and ApoA5 on the chromosome. The strength of the node linking ApoA2 and ApoD (reinforced by results from reciprocal BLASTp) indicates that they are a recent duplication and that perhaps ApoA2 should be called ApoD2. The presence of only ApoL as a true subfamily underscores the non-ancestral nature of the human Apo protein family.

There are eight fly Apo proteins identified via DIOPT in Flybase (Supplementary Table 2). They range in size from 157 to 4,333 amino acids with two above 3,350 amino acids. The two largest (ApoLpp and ApoLTP) are subject to furin cleavage creating N-terminal (ApoLppII and ApoLTPII) and C-terminal isoforms (ApoLppI and ApoLTPI). Six proteins are secreted, one is cytoplasmic and one is in the mitochondrial inner membrane. Three structural domains shared with humans are: (i) pfam09172 Vitellogenin-N (ApoB with fly ApoLpp and Cv-d), (ii) pfam00061 Lipocalin (ApoM with fly CG31659/Fabp/Glaz/Nlaz), and (iii) pfam09769 ApoO (ApoO/ApoOL with fly Mic26-27).

We examined paralogous relationships via an alignment (Supplementary Fig. 9) and an intraspecific tree (Supplementary Fig. 1). In the tree two strong clusters stand out. Glaz and Nlaz are a recent duplication (as noted previously, Liu et al. 2017) and the N-terminal isoforms ApoLppII and ApoLTPII. The cluster of the C-terminal isoforms ApoLppI and ApoLTPI is distant and considerably weaker. This scenario suggests divergent selection on the N-terminal and C-terminal isoforms with the similarity of ApoLppII and ApoLTPII maintained by selection.

**Fig. 2.**
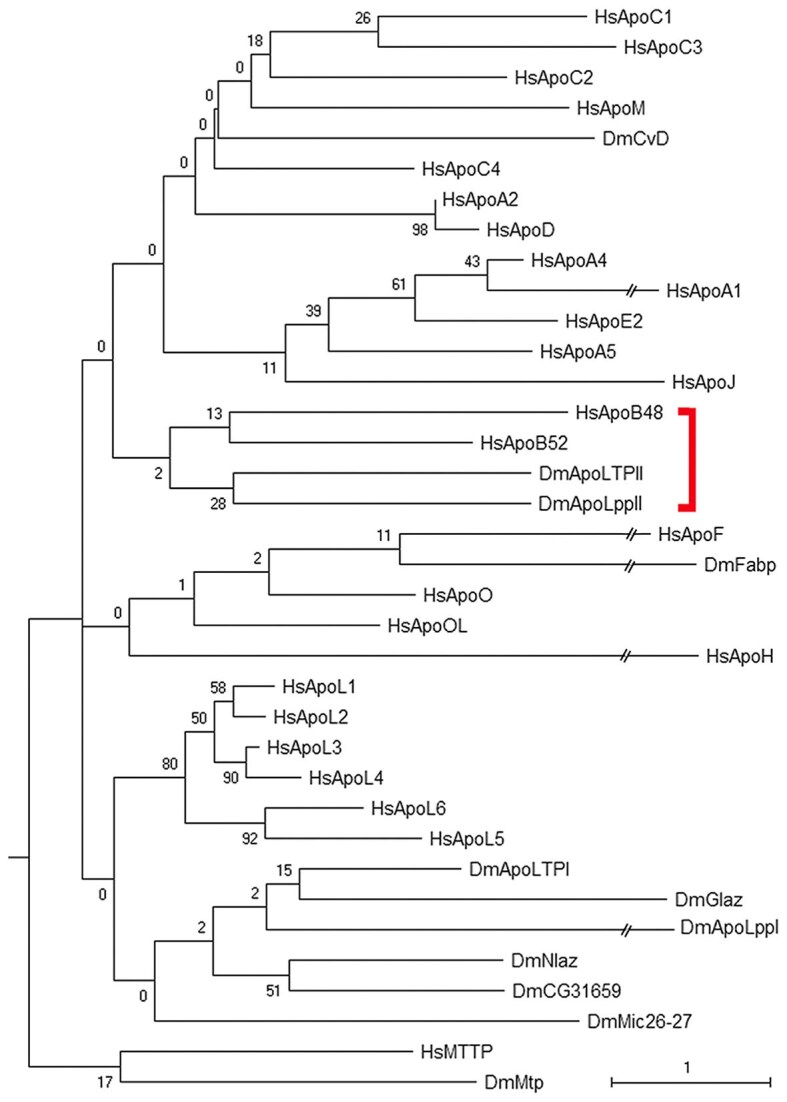
Human ApoB48 and fly ApoLppII are conserved. Tree as in Fig. 1. There are 25 human (Hs) and 11 fly (Dm) sequences in the tree with both outgroups. The two isoforms of human ApoB, fly ApoLpp and fly ApoLTP are analyzed separately. There are 172 informative positions in the alignment (Supplementary Fig. 10). Deep nodes shown as zero are actually present one to four times (below the 1% bootstrap threshold for 500 replicates) and are the most frequent among the connected sequences. Confidence in the tree is bolstered by the clustering of the two outgroups at the bottom as the most divergent. Red bar indicates the cluster containing human ApoB48 and ApoB52, fly ApoLppII and ApoLTPII. The other interspecific cluster (human ApoF with fly Fabp) is shown directly underneath but is not related.

To identify conservation between human and fly Apo proteins we generated an interspecific alignment (Supplementary Fig. 10) and a tree (Fig. 2). The tree has only two clusters that contain human and fly proteins. Within one, ApoB48 (N-terminal region of human full length ApoB100) and the C-terminal region of ApoB100 (called here ApoB52 that is not a biological isoform) are linked. Intra-protein similarity between these regions was previously noted (Chen et al. 1986). The fly N-terminal regions, ApoLppII and ApoLTPII are also clustered together and both are linked with the ApoB48/ApoB52 cluster. The only other human and fly cluster is human ApoF with fly Fabp. The remaining proteins are in monospecific clusters suggesting only human ApoB and ApoF predate the human and fly divergence. One note of caution is that the similarity of human ApoA2 and ApoD from their recent duplication, obscures in our trees the previously noted similarity of human ApoD with fly Glaz and Nlaz. On the other hand, confidence in the tree is bolstered by clustering of the two outgroups that share pfam19444.

To further extend the reach of our evolutionary analysis, an interspecific tree of fly and nematode Apo proteins was generated. There are nine nematode Apo proteins identified via WormBase (Supplementary Table 3). The six Vit proteins are roughly 1,650 amino acids, while the other three are roughly 250 amino acids. All six Vit proteins contain a predicted furin cleavage site that is also present in ApoLpp and ApoLTP. Thus, Vit-1 through Vit-6 likely are split into isoforms to perform their function. We name the isoforms with the convention of ApoLpp (II is N-terminal and I is C-terminal). The Vit furin cleavage site is also present in the sole Vit protein in the distantly related nematode *Pristionchus pacificus*, called Vit-6 (KAF8354296) as it best matches Vit-6 in *Caenorhabditis elegans*. Further evidence that Vit-6 is the original Vit protein in *C. elegans* is that Vit-6 is on chromosome 4 while all the others are on the X chromosome. Vit structural domains shared with ApoLpp and ApoLTP are: pfam09172 Vitellogenin-N (in the N-terminal isoform) and pfam00094 von Willebrand type D (in the C-terminal isoform).

The interspecific alignment (Supplementary Fig. 11) and tree (Supplementary Fig. 2) with fly and nematode Apo proteins reveals that Vit-6II clusters with ApoLppII and ApoLTPII. The Vit-1II through Vit-5II isoforms are in two nearby clusters. Vit-1I through Vit-6I also cluster together, though at a distance from the VitII cluster. The Vit clustering pattern suggests multiple recent rounds of gene duplication in the *C. elegans* lineage. There are no other clusters of fly and nematode proteins indicating that ApoLppII and ApoLTPII alone in flies are conserved in nematodes, as Vit-6II.

The lack of common ancestry among Apo proteins in each species overwhelmed multiple approaches to constructing a tree from an alignment of humans, flies and nematodes (Supplementary Fig. 12). Neither a tree of human and nematode nor a tree of all three species was possible. Thus, by extension from the two interspecific trees, we suggest that among human Apo family members ApoB alone is conserved from a common ancestor with nematodes. In fairness, we are not the first to note a connection between ApoB and Vit proteins. This was suggested in a brief note nearly 40 years ago based on conserved structural domains (Baker 1988). For our purposes, evidence of evolutionary conservation all the way to nematodes increases the likelihood that new information about fly ApoLppII will suggest hypotheses for ApoB48 in humans.

### ApoLpp fat body expression is necessary and sufficient for survival to adult

Fly *apolpp* and human APOB are transcribed and secreted from the fat body (Palm et al. 2012) and liver (Higuchi et al. 1988), respectively. These are considered analogous tissues since they perform many of the same roles in triglyceride storage, metabolism and endocrine function (Ugur et al. 2016; Meschi and Delanoue 2021). We designed a pair of experiments to test the hypothesis that ApoLpp fat body expression alone is necessary and sufficient to rescue normally lethal *apolpp* mutant genotypes to adult.

Before embarking on these studies, we documented *apolpp* transcription and ApoLpp translation in the larval fat body utilizing *apolpp* T2A.GAL4 and ApoLppI C-eGFP transgenes (described below). Examining individual slices underlying the *apolpp* T2A.GAL4 image indicated that all larval fat body cells transcribe *apolpp* (Supplementary Fig. 3). The image of ApoLppI C-eGFP is consistent with all fat body cells translating *apolpp*. We then determined the stage of lethality for *apolpp* loss of function mutants. Three new lethal alleles of *apolpp* were generated by CRISPR: *apolpp^70A^* (2 bp deletion leading to a truncation at amino acid 124 causing a severe shortening of ApoLppII and complete loss of ApoLppI), *apolpp^32B^* (9 bp deletion within ApoLppII plus 39 bp deletion within ApoLppI) and *apolpp^81D^* (2 single base pair substitutions leading to missense mutations plus a 9 bp deletion within ApoLppII). The stage of lethality assay showed that these alleles as transheterozygotes died either as embryos or first instar larvae.

The rescue studies were conducted with a more severe loss of function genotype: *apolpp^102^/apolpp^70A^* (*apolpp^102^* has no initiator methionine; Tanaka et al. 2021). Expression of wild type ApoLpp in the fat body, with CG.GAL4, but no other tissue fully rescued *apolpp* loss of function mutants to adults (17.8% observed versus 12.5% expected; Table 1). Fat body expression of a double-tagged Apolpp rescued roughly one-third of expected adults. Expression of a cleavage resistant form of ApoLpp did not rescue. However, CG.GAL4 is also expressed in hemocytes and the lymph gland, leading us to repeat the experiment with R4.GAL4 that is fat body specific (Lee and Park 2004). We again obtained full rescue with wild type ApoLpp (12.1% observed versus 12.5% expected). These data show that fat body expression of ApoLpp is sufficient for survival of *apolpp* genomic loss of function mutants to adult.

**Table 1.** First instar lethality of *apolpp* mutants is rescued to adult only by fat body expression.

	CG.GAL4 (fat body)			Npc1.GAL4 (gut)			Mhc.GAL4 (muscle)			Repo.GAL4 (glia)		
	Adults	Rescue	%^a^	Adults	Rescue	%	Adults	Rescue	%	Adults	Rescue	%
UAS.ApoLpp.wt	236	42	17.8	311	0	0.0	72	0	0.0	103	0	0.0
UAS.5′-HA-ApoLpp-Myc-3′	288	13	4.5	100	0	0.0	210	0	0.0	92	0	0.0
UAS.HA-ApoLpp-Myc furin mutant	278	0	0.0	380	0	0.0	73	0	0.0	53	0	0.0

^a^Given the parental genotypes, the expected fraction of *apolpp* mutant progeny (*apolpp^102^*/*apolpp^70A^*) was one-eighth (12.5%) in all crosses.

In the complementary experiment, we employed UAS.*apolpp*.RNAi balanced over a chromosome with multiple larval and adult visible markers (Stubble, Tubby, and Humeral). First, to ascertain whether this transgene was capable of complete knock down we drove it ubiquitously with Da.GAL4. This combination was completely lethal prior to the third instar larval stage, thus recreating the lethal phase of *apolpp* loss of function mutants. Second, to test for off-target effects we expressed *apolpp*.RNAi in all neurons with Elav.GAL4 balanced over the marked chromosome. This combination had no effect on survival of experimental larvae or adult progeny, these were each present in their expected Mendelian proportions. Third, we drove *apolpp*.RNAi in the fat body with either CG.GAL4 or with R4.GAL4 to ascertain if expression in this specific tissue alone recreated the lethal phase of *apolpp* loss of function mutants. In both cases, no experimental third instar larvae were observed (Table 2). Failure to obtain larvae indicates that fat body expression of *apolpp* is necessary for survival of wild type flies. Taken together the rescue and RNAi data indicate that fat body expression is both necessary and sufficient for survival. The results further suggest that the fat body is the major source of ApoLpp.

**Table 2.** Fat body specific *apolpp*.RNAi phenocopies *apolpp* genomic loss of function.

	CG.GAL4 (fat body)			R4.GAL4 (fat body)			Da.GAL4 (ubiquitous)			Elav.Gal4 (neuron)		
	Adults	Not Tb	%^a^	Adults	Not Tb	%	Adults	Not Tb	%	Adults	Not Tb	%
UAS.*apolpp*.RNAi	140	0	0.0	131	0	0.0	127	0	0.0	147	50	34

^a^Given the parental genotypes, the expected fraction of *apolpp*.RNAi progeny (those not Tubby) was one-half for CG.GAL4 and one-third for the others.

To this point we know that ApoB and ApoLpp are: (i) both processed to create functional N-terminal isoforms (ApoB48 and ApoLppII), (ii) their N-terminal isoforms are highly conserved, and (iii) the tissues of origin for the N-terminal isoforms (liver and fat body, respectively) perform similar roles in homeostasis. These similarities add weight to our contention that new data on ApoLppII will suggest new hypotheses for ApoB48.

### ApoLppII N-eGFP and ApoLppI C-eGFP are present in adult brains

**Fig. 3.**
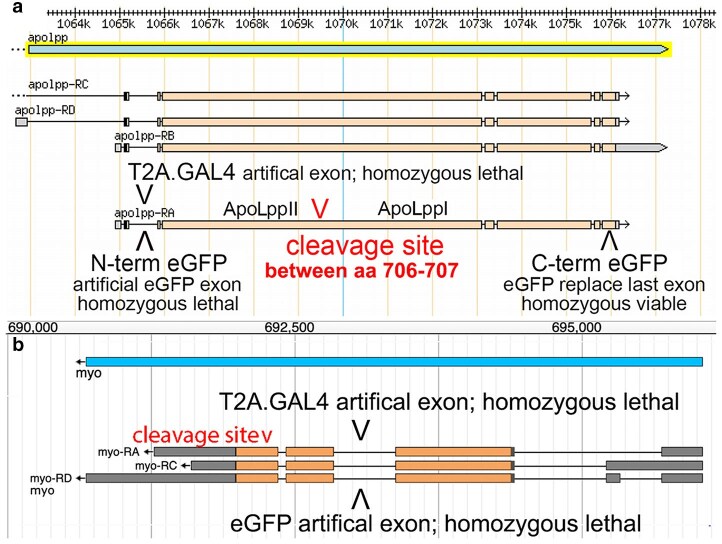
Fourth chromosome transgenes for *apolpp* and *myo*. a) Number line depicts the sequence of the fourth chromosome polytene region 102F7-8. The *apolpp* gene and four transcripts (from the numbered strand left to right) that encode a single ApoLpp protein are shown. The bottom transcript/protein is annotated to show a furin cleavage site between amino acids 706 and 707 and the isoforms created by cleavage. Three artificial exons carried in transgenes are shown. In the first intron of the coding region are two FCRP DoubleHeader conversions of CR70471. The *apolpp* T2A.GAL4 and ApoLppII N-eGFP transgenes are homozygous lethal. A custom designed CRIMIC provided by Oguz Kanca (Kanca et al. 2022; Bellen lab, Baylor) replaced the last coding exon of *apolpp* with eGFP but retained the endogenous *apolpp* 3′ UTR. The ApoLppI C-eGFP transgene is homozygous viable. b) Number line depicts the sequence of polytene region 102D4. The *myo* gene and three *myo* transcripts (from the opposite strand right to left) that encode a single Myo protein are shown. The transcripts/proteins are annotated to show a furin cleavage site between amino acids 476 and 477. In the first intron of the coding region are two FCRP DoubleHeader conversions of CR02262. The *myo* T2A.GAL4 and Myo eGFP transgenes are homozygous lethal. Note the Myo eGFP tag is in the prodomain but also tracks the receptor-binding ligand because the prodomain is attached to the ligand until released prior to receptor binding (Rifkin et al. 2022).

Based on the second instar larval data that ApoLpp has a function in the brain (Brankatschk and Eaton 2010), we examined adult brains for the presence of ApoLpp utilizing three transgenes generated by the FCRP (Stinchfield et al. 2024). Two are endogenous eGFP tagged transgenes with ApoLppII N-eGFP tracking the N-terminal isoform and ApoLppI C-eGFP tracking the C-terminal isoform (possibly also full-length ApoLpp; Brankatschk and Eaton 2010). The third transgene, *apolpp* T2A.GAL4 tracks *apolpp* transcription (Fig. 3).

**Fig. 4.**
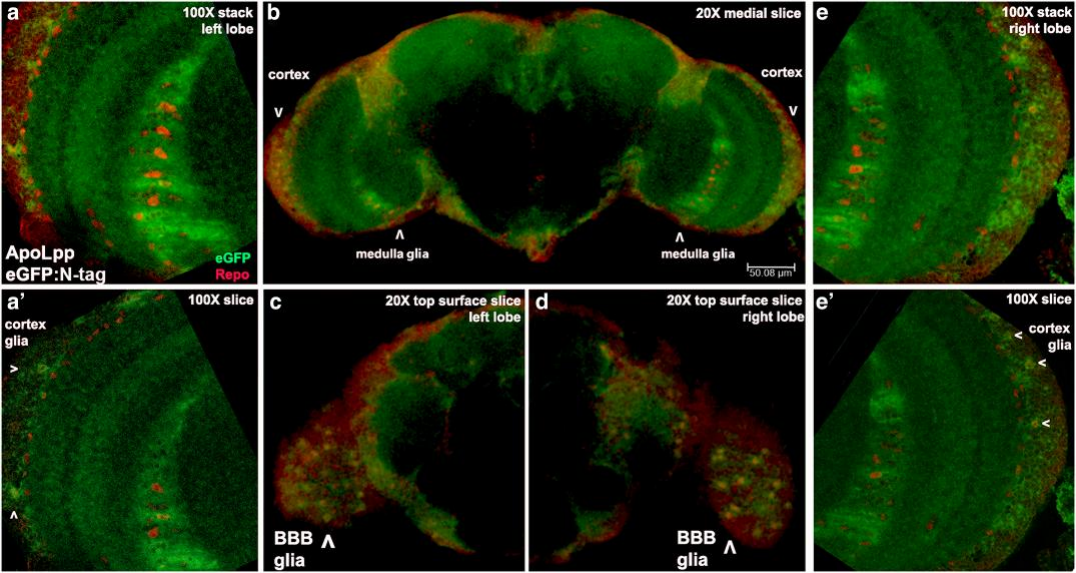
ApoLppII N-eGFP accumulates near BBB, cortex and medulla glia of adult brains. a′) Confocal stack and medial slice of the left optic lobe of an ApoLppII N-eGFP adult male brain (*n* = 4) displaying eGFP (green) and Repo (red). ApoLppII N-eGFP is present throughout the lobe, including near the nuclei of cortex glia (arrowheads in a′) and medulla glia at the right. b) Medial slice of the same brain revealing left-right symmetry for ApoLppII N-eGFP near cortex (down arrowheads) and medulla glia nuclei (up arrowheads). c and d) Slice of the left and right optic lobe anterior surfaces revealing that ApoLppII N-eGFP overlaps the nuclei of BBB glia in both locations (up arrowheads). e–e′) Confocal stack and medial slice of the right optic lobe indicates the same ApoLppII N-eGFP accumulation pattern as the left including the association with the nuclei of cortex (arrowheads in e′) and medulla glia at the left.

Examining eGFP in the adult brain revealed that ApoLppII N-eGFP is widely visible. Low magnification images from brains with glial cells marked by Repo show ApoLppII N-eGFP accumulation in the optic lobe cortex and medulla (Fig. 4). Higher magnification images reveal that ApoLppII N-eGFP is visible overlapping glial nuclei in the BBB. There is also ApoLppII N-eGFP in the underlying optic lobe cortex that contains both neurons and glia. Further medial in the optic lobe medulla, a high concentration of ApoLppII N-eGFP is present around three rows of medulla glia. In these rows are astrocytes (their projections tile neurons in specific area) and ensheathing glia (their projections are neuronal axon wrappers). ApoLppII N-eGFP is also visible apical and medial to the medulla in the lamina and lobula, respectively. In the BBB, cortex, and medulla ApoLppII N-eGFP overlaps a subset of Repo-expressing glial nuclei.

**Fig. 5.**
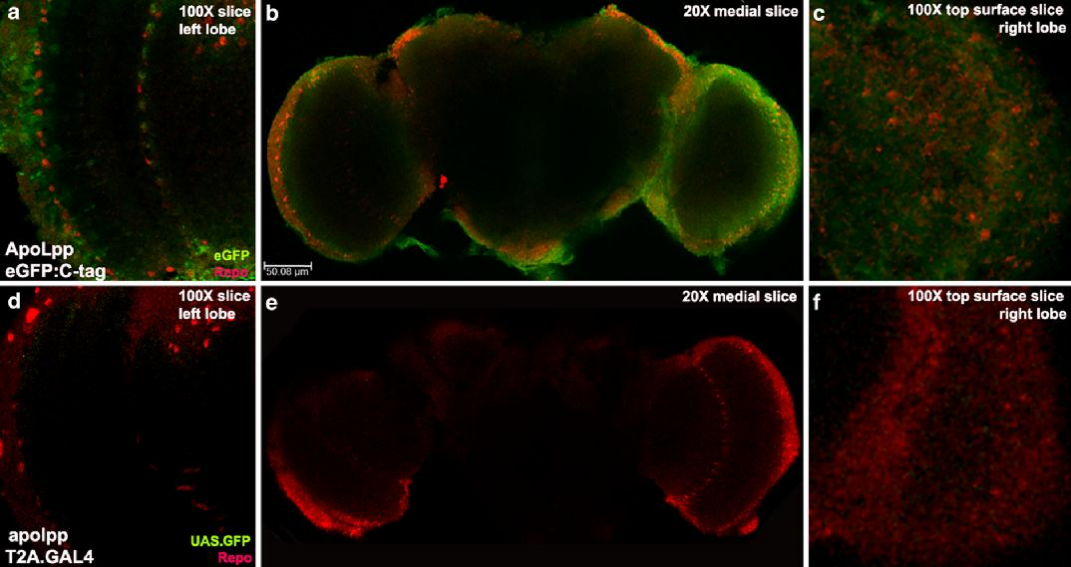
ApoLppI C-eGFP accumulates near BBB, cortex and medulla glia but *apolpp* is not transcribed in adult brains. a to c) ApoLppI C-eGFP adult female brain (*n* = 5) displaying eGFP (green) and Repo (red). a) Medial slice of the left optic lobe showing ApoLppI C-eGFP is prominent in the cortex while modest in the medulla. b) Medial slice of the whole brain revealing left-right symmetry for ApoLppI C-eGFP. c) Slice of the right optic lobe anterior surface showing that ApoLppI C-eGFP is present in BBB glia though not overlapping nuclei like ApoLppII N-eGFP. d to f) *apolpp* T2A.GAL4 heterozygous adult female brain (*n* = 4) expressing UAS.nls.GFP (green) and Repo (red). No GFP is visible. Functionality of *apolpp* T2A.GAL4 was verified via rescue of a transheterozygote with *apolpp^32B^* with two copies of wild type UAS.ApoLpp. The absence of *apolpp* transcription in the adult brain indicates that ApoLppI C-eGFP and ApoLppII N-eGFP in the adult brain are exogenous.

ApoLppI C-eGFP is also present in the adult brain, but appears more restricted than ApoLppII N-eGFP (Fig. 5). ApoLppI C-eGFP transits the BBB and enters the cortex where its deposition pattern resembles ApoLppII N-eGFP. ApoLppI C-eGFP maintains a presence in BBB glia though it does not overlap any nuclei. ApoLppI C-eGFP moves past the cortex into the medulla, but it appears to accumulate only near a single row of glia. In this row ApoLppI C-eGFP overlaps some but not all Repo-expressing medulla glial nuclei. Neither the lamina nor lobula show ApoLppI C-eGFP accumulation.

Then we analyzed adult brains for transcription utilizing *apolpp* T2A.GAL4 (Fig. 5). This transgene identifies cells transcribing the *apolpp* mRNA that leads to both ApoLppII and ApoLppI isoforms. We detect no transcription yet are confident the transgene is functioning due to its ability to reveal *apolpp* transcription in the fat body (Supplementary Fig. 3). The absence of *apolpp* transcription in the adult brain is also consistent with a donor's note for the *apolpp* T2A.GAL4 parent stock (CR80040; BDSC #97179) that little or no GAL4 expression was detected in the larval brain. Taken together multiple lines of evidence suggest that ApoLppII and ApoLppI in the adult brain are exogenous.

### ApoLppII, ApoLppI, and Myo are present in glia in adult brains

**Fig. 6.**
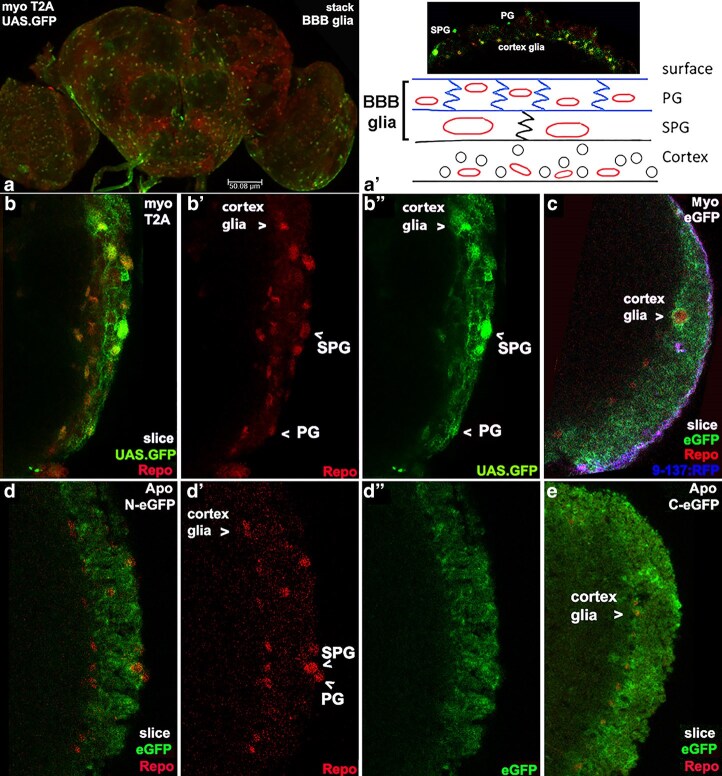
ApoLppII N-eGFP, ApoLppI C-eGFP and Myo are abundant in BBB and cortex glia. a) Confocal stack at low magnification of a *myo* T2A.GAL4 adult female brain (*n* = 4) displaying UAS.nls.GFP (green) with Repo (red).*myo* transcription is visible in many BBB glia. a′) Schematic below a confocal slice of the BBB plus cortex in cross-section from the same brain. The BBB consists of two distinct rows of glia packed tightly together. The perineural glia row (pg) is apical and in contact with the circulation. Medial is the subperineural glia (spg). These two rows can be distinguished empirically by their nuclei size and spacing as shown. Medial to the BBB is the cortex containing primarily neurons with glia; black circles represent neuronal and red circles glial nuclei without cell delineation. In the image many BBB and cortex glia transcribe *myo*. b–b″) Single slice at high magnification of the BBB and cortex from the right brain lobe of same brain in two colors and single channels. Strong *myo* transcription is visible in cortex, perineural and subperineural glia such that nls.GFP overflows into cell projections. c) Slightly lower magnification slice of a Myo eGFP adult female right brain lobe (*n* = 4) displaying eGFP (green), Repo (red) and 9-137:RFP (blue) that marks perineural glia. Myo eGFP is ubiquitous in the cortex reflecting efficient glial secretion. d–d″) Same view of the cortex in a slice from a ApoLppII N-eGFP adult female brain (*n* = 5) reveals abundant eGFP. e) Same view of an ApoLppI C-eGFP adult female brain (*n* = 2) also reveals abundant eGFP.

We then looked with more care at the accumulation patterns of both ApoLpp isoforms in the adult brain using Myoglianin (Myo) as a marker for a subset of glia in the BBB, cortex and medulla. Myo is a secreted protein in the TGF-β family (Wisotzkey and Newfeld 2020). We examined two *myo* transgenes created by the FCRP: *myo* T2A.GAL4 identifies cells transcribing *myo*, and Myo eGFP tracks the Myo protein (Fig. 3). *myo* is visible in many BBB glia, where its transcription appears similar to ApoLppII N-eGFP accumulation (Fig. 6). A closer view of the BBB and cortex reveals that *myo* transcription is so strong that UAS.nls.GFP overflows nuclei into glial projections. Subsequently, Myo eGFP is efficiently secreted from BBB and cortex glia as shown by the absence of accumulation in glial cells. Higher magnification views of ApoLppII N-eGFP and ApoLppI C-eGFP in the cortex show they appear ubiquitous like Myo eGFP. The overall impression is of similarity between Myo and ApoLpp in the BBB and in the cortex.

**Fig. 7.**
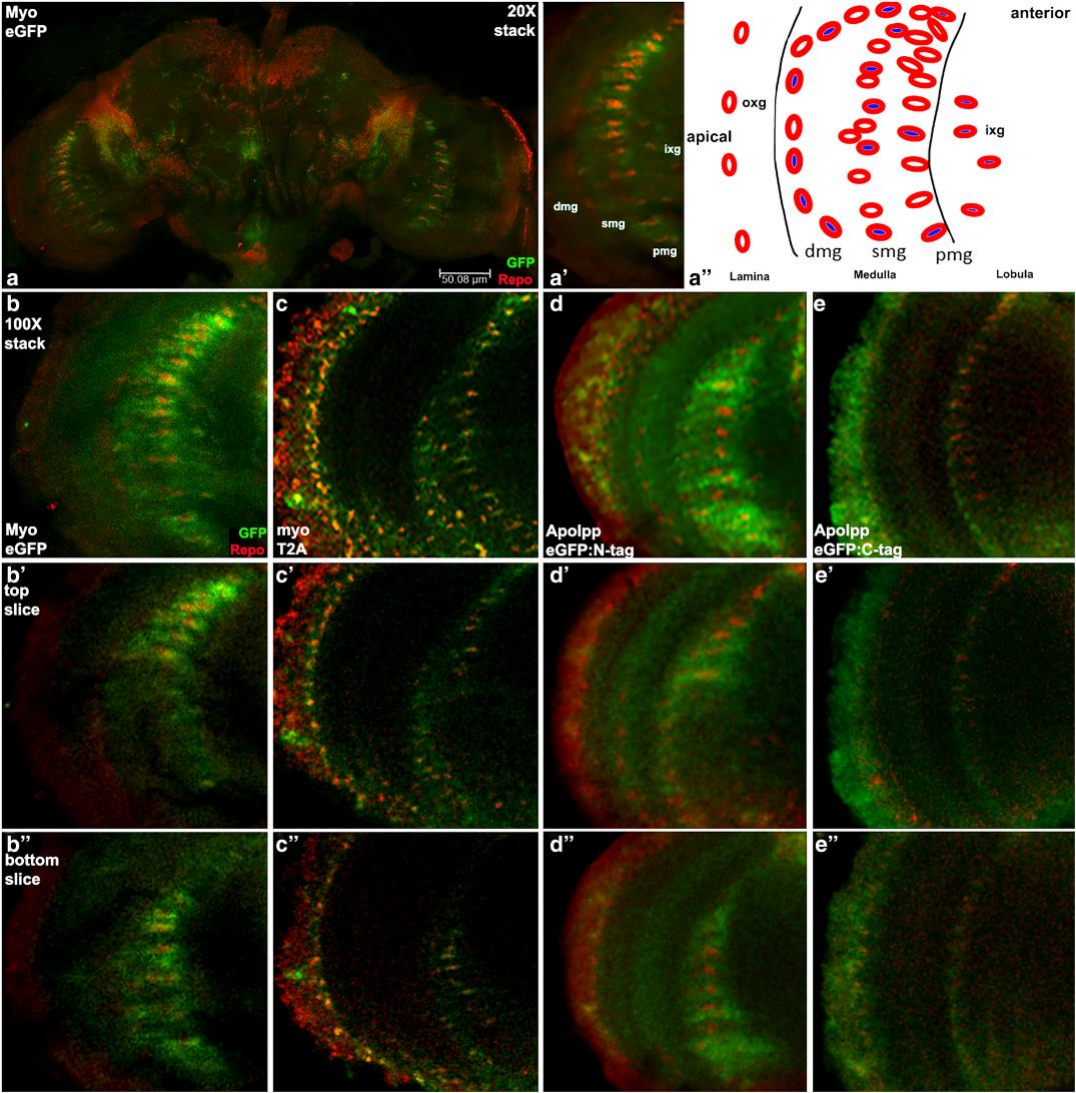
ApoLppII N-eGFP and ApoLppI C-eGFP are near medulla glia expressing Myo. a) Confocal stack at low magnification of a Myo eGFP female brain (*n* = 4) displaying eGFP (green) with Repo (red). Myo eGFP presence is strongest in glia of the medulla but also visible in the lamina and lobula. a′) Single slice at high magnification of the left brain lobe reveals that Myo eGFP is present in a subset of all three rows of medulla glia. a″) Schematic of glial subtypes in the medulla. Three roughly parallel rows of glia (red circles) are aligned from anterior to posterior (image top to bottom). The rows are bunched at the ends and well separated in between. These rows are named from left to right distal (dmg), serpentine (smg) and proximal (pmg). Each row contains two classes of glia. One class (blue center) is astrocytes with projections that tile the neurons in a specific area and function in neuron homeostasis. The other class (white center) is ensheathing glia with nuclei arrayed at the medulla boundary and projections that extend into the medulla where they act as neuronal axon wrappers. Apical to the medulla in the lamina there is an anterior-posterior row of glia called the outer chiasm glia (oxg). Medial to the medulla in the lobula there is a roughly triangular set of glia pointing toward the central brain called the inner chiasm glia (ixg). b–b‴) Confocal stack at high magnification with two slices from a Myo eGFP female brain expressing eGFP (green) and Repo (red). b′) In the dorsal slice, at the top of the medulla glia rows the diffuse nature of Myo eGFP suggests astrocyte expression. b″) In the ventral slice, in the middle of the medulla glia rows Myo eGFP emanates apically in a linear shape suggesting ensheathing glia expression. c–c″) Stack with two slices from a *myo* T2A.GAL4 female brain (*n* = 4) expressing UAS.nls.GFP (green) and Repo (red). *myo* is transcribed in cortex glia and a subset of all three rows of medulla glia. d–d″) Stack with two slices from a ApoLppII N-eGFP male brain (*n* = 4) expressing eGFP (green) and Repo (red). ApoLppII N-eGFP is visible in the cortex, lamina, medulla and lobula with highest abundance near glia in the cortex and medulla. The pattern of ApoLppII N-eGFP medulla expression is similar to Myo eGFP. e–e″) Stack with two slices from a ApoLppI C-eGFP female brain (*n* = 2) expressing eGFP (green) and Repo (red). ApoLppI C-eGFP is strong in the cortex but restricted to the serpentine row of medulla glia.

Within the medulla, Myo eGFP appears in a subset of all three rows of ensheathing glia and astrocytes (Fig. 7). Looking next at *myo* transcription, this is also visible in a subset of all three rows of medulla glia. Comparing Myo eGFP to ApoLppII N-eGFP in the medulla reveals a strikingly similar accumulation pattern. ApoLppII N-eGFP is visible in the cortex and medulla with the highest abundance near the three rows of medulla glia. Similarity between these 2 eGFP transgenes also exists outside the medulla with Myo eGFP visible in the lamina and lobula similar to ApoLppII N-eGFP. On the other hand, ApoLppI C-eGFP in the medulla appears restricted to the serpentine row, with faint expression in the lamina and lobula.

### BBB morphotraps confirm that both isoforms of ApoLpp in the adult brain are exogenous

Taken together the data thus far suggest the hypothesis that ApoLpp isoforms in the adult brain are not expressed there but are instead derived from the fat body after crossing the BBB. We tested this hypothesis in morphotrap experiments. A morphotrap transgene encodes an UAS controlled fusion of a nanobody against GFP to the external portion of the transmembrane protein CD8, that we call CD8-morpho. This transgene effectively traps nearby GFP-tagged secreted proteins at the outer membrane of any expressing cell, thus preventing the secreted protein from traveling further. In our hypothesis test, CD8-morpho was driven in the BBB by 9-137.GAL4 to block ApoLppII N-eGFP and/or ApoLppI C-eGFP at the BBB. CD8-morpho BBB expression was tracked by co-expression of UAS.CD8.RFP. A similar approach employing CD8-morpho and 9-137:RFP was reported in the analysis of an adult BBB enriched drug efflux pump (Hindle et al. 2017). We examined the brains of third instar larvae as well as adult females.

We confirmed that 9-137:RFP expression was restricted to BBB membranes in third instar larvae and adults by examining brain expression in otherwise wild type flies (Supplementary Fig. 4). Then to be sure CD8-morpho driven by 9-137:RFP was able to trap eGFP effectively in third instar larvae we examined ApoLppI C-eGFP homozygotes with and without CD8-morpho. Control larvae without CD8-morpho exhibited eGFP on the BBB and inside the brain. Larvae with CD-8 morpho displayed eGFP on the BBB but no eGFP inside the brain (Supplementary Fig. 5). Based on these observations, CD8-morpho should be effective at blocking eGFP from entering the adult brain.

Examination of one day old virgin female brains expressing CD8-morpho in the BBB and ApoLppII N-eGFP as a heterozygote with *apolpp^81D^* showed ApoLppII N-eGFP uniformly on the surface but not inside the brain (Supplementary Fig. 6). This is the same phenotype seen in larval brains homozygous for ApoLppI C-eGFP with CD8-morpho in the BBB (Supplementary Fig. 5). Data from one day old females is promising but perhaps not conclusive given the extent of neural development and vitellogenesis that occur immediately after eclosion in females (Zhang et al. 2022; Li et al. 2024). As a control, to ensure the exclusion of ApoLppII N-eGFP from the brain was due to CD8-morpho in the BBB, we substituted Nrv-morpho for CD8-morpho. Nrv-morpho is only effective in a polarized epithelium as Nrv is specifically inserted in the basolateral membrane (Harmansa et al. 2017). The BBB is a squamous epithelium and Nrv is not inserted in the membrane. All other features of the genotype were identical to CD8-morpho. In larvae and adults with Nrv-morpho, both ApoLppII N-eGFP and ApoLppI C-eGFP are detected inside the brain (Supplementary Fig. 7).

**Fig. 8.**
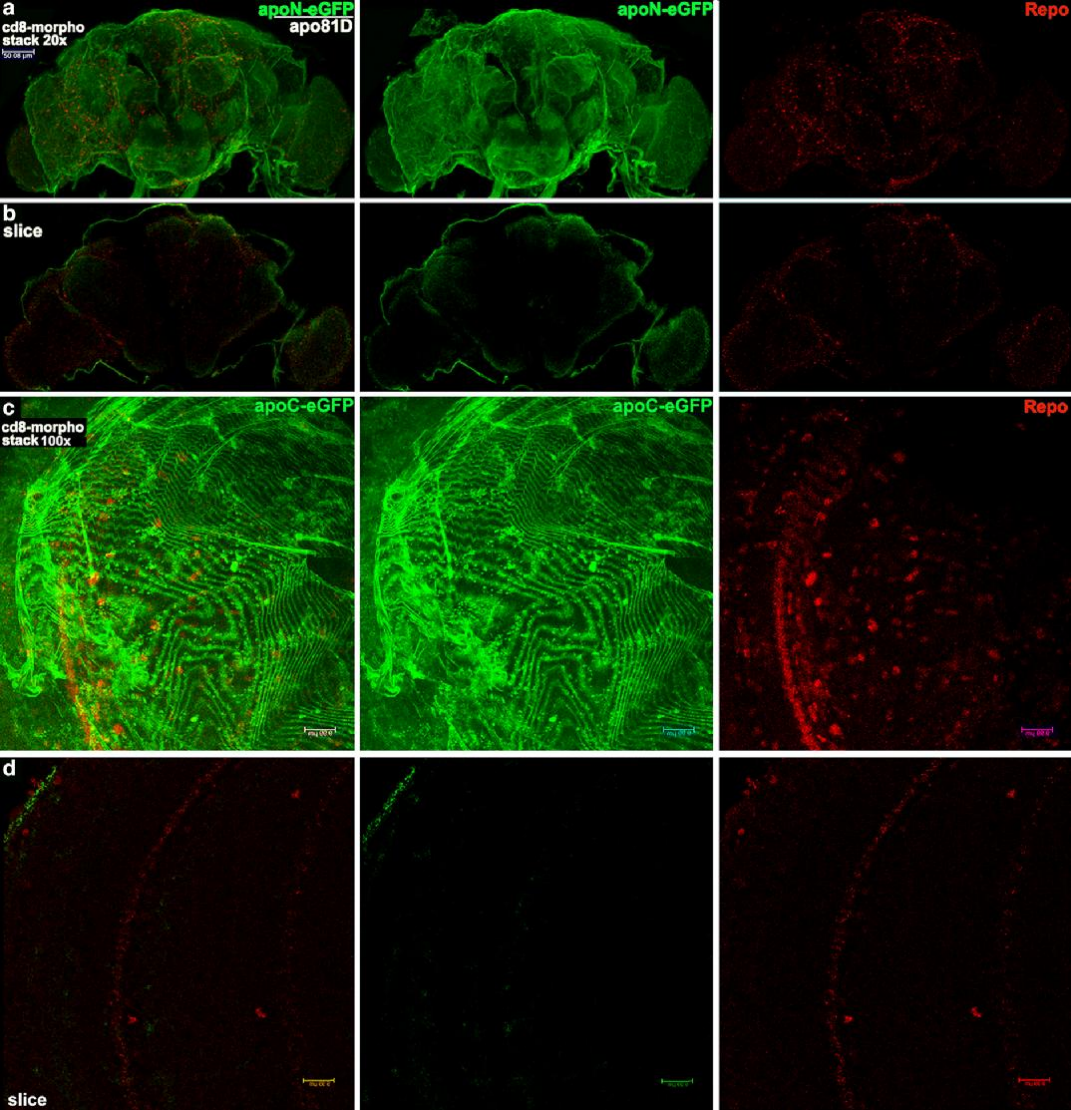
BBB morphotrap in ten day old unmated females shows that ApoLppII N-eGFP and ApoLppI C-eGFP in the adult brain are exogenous. Aged adult female brains (*n* = 3) with CD8-morpho driven by 9-137.GAL4 in the BBB displaying either ApoLppII N-eGFP in a heterozygote with *apolpp^81D^* or homozygous ApoLppI C-eGFP (as indicated in green) and Repo (red) in two colors (left column) and single channels. a) Low magnification confocal stack reveals ApoLppII N-eGFP uniformly distributed on the brain surface. b) Single slice from the same brain shows that ApoLppII N-eGFP is restricted to the brain surface. c) High magnification stack (*n* = 3) from the left brain lobe reveals that ApoLppI C-eGFP is uniformly distributed on the brain surface. d) Single slice from the same lobe shows that ApoLppI C-eGFP is restricted to the brain surface.

We then analyzed CD8-morpho brains from ten day old virgin females to represent aged adults that were expressing ApoLppII N-eGFP in a heterozygote with *apolpp^81D^* or homozygous for ApoLppI C-eGFP (Fig. 8). A low magnification confocal stack shows ApoLppII N-eGFP uniformly on the surface. Low magnification slices reveal that ApoLppII N-eGFP is not visible inside the brain. The impression is reinforced by high magnification stacks and slices of ApoLppI C-eGFP that reflect the same distribution. Taken together the one and ten day old virgin female data suggest that ApoLppII N-eGFP and ApoLppI C-eGFP visible inside the brain in our previous experiments were exogenous.

**Fig. 9.**
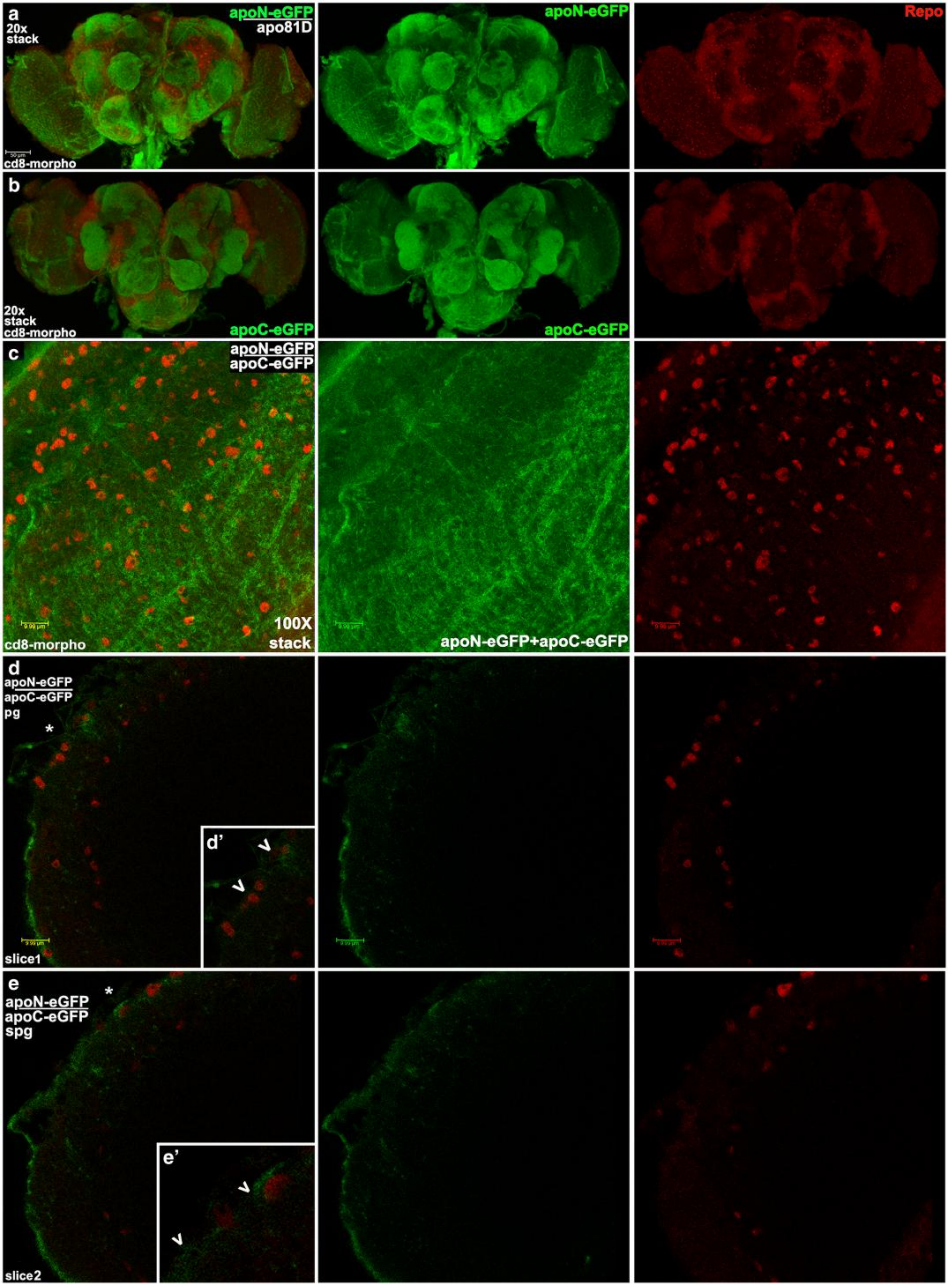
BBB morphotrap in ten day old mated females shows that exogenous ApoLppII N-eGFP and ApoLppI C-eGFP are blocked at the subperineural glia. Aged adult female brains with CD8-morpho driven by 9-137.GAL4 in the BBB displaying isoforms of ApoLpp eGFP as indicated. a and b) Low magnification stacks show that ApoLppII N-eGFP in a heterozygote with *apolpp^81D^* (*n* = 3) and homozygous ApoLppI C-eGFP (*n* = 3) are uniformly distributed on the brain surface. c) High magnification stack of the left lobe of a brain with ApoLppII N-eGFP over ApoLppI C-eGFP (*n* = 3) shows them uniformly distributed on the brain surface. d) Single slice of the same lobe focused on the structural layer of perineural glia (pg). The slice suggests that eGFP is largely restricted to the brain surface but can be seen medial to the perineural glia layer. Starred area shown as an inset. d′) Arrowheads indicate perineural glia whose Repo expressing nuclei are surrounded by eGFP. e) Single slice, dorsal to the previous slice, focused on the subperineural glia row (spg), that constitutes the BBB barrier layer underneath the perineural glia. Starred area shown as an inset. e′) Arrowheads indicate subperineural glia whose Repo expressing nuclei have eGFP primarily on their apical side with modest visibility medially.

Brains from mature ten day old mated CD8-morpho females (mated for three days then gender segregated), were consistent with those of ten day old virgins (Fig. 9). Images from adults expressing ApoLppII N-eGFP in a heterozygote with *apolpp^81D^* or homozygous for ApoLppI C-eGFP or both were examined. Low magnification confocal stacks of brains from ApoLppII N-eGFP or ApoLppI C-eGFP mated females show the same phenotype as their virgin siblings, with eGFP uniformly distributed on the surface. A high magnification confocal stack of a ten day old mated female with ApoLppII N-eGFP and ApoLppI C-eGFP looks virtually identical to the one from a ten day old virgin female, with eGFP uniformly distributed on the surface. A high magnification slice (Fig. 9d) shows that a very small amount of ApoLpp eGFP penetrates a short distance into the brain with most ApoLpp eGFP at the BBB adjacent to perineural glia. The insert shows that perineural glial nuclei are embedded in ApoLpp eGFP. Moving one slice dorsal (Fig. 9e) shows ApoLpp eGFP is barely visible for just a short distance inside the brain with most ApoLpp eGFP at the BBB adjacent to subperineural glia. The insert shows the asymmetric distribution of ApoLpp around subperineural glia with considerably more apically than medially.

Taken together morphotrap data shows that at larval and adult stages both isoforms of ApoLpp eGFP inside the brain are exogenous. This finding is consistent with prior results suggesting that the fat body is an essential source of ApoLpp in larvae and adults.

### ApoLpp expressed in the fat body is secreted then visible on the BBB and inside the brain

We then directly tested the hypothesis that ApoLpp protein in the larval and adult brain is not expressed there but is instead derived from the fat body after crossing the BBB. For these studies we employed three transgenes from the rescue crosses. There are the fat body drivers CG.GAL4, R4.GAL4 and UAS.5′-HA-ApoLpp-Myc-3′. This cDNA rescued *apolpp* mutants at roughly one-third of the Mendelian expectation (Table 2), indicating the tags do not fully interfere with function. This transgene can produce both isoforms: HA tagged ApoLppII N-terminal (5′-HA-ApoLppII) and Myc tagged ApoLppI C-terminal (ApoLppI-Myc-3′). The double-tagged protein tracking experiment was conducted in third instar larvae and adults.

**Fig. 10.**
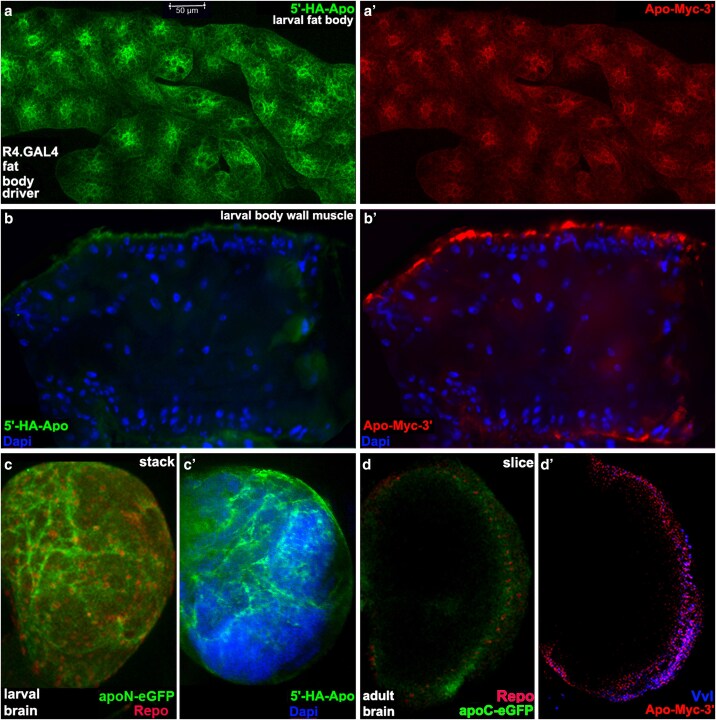
Double tagged ApoLpp tracked from fat body secretion to the BBB then inside the larval and adult brain. a and a′) Dissected larval fat body (*n* = 3) reflecting R4.GAL4 driven UAS.5′-HA-ApoLpp-Myc-3′ shown as single channels. Every cell expresses 5′-HA-ApoLppII (green) and ApolppI-Myc-3′ (red). b–b′) Dissected larval body wall muscle (*n* = 4) with multinucleated cells. 5′-HA-ApoLppII and ApolppI-Myc-3 are visible on the surface with diffuse accrual at the open end (right). c–c′) Larval brain left lobe stacks. c) ApoLppII N-eGFP (green) and Repo (red) displaying ApoLppII N-eGFP at the surface and inside the brain (*n* = 4). c′) CG.GAL4 driven 5′-HA-ApoLppII (green) and Dapi (blue) displaying 5′-HA-ApoLppII at the surface and inside the brain (*n* = 6) similar to ApoLppII N-eGFP. d–d′) Adult brain right lobe slices. d) R4.GAL4 driven ApoLppI C-eGFP (green) and Repo (red) displaying ApoLppI C-eGFP at the surface and inside the brain (*n* = 5). d′) ApoLpp-Myc-3′ (red) and Vvl (blue; marks BBB glial nuclei) displaying ApoLpp-Myc-3′ at the surface and inside the brain (*n* = 3) similar to ApoLppI C-eGFP.

High magnification single color views of dissected larval fat body with R4.GAL4 driving the double-tagged ApoLpp, revealed both 5′-HA-ApoLppII and ApolppI-Myc-3′ are present in every cell (Fig. 10). Same view of a dissected body wall muscle stained similarly plus Dapi showed the highest concentration of 5′-HA-ApoLppII and ApolppI-Myc-3 along the exterior surface of the muscle. There is a small amount of diffuse accumulation of each inside the muscle near the right side cut site. The distribution of 5′-HA-ApoLppII and ApolppI-Myc-3′ on the muscle suggests that both isoforms of ApoLpp were secreted by the fat body into the circulation then traveled to the muscle exterior surface.

Low magnification stack of a larval brain lobe stained with 5′-HA-ApoLppII and Dapi contains 5′-HA-ApoLppII accumulation on the surface and inside the brain. The distribution of 5′-HA-ApoLppII appears similar to ApoLppII N-eGFP in the larval brain. A high magnification confocal slice of an adult brain lobe reflecting ApoLppI-Myc-3′ and the BBB glial nuclear marker Vvl contains ApoLppI-Myc-3′ accumulation on the surface and inside the brain. The distribution of ApoLppI-Myc-3′ appears similar to ApoLppI C-eGFP in the adult brain. The double-tagged ApoLpp tracking data, together with the morphotrap, rescue and RNAi data strongly support the hypothesis that ApoLpp protein in the larval and adult brain is not expressed there but is instead derived from the fat body after crossing the BBB.

## Discussion

The Apolipoprotein (Apo) family, in humans and other species is not a traditional protein family where all members share sequence similarity derived from a common ancestor. As a result, the evolutionary relationships between Apo proteins overall and between ApoB and ApoLpp have not been clearly established. Our interspecific tree connected two human Apo proteins to fly proteins (ApoB48 with ApoLppII/ApoLTPII and ApoF with Fabp). This connection indicated that these proteins were conserved since the last common ancestor of mammals and flies. A second interspecific tree connected fly ApoLppII/ApoLTPII to nematode Vit-6II. Together the interspecific trees suggest a connection between human ApoB48, fly ApoLppII and nematode Vit6II in their common ancestor nearly 700 million years ago.

To extend our understanding of ApoLpp in flies beyond second instar and suggest new hypotheses for human ApoB, we conducted a series of experiments with new stocks from the FCRP to visualize endogenous *apolpp* transcription and ApoLpp isoform specific protein accumulation. In the rescue experiments, UAS.ApoLpp expressed in the fat body in an *apolpp* lethal genotype rescued these individuals to adulthood. Companion *apolpp*.RNAi experiments employing the same fat body drivers, eliminated larval survival. Taken together these experiments indicate that the fat body is a necessary and sufficient source of *apolpp* for survival to adult. Studies of *apolpp* transcription are consistent, with no expression seen in the larval or adult brain. Nevertheless, both isoforms of Apolpp eGFP were visible in the larval and adult brain. ApoLpp brain accumulation resembled *myo* transcription/translation in the adult BBB, cortex and medulla suggesting an association of ApoLpp eGFP with adult brain glia and *myo*.

We directly tested the hypothesis that ApoLpp protein in the larval and adult brain is not expressed there but is instead derived from the fat body after crossing the BBB in two sets of experiments. The first set employed a morphotrap expressed in the BBB to block circulating ApoLppII N-eGFP or ApoLppI C-eGFP from entering the brain. When the morphotrap was present, neither ApoLppII N-eGFP nor ApoLppI C-eGFP were visible in third instar or adult brains, confirming that ApoLpp protein in the larval and adult brain is exogenous. The second set showed that the source of the exogenous ApoLpp protein in the brain is the fat body. Double-tagged UAS.5′-HA-ApoLpp-Myc-3′ was expressed exclusively in the fat body and tracked. Imaging showed both isoforms were expressed in the fat body, secreted into the circulation, then visible on the BBB surface and inside the brain. Double tagged ApoLpp results confirm the hypothesis that ApoLpp protein in the larval and adult brain is not expressed there but is instead derived from the fat body after crossing the BBB.

Our ApoLpp adult data extends the prior ApoLpp functional study in second instar (Brankatschk and Eaton 2010). Our adult data showing accumulation of ApoLpp near glia is consistent with a recent report employing RNAi to suggest that ApoLpp from the fat body enters the adult brain where it plays a role in glial phagocytosis in response to neuronal debris created by physical injury (Alassaf et al. 2025). Our adult data adds a third signal to recent reports of fat body/gut to brain signaling. Two examples are Vaha (a secretory lipase) and Neuropeptide F that are secreted by the gut, then cross the adult BBB to target insulin producing neurons (Chen et al. 2024; Singh et al. 2024).

The possibility that ApoLpp is a fat body to brain signal, in addition to delivering lipids, suggested two potential relationships between ApoLpp and *myo* in brain glia. Perhaps ApoLpp delivers lipids to *myo* expressing glia and/or *myo* expressing glia receive an ApoLpp signal from the fat body. In the latter case, it is tempting to speculate that Myo secreted by brain glia then plays the role of GLP-1 in signaling from the hypothalamus to the liver and gut, to regulate ApoB48 expression/secretion (Farr et al. 2015). Since we have shown ApoLpp travels from the fat body to the brain, if Myo from the brain then fulfilled the role of GLP-1 in signaling back to the fat body, it would suggest a feedback loop regulates ApoLpp, and perhaps also ApoB, expression/secretion.

Taken together our ApoLpp experimental data and evidence of conservation between ApoB48 and ApoLppII suggest the new hypothesis that human ApoB48 exits the circulation to fulfill a function in the brain. This hypothesis is supported by several circumstantial factors. First, there are many established similarities between the fly and mammal BBB (e.g. Desalvo et al. 2014; Contreras and Klämbt 2023). Second, ApoB mutations or serum levels are associated with features of cognitive impairment that are not readily connected to atherosclerosis. Third, studies of rodent cells in culture suggest a possible mechanism for ApoB BBB transit via LDL receptor transcytosis. This mechanism is similar to reports of ApoLTP passage through the BBB via endocytosis of its transporters LRP1 and LRP2 (Li et al. 2023; Singh et al. 2024).

Experimental evidence of ApoB inside the brain of a vertebrate derives from two studies in zebrafish, an organism that recapitulates human ApoB physiology including exclusive expression in the liver/gut (Schlegel 2016). The first paper employed a luciferase enzyme fused to endogenous ApoBb.1 to monitor accumulation in live larvae with specific genetic backgrounds under defined nutritional regimes. The authors noted ApoBb.1 presence in the brains of both wild type and experimental larvae (Thierer et al. 2019). The second paper employed the photoconvertible fluorophore Dendra2 fused to endogenous ApoBb.1 to monitor ApoBb.1 half-life in circulation in live wild type and juveniles with the same set experimental regimes. The authors again noted ApoBb.1 accumulation in the brain. Further, the photoconversion state of ApoBb.1 in the brain suggested to the authors that an LDL binding domain protein was capturing ApoBb.1 after BBB transit (Moll et al. 2025). Reports from humans, rodents, zebrafish and now flies are consistent with the hypothesis that ApoB (or perhaps only ApoB48) crosses the BBB to fulfill a function in the brain.

In summary, we advance our knowledge of ApoLpp function to third instar and adult brains while demonstrating strong conservation with ApoB. Imaging studies in the adult brain employed new tagged proteins to reveal that both ApoLpp isoforms are present near glia. Morphotraps that block tagged ApoLpp at the BBB demonstrated that ApoLpp in the adult brain is exogenous. A double tagged version of ApoLpp expressed solely in the fat body was tracked from fat body secretion to the BBB and into the adult brain. Overall, our data suggest that the previously documented role of ApoLpp in second instar larval brain lipid delivery is likely operating in third instar and adults. Strong conservation between ApoLppII and ApoB48 supports the hypothesis that ApoB48 may have a role in the brain outside the circulatory system.

## Supplementary Material

iyaf224_Supplementary_Data

## Data Availability

Strains are available at the Bloomington Drosophila Stock Center (Cook and Parks 2022) and described in Flybase (Öztürk-Çolak et al. 2024). The authors affirm that all data underlying the conclusions are present within the article, figures, tables and supplementary material. Supplemental material available at GENETICS online.

## References

[iyaf224-B1] Alassaf M et al 2025. Adipocyte metabolic state regulates glial phagocytic function. Cell Rep. 44:115704. 10.1016/j.celrep.2025.115704. 40372917 PMC12169346

[iyaf224-B2] Asha H et al 2003. Analysis of Ras-induced overproliferation in Drosophila hemocytes. Genetics. 163:203–215. 10.1093/genetics/163.1.203. 12586708 PMC1462399

[iyaf224-B3] Aumont-Rodrigue G, Picard C, Labonté A, Poirier J. 2024. Apolipoprotein B gene expression and regulation in relation to Alzheimer's disease pathophysiology. J Lipid Res. 65:100667. 10.1016/j.jlr.2024.100667. 39395793 PMC11602985

[iyaf224-B4] Baker M . 1988. Is vitellogenin an ancestor of apolipoprotein B-100 of human low-density lipoprotein and human lipoprotein lipase? Biochem J. 255:1057–1060. 10.1042/bj2551057. 3145737 PMC1135349

[iyaf224-B5] Beheshti S, Madsen C, Varbo A, Nordestgaard B. 2020. Worldwide prevalence of familial hypercholesterolemia. J Am Coll Cardiol. 75:2553–2566. 10.1016/j.jacc.2020.03.057. 32439005

[iyaf224-B6] Brankatschk M, Dunst S, Nemetschke L, Eaton S. 2014. Delivery of circulating lipoproteins to specific neurons in the Drosophila brain regulates systemic insulin signaling. Elife. 3:e02862. 10.7554/eLife.02862. 25275323 PMC4210815

[iyaf224-B7] Brankatschk M, Eaton S. 2010. Lipoprotein particles cross the blood-brain barrier in *Drosophila*. J Neurosci. 30:10441–10447. 10.1523/JNEUROSCI.5943-09.2010. 20685986 PMC6634680

[iyaf224-B9] Chen J, Nouzová M, Noriega FG, Tatar M. 2024. Gut-to-brain regulation of Drosophila aging through neuropeptide F, insulin, and juvenile hormone. Proc Natl Acad Sci U S A. 121:e2411987121. 39413128 10.1073/pnas.2411987121 PMC11513968

[iyaf224-B8] Chen S et al 1986. The complete cDNA and amino acid sequence of human apolipoprotein B-100. J Biol Chem. 261:12918–12921. 10.1016/S0021-9258(18)69248-8. 3759943

[iyaf224-B10] Contreras E, Klämbt C. 2023. The Drosophila blood-brain barrier emerges as a model for understanding human brain diseases. Neurobiol Dis. 180:106071. 10.1016/j.nbd.2023.106071. 36898613

[iyaf224-B901] Cook KR, Parks AL. 2022. The international exchange of *Drosophila melanogaster* strains. Rev Sci Tech. 41:82–90. 10.20506/rst.41.1.3305. 35925634 PMC10116490

[iyaf224-B11] Dehouck B et al 1997. A new function for the LDL receptor: transcytosis of LDL across the blood-brain barrier. J Cell Biol. 138:877–889. 10.1083/jcb.138.4.877. 9265653 PMC2138047

[iyaf224-B12] DeSalvo M et al 2014. Drosophila surface glia transcriptome: conserved blood-brain barrier processes. Front Neurosci. 8:346. 10.3389/fnins.2014.00346. 25426014 PMC4224204

[iyaf224-B13] Dhar A, Minin V. 2016. Maximum likelihood methods for phylogenetic inference. In: Kliman R, editor. Encyclopedia of evolutionary biology. Academic Press. p. 499–506.

[iyaf224-B14] Dupont S et al 2009. FAM/USP9X, a deubiquitinating enzyme essential for TGF-β signaling controls Smad4 monoubiquitination. Cell. 136:123–135. 10.1016/j.cell.2008.10.051. 19135894

[iyaf224-B15] Edgar R . 2004. MUSCLE: multiple sequence alignment with high accuracy and high throughput. Nucleic Acids Res. 32:1792–1797. 10.1093/nar/gkh340. 15034147 PMC390337

[iyaf224-B16] Farr S et al 2015. Central nervous system regulation of intestinal lipoprotein metabolism by glucagon-like peptide-1. Arterioscler Thromb Vasc Biol. 35:1092–1100. 10.1161/ATVBAHA.114.304873. 25675997

[iyaf224-B17] Felsenstein J . 2004. Inferring phylogenies. Sinauer Associates.

[iyaf224-B18] Goldsmith S, Newfeld S. 2023. Dsmad2 differentially regulates dILP2 and dILP5 in insulin producing and circadian pacemaker cells in unmated adult females. PLoS One. 18:e0280529. 10.1371/journal.pone.0280529. 36689407 PMC9870127

[iyaf224-B19] Gong J, Harris K, Peters S, Woodward M. 2022. Serum lipid traits and the risk of dementia: study of 254,575 women and 214,891 men in the UK Biobank. eClinicalMedicine. 54:101695. 10.1016/j.eclinm.2022.101695. 36247924 PMC9561731

[iyaf224-B20] Gratz S et al 2013. Genome engineering of *Drosophila* with the CRISPR RNA-guided Cas9 nuclease. Genetics. 194:1029–1035. 10.1534/genetics.113.152710. 23709638 PMC3730909

[iyaf224-B21] Harmansa S, Alborelli I, Bieli D, Caussinus E, Affolter M. 2017. A nanobody-based toolset to investigate the role of protein localization and dispersal in Drosophila. Elife. 6:e22549. 28395731 10.7554/eLife.22549 PMC5388529

[iyaf224-B22] Harmansa S, Hamaratoglu F, Affolter M, Caussinus E. 2015. Dpp spreading is required for medial but not for lateral wing disc growth. Nature. 527:317–322. 26550827 10.1038/nature15712

[iyaf224-B23] Higuchi K et al 1988. Human ApoB mRNA: identification of two distinct ApoB mRNAs, ApoB-100 and an ApoB mRNA containing a premature in-frame translational stop codon, in both liver and intestine. ProcNatl Acad Sci USA. 85:1772–1776. 10.1073/pnas.85.6.1772. PMC279861 2450346

[iyaf224-B24] Hindle S et al 2017. Evolutionarily conserved roles for blood-brain barrier xenobiotic transporters in endogenous steroid partitioning and behavior. Cell Rep. 21:1304–1316. 29091768 10.1016/j.celrep.2017.10.026 PMC5774027

[iyaf224-B25] Jung-Testas I et al 1992. Low density lipoprotein-receptors in primary cultures of rat glial cells. Steroid Biochem Mol Biol. 42:597–605. 10.1016/0960-0760(92)90450-W. 1637723

[iyaf224-B26] Kanca O et al 2022. An expanded toolkit for Drosophila gene tagging using synthesized homology donor constructs for CRISPR-mediated homologous recombination. Elife. 11:e76077. 10.7554/eLife.76077. 35723254 PMC9239680

[iyaf224-B27] Konikoff C, Wisotzkey R, Newfeld S. 2008. Lysine conservation and context in TGF-β and Wnt signaling suggests new targets and general themes for post-translational modification. J Mol Evol. 67:323–333. 10.1007/s00239-008-9159-4. 18797952 PMC2688696

[iyaf224-B28] Kumar S et al 2022. TimeTree 5: an expanded resource for species divergence times. Mol Biol Evol. 39:msac174. 10.1093/molbev/msac174. 35932227 PMC9400175

[iyaf224-B29] Kumar S, Stecher G, Li M, Knyaz C, Tamura K. 2018. MEGAX: molecular evolutionary genetics analysis across computing platforms. Mol Biol Evol. 35:1547–1549. 10.1093/molbev/msy096. 29722887 PMC5967553

[iyaf224-B30] Lee M et al 2023. High ApoB/ApoA-I ratio predicts post-stroke cognitive impairment in patients with large artery atherosclerosis. Nutrients. 15:4670. 10.3390/nu15214670. 37960323 PMC10648714

[iyaf224-B31] Lee G, Park J. 2004. Hemolymph sugar homeostasis and starvation-induced hyperactivity affected by genetic manipulations of the adipokinetic hormone-encoding gene in Drosophila. Genetics. 167:311–323. 10.1534/genetics.167.1.311. 15166157 PMC1470856

[iyaf224-B32] Li F, Artiushin G, Sehgal A. 2023. Modulation of sleep by trafficking of lipids through the Drosophila blood-brain barrier. Elife. 12:e86336. 10.7554/eLife.86336. 37140181 PMC10205086

[iyaf224-B51] Li J, Ning C, Liu Y, Deng B, Wang B, Shi K, Wang R, Fang R, Zhou C. 2024. The function of juvenile-adult transition axis in female sexual receptivity of *Drosophila melanogaster*. Elife. 12:RP92545. 39240259 10.7554/eLife.92545 PMC11379460

[iyaf224-B33] Liu L, MacKenzie K, Putluri N, Maletić-Savatić M, Bellen H. 2017. The glia-neuron lactate shuttle and elevated ROS promote lipid synthesis in neurons and lipid droplet accumulation in glia via APOE/D. Cell Metab. 26:719–737. 10.1016/j.cmet.2017.08.024. 28965825 PMC5677551

[iyaf224-B34] Luo L, Liao Y, Jan L, Jan Y. 1994. Distinct functions of similar small GTPases: Drosophila Drac1 is involved in axonal outgrowth and myoblast fusion. Genes Dev. 8:1787–1802. 10.1101/gad.8.15.1787. 7958857

[iyaf224-B35] Meschi E, Delanoue R. 2021. Adipokine and fat body in flies: connecting organs. Mol Cell Endocrinol. 533:111339. 10.1016/j.mce.2021.111339. 34082046

[iyaf224-B36] Moll TOC, Kiefer JG, Klemek ML, Wilson MH, Farber SA . 2025. Directly measuring atherogenic lipoprotein kinetics in zebrafish with the photoconvertible LipoTimer reporter. Arterioscler Thromb Vasc Biol. 45:1762–1783. 10.1161/ATVBAHA.125.322969 PMC12597096 40836916

[iyaf224-B902] Öztürk-Çolak A et al 2024. FlyBase: updates to the *Drosophila* genes and genomes database. Genetics. 227:iyad211. 10.1093/genetics/iyad211. 38301657 PMC11075543

[iyaf224-B37] Palm W et al 2012. Lipoproteins in Drosophila: assembly, function, and influence on tissue lipid composition. PLoS Genet. 8:e1002828. 10.1371/journal.pgen.1002828. 22844248 PMC3406001

[iyaf224-B38] Rifkin D et al 2022. The role of LTBPs in TGF-β signaling. Dev Dyn. 251:95–104. 10.1002/dvdy.331. 33742701

[iyaf224-B39] Schlegel A . 2016. Zebrafish models for dyslipidemia and atherosclerosis research. Front Endocrinol. 7:159. 10.3389/fendo.2016.00159. PMC5159437 28018294

[iyaf224-B40] Schuster C, Davis G, Fetter R, Goodman C. 1996. Fasciclin II controls synaptic stabilization and growth. Neuron. 17:641–654. 10.1016/S0896-6273(00)80197-X. 8893022

[iyaf224-B41] Singh A et al 2024. A nutrient responsive lipase mediates gut-brain communication to regulate insulin secretion in Drosophila. Nat Commun. 15:4410. 10.1038/s41467-024-48851-8. 38782979 PMC11116528

[iyaf224-B42] Stinchfield M et al 2024. Fourth Chromosome Resource Project: a comprehensive resource for genetic analysis in Drosophila that includes humanized stocks. Genetics. 226:iyad201. 10.1093/genetics/iyad201. 37981656 PMC10847715

[iyaf224-B43] Tanaka T, Tani N, Nakamura A. 2021. Receptor-mediated yolk uptake is required for oskar mRNA localization and cortical anchorage of germ plasm components in the Drosophila oocyte. PLoS Biol. 19:e3001183. 10.1371/journal.pbio.3001183. 33891588 PMC8064586

[iyaf224-B44] Thierer J, Ekker S, Farber S. 2019. The LipoGlo reporter system for sensitive and specific monitoring of atherogenic lipoproteins. Nat Commun. 10:3426. 10.1038/s41467-019-11259-w. 31366908 PMC6668417

[iyaf224-B45] Tran N et al 2018. *CORL* expression and function in insulin producing neurons reversibly influences adult longevity in *Drosophila*. G3 (Bethesda). 8:2979–2990. 10.1534/g3.118.200572. 30006413 PMC6118311

[iyaf224-B46] Ugur B, Chen K, Bellen H. 2016. *Drosophila* tools and assays for the study of human diseases. Dis Model Mech. 9:235–244. 10.1242/dmm.023762. 26935102 PMC4833332

[iyaf224-B47] Wisotzkey R, Newfeld S. 2020. TGF-β prodomain alignments reveal unexpected cysteine conservation consistent with phylogenetic predictions of cross-subfamily heterodimerization. Genetics. 214:447–465. 10.1534/genetics.119.302255. 31843757 PMC7017013

[iyaf224-B48] Wodarz A, Hinz U, Engelbert M, Knust E. 1995. Expression of crumbs confers apical character on plasma membrane domains of ectodermal epithelia of Drosophila. Cell. 82:67–76. 10.1016/0092-8674(95)90053-5. 7606787

[iyaf224-B49] Zhang C, Kim AJ, Rivera-Perez C, Noriega FG, Kim YJ. 2022. The insect somatostati pathway gates vitellogenesis progression during reproductive maturation and the post-mating response. Nat Commun. 3:969. 10.1038/s41467-022-28592-2 PMC8857180 35181671

