# Supplementary material for: Both isoforms of Drosophila ApoLpp (ApoB) cross the blood–brain barrier in adults
Source: Genetics. 2025 Oct 16;232(1):iyaf224. doi: 10.1093/genetics/iyaf224 (PMC12774823; doi:10.1093/genetics/iyaf224)
Supplement: iyaf224_Supplementary_Data [file iyaf224_supplementary_data.pdf]

Supplemental Data: Three Tables and Twelve Figures

Table S1. Twenty-three human Apo family members identified by name in Uniprot.

Protein	Longest RefSeq	HGNC	Size	Secreted?	pfam structural domains
ApoA1	NP_001412021	600	158	Secreted	pfam01442 - A1/A4/E family
ApoA2	NP_001634	601	100	Secreted	pfam04711 - ApoA2 family
ApoA4	NP_000473	602	396	Secreted	pfam01442 - A1/A4/E family
ApoA5	NP_001358833	17288	366	Secreted	pfam01442 - A1/A4/E family
ApoB[a]	NP_000375	603	4563	Secreted	N-term: pfam09172 - Vitellogenin-N
				Secreted	C-term: pfam06316 - Porin channels
Two true isoforms with common N-term; ApoB48 is 2180aa and ApoB100 is full length					
Alignments and trees employ the two parts of ApoB100 as distinct sequences (ApoB48 and ApoB52)					
ApoC1	NP_001366616	607	129	Secreted	pfam04691 - ApoC1 family
ApoC2	NP_000474	609	101	Secreted	pfam05355 - ApoC2 family
ApoC3	NP_000031	610	99	Secreted	pfam05778 - ApoC3 family
ApoC4	NP_001637	611	127	Secreted	pfam15119 - ApoC4 family
ApoD	AAB32200	612	266[b]	Secreted	pfam04711 - ApoA2 family
ApoE2	NP_000032	613	317	Secreted	pfam01442 - A1/A4/E family
ApoF	NP_001629	615	326	Secreted	pfam15148 - ApoF family
ApoH	NP_000033	616	345	Secreted	pfam09014 - Sushi2 (6Cys-CCP)
ApoJ	NP_001822	2095	449	Secreted	pfam01093 - Clusterin
ApoL1	NP_001130012	618	398	Secreted	pfam05461 - ApoL family
ApoL2	NP_663612	619	337	Cytoplasm	pfam05461 - ApoL family
ApoL3	NP_001380522	14868	331	Cytoplasm	pfam05461 - ApoL family
ApoL4	XP_054181922	14867	348	Predicted	pfam05461 - ApoL family
ApoL5	NP_085145	14869	433	Cytoplasm	pfam05461 - ApoL family
ApoL6	NP_085144	14870	343	Cytoplasm	pfam05461 - ApoL family
ApoM	NP_061974	13916	188	Secreted-Membrane	pfam00061 - Lipocalin
ApoO	NP_077027	28727	198	Mito Inner Membrane	pfam09769 - ApoO (MICOS 26)
ApoOL	NP_940852.	24009	268	Mito Inner Membrane	pfam09769 - ApoO (MICOS 27)
ApoP	an apoptotic protein				
outgroup (similar function but not Apo name)					
MTTP	NP_000244	7467	894	Secreted-ER	pfam19444 - MTP lipid-binding

a. Red indicates that ApoB48 (N-terminal) has the same Vitellogenin-N domain as fly ApoLppII.

b. AAB32200 does not contain the first 20 amino acids. These came from CAG33119.

Table S2. Eight fly Apo family members identified via DIOPT in Flybase.				
**Protein**	**Longest RefSeq**	**Size**	**Secreted?**	**pfam structural domains**
ApoLpp[a]	NP_001284721	3351	Secreted-Cleaved	N-term: pfam09172 - Vitellogenin-N<br>C-term: pfam00094 - vonWillebrand typeD
Two isoforms separated by furin cleavage @ 706/707aa: ApoLppII is N-term and ApoLppI is C-term<br>Alignments and trees utilized each isoform as a distinct sequence				
ApoLTP	NP_995670	4333	Secreted-Cleaved	N-term: pfam09172 - Vitellogenin-N<br>C-term: pfam00094 - vonWillebrand typeD
Two isoforms separated by furin cleavage @ 779/780aa: ApoLTPII is N-term and ApoLTPI is C-term<br>Alignments and trees utilized each isoform as a distinct sequence				
Cv-d<br>No obvious furin cleavage site	NP_732076	1470	Secreted	N-term: pfam09172 - Vitellogenin-N<br>C-term: pfam00061 - Lipocalin
Glaz	NP_523727	212	Secreted	pfam00061 - Lipocalin
Nlaz	NP_001259867	245	Secreted	pfam00061 - Lipocalin
CG31659	NP_722702	192	Secreted	pfam00061 - Lipocalin
Fabp	NP_001027179	157	Cytoplasm	pfam00061 - Lipocalin
Mic26-27	NP_650523	226	Mito Inner Membrane	pfam09769 - ApoO
outgroup (homolog of human outgroup by BLASTp)				
Mtp	NP_610075	886	Secreted-ER	pfam19444 - MTP lipid-binding

a. Red indicates that ApoLppII alone contains the same N-terminal Vitellogenin-N domain as ApoB48.

Table S3. Nine nematode Apo family members identified via Wormbase.				
**Protein**	**Longest RefSeq**	**Size**	**Secreted?**	**pfam structural domains**
Vitellogenin-1	NP_001367671	1616	Secreted predicted.	N-term: pfam09172- Vitellogenin-N<br>C-term: pfam00094 - vonWillebrand typeD
			predicted furin cleavage @ 740-741aa: RGR^R	
For all Vit proteins, alignments and trees employ each predicted isoform as a distinct sequence				
Vit-2	NP_508868	1613	Secreted predicted.	N-term: pfam09172 - Vitellogenin-N<br>C-term: pfam00094 - vonWillebrand typeD
			predicted furin cleavage @ 738-739aa: RGR^R	
Vit-3	NP_001367903	1603	Secreted predicted	N-term: pfam09172- Vitellogenin-N<br>C-term: pfam00094 - vonWillebrand typeD
			predicted furin cleavage @ 735-736 aa: RSR^R	
Vit-4	NP_508612	1603	Secreted predicted.	N-term: pfam09172 - Vitellogenin-N<br>C-term: pfam00094 - vonWillebrand typeD
			predicted furin cleavage @ 735-736aa: RSR^R	
Vit-5	NP_508589	1603	Secreted predicted.	N-term: pfam09172 - Vitellogenin-N<br>C-term: pfam00094 - vonWillebrand typeD
			predicted furin cleavage @ 735-736aa: RSR^R	
Vit-6[a]	NP_001023274	1650	Secreted predicted.	N-term: pfam09172 - Vitellogenin-N<br>C-term: pfam00094 - vonWillebrand typeD
predicted furin cleavage @ 766-767aa: RGK^R site is conserved in *Pristionchus pacificus* Vit-6				
Moma-1	NP_497275	201	Mito Inner Membrane	pfam09769 - ApoO (MICOS 27)
ApoL1	NP_740988	265	Cytoplasm	pfam05461 - ApoL<br>(not likely homolog as human ApoL1 is secreted)
ApoL3-like	NP_001379858	318	Cytoplasm	pfam05461 - ApoL<br>(possible homolog as human ApoL3 is cytoplasmic)
outgroup (similar function but not homolog of either human or fly outgroup by reciprocal BLASTp)				
Dsc-4	NP_499903	892	Secreted-ER	pfam19444 - MTP lipid-binding

a. Red indicates that BLASTp shows Vit-6 is the original member of the Vit family in *C. elegans* with the other Vit proteins derived from serial duplications. As a result, all six *C. elegans* Vit proteins contain a predicted furin cleavage site conserved in ApoLpp and ApoLTP that creates two isoforms and their N-terminal isoforms contain the same Vitellogenin-N domain as ApoLppII and ApoB48.

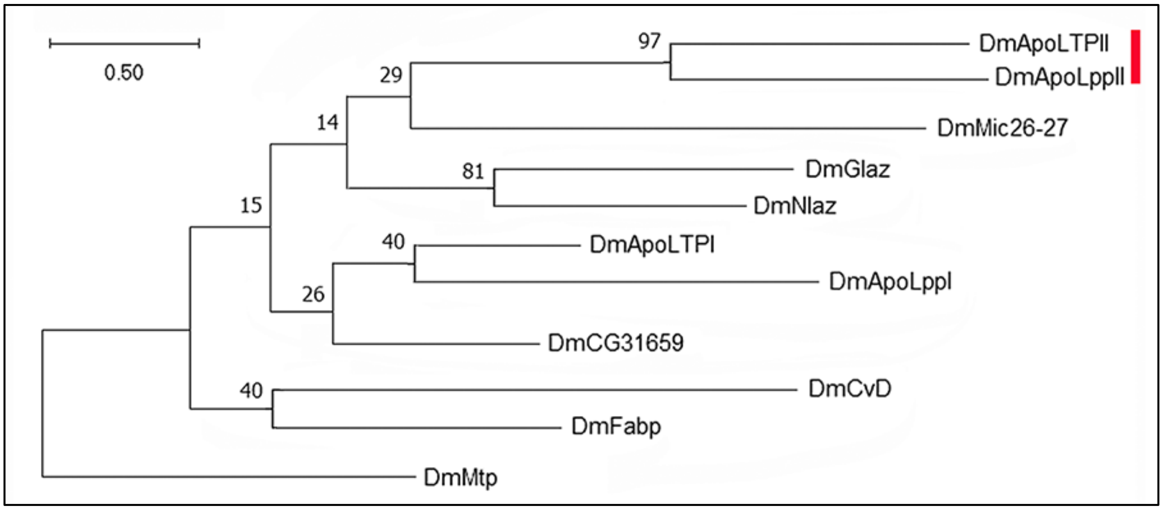

Fig. S1. Tree of 11 fly sequences with isoforms of ApoLpp and ApoLTP separated plus an outgroup. Maximum Likelihood tree with the highest log likelihood is shown as in **Fig. 1**. There are 194 informative positions in the MUSCLE alignment. Red bar indicates the strong grouping of ApoLppII with ApoLTPII. At a distance ApoLppI and ApoLTPI cluster together. The distinct placement of the ApoLpp/ApoLTP isoforms on the tree suggests divergent selection on the N-terminal versus the C-terminal isoforms in ApoLpp prior to the duplication creating ApoLTP.

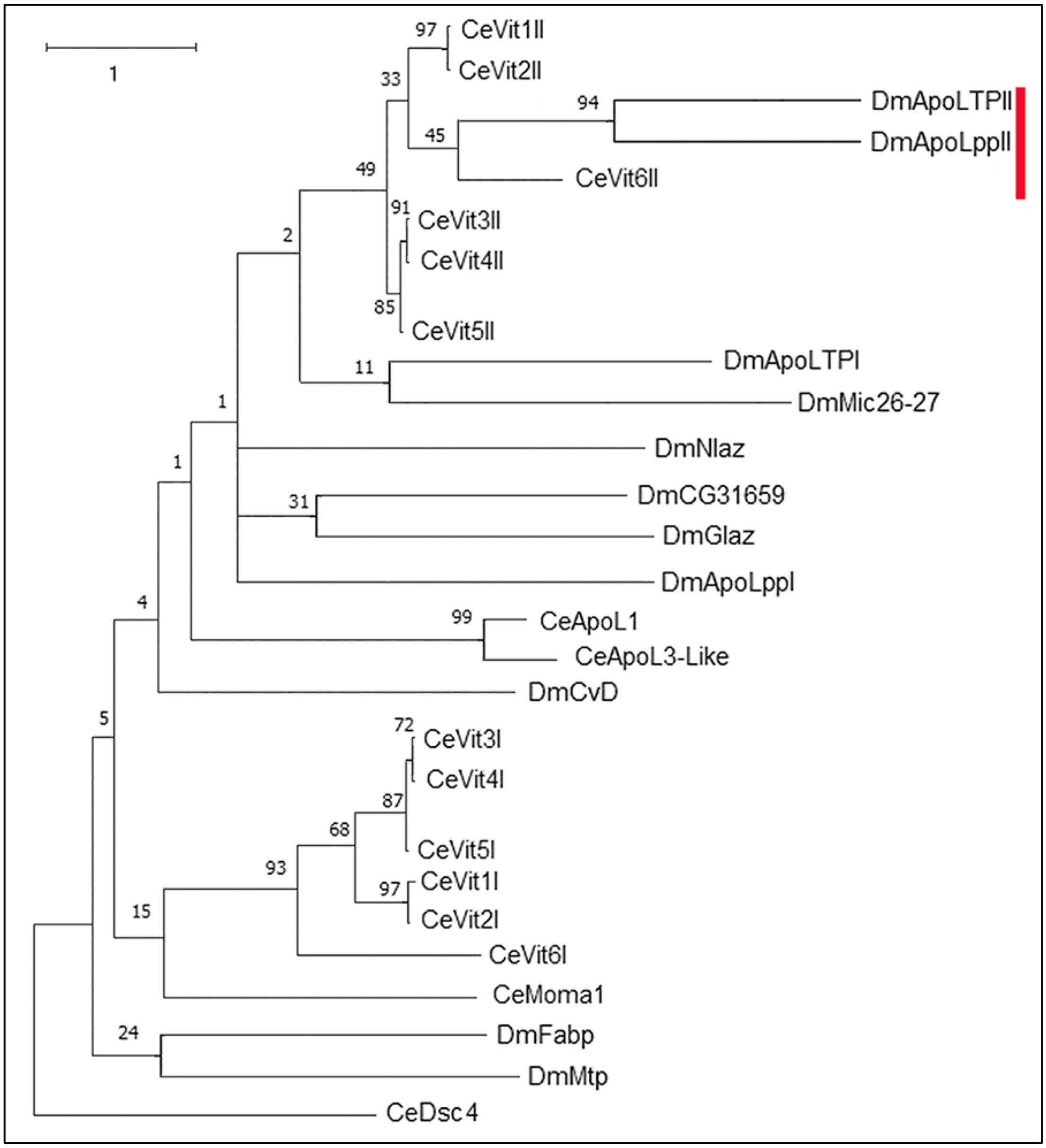

Fig. S2. Fly ApoLppII and nematode Vit-6II are conserved. Maximum Likelihood tree with the highest log likelihood is shown as in **Fig. 1**. There are 11 fly (Dm) and 16 nematode (Ce) sequences in the tree with the nematode outgroup. The two isoforms of ApoLpp, ApoLTP and Vit-1 through Vit-6 are shown separately. There are 279 informative positions in the MUSCLE alignment. Red bar indicates the cluster of Vit-6II with ApoLppII and ApoLTPII (all N-terminal domains). This cluster plus the cluster of the N-terminal domains ApoLppII and ApoLTPII with human ApoB48 in **Fig. 2** reveals that ApoB and Vit6 likely descended from a common ancestor.

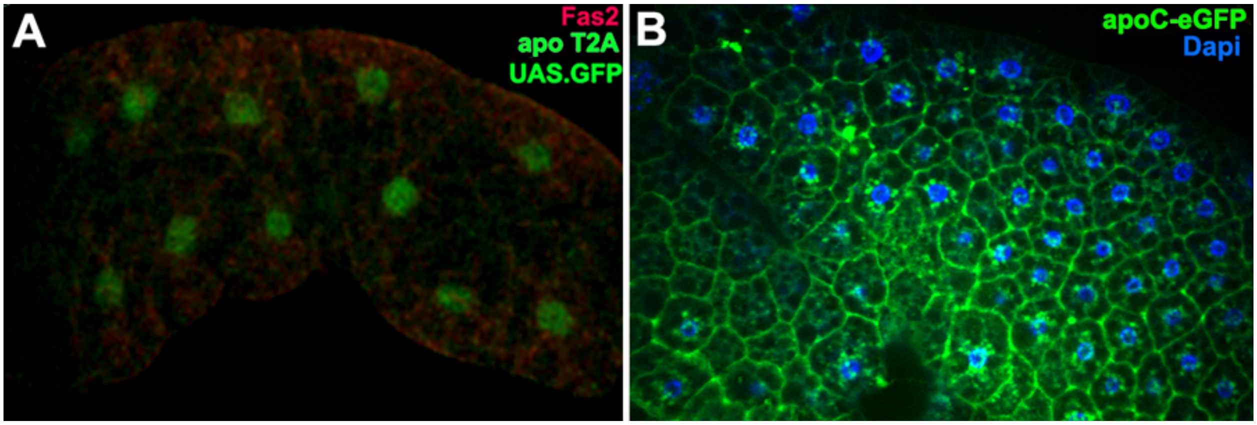

Fig. S3. ***apolpp*** **T2A.GAL4 and ApoLppI C-eGFP are visible in the larval fat body.** A,B) Confocal stacks of larval female fat bodies (n=2 each) with green displaying either UAS.nls-GFP driven by *apolpp* T2A.GAL4 (green) with FasII (red) or ApoLppI C-eGFP (green) with DAPI (blue). As FasII is a neuronal membrane protein, no expression is expected but a low level of background staining is visible outlining the tissue. **In both views,** GFP is visible in many if not all cells.

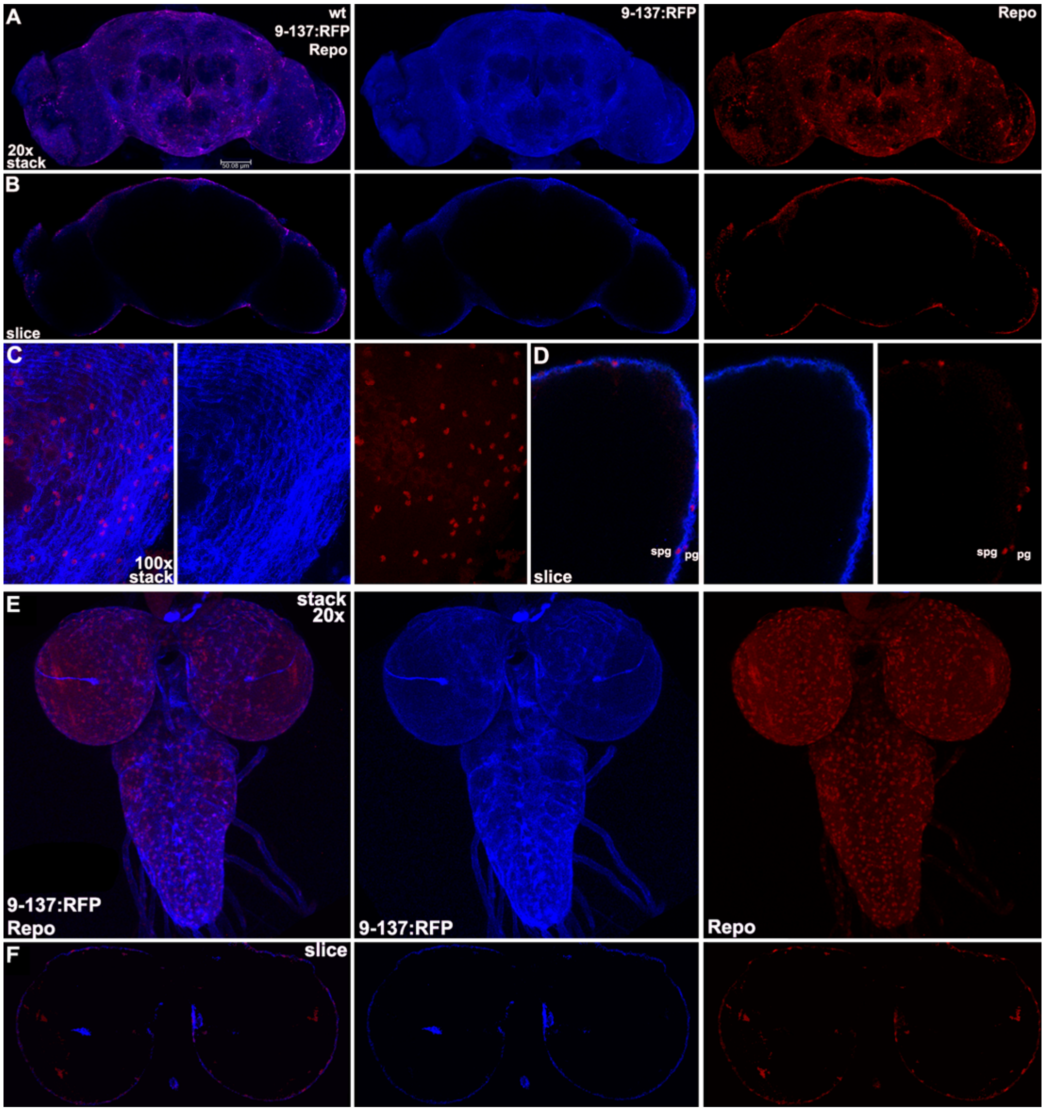

Fig. S4. 9-137.GAL4 driving UAS.CD8-RFP is BBB specific in adult and larval brains. A-D) Ten day old mated female adult brain (n=10) displays 9-137:RFP (blue) with Repo (red). A) Low magnification stack shows 9-137:RFP is uniformly visible. B) Single slice shows that 9-137:RFP is restricted to the BBB. C) High magnification stack of one brain lobe reveals 9-137:RFP is uniformly visible. D) Single slice demonstrates that 9-137:RFP is restricted to BBB glia (apical row of pg and medial row of spg nuclei are indicated). E,F) Female third instar larval brain (n=7) as above. E) Low magnification stack shows 9-137:RFP is uniformly visible. The transformation marker 3xP3-RFP seen in Bolwig's organ in the center of the optic lobes is unrelated to 9:137:RFP in the BBB and does not interfere with morphotrap experiments. F) Single slice of the brain lobes at low magnification indicates that 9-137:RFP is restricted to BBB glia.

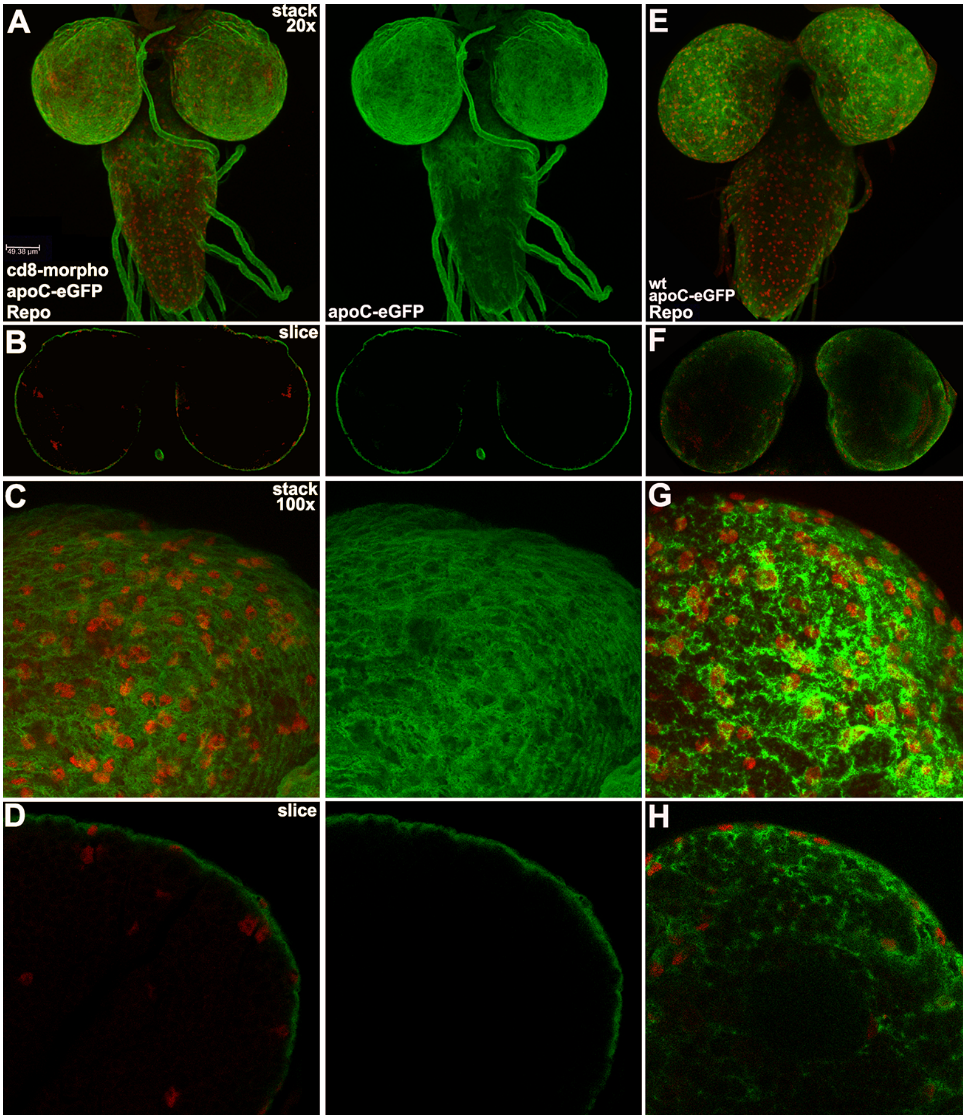

Fig. S5. BBB morphotrap in a third instar larva shows that ApoLppI C-eGFP in the brain is exogenous A-D) Female brain (n=6) with ApoLppI C-eGFP and CD8-morpho as shown in Fig. 8. A) ApoLppI C-eGFP is uniformly distributed on the brain surface. B) Single slice shows that ApoLppI C-eGFP is restricted to the brain surface. C) High magnification stack indicates ApoLppI C-eGFP is uniformly distributed on the brain surface. D) Single slice demonstrates that ApoLppI C-eGFP is restricted to the brain surface. E-H) Female brain (n=4) with ApoLppI C-eGFP but without CD8-morpho. E) ApoLppI C-eGFP is uniformly distributed on the brain surface. F) Single slice shows ApoLppI C-eGFP is present on the surface and inside the brain. G) High magnification stack of one optic lobe reveals ApoLppI C-eGFP is uniformly distributed on the brain surface. H) Single slice of the same lobe reveals ApoLppI eGFP is present on the surface and inside the brain.

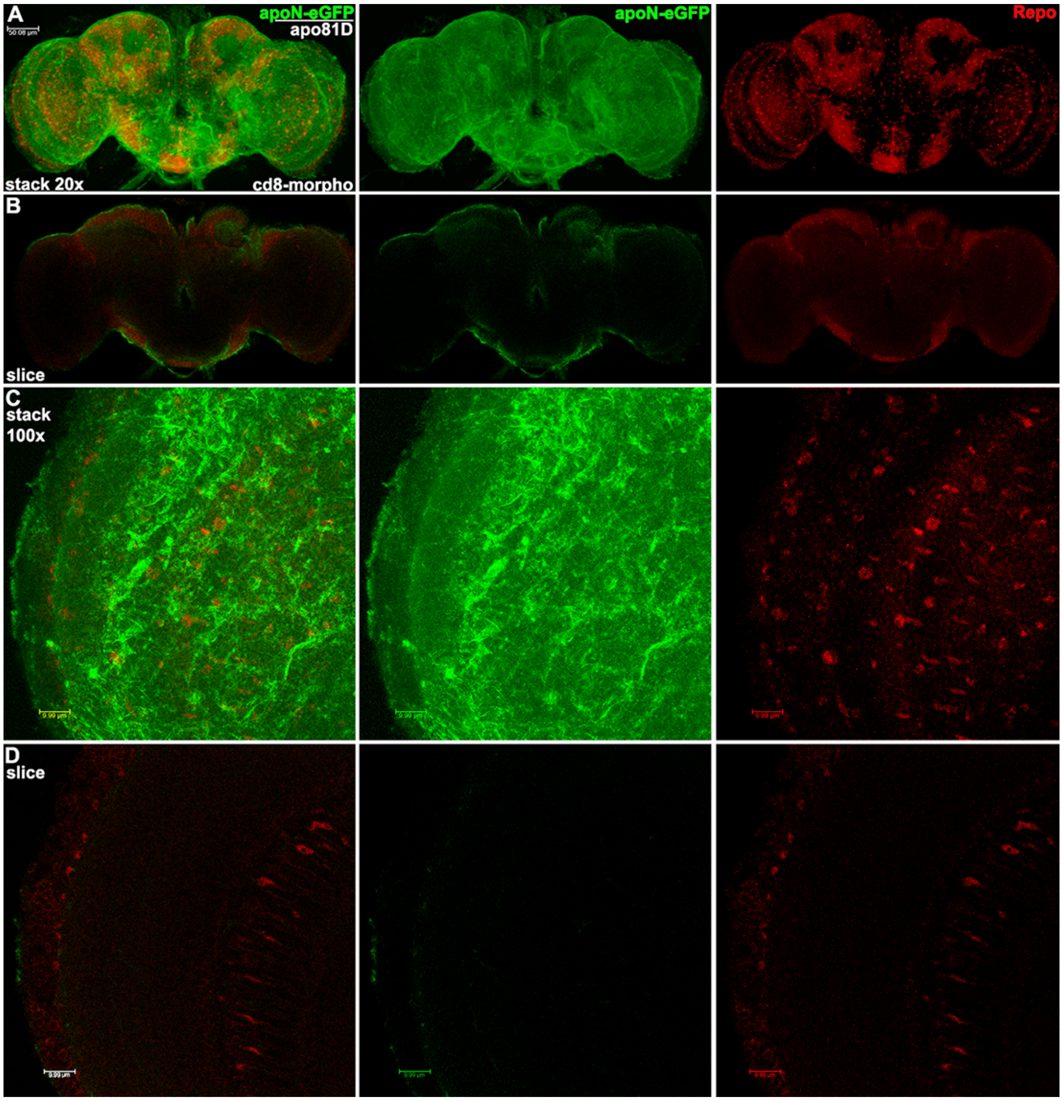

Fig. S6. BBB morphotrap in a one day old unmated adult female shows that ApoLppII N-eGFP in the brain is exogenous. One day old adult virgin female brain (n=3) as shown in Fig. 8 displaying ApoLppII N-eGFP (green) and Repo (red). A) Low magnification stack as two color and individual channels. ApoLppII N-eGFP is uniformly distributed. B) Single slice of the same brain indicates that ApoLppII N-eGFP is restricted to the surface. C) High magnification stack of one brain lobe shows that ApoLppII N-eGFP is uniformly distributed. D) Single slice of the same lobe demonstrates ApoLppII N-eGFP is restricted to the surface.

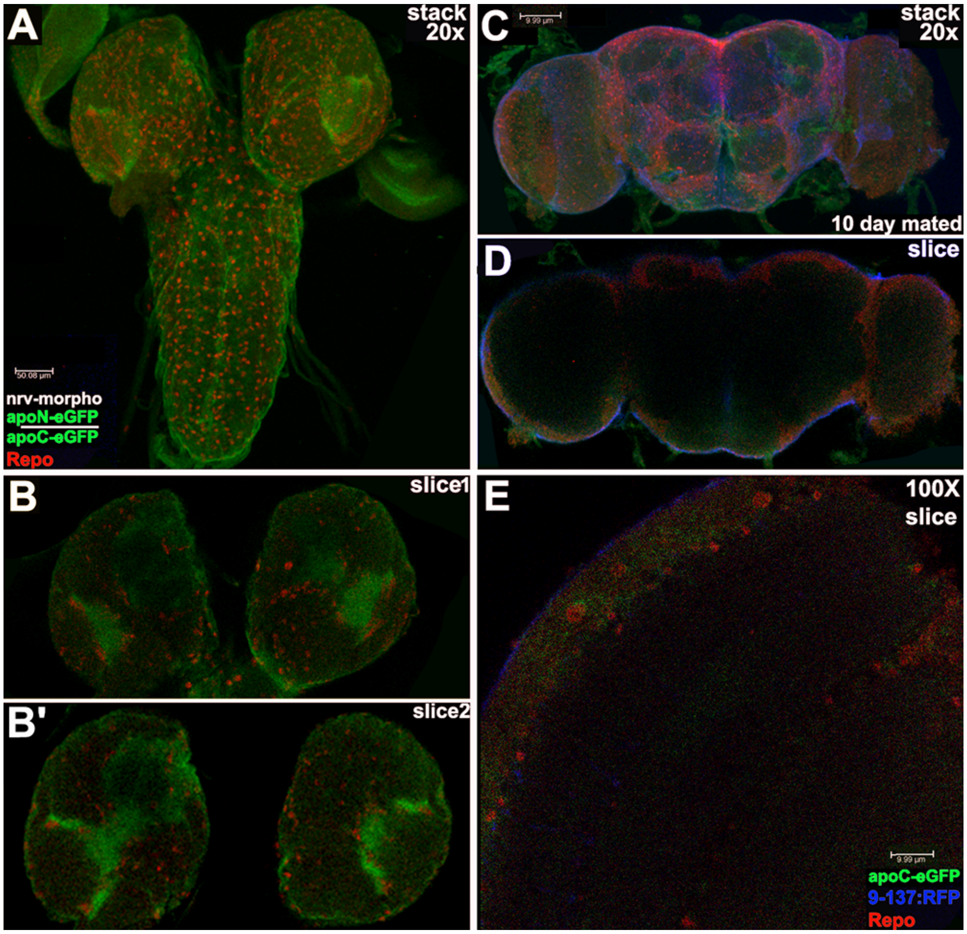

Fig. S7. ApoLpp eGFP is not trapped at the BBB by Nrv-morpho. A,B) Single female third instar larval brain (n=3) expressing Nrv-morpho with 9-137.GAL4 in the BBB that displays ApoLpp eGFP (green) and Repo (red). A) Low magnification confocal stack of the brain and cord (with an eye and wing disk) in two colors demonstrating that Nrv-morpho is not effective at restricting ApoLpp eGFP to the surface. B, B') Low magnification slices of the same brain show internal accumulation of eGFP near glia in the visual system. C-E) Single ten day old adult mated female brain expressing Nrv-morpho with 9-137.GAL4 displaying ApoLppI C-eGFP (green), 9-137:RFP (blue) and Repo (red). D,E) Low and high magnification of the same brain single slices show the wild type accumulation pattern of ApoLppI C-eGFP in the cortex and medulla.

Fig. S8. Alignment of 25 human sequences with ApoB48 and ApoB52 separated plus an outgroup.

```
HsApoA1  0    ------------------------------------------------------------------------------------------
HsApoA2  0    ------------------------------------------------------------------------------------------
HsApoA4  1    ---------------------------------------------------------------------------------------MFL
HsApoA5  0    ------------------------------------------------------------------------------------------
HsApoB48 1    ------------------MDPPRPALLALLALPALLLLLLAGARAEEEMLENVSLVCPKDATRFKHLRKYTYNYEAESSSGVPGTADSRS
HsApoB52 1    FDQYIKDSYDLHDLKIAIANIIDEIIEKLKSLDEHYHIRVNLVKTIHDLHLFIENIDFNKSGSSTASWIQNVDTKYQIRIQIEKLQQLK
HsApoC1  0    ------------------------------------------------------------------------------------------
HsApoC2  0    ------------------------------------------------------------------------------------------
HsApoC3  0    ------------------------------------------------------------------------------------------
HsApoC4  0    ------------------------------------------------------------------------------------------
HsApoD   0    ------------------------------------------------------------------------------------------
HsApoE2  0    ------------------------------------------------------------------------------------------
HsApoF   0    ------------------------------------------------------------------------------------------
HsApoH   0    ------------------------------------------------------------------------------------------
HsApoJ   1    ----------------------------------------------------------------------------------------MM
HsApoL1  1    -------------------------------------------------------------------------MEGAALLRVSVLCIWMS
HsApoL2  0    ------------------------------------------------------------------------------------------
HsApoL3  0    ------------------------------------------------------------------------------------------
HsApoL4  0    ------------------------------------------------------------------------------------------
HsApoL5  0    ------------------------------------------------------------------------------------------
HsApoL6  0    ------------------------------------------------------------------------------------------
HsApoM   0    ------------------------------------------------------------------------------------------
HsApoO   0    ------------------------------------------------------------------------------------------
HsApoOL  0    ------------------------------------------------------------------------------------------
HsMTTP   1    ----------------------------------------------------------MILLAVLFLCFISSYSASVKGHTTGLSLNNDR

HsApoA1  1    -------------------------------------------------MSKDLE-----------------------------------
HsApoA2  0    ------------------------------------------------------------------------------------------
HsApoA4  4    KAVVLTLALVAVAGARAEVSADQVATVMWDYFSQLSNNAKEAVEHLQKSELTQQLNALFQDKLGEVNTYAGDLQKKLVPFATELHERLAK
HsApoA5  1    -MASMAAVLTWALALLSAFSATQARKGFWDYFSQTSGDKGRVEQIHQQKMAREPATL---------KDSLEQDLNNMNKFLEKLRPLSGS
HsApoB48 73   ATRINCKVELEVPQLCSFILKTSQCTLKEVYGFNPEGKALLKKTKNSEEFAAMSRYELKLAIPEGKQVFLYPEKDEPTYILNIKRGIIS
HsApoB52 91   RHIQNIDIQHLAGKLKQHIEAIDVRVLLDQLGTTISFERINDILEHVKHFVINLIGDFEVAEKINAFRAKVHELIERYEVDQQIQVLMDK
HsApoC1  0    ------------------------------------------------------------------------------------------
HsApoC2  0    ------------------------------------------------------------------------------------------
HsApoC3  1    -------------------------------------------MQPRVLLVVALLAL---------------------------------
HsApoC4  0    ------------------------------------------------------------------------------------------
HsApoD   1    --------MVMLLLLLSALAGLFGAAEGQAFHLGKCPNPPVQENFDVNKY---LGRWYEIEKIPTTFENGRCIQ--------ANYSLMEN
HsApoE2  1    ------MKVLWAALLVTFLAGCQAKVEQAVETEPEPELRQQTEWQSGQRWELALGRFWDYLRWVQTLSEQVQEELLSSQVTQELRALMDE
HsApoF   1    -MTGLCGYSAPDMRGLRLIMIPVELLLCYLLLHPVDATSYGKQTNVLMHFPLSLESQ---------------TPSSDPLSCQFLHPKSLP
HsApoH   1    -----------------MISPVLILFSSFLCHVAIAGRTCPKPDDLPFSTVVPLKTF--------------------YEPGEEITYSCKP
HsApoJ   3    KTLLLFVGLLLTWESGQVLGDQTVSDNELQEMSNQGSKYVNKEIQNAVNGVKQIKTLIEKTNEERKTLLSNLEEAKKKKEDALNETRESE
HsApoL1  18   ALFLGVGVRAEEAGARVQQNVPSGTDTGDPQSKPLGDWAAGTMDPESSIFIEDAIKY-------------------------FKEKVSTQ
HsApoL2  1    ------------------------------------------MNPESSIFIEDYLKY-------------------------FQDQVSRE
HsApoL3  1    ------------------------------------------MDSEKKRFTEEATKY-------------------------FRERVSPV
HsApoL4  1    ------------------MGSWVQLITSVGVQQNHPGWTVAGQFQEKKRFTEEVIEY-------------------------FQKKVSPV
HsApoL5  1    -MPCGKQGNLQVPGSKVLPGLGEGCKEMWLRKVIYGGEVWGKSPEPEFPSLVNLCQSWKINN----------------------------
HsApoL6  1    ---------------------------------------MDNQAERESEAGVGLQRD---------------------------------
HsApoM   1    --------------------------------------------MFHQIWAALLYFY---------------------------------
HsApoO   1    --------------MFKVIQRSVGPASLSLLTFKVYAAPKKDSPPKNSVKVDELSLY---------------------------------
HsApoOL  1    -------------MAAIRMGKLTTMPAGLIYASVSVHAAKQEESKKQLVKPEQLPIY---------------------------------
HsMTTP   33   LYKLTYSTEVLLDRGKGKLQDSVGYRISSNVDVALLWRNPDGDDDQLIQITMKDVNVENVNQQRGEKSIFKGKSPSKIMGKENLEALQRP

HsApoA1  6    ------------------------------------------------------------------------------------------
HsApoA2  0    ------------------------------------------------------------------------------------------
HsApoA4  94   DSEKLKEEIGKELE----------------------------------------------------------------------------
HsApoA5  81   EAPRLPQDPVGMRRQLQEELE---------------------------------------------------------------------
HsApoB48 163  ALLVPPETEEAKQVLFLDTVYGNCSTHFTVKTRKGNVATEISTERDLGQCDRFKPIRTGISPLALIKGMTRPLSTLISSSQSCQYTLDAK
HsApoB52 181  LVELAHQYKLKETIQKLSNVLQQVKIKDYFEKLVGFIDDAVKKLNELSFKTFIEDVNKFLDMLIKKLKSFDYHQFVDETNDKIREVTQRL
HsApoC1  0    ------------------------------------------------------------------------------------------
HsApoC2  0    ------------------------------------------------------------------------------------------
HsApoC3  14   ------------------------------------------------------------------------------------------
HsApoC4  0    ------------------------------------------------------------------------------------------
HsApoD   71   GKIKVL------------------------------------------------------------------------------------
HsApoE2  85   TMKELKAYKSELEEQLTPVAE---------------------------------------------------------------------
HsApoF   75   GF----------------------------------------------------------------------------------------
HsApoH   54   GY----------------------------------------------------------------------------------------
HsApoJ   93   TKLKELPGVCNETMMALWEECKPCLKQ---------------------------------------------------------------
HsApoL1  83   NLLLLLTDNEAWNGFVAAAELPR-------------------------------------------------------------------
HsApoL2  24   NLLQLLTDDEAWNGFVAAAELPR-------------------------------------------------------------------
HsApoL3  24   HLQILLTNNEAWKRFVTAAELPR-------------------------------------------------------------------
HsApoL4  48   HLKILLTSDEAWKRFVRVAELPR-------------------------------------------------------------------
HsApoL5  61   ------------------------------------------------------------------------------------------
HsApoL6  18   ------------------------------------------------------------------------------------------
HsApoM   13   ------------------------------------------------------------------------------------------
HsApoO   43   ------------------------------------------------------------------------------------------
HsApoOL  44   ------------------------------------------------------------------------------------------
HsMTTP   123  TLLHLIHGKVKEFYSYQNEAVAIENIKRGLASLFQTQLSSGTTNEVDISGNCKVTYQAHQDKVIKIKALDSCKIARSGFTTPNQVLGVSS
```

```
HsApoA1  6    ------------------------------------------------------------------------------------------
HsApoA2  0    ------------------------------------------------------------------------------------------
HsApoA4  107  ------------------------------------------------------------------------------------------
HsApoA5  101  ------------------------------------------------------------------------------------------
HsApoB48 253  RKHVAEAICKEQHLFLPFSYKNKYGMVAQVTQTLKLEDTPKINSRFFGEGTKKMGLAFESTKSTSPPKQAEAVLKTLQELKKLTISEQNI
HsApoB52 271  NGEIQALELPQKAEALKLFLEETKATVAVYLESLQDTKITLIINWLQEALSSASLAHMKAKFRETLEDTRDRMYQMDIQQELQRYLSLVG
HsApoC1  0    ------------------------------------------------------------------------------------------
HsApoC2  0    ------------------------------------------------------------------------------------------
HsApoC3  14   ------------------------------------------------------------------------------------------
HsApoC4  0    ------------------------------------------------------------------------------------------
HsApoD   77   ------------------------------------------------------------------------------------------
HsApoE2  105  ------------------------------------------------------------------------------------------
HsApoF   76   ------------------------------------------------------------------------------------------
HsApoH   55   ------------------------------------------------------------------------------------------
HsApoJ   119  ------------------------------------------------------------------------------------------
HsApoL1  105  ------------------------------------------------------------------------------------------
HsApoL2  46   ------------------------------------------------------------------------------------------
HsApoL3  46   ------------------------------------------------------------------------------------------
HsApoL4  70   ------------------------------------------------------------------------------------------
HsApoL5  61   ------------------------------------------------------------------------------------------
HsApoL6  18   ------------------------------------------------------------------------------------------
HsApoM   13   ------------------------------------------------------------------------------------------
HsApoO   43   ------------------------------------------------------------------------------------------
HsApoOL  44   ------------------------------------------------------------------------------------------
HsMTTP   213  KATSVTTYKIEDSFVIAVLAEETHNFGLNFLQTIKGKIVSKQKLELKTTEAGPRLMSGKQAAAIIKAVDSKYTAIPIVG-----------

HsApoA1  6    ------------------------------------------------------------------------EVKAKVQPYLDDFQKKWQ
HsApoA2  0    ------------------------------------------------------------------------------------------
HsApoA4  107  ------------------------------------------------------------------------ELRARLLPHANEVSQKIG
HsApoA5  101  ------------------------------------------------------------------------EVKARLQPYMAEAHELVG
HsApoB48 343  QRANLFNKLVTELRGLSDEAVTSLLPQLIEVSSPITLQALVQCGQPQCSTHILQWLKRVHANPLLI------DVVTYLVALIPEPSAQQL
HsApoB52 361  QVYSTLVTYISDWWTLAAKNLTDFAEQYSIQDWAKRMKALVEQGFTVPEIKTILGTMPAFEVSLQALQKATFQTPDFIVPLTDLRIPSVQ
HsApoC1  0    ------------------------------------------------------------------------------------------
HsApoC2  0    ------------------------------------------------------------------------------------------
HsApoC3  14   ------------------------------------------------------------------------------------------
HsApoC4  0    ------------------------------------------------------------------------------------------
HsApoD   77   ------------------------------------------------------------------------NQELRADGTVNQIEGEAT
HsApoE2  105  ------------------------------------------------------------------------ETRARLSKELQAAQARLG
HsApoF   76   ------------------------------------------------------------------------SHMAPLPKFLVSLALRNA
HsApoH   55   ------------------------------------------------------------------------VSRGGMRKFICPLTGLWP
HsApoJ   119  ------------------------------------------------------------------------TCMKFYARVCRSGSGLVG
HsApoL1  105  ------------------------------------------------------------------------NEADELRKALDNLARQMI
HsApoL2  46   ------------------------------------------------------------------------DEADELRKALNKLASHMV
HsApoL3  46   ------------------------------------------------------------------------DEADALYEALKKLRTYAA
HsApoL4  70   ------------------------------------------------------------------------EEADALYEALKNLTPYVA
HsApoL5  61   ------------------------------------------------------------------------LMSTVHSDEAGMLSYFLF
HsApoL6  18   ------------------------------------------------------------------------EDDAPLCEDVELQDGDLS
HsApoM   13   ------------------------------------------------------------------------GIILNSIYQCPEHSQLTT
HsApoO   43   ------------------------------------------------------------------------SVPEGQSKYVEEARSQLE
HsApoOL  44   ------------------------------------------------------------------------TAPPLQSKYVEEQPGHLQ
HsMTTP   291  ------------------------------------------------------------------------QVFQSHCKGCPSLSELWR

HsApoA1  25   EEMELYRQKVEPLRAEL-----------------QEGARQKLHELQEKLSPLGEE-----------------------------------
HsApoA2  1    ---------------------------------------MKLLAATVLLLTICS------------------------------------
HsApoA4  126  DNLRELQQRLEPYADQLRTQVSTQ----------AEQLRRQLTPYAQRMERVLRE-----------------------------------
HsApoA5  120  WNLEGLRQQLKPYTMDL-----------------MEQVALRVQELQEQLRVVGED-----------------------------------
HsApoB48 427  REIFNMARDQRSRATLYALSHAVNNYHKTN-PTGTQELLDIANYLMEQIQDDCTGDEDYTYLILRVIGNMGQTMEQLTPELKSSILKCVQ
HsApoB52 451  INFKDLKNIKIPSRFSTPEFTILNTFHIPSFTIDFVEMKVKIIRTIDQMLNSELQWPVPDIYLRDLKVEDIPLARITLPDFRLPEIAIPE
HsApoC1  1    -----------------------------------MRLFLSLPVLVVVLSIVLEG-----------------------------------
HsApoC2  1    ------------------------------------MGTRLLPALFLVLLVLGFE-----------------------------------
HsApoC3  14   ----------------------------------LASARASEAEDASLLSFMQGY-----------------------------------
HsApoC4  1    --------------------------------------MSLLRNRLQALPALCLC-----------------------------------
HsApoD   96   PVNLTEPAKLEVKFSWFM----------------PSAPYWILATDYENYALVYSC-----------------------------------
HsApoE2  124  ADMEDVCGRLVQYRGEVQAMLGQS----------TEELRVRLASHLRKLRKRLLR-----------------------------------
HsApoF   95   LEEAGCQADVWALQLQLY----------------RQGGVNATQVLIQHLRGLQKG-----------------------------------
HsApoH   74   INTLKCTPRVCPFAGILE----------------NGAVRYTTFEYPNTISFSCNT-----------------------------------
HsApoJ   138  RQLEEFLNQSSPFYFWMNGDRIDSLLENDRQ---QTHMLDVMQDHFSRASSIIDE-----------------------------------
HsApoL1  124  MKDKNWHDKGQQYRNWFLKE--------------FPRLKSELEDNIRRLRALADG-----------------------------------
HsApoL2  65   MKDKNRHDKDQQHRQWFLKE--------------FPRLKRELEDHIRKLRALAEE-----------------------------------
HsApoL3  65   IEDEYVQQKDEQFREWFLKE--------------FPQVKRKIQESIEKLRALANG-----------------------------------
HsApoL4  89   IEDKDMQQKEQQFREWFLKE--------------FPQIRWKIQESIERLRVIANE-----------------------------------
HsApoL5  80   EELMRCDKDSMPDGNLSEEEKLFLSY--------FPLHKFELEQNIKELNTLADQ-----------------------------------
HsApoL6  37   PEEKIFLRE-------------------------FPRLKEDLKGNIDKLRALADD-----------------------------------
HsApoM   32   LGVDGKEFPEVHLGQWY-----------------FIAGAAPTKEELATFDPVDNI-----------------------------------
HsApoO   62   ESISQLRHYCEPYTTWCQETYSQT----------KPKMQSLVQWGLDSYDYLQNA-----------------------------------
HsApoOL  63   MGFASIRTATGCYIGWCKGVYVFV----------KNGIMDTVQFGKDAYVYLKNP-----------------------------------
HsMTTP   310  STRKYLQPDNLSKAEAVRNFLAF-----------IQHLRTAKKEEILQILKMENK-----------------------------------
```

```
HsApoA1  62   ------------------------------------------------------------------------------------------
HsApoA2  15   ------------------------------------------------------------------------------------------
HsApoA4  170  ---------------------------------------------------------------------NADSLQASLRPHADELKAKID
HsApoA5  157  ------------------------------------------------------------------------------TKAQLLGGVDEA
HsApoB48 516  STKPSLMIQKAAIQALRKMEPKDKDQEVLLQTFLDDASPGDKRLAAYLMLMRSPSQADINKIVQILPWEQNEQVKNFVASHIANILNSEE
HsApoB52 541  FIIPTLNLNDFQVPDLHIPEFQLPHISHTIEVPTFGKLYSILKIQSPLFTLDANADIGNGTTSANEAGIAASITAKGESKLEVLNFDFQA
HsApoC1  20   ------------------------------------------------------------------------------------------
HsApoC2  19   ------------------------------------------------------------------------------------------
HsApoC3  35   ------------------------------------------------------------------------------------------
HsApoC4  17   ------------------------------------------------------------------------------------------
HsApoD   135  ------------------------------------------------------------------------------------------
HsApoE2  168  ------------------------------------------------------------------------------------------
HsApoF   133  --------------------------------------------------------------------------RSTERNVSVEALASA-
HsApoH   112  ---------------------------------------------------------------------GFYLNGADSAKCTEEGKWSPE
HsApoJ   189  ---------------------------------------------------------LFQDRFFTREPQDTYHYLPFSLPHRRPHFFFPK
HsApoL1  164  -------------------------------------------------------------------------VQKVHKGTTIANVVSGS
HsApoL2  105  -------------------------------------------------------------------------VEQVHRGTTIANVVSNS
HsApoL3  105  -------------------------------------------------------------------------IEEVHRGCTISNVVSSS
HsApoL4  129  -------------------------------------------------------------------------IEKVHRGCVIANVVSGS
HsApoL5  126  -------------------------------------------------------------------------VDTTHELLTKTSLVASS
HsApoL6  66   -------------------------------------------------------------------------IDKTHKKFTKANMVATS
HsApoM   69   ------------------------------------------------------------------------------------------
HsApoO   106  ------------------------------------------------------------------------------------------
HsApoOL  107  ------------------------------------------------------------------------------------------
HsMTTP   353  ----------------------------------------------------------EVLPQLVDAVTSAQTSDSLEAILDFLDFKSDS

HsApoA1  62   ------------------------------------------------------------------------------------------
HsApoA2  15   ------------------------------------------------------------------------------------------
HsApoA4  192  QNVEE-------------------------------------------------------------------------------------
HsApoA5  170  WALLQG------------------------------------------------------------------------------------
HsApoB48 606  LDIQDLKKLVKEALKESQLPTVMDFRKFSRNYQLYKSVSLPSLD----------------------------------------------
HsApoB52 631  NAQLSNPKINPLALKESVKFSSKYLRTEHGSEMLFFGNAIEGKSNTVASLHTEKNTLELSNGVIVKINNQLTLDSNTKYFHKLNIPKLDF
HsApoC1  20   ------------------------------------------------------------------------------------------
HsApoC2  19   ------------------------------------------------------------------------------------------
HsApoC3  35   ------------------------------------------------------------------------------------------
HsApoC4  17   ------------------------------------------------------------------------------------------
HsApoD   135  ------------------------------------------------------------------------------------------
HsApoE2  168  ------------------------------------------------------------------------------------------
HsApoF   148  ------------------------------------------------------------------------------------------
HsApoH   134  LPVCAP------------------------------------------------------------------------------------
HsApoJ   223  SRIVR-------------------------------------------------------------------------------------
HsApoL1  182  LSISSGILTL--------------------------------------------------------------------------------
HsApoL2  123  VGTTSGILTL--------------------------------------------------------------------------------
HsApoL3  123  TGAASGIMSL--------------------------------------------------------------------------------
HsApoL4  147  TGILSV------------------------------------------------------------------------------------
HsApoL5  144  SGAVSGVMNI--------------------------------------------------------------------------------
HsApoL6  84   TAVISGVMSL--------------------------------------------------------------------------------
HsApoM   69   ------------------------------------------------------------------------------------------
HsApoO   106  ------------------------------------------------------------------------------------------
HsApoOL  107  ------------------------------------------------------------------------------------------
HsMTTP   386  SIILQERFL---------------------------------------------------------------------------------

HsApoA1  62   ------------------------------------------------------------------------------------------
HsApoA2  15   ------------------------------------------------------------------------------------------
HsApoA4  196  ----------------------------------------------LKGRLTPYADEFKVKIDQT-------------------------
HsApoA5  175  -----------------------------------------------LQSRVVHHTGRFKELFHPY------------------------
HsApoB48 649  -------------------PASAKIEGNL-----------------IFDPNNYLPKESMLKTTLTAFGFASADLIEIGLEGKGFEPTLEA
HsApoB52 721  SSQADLRNEIKTLLKAGHIAWTSSGKGSWKWACPRFSDEGTHESQISFTIEGPLTSFGLSNKINSKHLRVNQNLVYESGSLNFSKLEIQS
HsApoC1  20   ------------------------------------------------------------------------------------------
HsApoC2  19   ------------------------------------------------------------------------------------------
HsApoC3  35   ------------------------------------------------------------------------------------------
HsApoC4  17   ----------------------------------------------------------VLVLACI-------------------------
HsApoD   135  ----------------------------------------------TCIIQLFHVDFAWILARNP-------------------------
HsApoE2  168  -----------------------------------------------------DADDLQKRLAVY-------------------------
HsApoF   148  ----------------------------------------------LQLLAREQQSTGRVGRSLP-------------------------
HsApoH   139  ----------------------------------------------IICPPPSIPTFATLRVYKP-------------------SAGNNS
HsApoJ   227  ----------------------------------------------SLMPFSPYEPLNFHAMFQP-------------------------
HsApoL1  191  ----------------------------------------------VGMGLAPFTEGGSLVLLEP-------------------GMELGI
HsApoL2  132  ----------------------------------------------LGLGLAPFTEGISFVLLDT-------------------GMGLGA
HsApoL3  132  ----------------------------------------------AGLVLAPFTAGTSLALTAA-------------------GVGLGA
HsApoL4  152  ----------------------------------------------IGVMLAPFTAGLSLSITAA-------------------GVGLGI
HsApoL5  153  ----------------------------------------------LGLALAPVTAGGSLMLSAT-------------------GTGLGA
HsApoL6  93   ----------------------------------------------LGLALAPATGGGSLLLSTA-------------------GQGLAT
HsApoM   69   ---------------------------------------------VFNMAAGSAPMQLHLRATIR-------------------------
HsApoO   106  -------------------------------------------------PPGFFPRLGVIGFAG--------------------------
HsApoOL  107  -------------------------------------------------PRDFLPKMGVITVSGL-------------------------
HsMTTP   394  ----------------------------------------------YACGFASHPNEELLRALISKFKGSIGSSDIRETVMIITGTLVRK
```

```
HsApoA1  62   ------------------------------------------------------------------------------------------
HsApoA2  15   ------------------------------------------------------------------------------------------
HsApoA4  215  ---------------------------------------VEELRRSLAPY----------------------------------------
HsApoA5  194  ------------------------------------------------------------------------------------------
HsApoB48 704  LFGKQGFFPDSVNKALYWVNGQVPDGVSKVLVDHFGYTKDDKHEQDMVNGIMLSVEKLIKDLKSKEVPEARAYLRILGEELGFASLHDLQ
HsApoB52 811  QVDSQHVGHSVLTAKGMALFGEGKAEFTGRHDAHLNGKVIGTLKNSLFFSAQPFEITASTNNEGNLKVRFPLRLTGKIDFLNNYALFLSP
HsApoC1  20   ------------------------------------------------------------------------------------------
HsApoC2  19   ------------------------------------------------------------------------------------------
HsApoC3  35   ------------------------------------------------------------------------------------------
HsApoC4  24   ------------------------------------------------------------------------------------------
HsApoD   154  ----------------------------------------NLPPETVDS-----------------------------------------
HsApoE2  180  ------------------------------------------------------------------------------------------
HsApoF   167  ------------------------------------------------------------------------------------------
HsApoH   165  LY-------------------------------RDTAVFECLPQHAMFG-----------------------------------------
HsApoJ   246  ------------------FLEMIHEAQQAMDIHFSPAFQHPPTEFIREGD----------------------------------------
HsApoL1  217  TA-----------------------------------ALTGITSSTMDYGK---------------------------------------
HsApoL2  158  AA-----------------------------------AVAGITCSVVELVN---------------------------------------
HsApoL3  158  AS-----------------------------------AVTGITTSIVEHSY---------------------------------------
HsApoL4  178  AS-----------------------------------ATAGIASSIVENTY---------------------------------------
HsApoL5  179  AA-----------------------------------AITNIVTNVL-------------------------------------------
HsApoL6  119  AA-----------------------------------GVTSIVSGTLERSK---------------------------------------
HsApoM   89   ------------------------------------------------------------------------------------------
HsApoO   121  ------------------------------------------------------------------------------------------
HsApoOL  123  ------------------------------------------------------------------------------------------
HsMTTP   439  LCQNEGCKLKAVVEAKKLILGGLEKAEKKEDTRMYLLALKNALLPEGIPSLLKYAEAGEGPISHLATTALQRYDLPFITDEVKKTLNRIY

HsApoA1  62   ------------------------------------------------------------------------------------------
HsApoA2  15   ------------------------------------------------------------------------------------------
HsApoA4  226  ------------------------------------------------------------------------------------------
HsApoA5  194  ------------------------------------------------------------------------------------------
HsApoB48 794  LLGKLLLMGARTLQGIPQMIGEVIRKGSKNDFFLHYIFMENAFELPTGAGLQLQISSSGVIAPGAKAGVKLEVANMQAELVAKPSVSVEF
HsApoB52 901  SAQQASWQVSARFNQYKYNQNFSAGNNENIMEAHVGINGEANLDFLNIPLTIPEMRLPYTIITTPPLKDFSLWEKTGLKEFLKTTKQSFD
HsApoC1  20   ------------------------------------------------------------------------------------------
HsApoC2  19   ------------------------------------------------------------------------------------------
HsApoC3  35   ------------------------------------------------------------------------------------------
HsApoC4  24   ------------------------------------------------------------------------------------------
HsApoD   163  ------------------------------------------------------------------------------------------
HsApoE2  180  ------------------------------------------------------------------------------------------
HsApoF   167  ------------------------------------------------------------------------------------------
HsApoH   182  ------------------------------------------------------------------------------------------
HsApoJ   279  ------------------------------------------------------------------------------------------
HsApoL1  232  ------------------------------------------------------------------------------------------
HsApoL2  173  ------------------------------------------------------------------------------------------
HsApoL3  173  ------------------------------------------------------------------------------------------
HsApoL4  193  ------------------------------------------------------------------------------------------
HsApoL5  190  ------------------------------------------------------------------------------------------
HsApoL6  134  ------------------------------------------------------------------------------------------
HsApoM   89   ------------------------------------------------------------------------------------------
HsApoO   121  ------------------------------------------------------------------------------------------
HsApoOL  123  ------------------------------------------------------------------------------------------
HsMTTP   529  HQNRKVHEKTVRTAAAAIILNNNPSYMDVKNILLSIGELPQEMNKYM-------------------------------------------

HsApoA1  62   ----MRDRARAHVDALRTHLAPYSDELR--------------------------------------------------------------
HsApoA2  15   -LEGALVRRQAKEPCVESLVSQ--------------------------------------------------------------------
HsApoA4  226  -AQDTQEKLNHQLEGLTFQMKKNAEELK--------------------------------------------------------------
HsApoA5  194  -AESLVSGIGRHVQELHRSVAPHAPASP--------------------------------------------------------------
HsApoB48 884  VTNMGIIIPDFARSGVQMNTNFFHESGLEAHVALKAGKLKFIIPSPKRPVKLLSGGNTLHLVSTTKTEVIPPLIENRQSWSVCKQVFPGL
HsApoB52 991  LSVKAQYKKNKHRHSITNPLAVLCEFISQSIKSFDRHFEKNRNNALDFVTKSYNETKIKFDKYKAEKSHDELPRTFQIPGYTVPVVNVEV
HsApoC1  20   ------PAPAQGTPDVSSALDKLKEFGN--------------------------------------------------------------
HsApoC2  19   -VQGTQQPQQDEMPSPTFLTQVKESLSS--------------------------------------------------------------
HsApoC3  35   -MKHATKTAKDALSSVQESQ----------------------------------------------------------------------
HsApoC4  24   -GACQPEAQEGTLSPPPKLKMSRWSLVR--------------------------------------------------------------
HsApoD   163  -LKNILTSNNIDVKKMTVTDQVNCPKLSQ-------------------------------------------------------------
HsApoE2  180  -QAGAREGAERGLSAIRERLGPLVEQGR--------------------------------------------------------------
HsApoF   167  -TEDCENEKEQAVHNVVQLLPGVGTF----------------------------------------------------------------
HsApoH   182  -NDTITCTTHGNWTKLPECREVKCPF----------------------------------------------------------------
HsApoJ   279  -DDRTVCREIRHNSTGCLRMKDQCDKCR--------------------------------------------------------------
HsApoL1  232  -KWWTQAQAHDLVIKSLDKLKEVREFLG--------------------------------------------------------------
HsApoL2  173  -KLRARAQARNLDQSGTNVAKVMKEFVG--------------------------------------------------------------
HsApoL3  173  -TSSAEAEASRLTATSIDRLKVFKEVMR--------------------------------------------------------------
HsApoL4  193  -TRSAELTASRLTATSTDQLEALRDILH--------------------------------------------------------------
HsApoL5  190  -ENRSNSAARDKASRLGPLTTSHEAFGGINW-----------------------------------------------------------
HsApoL6  134  -NKEAQARAEDILPTYDQEDREDEEEKA--------------------------------------------------------------
HsApoM   89   -MKDGLCVPRKWIYHLTEGSTDLRTEGR--------------------------------------------------------------
HsApoO   121  ------------------------------------------------------------------------------------------
HsApoOL  123  -AGLVSARKGSKFKKITYPLGLATLGAT--------------------------------------------------------------
HsMTTP   575  -LAIVQDILRFEMPASKIVRRVLKEMVAHNYDRFSRSG----------------------------------------------------
```

```
HsApoA1  86   -------------------------------------------------------------QRLAA------------------------
HsApoA2  36   ------------------------------------------------------------------------------------------
HsApoA4  253  -------------------------------------------------------------ARISASAEE--------------------
HsApoA5  221  -------------------------------------------------------------ARLSRCVQVLSRKLTLKAKALHAR-----
HsApoB48 974  NYCTSGAYSNASSTDSASYYPLTGDTRLELELRPTGEIEQYSVSATYELQREDRALVDTLKFVTQAEGAKQTEATMTFKYNRQSM-----
HsApoB52 1081 SPFTIEMSAFGYVFPKAVSMPSFSILGSDVRVPSYTLILPSLELPVLHVPRNLKLSLPDF-KELCTISHIFIPAMGNITYDFSFKSSVIT
HsApoC1  42   ------------------------------------------------------------------------------------------
HsApoC2  46   ------------------------------------------------------------------------------------------
HsApoC3  54   ------------------------------------------------------------------------------------------
HsApoC4  51   -------------------------------------------------------------GRMKE------------------------
HsApoD   192  -------------------------------------------------------------AKEPCVESLVSQ-----------------
HsApoE2  207  -------------------------------------------------------------VRAATVGSLAGQP----------------
HsApoF   192  -------------------------------------------------------------YNLGTALYYATQN----------------
HsApoH   207  -------------------------------------------------------------PSRPDNGFVNYPAKPTLYYKDK-A-----
HsApoJ   306  -------------------------------------------------------------EILSVDCSTNNPSQAKLRRELDE------
HsApoL1  259  -------------------------------------------------------------ENISNFLSLAGN-----------------
HsApoL2  200  -------------------------------------------------------------GNTPNVLTLVDN-----------------
HsApoL3  200  -------------------------------------------------------------DITPNLLSLLNN-----------------
HsApoL4  220  -------------------------------------------------------------DITPNVLSFALD-----------------
HsApoL5  220  -------------------------------------------------------------SEIEAAGFCVNK-----------------
HsApoL6  161  -------------------------------------------------------------DYVTAAGKIIYNLRN--------------
HsApoM   116  -------------------------------------------------------------PDMKTELFSSSCPGGIM------------
HsApoO   121  ------------------------------------------------------------------------------------------
HsApoOL  150  -------------------------------------------------------------VCYPVQSVIIAKVTAKKVYA---------
HsMTTP   612  -------------------------------------------------------------SSSAYTGYIERSPRSASTYSLDI------

HsApoA1  91   ----------------------------------RLEALKENGG----------------------------------------------
HsApoA2  36   ----------------------------------YFQTVTDYGK---DLMEKVK------------------------------------
HsApoA4  262  ----------------------------------LRQRLAPLAE---DVRGNLR------------------------------------
HsApoA5  245  ----------------------------------IQQNLDQLRE---ELSRAFA------------------------------------
HsApoB48 1058 ----------------------------------TLSSEVQIPDFDVDLGTILRVNDESTEGKTSYRLTLDIQNKKITEVALMGHLSCDT
HsApoB52 1170 LNTNAELFNQSDIVAHLLSSSSSVIDALQYKLEGTTRLTRKRGL---KLATALSLSNKFVEGSHNSTVSLTTKNMEVSVATTTKAQIPIL
HsApoC1  42   -------------------------------------TLEDKAR---ELISRIK------------------------------------
HsApoC2  46   ----------------------------------YWESAKTAAQ---NLYEKTY------------------------------------
HsApoC3  54   ----------------------------------VAQQARGWVT---DGFSSLK------------------------------------
HsApoC4  56   ----------------------------------LLETVVNRTR---DGWQWFW------------------------------------
HsApoD   203  ----------------------------------YFQTVTDYGK---DLMEKVK------------------------------------
HsApoE2  220  ----------------------------------LQERAQAWGE---RLRARME------------------------------------
HsApoF   205  ----------------------------------CLGKARERGR---DGAIDLG------------------------------------
HsApoH   230  ----------------------------------TFGCHDGYSL---DGPEEIE------------------------------------
HsApoJ   329  ----------------------------------SLQVAERLTR---KYNELLK------------------------------------
HsApoL1  271  ----------------------------------TYQLTRGIGK---DIRALRR------------------------------------
HsApoL2  212  ----------------------------------WYQVTQGIGR---NIRAIRR------------------------------------
HsApoL3  212  ----------------------------------YYEATQTIGS---EIRAIRQ------------------------------------
HsApoL4  232  ----------------------------------FDEATKMIAN---DVHTLRR------------------------------------
HsApoL5  232  ----------------------------------CVKAIQGIK----DLHAYQM------------------------------------
HsApoL6  176  ----------------------------------TLKYAKKNVR---AFWKLRA------------------------------------
HsApoM   133  ----------------------------------LNETGQGYQR----------------------------------------------
HsApoO   121  ----------------------------------LIGLLLARGS---KIKKLVY------------------------------------
HsApoOL  170  ----------------------------------TSQQIFGAVK---SLWTKSS------------------------------------
HsMTTP   635  ----------------------------------LYSGSGILRRSNLNIFQYIG------------------------------------

HsApoA1  101  ------------------------------------------------------------------------------------------
HsApoA2  53   ------------------------------------------------------------------------------------------
HsApoA4  279  ------------------------------------------------------------------------------------------
HsApoA5  262  ------------------------------------------------------------------------------------------
HsApoB48 1115 KEERKIKGVISIPRLQAEARSEILAHWSPAKLLLQMDSSATAYGSTVSKRVAWHYDEEKIEFEWNTGTNVDTKKMTSNFPVDLSDYPKSL
HsApoB52 1257 RMNFKQELNGNTKSKPTVSSSMEFKYDFNSSMLYSTAKGAVDHKLSLESLTSYFSIESSTKGDVKGSVLSREYSGTIASEANTYLNSKST
HsApoC1  56   ------------------------------------------------------------------------------------------
HsApoC2  63   ------------------------------------------------------------------------------------------
HsApoC3  71   ------------------------------------------------------------------------------------------
HsApoC4  73   ------------------------------------------------------------------------------------------
HsApoD   219  ------------------------------------------------------------------------------------------
HsApoE2  237  ------------------------------------------------------------------------------------------
HsApoF   222  ------------------------------------------------------------------------------------------
HsApoH   247  ------------------------------------------------------------------------------------------
HsApoJ   346  ------------------------------------------------------------------------------------------
HsApoL1  288  ------------------------------------------------------------------------------------------
HsApoL2  229  ------------------------------------------------------------------------------------------
HsApoL3  229  ------------------------------------------------------------------------------------------
HsApoL4  249  ------------------------------------------------------------------------------------------
HsApoL5  248  ------------------------------------------------------------------------------------------
HsApoL6  193  ------------------------------------------------------------------------------------------
HsApoM   143  ------------------------------------------------------------------------------------------
HsApoO   138  ------------------------------------------------------------------------------------------
HsApoOL  187  ------------------------------------------------------------------------------------------
HsMTTP   655  ------------------------------------------------------------------------------------------
```

```
HsApoA1  101  ------------------------------------------------------------------------------------------
HsApoA2  53   ------------------------------------------------------------------------------------------
HsApoA4  279  ------------------------------------------------------------------------------------------
HsApoA5  262  ------------------------------------------------------------------------------------------
HsApoB48 1205 HMYANRLLDHRVPQTDMTFRHVGSKLIVAMSSWLQKASGSLPYTQTLQDHLNSLKEFNLQNMGLPDFHIPENLFLKSDGRVKYTLNKNSL
HsApoB52 1347 RSSVKLQGTSKIDDIWNLEVKENFAGEATLQRIYSLWEHSTKNHLQLEGLFFTNGEHTSKATLELSPWQMSALVQVHASQPSSFHDFPDL
HsApoC1  56   ------------------------------------------------------------------------------------------
HsApoC2  63   ------------------------------------------------------------------------------------------
HsApoC3  71   ------------------------------------------------------------------------------------------
HsApoC4  73   ------------------------------------------------------------------------------------------
HsApoD   219  ------------------------------------------------------------------------------------------
HsApoE2  237  ------------------------------------------------------------------------------------------
HsApoF   222  ------------------------------------------------------------------------------------------
HsApoH   247  ------------------------------------------------------------------------------------------
HsApoJ   346  ------------------------------------------------------------------------------------------
HsApoL1  288  ------------------------------------------------------------------------------------------
HsApoL2  229  ------------------------------------------------------------------------------------------
HsApoL3  229  ------------------------------------------------------------------------------------------
HsApoL4  249  ------------------------------------------------------------------------------------------
HsApoL5  248  ------------------------------------------------------------------------------------------
HsApoL6  193  ------------------------------------------------------------------------------------------
HsApoM   143  ------------------------------------------------------------------------------------------
HsApoO   138  ------------------------------------------------------------------------------------------
HsApoOL  187  ------------------------------------------------------------------------------------------
HsMTTP   655  ------------------------------------------------------------------------------------------

HsApoA1  101  ----------------------------------------------------------ARLAEYHAKATEHLSTLSEKAKPA--------
HsApoA2  53   --------------------------------------------------------------------SPELQAEAKSY-----------
HsApoA4  279  ------------------------------------------------GNTEGLQKSLAELGGHLDQQVEEFRRRVEPYGENFNKALVQQ
HsApoA5  262  -----------------------------------------------------GTGTEEGAGPDPQMLSEEVRQRLQAFRQDTYLQIAAF
HsApoB48 1295 KIEIPLPFGGKSSRDLKMLETVRTPALHFKSVGFHLPSREFQVPTFTIPKLYQLQVPLLGVLDLSTNVYSNLYNWSASYSGGNTSTDHFS
HsApoB52 1437 GQEVALNANTKNQKIRWKNEVRIHSGSFQSQVELSNDQEKAHLDIAGSLEGHLRFLKNIILPVYDKSLWDFLKLDVTTSIGRRQHLRVST
HsApoC1  56   ---------------------------------------------------QSELSAKMRLEPFPGHGRAGVCFWVEPWQ----------
HsApoC2  63   ---------------------------------------------------------LPAVDEKLRDLYSKSTAAMSTYTGI--------
HsApoC3  71   ------------------------------------------------------------------DYWSTVKDK---------------
HsApoC4  73   -------------------------------------------------------------------SPSTFRGFMQTYYDD--------
HsApoD   219  --------------------------------------------------------------------SPELQAEAKSY-----------
HsApoE2  237  ---------------------------------------------------EMGSRTRDRLDEVKEQVAEVRAKLEEQAQQIRLQAEAFQ
HsApoF   222  -----------------------------------------YDLLMTMAGMSGGPMGLAISAALKPALRSGVQQLIQYYQDQKDAN----
HsApoH   247  ---------------------------------------------------CTKLGNWSAMPSCKASCKVPVKKATVVYQGER-------
HsApoJ   346  -------------------------------------------------SYQWKMLNTSSLLEQLNEQFNWVSRLANLTQGEDQYYLR--
HsApoL1  288  ----------------------------------------------ARANLQSVPHASASRPRVTEPISAESGEQVERVNEPSILEMSRG
HsApoL2  229  ------------------------------------------------ARANPQLGAYAPPPHVIGRISAEGGEQVERVVEGPAQAMSRG
HsApoL3  229  ------------------------------------------------------ARARARLPVTTWRISAGSGGQAERTIAGTTRAVSRG
HsApoL4  249  ------------------------------------------------------SKATVGRPLIAWRYVPINVVETLRTRGAPTRIVRKV
HsApoL5  248  ------------------------------------------------AKSNSGFMAMVKNFVAKRHIPFWTARGVQRAFEGTTLAMTNG
HsApoL6  193  ---------------------------------------------------NPRLANATKRLLTTGQVSSRSRVQVQKAFAGTTLAMTKN
HsApoM   143  ------------------------------------------------------FLLYNRSPHPPEKCVEEFKSLTSC------------
HsApoO   138  ---------------------------------------------------PPGFMGLAASLYYPQQAIVFAQVSGER------------
HsApoOL  187  --------------------------------------------------------KEESLPKPKEKTKLGSSSEIEVPAKTTHV-----
HsMTTP   655  ---------------------------------------------KAGLHGSQVVIEAQGLEALIAATPDEGEENLDSYAGMSAILFDVQ

HsApoA1  125  ------------------------------------------------------------------------------------------
HsApoA2  64   ------------------------------------------------------------------------------------------
HsApoA4  322  ME---------------QLRQKLGPHAGDVEGHLSF------------------------------------------------------
HsApoA5  299  -----------------TRAIDQETEE---------------------------------------------------------------
HsApoB48 1385 LRARYHMKADSVVDLLSYNVQGSGETTYDHKNTFTL------------------------------------------------------
HsApoB52 1527 AFVYTKNPNGYSFSIPVKVLADKFIIPGLKLNDLNSVLVMPTFHVPFTDLQVPSCKLDFREIQIYKKLRTSSFALNLPTLPEVKFPEVDV
HsApoC1  85   ------------------------------------------------------------------------------------------
HsApoC2  88   ------------------------------------------------------------------------------------------
HsApoC3  80   ------------------------------------------------------------------------------------------
HsApoC4  88   -----------------------------HLRDLGP------------------------------------------------------
HsApoD   231  ------------------------------------------------------------------------------------------
HsApoE2  277  AR----------------------------------------------------------------------------------------
HsApoF   267  -----------------ISQPETTKEGLRAISDVSD------------------------------------------------------
HsApoH   279  -----------------VKIQEKFKNGMLHGDKVSF------------------------------------------------------
HsApoJ   385  -----------------VTTVASHTSDSDVPSGVTE------------------------------------------------------
HsApoL1  332  -----------------VKLTDVAPVSFFLVLDVVY------------------------------------------------------
HsApoL2  271  -----------------TMIVGAATGGILLLLDVVS------------------------------------------------------
HsApoL3  265  -----------------ARILSATTSGIFLALDVVN------------------------------------------------------
HsApoL4  285  -----------------ARNLGKATSGVLVVLDVVN------------------------------------------------------
HsApoL5  290  -----------------AWVMGAAGAGFLLMKDMSS------------------------------------------------------
HsApoL6  232  -----------------ARVLGGVMSAFSLGYDLAT------------------------------------------------------
HsApoM   167  ------------------------------------------------------------------------------------------
HsApoO   165  ------------------------------------------------------------------------------------------
HsApoOL  216  ------------------------------------------------------------------------------------------
HsMTTP   701  LRPVTFFNGYSDLM---SKMLSASGDPISVVKGLIL------------------------------------------------------
```

```
HsApoA1  126  LEDLRQGLLPV-------------------------------------------------------------------------------
HsApoA2  65   FEKSKEQLTPL-------------------------------------------------------------------------------
HsApoA4  343  LEKDLRDK----------------------------------------------------------------------------------
HsApoA5  310  VQQQLAPPPPG-------------------------------------------------------------------------------
HsApoB48 1421 SCDGSLRHKFLDSNIKFSHVEKL----------GNNPVSKGLLIFDASSSWGPQMSASVHLDSKKKQHLFVKEVKIDGQFRVSSFYAKGT
HsApoB52 1617 LTKYSQPEDSLIPFFEITVPESQLTVSQFTLPKSVSDGIAALDLNAVANKIADFELPTIIVPEQTIEIPSIKFSVPAGIVIPSFQALTAR
HsApoC1  86   MVQDEQIE----------------------------------------------------------------------------------
HsApoC2  89   FTDQVLSV----------------------------------------------------------------------------------
HsApoC3  81   FSEFWDLD----------------------------------------------------------------------------------
HsApoC4  96   LTKAWFLE----------------------------------------------------------------------------------
HsApoD   231  FEKSKEQLTPL-------------------------------------------------------------------------------
HsApoE2  279  LKSWFEPL----------------------------------------------------------------------------------
HsApoF   287  LEETTTLASFISEV----------------------------------------------------------------------------
HsApoH   299  FCKNKEKKCSY-------------------------------------------------------------------------------
HsApoJ   405  VVVKLFDSDPI-------------------------------------------------------------------------------
HsApoL1  352  LVYESKHL----------------------------------------------------------------------------------
HsApoL2  291  LAYESKHL----------------------------------------------------------------------------------
HsApoL3  285  LVYESKHL----------------------------------------------------------------------------------
HsApoL4  305  LVQDSLDL----------------------------------------------------------------------------------
HsApoL5  310  FLQSWKHL----------------------------------------------------------------------------------
HsApoL6  252  LSKEWKHL----------------------------------------------------------------------------------
HsApoM   168  LDSKAFLLTP--------------------------------------------------------------------------------
HsApoO   166  LYDWGLRGYIV-------------------------------------------------------------------------------
HsApoOL  217  LKHSVPLPTEL-------------------------------------------------------------------------------
HsMTTP   734  LIDHSQELQLQ-------------------------------------------------------------------------------

HsApoA1  136  ------------------------------------------------------------------------LESFKVSFLSA-LEEYTK
HsApoA2  75   ------------------------------------------------------------------------IKKAGTELVN--FLSYFV
HsApoA4  350  ------------------------------------------------------------------------VNSFFSTFKEKESQDKTL
HsApoA5  320  ------------------------------------------------------------------------HSAFAPEFQQTDSGKVLS
HsApoB48 1501 YGLSCQRDPNTGRLNGESNLRFNSSYLQGTNQITGRYEDGTLSLTSTSDLQSGIIKNTASLKYENYELTLKSDTNGKYKNFAT-SNKMDM
HsApoB52 1707 FEVDSPVYNATWSASLKNKADYVETVLDSTCSSTVQFLEYELNVLGTHKIEDGTLASKTKGTFAHRDFSAEYEEDGKYEGLQEWEGKAHL
HsApoC1  93   ------------------------------------------------------------------------KKTSPGEADNI-PLVTQL
HsApoC2  96   ------------------------------------------------------------------------LKGEE-------------
HsApoC3  88   ------------------------------------------------------------------------PEVRPTSAVAA-------
HsApoC4  103  ------------------------------------------------------------------------SKDSLLKKTHS-LCPRLV
HsApoD   242  ------------------------------------------------------------------------IKKAGTELVN--FLSYFV
HsApoE2  286  ------------------------------------------------------------------------VEDMQRQWAGL-VEKVQA
HsApoF   300  ------------------------------------------------------------------------VSSAPYWGWAI-IKSYDL
HsApoH   309  ------------------------------------------------------------------------TEDAQCIDGTI-EVPKCF
HsApoJ   415  ------------------------------------------------------------------------TVTVPVEVSRK-NPKFME
HsApoL1  359  ------------------------------------------------------------------------HEGAKSETAEE-LKKVAQ
HsApoL2  298  ------------------------------------------------------------------------LEGAKSESAEE-LKKRAQ
HsApoL3  292  ------------------------------------------------------------------------HEGAKSASAEE-LRRQAQ
HsApoL4  312  ------------------------------------------------------------------------HKGEKSESAEL-LRQWAQ
HsApoL5  317  ------------------------------------------------------------------------EDGARTETAEE-LRALAK
HsApoL6  259  ------------------------------------------------------------------------KEGARTKFAEE-LRAKAL
HsApoM   177  ------------------------------------------------------------------------RNQEACELSNN-------
HsApoO   176  ------------------------------------------------------------------------IEDLWKENFQK-PGNVKN
HsApoOL  227  ------------------------------------------------------------------------SSEAKTKSEST-SGATQF
HsMTTP   744  ---SGLKANIEVQGGLAIDISGAMEFSLWYRESKTRVKNRVTVVITTDITVDSSFVKAGLETSTETEAGLEFISTVQFSQYPF-LVCMQM

HsApoA1  154  KLNTQ-------------------------------------------------------------------------------------
HsApoA2  92   ELGTQPATQ---------------------------------------------------------------------------------
HsApoA4  369  SLPELEQQQEQQQEQQQEQVQMLAPLES--------------------------------------------------------------
HsApoA5  339  KLQARLDDLWEDITHSLHDQGHSHLGDP--------------------------------------------------------------
HsApoB48 1590 TFSKQNA-LLRSEYQADYESLRFFSLLSGSLNSHGLELNADILGTDKINSGAHKATLRIGQDGISTSATTNLKCSLLVLENELNAELGLS
HsApoB52 1797 NIKSPAFTDLHLRYQKDKKGISTSAASPAVGTVGMDMDEDDDFSKWNFYYSPQSSPDKKLTIFKTELRVRESDEETQIKVNWEEEAASGL
HsApoC1  111  DLKGVVFRDISESEGETQD-----------------------------------------------------------------------
HsApoC2  101  ------------------------------------------------------------------------------------------
HsApoC3  99   ------------------------------------------------------------------------------------------
HsApoC4  121  CGDKDQG-----------------------------------------------------------------------------------
HsApoD   258  ELGTQPATQ---------------------------------------------------------------------------------
HsApoE2  304  AVGTSAAPVPSDNH----------------------------------------------------------------------------
HsApoF   318  DPGAGSLEI---------------------------------------------------------------------------------
HsApoH   327  KEHSSLA-FWKTDASDVKPC----------------------------------------------------------------------
HsApoJ   433  TVAEKALQEYRKKHREE-------------------------------------------------------------------------
HsApoL1  377  ELEEKLN-ILNNNYKILQADQEL-------------------------------------------------------------------
HsApoL2  316  ELEGKLN-FLTKIHEMLQPGQDQ-------------------------------------------------------------------
HsApoL3  310  ELEENLM-ELTQIYQRLNPCHTH-------------------------------------------------------------------
HsApoL4  330  ELEENLN-ELTHIHQSLKAG----------------------------------------------------------------------
HsApoL5  335  KLEQELD-RLTQHHRHLPQKASQTCSSSRGRAVRGSRVVKPEGSRSPLPWPVVEHQPRLGPGVALRTPKRTVSAPRMLGHQPAPPAPARK
HsApoL6  277  ELERKLT-ELTQLYKSLQQKVRSRARGVGKDLTGTCETEAYWKELREHVWMWLWLCVCLCVCVYVQFT----------------------
HsApoM   188  ------------------------------------------------------------------------------------------
HsApoO   194  SPGTK-------------------------------------------------------------------------------------
HsApoOL  245  MPDPKLM-DHGQSHPEDIDMYSTRS-----------------------------------------------------------------
HsMTTP   831  DKDEAPFRQFEKKYERLSTGRGYVSQKRKESVLAGCEFPLHQENSEMCKVVFAPQPDSTSSGWF--------------------------
```

```
HsApoA1  158  ------------------------------------------------------------------------------------------
HsApoA2  100  ------------------------------------------------------------------------------------------
HsApoA4  396  ------------------------------------------------------------------------------------------
HsApoA5  366  ------------------------------------------------------------------------------------------
HsApoB48 1679 GASMKLTTNGRFREHNAKFSLDGKAALTELSLGSAYQAMILGVDSKNIFNFKVSQEGLKLSNDMMGSYAEMKFDHTNSLNIAGLSLDFSS
HsApoB52 1887 LTSLKDNVPKATGVLYDYVNKYHWEHTGLTLREVSSKLRRNLQNNAEWVYQGAIRQIDDIDVRFQKAASGTTGTYQEWKDKAQNLYQELL
HsApoC1  129  ------------------------------------------------------------------------------------------
HsApoC2  101  ------------------------------------------------------------------------------------------
HsApoC3  99   ------------------------------------------------------------------------------------------
HsApoC4  127  ------------------------------------------------------------------------------------------
HsApoD   266  ------------------------------------------------------------------------------------------
HsApoE2  317  ------------------------------------------------------------------------------------------
HsApoF   326  ------------------------------------------------------------------------------------------
HsApoH   345  ------------------------------------------------------------------------------------------
HsApoJ   449  ------------------------------------------------------------------------------------------
HsApoL1  398  ------------------------------------------------------------------------------------------
HsApoL2  337  ------------------------------------------------------------------------------------------
HsApoL3  331  ------------------------------------------------------------------------------------------
HsApoL4  348  ------------------------------------------------------------------------------------------
HsApoL5  424  GRQAPGRHRQ--------------------------------------------------------------------------------
HsApoL6  343  ------------------------------------------------------------------------------------------
HsApoM   188  ------------------------------------------------------------------------------------------
HsApoO   198  ------------------------------------------------------------------------------------------
HsApoOL  268  ------------------------------------------------------------------------------------------
HsMTTP   894  ------------------------------------------------------------------------------------------

HsApoA1  158  ------------------------------------------------------------------------------------------
HsApoA2  100  ------------------------------------------------------------------------------------------
HsApoA4  396  ------------------------------------------------------------------------------------------
HsApoA5  366  ------------------------------------------------------------------------------------------
HsApoB48 1769 KLDNIYSSDKFYKQTVNLQLQPYSLVTTLNSDLKYNALDLTNNGKLRLEPLKLHVAGNLKGAYQNNEIKHIYAISSAALSASYKADTVAK
HsApoB52 1977 TQEGQASFQGLKDNVFDGLVRVTQEFHMKVKHLIDSLIDFLNFPRFQFPGKPGIYTREELCTMFIREVGTVLSQVYSKVHNGSEILFSYF
HsApoC1  129  ------------------------------------------------------------------------------------------
HsApoC2  101  ------------------------------------------------------------------------------------------
HsApoC3  99   ------------------------------------------------------------------------------------------
HsApoC4  127  ------------------------------------------------------------------------------------------
HsApoD   266  ------------------------------------------------------------------------------------------
HsApoE2  317  ------------------------------------------------------------------------------------------
HsApoF   326  ------------------------------------------------------------------------------------------
HsApoH   345  ------------------------------------------------------------------------------------------
HsApoJ   449  ------------------------------------------------------------------------------------------
HsApoL1  398  ------------------------------------------------------------------------------------------
HsApoL2  337  ------------------------------------------------------------------------------------------
HsApoL3  331  ------------------------------------------------------------------------------------------
HsApoL4  348  ------------------------------------------------------------------------------------------
HsApoL5  433  ------------------------------------------------------------------------------------------
HsApoL6  343  ------------------------------------------------------------------------------------------
HsApoM   188  ------------------------------------------------------------------------------------------
HsApoO   198  ------------------------------------------------------------------------------------------
HsApoOL  268  ------------------------------------------------------------------------------------------
HsMTTP   894  ------------------------------------------------------------------------------------------

HsApoA1  158  ------------------------------------------------------------------------------------------
HsApoA2  100  ------------------------------------------------------------------------------------------
HsApoA4  396  ------------------------------------------------------------------------------------------
HsApoA5  366  ------------------------------------------------------------------------------------------
HsApoB48 1859 VQGVEFSHRLNTDIAGLASAIDMSTNYNSDSLHFSNVFRSVMAPFTMTIDAHTNGNGKLALWGEHTGQLYSKFLLKAEPLAFTFSHDYKG
HsApoB52 2067 QDLVITLPFELRKHKLIDVISMYRELLKDLSKEAQEVFKAIQSLKTTEVLRNLQDLLQFIFQLIEDNIKQLKEMKFTYLINYIQDEINTI
HsApoC1  129  ------------------------------------------------------------------------------------------
HsApoC2  101  ------------------------------------------------------------------------------------------
HsApoC3  99   ------------------------------------------------------------------------------------------
HsApoC4  127  ------------------------------------------------------------------------------------------
HsApoD   266  ------------------------------------------------------------------------------------------
HsApoE2  317  ------------------------------------------------------------------------------------------
HsApoF   326  ------------------------------------------------------------------------------------------
HsApoH   345  ------------------------------------------------------------------------------------------
HsApoJ   449  ------------------------------------------------------------------------------------------
HsApoL1  398  ------------------------------------------------------------------------------------------
HsApoL2  337  ------------------------------------------------------------------------------------------
HsApoL3  331  ------------------------------------------------------------------------------------------
HsApoL4  348  ------------------------------------------------------------------------------------------
HsApoL5  433  ------------------------------------------------------------------------------------------
HsApoL6  343  ------------------------------------------------------------------------------------------
HsApoM   188  ------------------------------------------------------------------------------------------
HsApoO   198  ------------------------------------------------------------------------------------------
HsApoOL  268  ------------------------------------------------------------------------------------------
HsMTTP   894  ------------------------------------------------------------------------------------------
```

```
HsApoA1  158  ------------------------------------------------------------------------------------------
HsApoA2  100  ------------------------------------------------------------------------------------------
HsApoA4  396  ------------------------------------------------------------------------------------------
HsApoA5  366  ------------------------------------------------------------------------------------------
HsApoB48 1949 STSHHLVSRKSISAALEHKVSALLTPAEQTGTWKLKTQFNNNEYSQDLDAYNTKDKIGVELTGRTLADLTLLDSPIKVPLLLSEPINIID
HsApoB52 2157 FSDYIPYVFKLLKENLCLNLHKFNEFIQNELQEASQELQQIHQYIMALREEYFDPSIVGWTVKYYELEEKIVSLIKNLLVALKDFHSEYI
HsApoC1  129  ------------------------------------------------------------------------------------------
HsApoC2  101  ------------------------------------------------------------------------------------------
HsApoC3  99   ------------------------------------------------------------------------------------------
HsApoC4  127  ------------------------------------------------------------------------------------------
HsApoD   266  ------------------------------------------------------------------------------------------
HsApoE2  317  ------------------------------------------------------------------------------------------
HsApoF   326  ------------------------------------------------------------------------------------------
HsApoH   345  ------------------------------------------------------------------------------------------
HsApoJ   449  ------------------------------------------------------------------------------------------
HsApoL1  398  ------------------------------------------------------------------------------------------
HsApoL2  337  ------------------------------------------------------------------------------------------
HsApoL3  331  ------------------------------------------------------------------------------------------
HsApoL4  348  ------------------------------------------------------------------------------------------
HsApoL5  433  ------------------------------------------------------------------------------------------
HsApoL6  343  ------------------------------------------------------------------------------------------
HsApoM   188  ------------------------------------------------------------------------------------------
HsApoO   198  ------------------------------------------------------------------------------------------
HsApoOL  268  ------------------------------------------------------------------------------------------
HsMTTP   894  ------------------------------------------------------------------------------------------

HsApoA1  158  ------------------------------------------------------------------------------------------
HsApoA2  100  ------------------------------------------------------------------------------------------
HsApoA4  396  ------------------------------------------------------------------------------------------
HsApoA5  366  ------------------------------------------------------------------------------------------
HsApoB48 2039 ALEMRDAVEKPQEFTIVAFVKYDKNQDVHSINLPFFETLQEYFERNRQTIIVVLENVQRNLKHINIDQFVRKYRAALGKLPQQANDYLNS
HsApoB52 2247 VSASNFTSQLSSQVEQFLHRNIQEYLSILTDPDGKGKEKIAELSATAQEIIKSQAIATKKIISDYHQQFRYKLQDFSDQLSDYYEKFIAE
HsApoC1  129  ------------------------------------------------------------------------------------------
HsApoC2  101  ------------------------------------------------------------------------------------------
HsApoC3  99   ------------------------------------------------------------------------------------------
HsApoC4  127  ------------------------------------------------------------------------------------------
HsApoD   266  ------------------------------------------------------------------------------------------
HsApoE2  317  ------------------------------------------------------------------------------------------
HsApoF   326  ------------------------------------------------------------------------------------------
HsApoH   345  ------------------------------------------------------------------------------------------
HsApoJ   449  ------------------------------------------------------------------------------------------
HsApoL1  398  ------------------------------------------------------------------------------------------
HsApoL2  337  ------------------------------------------------------------------------------------------
HsApoL3  331  ------------------------------------------------------------------------------------------
HsApoL4  348  ------------------------------------------------------------------------------------------
HsApoL5  433  ------------------------------------------------------------------------------------------
HsApoL6  343  ------------------------------------------------------------------------------------------
HsApoM   188  ------------------------------------------------------------------------------------------
HsApoO   198  ------------------------------------------------------------------------------------------
HsApoOL  268  ------------------------------------------------------------------------------------------
HsMTTP   894  ------------------------------------------------------------------------------------------

HsApoA1  158  ----------------------------------------------------
HsApoA2  100  ----------------------------------------------------
HsApoA4  396  ----------------------------------------------------
HsApoA5  366  ----------------------------------------------------
HsApoB48 2129 FNWERQVSHAKEKLTALTKKYRITENDIQIALDDAKINFNEKLSQLQTYMIQ
HsApoB52 2337 SKRLIDLSIQNYHTFLIYITELLKKLQSTTVMNPYMKLAPGELTIIL-----
HsApoC1  129  ----------------------------------------------------
HsApoC2  101  ----------------------------------------------------
HsApoC3  99   ----------------------------------------------------
HsApoC4  127  ----------------------------------------------------
HsApoD   266  ----------------------------------------------------
HsApoE2  317  ----------------------------------------------------
HsApoF   326  ----------------------------------------------------
HsApoH   345  ----------------------------------------------------
HsApoJ   449  ----------------------------------------------------
HsApoL1  398  ----------------------------------------------------
HsApoL2  337  ----------------------------------------------------
HsApoL3  331  ----------------------------------------------------
HsApoL4  348  ----------------------------------------------------
HsApoL5  433  ----------------------------------------------------
HsApoL6  343  ----------------------------------------------------
HsApoM   188  ----------------------------------------------------
HsApoO   198  ----------------------------------------------------
HsApoOL  268  ----------------------------------------------------
HsMTTP   894  ----------------------------------------------------
```

Fig. S9. Alignment of 11 fly sequences with ApoLpp and ApoLTP separated plus an outgroup.

```
DmApoLppI      1  -------------------------------SIVDDVSKISKKYKMYGVKNVQDLNLDVSLKLFGSELAFLSLGDNIPSS
DmApoLppII     0  --------------------------------------------------------------------------------
DmApoLTPI      1  RKAQEQARASEEEVDEDYDFEESLEGNKRQKRDVSTTRKQEIDRNVDSLGYKLKYDYNNPRAQFGLRVFGNDLRYFNVES
DmApoLTPII     0  --------------------------------------------------------------------------------
DmCG31659      0  --------------------------------------------------------------------------------
DmCvD          0  --------------------------------------------------------------------------------
DmFabp         0  --------------------------------------------------------------------------------
DmGlaz         0  --------------------------------------------------------------------------------
DmMic26-27     0  --------------------------------------------------------------------------------
DmNlaz         0  --------------------------------------------------------------------------------
DmMtp          0  --------------------------------------------------------------------------------

DmApoLppI     50   LDDIINYFSTSFEKAKQELSSFEKQFSSHHLFLDTDLAYPTSIGVPLELVAQGF-AATKVDLAVSL---DINAILEQNWQ
DmApoLppII     1   ------------------------MARMKYNIALIGILASVLLTIAVNAENACNLGCPKSDNGLLKYIPGNYYDYSFDSI
DmApoLTPI     81   LVEVMALAAKFNPFQQAKNVLSGKEFTYTKSRVFLDASYTVPLAVGLPLAIHAF-GASSIDLRVSG---NLDEMDPPTDW
DmApoLTPII     1   ----------------------MGGLKPQAAIWLLLLIAHTQAVRENPLKDPRICGRPQCDSKSAKF--NYGEQLYKYQY
DmCG31659      1   -------------------------------MIKWKIFVPAILYLQSSMAMRAFHGACPSNMTAVG---DLDMDRFKGKW
DmCvD          1   -------------------MRLLKCSVFVYLFLIFDAGHAFSIIGLNKQMLYEYEGNVLVGAKPQD---EGHQAPPTTGW
DmFabp         1   ----------------------MSFVGKKYKLDKSENFDEYMKELGVGLVTRKM-GNSLSP--------TVEVTLEGDTY
DmGlaz         1   ------------------------MMSGQPLGSRVWLLSGVLLVTFAGTDAYGF-GRCPNYPSMP----KFNMSRVLGHW
DmMic26-27     1   ---------------------MLRKTATMGIMAAVAVKAAPEPQKPASSAADCSLVCRPSELPIYG---SLRKTEPKPER
DmNlaz         1   ----------------------MNHHSSSHLLLLISVVFGAVWVAHAQVP---FPGKCP-DVKLLD---TFDAEAYMGVW
DmMtp          1   --------------------MENKNKKCLRTLLLLALFLGLLEDGKTALIAPNSQQIFKLQNQVILQELGRDSSSAETSY

DmApoLppI    126   KAKYRLKFV---PSVDINANVQIGFNAQVLSTGLRVVSSAHSATGSDITVAVISDGEGFNVDLELPREKLELINFNVDTE
DmApoLppII    56   LTIGASSDV---PNDSDDTSLKVSGSAKIFAKGNCGYTLQLSSVKVTNTKESVEKKILNSIQKPV---------------
DmApoLTPI    157   HFDVEGQFK---PSVSVDVITTMQTDMFWEQSGIKVKSNLYSNSEL-VAKLKVRGRNLVSFSFDLPRDKNEIFSVRSELL
DmApoLTPII    56   TVAVRTEFA---GSGDNSSDLLLKSDLEIFFPKPCEGYLRINDAKLYDTLDELNDNESDSSEKT----------------
DmCG31659     46   YTHSIYPHL---SLRVEKCQST----------------------------------------------------------
DmCvD         58   IVRGKLTLQ---RQSELVLAAALVIDDVTLNNSGEKFLQNKEMYPPYKPF------------------------------
DmFabp        49   TLTTTSTFK-----------------------------------------------------------------------
DmGlaz        51   YEVERSFYL---PEIASGCTTF----------------------------------------------------------
DmMic26-27    56   HPPQDSVLH---KNLEAGVRYV----------------------------------------------------------
DmNlaz        51   YEYAAYPFA---FEIGKKCIYA----------------------------------------------------------
DmMtp         60   TFETDLKINSVWSGDEDQLLEVFISGSKVDASGKARSITRIPDRPFYISLVR----------------------------

DmApoLppI    203   LYVAEQDKQKAIALKGNKKNKNSQPSEICFNQLELVGLNICIKSSTSLSEVQAGNGNVAERGLSVSEKFHLSRPFNFAVY
DmApoLppII   118   --------------------------------------------------------------------------------
DmApoLTPI    233   VQKREEQLPQAGIANRSANSTCTWPV-----------LDQAIGLQMCSHYSVPDLSNATEIYPSLLLAGPLNFSLILKKS
DmApoLTPII   117   --------------------------------------------------------------------------------
DmCG31659     65   --------------------------------------------------------------------------------
DmCvD        105   --------------------------------------------------------------------------------
DmFabp        58   --------------------------------------------------------------------------------
DmGlaz        70   --------------------------------------------------------------------------------
DmMic26-27    75   --------------------------------------------------------------------------------
DmNlaz        70   --------------------------------------------------------------------------------
DmMtp        112   --------------------------------------------------------------------------------

DmApoLppI    283   LTTERKFTFKGIHTQEAFSQKWKLDYSTPGSKVSHDTTVVYELGNKPKTFSRLSFDNSQCHFAVEGGINNDKNELVVYGQ
DmApoLppII   118   --------------------------------------------------------------------------------
DmApoLTPI    302   DLSAKKYVFEYKWDQQEEDNNFSLVFTTPGSKVPR--VLVANVTKVPDAFNASVAFVNGPNRVSAGCSYDGNPDFRRLDI
DmApoLTPII   117   --------------------------------------------------------------------------------
DmCG31659     65   --------------------------------------------------------------------------------
DmCvD        105   --------------------------------------------------------------------------------
DmFabp        58   --------------------------------------------------------------------------------
DmGlaz        70   --------------------------------------------------------------------------------
DmMic26-27    75   --------------------------------------------------------------------------------
DmNlaz        70   --------------------------------------------------------------------------------
DmMtp        112   --------------------------------------------------------------------------------

DmApoLppI    363   YEQDKEIKKSKIGF------------------------------------SKNGNEYKPLIEIQDNNGISNSINGYHADG
DmApoLppII   118   --------------------------------------------------------------------------------
DmApoLTPI    380   YLDTNGNRSLDLGMELRRHQDFTAWIYNPRMLLAINGVNITGLAGTVKVNEKNGIKQHDVDLSFETKKLQAVIKGNVVQS
DmApoLTPII   117   -------------------------------------------------------KEYDYYDNLANEESESQNYDNMHPK
DmCG31659     65   --------------------------------------------------------------------------------
DmCvD        105   --------------------------------------------------------------------------------
DmFabp        58   --------------------------------------------------------------------------------
DmGlaz        70   --------------------------------------------------------------------------------
DmMic26-27    75   --------------------------------------------------------------------------------
DmNlaz        70   --------------------------------------------------------------------------------
DmMtp        112   --------------------------------------------------------------------------------
```

```
DmApoLppI    407   KIVVKKNSNNIERYNFENFQVSNSNNAHVAVNGWSDVGTNSLTSELRISLDHQTFLIKENLKLENGLYEAGFFINDE-HS
DmApoLppII   118   ------------QFTLVSGILEPQICSDSSDLDYSLNIKRAVVSLLQSGIEA------EHEVDVFGMCPTHTSTSKV-GN
DmApoLTPI    460   EITTSTNMTIKYRFQANKIEEINFAGKLVNNGDKSKTEYRGNMKLQTSAYPKLNFASESTWLSLQGHTEGMITYNNA-PD
DmApoLTPII   142   SSDFNVDLTKNLLRFAFHDGLISEVCPQEQETPWVLNIKKGILSAFQNTMMRFDVDANTTETDVSGQCQVQYALEDTDSS
DmCG31659     65   --------------------------------------------------------------------------------
DmCvD        105   ------------KIALTKDGAISHVVFKEGDPIWSMNFKRAIASVLQFQMKSSGAFV-VDELGIHGTCRTEYFVSNR-TN
DmFabp        58   --------------------------------------------------------------------------------
DmGlaz        70   --------------------------------------------------------------------------------
DmMic26-27    75   --------------------------------------------------------------------------------
DmNlaz        70   --------------------------------------------------------------------------------
DmMtp        112   -------------------GQPDKVIAHTSKDQSLLNLERGIASLLQLRLDASQ----EEELDVSGLCRVSYNVKSS-TK

DmApoLppI    486   PENIYGSSIHLTIADQSYALKTNGKAAAWSIGSDGSFNFQKLADSNSARAGSLVENVEIQYKNKQVGGIKIMSNFDVNKM
DmApoLppII   179   ANIITKARNLNSCSHREQINSGLV--------------------------------------------------------
DmApoLTPI    539   YVNPNYTSLVRLIFARSHSEDSFLDGTQTRASLELKLPRSKIDYRILVKHKEHIKN---GTEHNVIVGLKYTPEKEITGL
DmApoLTPII   222   YVTIRKTKDINSCRQRYATHSVLQ--------------------------------------------------------
DmCG31659     65   --------------------------------------------------------------------------------
DmCvD        171   YISIRKTPEVKTCKPYSEAVHTTRSNVPPNTCEFDHQKSVIIGNEAIYGMSPHNETGYYLSMAHAKGTTLIHTFESTGEA
DmFabp        58   --------------------------------------------------------------------------------
DmGlaz        70   --------------------------------------------------------------------------------
DmMic26-27    75   --------------------------------------------------------------------------------
DmNlaz        70   --------------------------------------------------------------------------------
DmMtp        168   VEKTKRDCSLWDLRVNYNPEEALGVTQQAQETVFYELSSEGTLLHAESQENHRLNLAAKPDVGSFVKSSLILQHVSQGSE

DmApoLppI    566   DVDVEISREQKIGSIIVKYESNQRHAQDYSLEASAKINKHSIDVISKCDFNGNVYVVDNSLVTSWGTLLSAKG-------
DmApoLppII   203   --------------------------------------------------------------------------------
DmApoLTPI    616   FSVHLPRRNLFAIDAYMNVTVPEFNSCTASLKVNEKATKDYIIFINGSWFTGHSVAVKANYKDRSSRVQALHHLKMIVES
DmApoLTPII   246   --------------------------------------------------------------------------------
DmCG31659     65   --------------------------------------------------------------------------------
DmCvD        251   QFINSELLLNFLNETPIDNPIDIETSMAAEPSNLELQRLDPNDPTGGR--------------------------------
DmFabp        58   --------------------------------------------------------------------------------
DmGlaz        70   --------------------------------------------------------------------------------
DmMic26-27    75   --------------------------------------------------------------------------------
DmNlaz        70   --------------------------------------------------------------------------------
DmMtp        248   EVKQLQLGSL----------------------------------------------------------------------

DmApoLppI    639   --------------------------------EIGQRYSAQDININIQGNVQISGKDKVTQWILKVIGTPDKTNSDFRIS
DmApoLppII   203   --------------------------------------------------------------------------------
DmApoLTPI    696   PSFNITSLNIIYRRKQLLIFYDIQAKYDQDPYGLTIQYASNAHNRNTNAEVRLKVKERDYWINAKLLSEQPKLLQLEIHM
DmApoLTPII   246   --------------------------------------------------------------------------------
DmCG31659     65   --------------------------------------------------------------------------------
DmCvD        299   --------------------------------------------------------------------------------
DmFabp        58   --------------------------------------------------------------------------------
DmGlaz        70   --------------------------------------------------------------------------------
DmMic26-27    75   --------------------------------------------------------------------------------
DmNlaz        70   --------------------------------------------------------------------------------
DmMtp        258   --------------------------------------------------------------------------------

DmApoLppI    687   RDTSELIKLTSESQHPQDKISFAKLNLIVKNQLTAKGEFRVAKNGKGDFTASIDTLKTEPKHKLEIESKFHIQSPKYDID
DmApoLppII   203   --------------------------------------------------------------------------------
DmApoLTPI    776   DKIRDVHIQVGLLNVDKRKELSLELKWDANRDPSQRLGLLAEYNSPGTKHYDGNLMITYPERTIHFGFNSFTGGPKYFGK
DmApoLTPII   246   --------------------------------------------------------------------------------
DmCG31659     65   --------------------------------------------------------------------------------
DmCvD        299   --------------------------------------------------------------------------------
DmFabp        58   --------------------------------------------------------------------------------
DmGlaz        70   --------------------------------------------------------------------------------
DmMic26-27    75   --------------------------------------------------------------------------------
DmNlaz        70   --------------------------------------------------------------------------------
DmMtp        258   --------------------------------------------------------------------------------

DmApoLppI    767   ASLTLDGKRKVHLKSE----------------------------------------------------------------
DmApoLppII   203   --------------------------------------------------------------------------------
DmApoLTPI    856   VHASWSINEVIEFEYEAGILPGHTLHNWVKAELRTPFDGWRVNSLDAGIYSLKNLILVNSTLFWADDQKLQVGYKSDYDV
DmApoLTPII   246   --------------------------------------------------------------------------------
DmCG31659     65   --------------------------------------------------------------------------------
DmCvD        299   --------------------------------------------------------------------------------
DmFabp        58   --------------------------------------------------------------------------------
DmGlaz        70   --------------------------------------------------------------------------------
DmMic26-27    75   --------------------------------------------------------------------------------
DmNlaz        70   --------------------------------------------------------------------------------
DmMtp        258   --------------------------------------------------------------------------------
```

```
DmApoLppI    783  ------------------------------------------------NTIEKLKFSTKNIGEANDKIIAFEANGSLKGE
DmApoLppII   203  --------------------------------------------------------------------------------
DmApoLTPI    936  NDQLMSFDVRFGINSTIRDIPTINVKVKHWMDVKKVDTELYLGYSGQNDTFNTYSMDSSWEIEKNQRYNNYSGLVHLVSP
DmApoLTPII   246  --------------------------------------------------------------------------------
DmCG31659     65  --------------------------------------------------------------------------------
DmCvD        299  --------------------------------------------------------------------------------
DmFabp        58  --------------------------------------------------------------------------------
DmGlaz        70  --------------------------------------------------------------------------------
DmMic26-27    75  --------------------------------------------------------------------------------
DmNlaz        70  --------------------------------------------------------------------------------
DmMtp        258  --------------------------------------------------------------------------------

DmApoLppI    815  LRGNGEIQGTFIFNAPDGRVIDGSINRKISTNAKSGLSQGNIDAQLSDTPF-----------------------------
DmApoLppII   203  --------------------------------------------------------------------------------
DmApoLTPI   1016  FKGYEKGGLVAHFSLSDQRVVSGAASLNFDLREFTLTMNGYVKKFTDNMLTVNITTPLEKFGTINARFGLNEKKRHAVAE
DmApoLTPII   246  --------------------------------------------------------------------------------
DmCG31659     65  --------------------------------------------------------------------------------
DmCvD        299  --------------------------------------------------------------------------------
DmFabp        58  --------------------------------------------------------------------------------
DmGlaz        70  --------------------------------------------------------------------------------
DmMic26-27    75  --------------------------------------------------------------------------------
DmNlaz        70  --------------------------------------------------------------------------------
DmMtp        258  --------------------------------------------------------------------------------

DmApoLppI    866  ----------------------------------------------------GSNKKRSISLIGKLDRLNTKTKEFSANS
DmApoLppII   203  --------------------------------------------------------------------------------
DmApoLTPI   1096  VRAPTAALGVEVLADIKNLLNFDVKLSVATPIESFQQAAIFALFNPERVDMRGLWNNVTLGFTGVWHMQNITDFEYSYHV
DmApoLTPII   246  --------------------------------------------------------------------------------
DmCG31659     65  --------------------------------------------------------------------------------
DmCvD        299  --------------------------------------------------------------------------------
DmFabp        58  --------------------------------------------------------------------------------
DmGlaz        70  --------------------------------------------------------------------------------
DmMic26-27    75  --------------------------------------------------------------------------------
DmNlaz        70  --------------------------------------------------------------------------------
DmMtp        258  --------------------------------------------------------------------------------

DmApoLppI    894  NLVYTAFNGEKSEISYQIKQQPNGDAKNIDFSLKAYGNPLPQPFEIAFALGDYSAQHAVVSITSKYGEIFSVSANGNYNN
DmApoLppII   203  --------------------------------------------------------------------------------
DmApoLTPI   1176  FTPLAGFEENGFIVQLLKRKEFVFQLHGKMSNYKLGVKINGEPKSDLVNQLGSNKMELEMLYDADFKPLNAETDYKPDAD
DmApoLTPII   246  --------------------------------------------------------------------------------
DmCG31659     65  --------------------------------------------------------------------------------
DmCvD        299  --------------------------------------------------------------------------------
DmFabp        58  --------------------------------------------------------------------------------
DmGlaz        70  --------------------------------------------------------------------------------
DmMic26-27    75  --------------------------------------------------------------------------------
DmNlaz        70  --------------------------------------------------------------------------------
DmMtp        258  --------------------------------------------------------------------------------

DmApoLppI    974  NQALEYGLQANIEIPKSTLKSLEINSHGKVLKSLIGNENAAYNVEFFLDSKTSLGQYARVNTVWNGTANDGSYDFEAQTN
DmApoLppII   203  --------------------------------------------------------------------------------
DmApoLTPI   1256  EEYFSYFTNFQVDTLVWPTIVGNVDIQEIIDFYLVVGHVELPQGKVEFKDRLHYPDYINVHNLLTVTTPFAVAKNIKSIV
DmApoLTPII   246  --------------------------------------------------------------------------------
DmCG31659     65  --------------------------------------------------------------------------------
DmCvD        299  --------------------------------------------------------------------------------
DmFabp        58  --------------------------------------------------------------------------------
DmGlaz        70  --------------------------------------------------------------------------------
DmMic26-27    75  --------------------------------------------------------------------------------
DmNlaz        70  --------------------------------------------------------------------------------
DmMtp        258  --------------------------------------------------------------------------------

DmApoLppI   1054  NMESPLKFNGKYHRKQTGNIKDGDLTGKQTYVLNAQYGAQYVKMDASLGYGAEKVDIAYVIDSSFDSVKDIKVNIRTFKP
DmApoLppII   203  --------------------------------------------------------------------------------
DmApoLTPI   1336  EYHVDLNFNAFYERVKFIVNDDKDNTQELGFVFNYTALQDNVKPKAHDVQVTLLTPYEMLHEIYVHGHIELDDNAYKGNI
DmApoLTPII   246  --------------------------------------------------------------------------------
DmCG31659     65  --------------------------------------------------------------------------------
DmCvD        299  --------------------------------------------------------------------------------
DmFabp        58  --------------------------------------------------------------------------------
DmGlaz        70  --------------------------------------------------------------------------------
DmMic26-27    75  --------------------------------------------------------------------------------
DmNlaz        70  --------------------------------------------------------------------------------
DmMtp        258  --------------------------------------------------------------------------------
```

```
DmApoLppI   1134   LDDSTYVVTALFKQTDKSYGLDTTFYHSAHKKGVDIRLDLLKEKPIIISSIAELLGDRKGKVLFEILNLADLDIKINSEA
DmApoLppII   203   --------------SGKVNEKAGITSSLLLQANYIKESRIVNHLIENVQLTETYKFIGNTKRNSDISA------------
DmApoLTPI   1416   SAVTAHTHLSMAASIENEDNFLETSVGIQLETDAIPHYGCQVYFKKDFSAVDKAIDIRFEVTDNGTLNQLHISTDWHTDP
DmApoLTPII   246   --------------TTPYTFRDDKTIWPILKSQSHCNLTIDNNVYKEIKCLETHLLVPFSNASSGALT------------
DmCG31659     65   --------------------------------------DFIEKEENKFSVVARELNTQTGTVKM----------------
DmCvD        299   --------------SPQQQETLIAQAGTLLDSLAEALETTEFKFSEPYDSTLSDVIKLLSEMDFDSLTKLYREVDIGTSY
DmFabp        58   -----------------------------------------------TSAISFKLGVEFDEETLDGR-------------
DmGlaz        70   ----------------------------------------------QFEPYNKGEQSKFSNFKLAVAI------------
DmMic26-27    75   ----------------------------------------REEVQSGYKAVADQAGIVGHYVETA---------------
DmNlaz        70   ----------------------------------------------NYSLIDNSTVSVVN--------------------
DmMtp        258   --------------DKAIQSLLEWYRVFELESDVDGMISAIKEQTLEDQLKASLTELQSADVGKSSLALAYVKL------

DmApoLppI   1214   SYV----------------------SIDEFYIIVNWSSKKLKLDGYELEARAQSKNIKIQLKNE----------------
DmApoLppII   257   -------------------------------------------------------KVVTILKLK----------------
DmApoLTPI   1496   SYIVNANGRIKTTMLPLQMASTSVLVIQGQNPHLNFDLNLLSQNGQSIAYGARANKKKDVFNIEVWTPMKNFRNISMHGT
DmApoLTPII   300   -------------------------------------------------------TSTSRLKLD----------------
DmCG31659     91   -------------------------------------------------------RKADILNVE----------------
DmCvD        365   R----------------------------QETIRNIFHEIIPRIGTKASVFLTHHLVLNKLTKP----------------
DmFabp        78   -------------------------------------------------------NVKSIITLD----------------
DmGlaz        92   -------------------------------------------------------KNINRITGN----------------
DmMic26-27   100   -------------------------------------------------------KAHTQSTID----------------
DmNlaz        84   -------------------------------------------------------AAINRFTGQ----------------
DmMtp        318   -------------------------------------------------------IPLARITRQ----------------

DmApoLppI   1256   -------------NGIIFSGTATYALKKELNKTIIDGQGKVQYQGKALSGNFKLTRQHFDFGTDREVGFSYTFMGNLGSK
DmApoLppII   266   --------------------------------------------------------------------------------
DmApoLTPI   1576   AIRSPRDPGRYDVSGFLYRNMATYEVTGAVRMTNSLPIDVVLRVQPKAGGRDGVIELNIHEAGPKKIRFSFSAIEDGKMC
DmApoLTPII   309   --------------------------------------------------------------------------------
DmCG31659    100   --------------------------------------------------------------------------------
DmCvD        401   --------------------------------------------------------------------------------
DmFabp        87   --------------------------------------------------------------------------------
DmGlaz       101   --------------------------------------------------------------------------------
DmMic26-27   109   --------------------------------------------------------------------------------
DmNlaz        93   --------------------------------------------------------------------------------
DmMtp        327   --------------------------------------------------------------------------------

DmApoLppI   1323   NGLGTLKITNKEFNTKFSVCEEKRQCTNLIVQSIVSIDEQ----------------------------------------
DmApoLppII   266   --------------------------------------------------------------------------------
DmApoLTPI   1656   QMSGGYSVSKTNGAMDFSVLVESTEPEIARINFYGNLSPNSEGSLVGDLSLETPWKALGIDTVHLHSDVGFLNKGGHIVG
DmApoLTPII   309   --------------------------------------------------------------------------------
DmCG31659    100   --------------------------------------------------------------------------------
DmCvD        401   --------------------------------------------------------------------------------
DmFabp        87   --------------------------------------------------------------------------------
DmGlaz       101   --------------------------------------------------------------------------------
DmMic26-27   109   --------------------------------------------------------------------------------
DmNlaz        93   --------------------------------------------------------------------------------
DmMtp        327   --------------------------------------------------------------------------------

DmApoLppI   1363   -----------------------------------------------------------------------------KLD
DmApoLppII   266   --------------------------------------------------------------------------------
DmApoLTPI   1736   EYKIGQYIGRGSCLWSWILAEDMQLVLENYLERPNAKPRIVHASAKYQNPGQTFTQLQAGGRLSVDSKWNLDVNGSAEYK
DmApoLTPII   309   --------------------------------------------------------------------------------
DmCG31659    100   --------------------------------------------------------------------------------
DmCvD        401   --------------------------------------------------------------------------------
DmFabp        87   --------------------------------------------------------------------------------
DmGlaz       101   --------------------------------------------------------------------------------
DmMic26-27   109   --------------------------------------------------------------------------------
DmNlaz        93   --------------------------------------------------------------------------------
DmMtp        327   --------------------------------------------------------------------------------

DmApoLppI   1366   AVEHTTLIIVDLRDFGYPYEFELKSQNTRQGLKYQYHLDSFIITGNNFKYQFTANVQPTSSTIKLALPKRQILFETTQKI
DmApoLppII   266   -----------------------------NPSGTKANSPGTGSTVRSLIFQRPETYTSKN--INALKTILSDLVDSTGDY
DmApoLTPI   1816   SVDDFKFRVITALPLPVGDRHQLSASYQGNVISQQFNNPDFVLEASYESFEAQNKLLSRISYKNATNNLKGLGHVEWGKI
DmApoLTPII   309   ------------------------GVESYSAGEFLEQNPELVERRATLVFDHTPAVKPSHDEIKAARELLVEMCRVGFPN
DmCG31659    100   --------------------------------------PEF---------------------------------------
DmCvD        401   ------QIAVQLLIPMPFHIFELSAELVQKCEDFLNIGPDRPDVRQAAILSFATLIHNVYVAKGIDKEKFEEYVQKYFNA
DmFabp        87   --------------------------------------------------------------------------------
DmGlaz       101   --------------------------------------PNVNI-------------------------------------
DmMic26-27   109   ----------------------------------MLNEPQNSLHRS----------------------------------
DmNlaz        93   --------------------------------------PSNVT-------------------------------------
DmMtp        327   --------------EQFEDLLTEHAEVLPQLVDLLGAVQTFDAHNATFGFLYKESETTSEQLDLLEKYLQSLAVATHPDR
```

```
DmApoLppI   1446   PADGSLFGRYEQTASFFIDK------------------------------------------------------------
DmApoLppII   315   VKKETAKKFVEFIRLLRQS-------------------------------------------------------------
DmApoLTPI   1896   QNLSVVEGDFELLHKQGAQREFSAKIITPKFKNEHTFALTGSYDLEKSGHHNVVGSLDYPASRRITDLDVSVSSLSNMHG
DmApoLTPII   365   IQREFIDVFTNFLQTSKSL-------------------------------------------------------------
DmCG31659    103   --------------------------------------------------------------------------------
DmCvD        475   YLSDRDFDQKMLYLQGL---------------------------------------------------------------
DmFabp        87   --------------------------------------------------------------------------------
DmGlaz       106   --------------------------------------------------------------------------------
DmMic26-27   121   --------------------------------------------------------------------------------
DmNlaz        98   --------------------------------------------------------------------------------
DmMtp        393   KIVEHLFGLLEQESIKKHLKLRESVIQTV---------------------------------------------------

DmApoLppI   1466   ------------------------------------LQKPDDVARFSAIVDVTGTERVAFNANGKLKFEHPTIRPLSISG
DmApoLppII   334   --------------------------------------------------------------------------------
DmApoLTPI   1976   IFNSTLPTFLNVSWLKTDFNFTTNNGKSYRYCRCFWPQDTAYFKLNSNYDSDSSNFNHNLNGNVEIEVPLATRHRADIVY
DmApoLTPII   384   --------------------------------------------------------------------------------
DmCG31659    103   --------------------------------------------------------------------------------
DmCvD        492   --------------------------------------------------------------------------------
DmFabp        87   --------------------------------------------------------------------------------
DmGlaz       106   --------------------------------------------------------------------------------
DmMic26-27   121   --------------------------------------------------------------------------------
DmNlaz        98   --------------------------------------------------------------------------------
DmMtp        422   --------------------------------------------------------------------------------

DmApoLppI   1510   QLNGDVNQQIASAEVIFDIFRLPEQKVVGNSELRNSRSQNGFNIAYITTVKSAGLQFQYQINSNAAVDIEAHEYNIGLEL
DmApoLppII   334   --------------------------------------------------------------------------------
DmApoLTPI   2056   GLQKRRNQDAGNVKVVYNEKQVLDGKYKRLEQAKAPIYKETTDISLENEVKPLGIHFVSTRDASDPAGSQDVKHIEIYEL
DmApoLTPII   384   --------------------------------------------------------------------------------
DmCG31659    103   --------------------------------------------------------------------------------
DmCvD        492   --------------------------------------------------------------------------------
DmFabp        87   --------------------------------------------------------------------------------
DmGlaz       106   --------------------------------------------------------------------------------
DmMic26-27   121   --------------------------------------------------------------------------------
DmNlaz        98   --------------------------------------------------------------------------------
DmMtp        422   --------------------------------------------------------------------------------

DmApoLppI   1590   NNGEI-----DVKAISFLNKEKFEISLSESNKHIIYIVGDFSKQNHYAKLNTKVQILDK---------------------
DmApoLppII   334   --------------------------------------------------------------------------------
DmApoLTPI   2136   RNTQNFNLTGELHSRATLKAQDFKVVAIHPNRAVVLSTKYEDVSPEVVRHHSKLELSETAWIGYNLELGNFSKVGNESQS
DmApoLTPII   384   --------------------------------------------------------------------------------
DmCG31659    103   --------------------------------------------------------------------------------
DmCvD        492   --------------------------------------------------------------------------------
DmFabp        87   --------------------------------------------------------------------------------
DmGlaz       106   --------------------------------------------------------------------------------
DmMic26-27   121   --------------------------------------------------------------------------------
DmNlaz        98   --------------------------------------------------------------------------------
DmMtp        422   --------------------------------------------------------------------------------

DmApoLppI   1644   ---------------NPIEITSEVQPNSAKIILKRQDFIDGTAEVKLGKEFKVDVIGSGKQLF--------NGRVALDAT
DmApoLppII   334   ---------------DSETLLELAAFPHPNKVLARKVYLDGLFRTSTAESARVILKQLSKF----------DEKEKLLAI
DmApoLTPI   2216   FALEIFYPKRNLSSSGQYYMTDTNFNSDLSFQWLGGNYDQQPKIIHSNLQWKAEPLHRGDREH--------RTIALTVAH
DmApoLTPII   384   ---------------DYKTLSVLLQRSASTCEQGRNHLLESLPFIGSTASYKVMRDQIINEKL--------TKQMAHDWM
DmCG31659    103   ---------------GRYVLG-----------------------------------------------------------
DmCvD        492   ---------------NNLQLGNVANYLEPIVQDPNEHEDLKFQAAWTTLALADRRAERIYEVYWPIFESRNASLELRVAA
DmFabp        87   ---------------GNKLTQEQKGD------------------------------------------------------
DmGlaz       106   ---------------GYATPENSR--------------------------------------------------------
DmMic26-27   121   ---------------GAIVVGGLAGFIFAARGGF----------------------------------------------
DmNlaz        98   ---------------GQAKVLGPGQ-------------------------------------------------------
DmMtp        422   ---------------ATLTRQSGLDVEDPLLKEVRSYLLQGLTSKEPTLYIRALQNLQDPATIEALLEHAQTGEAPNLSV

DmApoLppI   1701   NFLQTNYFINEDHLNGFWHIVESEINKDSEYISENIKERLKKSRQVTDKIVKL---------------------------
DmApoLppII   389   LSLNIVKSVDKETLNQAASQLLPN--------------------------------------------------------
DmApoLTPI   2288   PLLEKDINCKATYYRGLRDLLRTHLTIDYSEYPDQLIELGAQLTDRYSELGHTNYTFHVYGKHIASELDVQLNGTLAAMN
DmApoLTPII   441   TALSFITRPDEETLETFHSILEY---------------------------------------------------------
DmCG31659    109   --------------------------------------------------------------------------------
DmCvD        557   VTLLLISNPTAARLISIHRIIQSETDPHMINYYRTTVTS-----------------------------------------
DmFabp        98   --------------------------------------------------------------------------------
DmGlaz       115   --------------------------------------------------------------------------------
DmMic26-27   140   --------------------------------------------------------------------------------
DmNlaz       108   --------------------------------------------------------------------------------
DmMtp        487   AALQALKAFPLGSFNSSHRLQFESIFYQRKRRFDSSARTLALD-------------------------------------
```

```
DmApoLppI   1754   ---------------------------------------------------------------------AKEAGPDFSKL
DmApoLppII   413   --------------------------------------------------------------------------------
DmApoLTPI   2368   SYYKTESTAHYKRDIFPARYGKFLALLDVNKRELEYERQSPFHAVRLHLLPTIRYPIYGLNATIWDTPDTNHSGYIYMDI
DmApoLTPII   464   --------------------------------------------------------------------------------
DmCG31659    109   --------------------------------------------------------------------------------
DmCvD        596   --------------------------------------------------------------------------------
DmFabp        98   --------------------------------------------------------------------------------
DmGlaz       115   --------------------------------------------------------------------------------
DmMic26-27   140   --------------------------------------------------------------------------------
DmNlaz       108   --------------------------------------------------------------------------------
DmMtp        530   --------------------------------------------------------------------------------

DmApoLppI   1765   QGKLLDYKNDIVQELEADQSIAPIIDGIRTLFKKIAGIVDD---------------------------INKAISEILEKA
DmApoLppII   413   -------------------------------------------------------------------------------A
DmApoLTPI   2448   LERYARMDFNLTEDASQNLQMVGYIPDTRSAFLDIWRNYEEIRVIDVSSYLKMNHSRLITGRFHWRPSIRQEVREKIQAV
DmApoLTPII   464   -------------------------------------------------------------------------------A
DmCG31659    109   --------------------------------------------------------------------------------
DmCvD        596   -------------------------------------------------------------------------------I
DmFabp        98   -------------------------------------------------------------------------------K
DmGlaz       115   --------------------------------------------------------------------------------
DmMic26-27   140   -------------------------------------------------------------------------------I
DmNlaz       108   -------------------------------------------------------------------------------L
DmMtp        530   -------------------------------------------------------------------------------I

DmApoLppI   1818   QKSIVDIYDKL--QALWKDSLLKAWE--DFIITVQKLISTLKTEFIK---------------------------------
DmApoLppII   414   PKELYIAVGNL--VAKYCLKNYCQGP---------EIDAISKKFSDGLKHCKPNTKREE---------------------
DmApoLTPI   2528   GKSVYSSFSEG--IDFWIKSIYTETT--ESMGVVWNTAKEYNRDFIDDIGQLSVLEEDLADLRLFVNQSYEANDFYIKNV
DmApoLTPII   465   KNRLDAEYTLG--ATAVVHSFCKHHEACEENLRVQQIINLLETEFLNLYNLFKGERRTR---------------------
DmCG31659    109   --TTSTAFPEG--VLMYV----------------------LDTDYV----------------------------------
DmCvD        597   SETTYPCYQHLRRLLSYMHRHLPQKPESRYWVTGNYIFDYRDSKFGIGAMLQVFLVGDPKSDMPVVAFFKFDTEALGKFT
DmFabp        99   PTTIVREFTDNELITLIPHLTPSQHG------------------------------------------------------
DmGlaz       115   --SSIMDFKFTTRFPDVIARLLPGSG----------KYQVLYTDYE----------------------------------
DmMic26-27   141   KKVLYSGIGAG----AVASMCYPRQA------------------------------------------------------
DmNlaz       109   AVAFYPTQPLT--KANYLV---------------------LGTDYE----------------------------------
DmMtp        531   ILSLRPTQEQLGNFLDYLASNDRQFEIKTYVLQKLRMLAEKCPRFRALFKSELVKRRHVN--------------------

DmApoLppI   1861   ------------------ICTQSFKDLLSALEKYGPALKNYGKAIGEIVKPINDAA------------------------
DmApoLppII   462   --------------------------------------------------------------------------------
DmApoLTPI   2604   VNFTLTILDELAIRDHIESLPKIFSELWQAMGDSGKALRNSIVWLIETIKTTYNNLLDAVARFFHGESLVYISGLLEKGI
DmApoLTPII   522   --------------------------------------------------------------------------------
DmCG31659    129   --------------------------------------------------------------------------------
DmCvD        677   GQLALYIKARGLPDTILNKMQSRNGSDPFTFKSIKALLAMLQAPIINSK-------------------------------
DmFabp       125   --------------------------------------------------------------------------------
DmGlaz       149   --------------------------------------------------------------------------------
DmMic26-27   163   --------------------------------------------------------------------------------
DmNlaz       132   --------------------------------------------------------------------------------
DmMtp        591   --------------------------------------------------------------------------------

DmApoLppI   1899   --------QEVIKIVVN---------------------------------------------------------------
DmApoLppII   462   --------ERIVYILKGLG-------------------------------------------------------------
DmApoLTPI   2684   AKYDSFIKDLHIKFIKYIENLWHKTWTLAENHWKAVLKRFEPHLFKMISFIETTAWNLSKEVFDFIYKRTNELAESPYFN
DmApoLTPII   522   --------ERMVILLKGLG-------------------------------------------------------------
DmCG31659    129   --------NFAIRFMCF---------------------------------------------------------------
DmCvD        726   --------DLHLEFILQMEGKTVLSYYLNQRMFRQL--------------------------------------------
DmFabp       125   --------SLRLRVPC----------------------------------------------------------------
DmGlaz       149   --------NFAILWSCG---------------------------------------------------------------
DmMic26-27   163   --------EENCRVVLY---------------------------------------------------------------
DmNlaz       132   --------SYAVVYSCT---------------------------------------------------------------
DmMtp        591   --------NYNVLGQKG---------------------------------------------------------------

DmApoLppI   1908   -------AAEGVTHEFK--QYVASLPSFESIRNEFNDKVKVLKLFEKATELTNSLFDQINILPQTPETSEFLQKLHDYLI
DmApoLppII   473   -------NAKSLSGNTV------------AALSECASTGRSNRIRVAALHAFSKV--------------KCEETLQSKSL
DmApoLTPI   2764   KVSSFTADAERLYRDFKANDAITNIKKYSTIAWNFVKE-KYFKLVPFGAELNEVLTEIWQEIKELEKIDQVQIMVQKYYE
DmApoLTPII   533   -------NIGVVSSAFAE-----------QLQWIIREDEAPVDIRLHGILAFRRV---------------DCARHRSYFL
DmCG31659    138   -------DASKIF----------------SFHWAVIQTR-----------------------------------------
DmCvD        754   -------TYDNILERMQQIIRTDSHINMQTVRWPFMNRYTVPTVLGTSSDVLLQTTVLTSLRGNITEQRNSPITKHTLEI
DmFabp       133   --------------------------------------------------------------------------------
DmGlaz       158   -------SIGSLGHS--------------DQIWILGR-------------------------------------------
DmMic26-27   172   -------EGRKIF----------------AVAYNFIKGV-----------------------------------------
DmNlaz       141   -------SVTPLANF--------------KIVWILTR-------------------------------------------
DmMtp        600   -------LTTVLTRQLSQAPAFNETLLSTQEVYQGILKRGSVEFLLHAGRSQASSFKLGIYTAGLGSLVGDGDSGDGNDA
```

```
DmApoLppI   1979   AKLKQEHIDNEKYIEELGQLLIKAVRSIWVSI------RSTYPGSSDHVIDFQSWIGSLTHSF-----------------
DmApoLppII   520   ELLKNRNEDSELRIEAYLSAISCPNAEVANQI------SEIVNSETVNQVGGFISSNLKA--------------------
DmApoLTPI   2843   VMAKVDWVADELQLEHR-------LHQVYGLV------RNKFRNYAMNALETADMYREAKTKFVFDPEVGIIDLEQKLPM
DmApoLTPII   580   DNYGNYTLNSELRIYSYLQAMRCPDYISVGVI------KSILEHEEINQVGSFVWSHLTN--------------------
DmCG31659    154   --------------------------------------------------------------------------------
DmCvD        827   DARYSSYASVRSRSYNPFLNLDHEINREQGFLIYIPFSSELHLNESGSKCRRYSFSRPQNLTSGLSFKSRAVTKTRGLIT
DmFabp       133   --------------------------------------------------------------------------------
DmGlaz       174   --------------------------------------------------------------------------------
DmMic26-27   188   --------------------------------------------------------------------------------
DmNlaz       157   --------------------------------------------------------------------------------
DmMtp        673   IPADDEFSEDEAVTAGMEISVQGAQLRPLVFF------------SGQTELMGHVWGGSAS--------------------

DmApoLppI   2036   -----------DSLAVLPSILSFR----SSILNCLLNENWDVVFNKKLLYSWIFFNDFELRGHVVDGKHIFTFDGLNFAY
DmApoLppII   574   -----------IRDSTDVSRDQQK----YHLANIRVTKTFPV--------------------------------------
DmApoLTPI   2910   SWHAFNETPRFEEIPEYQVLAKAQ----SFFSETNSSIVMKLYNMRTHLDPKTWLPPYYSRALLIDSRHYMTFDQRYVGL
DmApoLTPII   634   -----------LAKSNSPVRIEAQ----GLLLNDELSERFKM--------------------------------------
DmCG31659    154   -----------KRLPSTQVIHMAQ--------------------------------------------------------
DmCvD        907   KTAAAPFEEIMVPEGRNDVVQLFS----YPMTDLGVRLSMTTNLNELIKYRGMLLKSEFTENGFSGNMVVNALMYIFGFT
DmFabp       133   -----------QPVPPTSVDDGAR--------------------------------------------------------
DmGlaz       174   -----------DRDFEVDIRSKVY--------------------------------------------------------
DmMic26-27   188   -----------KPGEDVPVVPFPT--------------------------------------------------------
DmNlaz       157   -----------QREPSAEAVDAAR--------------------------------------------------------
DmMtp        721   -----------DSTPAYQATTLSQDNEHYIILTSGATLHWRVLGARSVDLNGKVGFSLWNRNAQTE--------------

DmApoLppI   2101   ------------PGNCKYILAQDSVDNNFTIIGQLTNGKLKSITLIDREGSY-------FEVADNLALKLNGNLVEYPQH
DmApoLppII   601   --------------------------------------------------------------------------------
DmApoLTPI   2986   NLNFDELGNGRSTSQCSYLLAHDFFKRNFTLLLEPASKSLAGQGLTRKLSFIANGQLIEIDLETDHISINGNPQPILPLK
DmApoLTPII   661   --------------------------------------------------------------------------------
DmCG31659    167   --------------------------------------------------------------------------------
DmCvD        983   QLSSIHLGHDRNFTMLMYNEKNTRIEGNFCAEDVLKTSD-----------------------------------------
DmFabp       146   --------------------------------------------------------------------------------
DmGlaz       187   --------------------------------------------------------------------------------
DmMic26-27   201   --------------------------------------------------------------------------------
DmNlaz       170   --------------------------------------------------------------------------------
DmMtp        776   --------------------------------------------------------------------------------

DmApoLppI   2162   LSGLHAWRRFYTIHLYSEYGVGIVCTSDLKVCHININGFYTSKTRGL-LGNGNAEPYDDFLLIDGTLAENSAALGNDYGV
DmApoLppII   601   ----------------------------------DYRRYSFNNEVSYKLESLGVGA------------------------
DmApoLTPI   3066   LGDVNIHRDLDVLSITSDTEFSLHCNVQFDLCWFEVSGWYFGRTAGL-LGTLNNEPYDEYTMSSGVISNETQLFTDSWSL
DmApoLTPII   661   ----------------------------------DIRKFSRNYEHSLFFDEYNFGT------------------------
DmCG31659    167   ---------------------------------------YFGKSAGLVIGDMSKVP------------------------
DmCvD       1022   ---------------------------------------MKGKQIGLTLEHTDHMNENHAADALHRWNITLDVLASTKSN
DmFabp       146   --------------------------------------------------------------------------------
DmGlaz       187   ----------------------------------------------DVLKRLSLDP------------------------
DmMic26-27   201   -----------------------------------------------SLEDLKYMA------------------------
DmNlaz       170   ----------------------------------------------KILEDNDVSQ------------------------
DmMtp        776   ---------------------------------------IQQNTGSAVLGHLAVGFTYAKLVQDFSITHEPKLSLNA---

DmApoLppI   2241   GKCTAIEFDNNQFKSSKRQEMC-------------SELFGIESTLAFNFITLDSRPYRKACDIALAKVAEKEKEAT---A
DmApoLppII   623   --------STDYQIIYSQHGFL-------------PRSSRINVT------------------------------------
DmApoLTPI   3145   KQCRQNKLAQTQEVSQEVSDAC-------------TSFFRTGILATCSAVLDPTPFYEMCMDLGMKSPPIRKGHPAVKGA
DmApoLTPII   683   --------TTDANVIFGTDSYL-------------PRIASVNFT------------------------------------
DmCG31659    184   ------------------QESC-------------PYDT-----------------------------------------
DmCvD       1063   WFKLTGQVQRNSKDDEDDWKACTKLTYEPLVFTKRPHTLNGDVVFGLATEESECPEKGSTVQFAARAGPSEHARAFLRSD
DmFabp       146   ------------------SGGC----------GQRPGGPG----------------------------------------
DmGlaz       197   -----------ERLIISKNKQC-------------PEAL-----------------------------------------
DmMic26-27   210   -----------SDLYDEAKDLI------------FPKKK-----------------------------------------
DmNlaz       180   -----------AFLIDTVQKNC-------------PRLDGNGTGL-----------------------------------
DmMtp        814   -----------DLDFYSGIKLC--------MQLQRPEQLLKQTNV-----------------------------------

DmApoLppI   2305   CTFALAYGSAVKQINKWVLLPPRCIKCAGPAGQHDFGDEFTVKLPNNKVDVVFVVDINVTPGVLSNL-----IAPAINDI
DmApoLppII   646   --------------------------------------------------------------------------------
DmApoLTPI   3212   CAAALAYIEACTALKVPMRVPSQCVFCQLSNGSYVPEGTFMELSGPEIPKSSDVVFIVEAKECNANLKTSKNIMTVVSSI
DmApoLTPII   706   --------------------------------------------------------------------------------
DmCG31659    192   --------------------------------------------------------------------------------
DmCvD       1143   KISLTDTDFCPKEVLKFSPIPTSRYCKRSNFENFTSITQYDMDLKFDNMPAWFELWSNRLDHLVSALSADKVDSLHMSQE
DmFabp       158   --------------------------------------------------------------------------------
DmGlaz       212   --------------------------------------------------------------------------------
DmMic26-27   226   --------------------------------------------------------------------------------
DmNlaz       201   --------------------------------------------------------------------------------
DmMtp        840   --------------------------------------------------------------------------------
```

```
DmApoLppI   2380   RESLRSRGFSDVQVGVIVFEETKRYPALLTSDGGKINYKGNVADVKLAGIKSFCDNCVEQIITEKRILDIYNSLKEIVKG
DmApoLppII   646   ------------------------------TEFFGTNYNVFEASVRQENVEDVLEYYLGPKGLVNKDFDEI---------
DmApoLTPI   3292   EEQLQAAKITNNRYAVVAFGGVSPY-----DKARSVIYEHNEFTSKPEQLADYFGHINTGNGSSNDILMAISAAAKLNFR
DmApoLTPII   706   ------------------------------ADLFGQSVNFFEFTARAEGLEELAANAFGPKGPLSGQLL-----------
DmCG31659    192   --------------------------------------------------------------------------------
DmCvD       1223   INISMQTPQDQFRLAVEVNGVKWRFHQIPFFYKLDSKFDASHELTFDSGLKRSCSVINGIVNTFDDYLINLRE-------
DmFabp       158   --------------------------------------------------------------------------------
DmGlaz       212   --------------------------------------------------------------------------------
DmMic26-27   226   --------------------------------------------------------------------------------
DmNlaz       201   ------------------------------AGEDGLDVDDFVSTTVPNAIEKA?EWLRRLYERLYDIFMNFLSY------
DmMtp        840   ------------------------------RSVFLQSVDRPYAKHVRSTLSHKTAGCTFALNQKNNEMCNLIFRDL----

DmApoLppI   2460   IAPQADEKAFQLALDYPFRAGAAKSIIGVRSDSLEYKNWWKFVRAQLTGSITKFDGALIHLIAPVKGLSLEGVLSEKLIG
DmApoLppII   687   --------------------------------------------------------------------------------
DmApoLTPI   3367   ----------------PGVSKTFILLSCSKCAARDMRFDYTSILQYLLEEGVNLHILADTEFDFERNKKLRHFFGLDSKL
DmApoLTPII   745   --------------------------------------------------------------------------------
DmCG31659    192   --------------------------------------------------------------------------------
DmCvD       1296   ------------------IAVRPDCLTLLVADCSPLPQIAVFVTPSPVQGLSTNYGLRVHIGQNYFNFRARTDNSSLPTD
DmFabp       158   --------------------------------------------------------------------------------
DmGlaz       212   --------------------------------------------------------------------------------
DmMic26-27   226   --------------------------------------------------------------------------------
DmNlaz       245   --------------------------------------------------------------------------------
DmMtp        886   --------------------------------------------------------------------------------

DmApoLppI   2540   FNSRLVATVDGKDSKKRTKLQFDNDMGIDFVLNNGGWVFATQNFEKLKASDQKKMLNQITSSLADTLFKTEIVSDCRCLP
DmApoLppII   687   ---------------------VKLIEVGNNGVAAGGRARR----------------------------------------
DmApoLTPI   3431   VYSKRFPEGDAETRNTTHIPKSNLGICTTLAVETQGSVFSARKLQPERKYPIKRFATIFAKRVALSATPIQSQTCECSAH
DmApoLTPII   745   ----------------RKKLSFLNRWLGNESAEEDDTLENLLSLDNLRLK------------------------------
DmCG31659    192   --------------------------------------------------------------------------------
DmCvD       1358   EPVLIYLNQDQTPHNVRKKPYQWPIETSDYDFRVELNEQNILIVECTQLSSTIQFDLYNILNFEIYGVYKHQMCGLCSKP
DmFabp       158   --------------------------------------------------------------------------------
DmGlaz       212   --------------------------------------------------------------------------------
DmMic26-27   226   --------------------------------------------------------------------------------
DmNlaz       245   --------------------------------------------------------------------------------
DmMtp        886   --------------------------------------------------------------------------------

DmApoLppI   2620   IHGLHGQHKCVIKSSTFVANKKAKSA------------------
DmApoLppII   706   --------------------------------------------
DmApoLTPI   3511   NTGVSYMACSPQALPEEKYDLDDYDSFNNWDWGDEPESETNVMS
DmApoLTPII   779   --------------------------------------------
DmCG31659    192   --------------------------------------------
DmCvD       1438   LNRMQNYTICELEANTPTPVPLQNSSDVVVVA------------
DmFabp       158   --------------------------------------------
DmGlaz       212   --------------------------------------------
DmMic26-27   226   --------------------------------------------
DmNlaz       245   --------------------------------------------
DmMtp        886   --------------------------------------------
```

Fig. S10. Alignment of 25 human and 11 fly sequences with ApoB, ApoLpp and ApoLTP separated plus both outgroups.

```
DmApoLppI         1  -------------------------------------------------------------------------------S
DmApoLppII        0  --------------------------------------------------------------------------------
DmApoLTPI         1  RKAQEQARASEEEVDEDYDFEESLEGNKRQKRDVSTTRKQEIDRNVDSLGYKLKYDYNNPRAQFGLRVFGNDLRYFNVES
DmApoLTPII        0  --------------------------------------------------------------------------------
DmCG31659         0  --------------------------------------------------------------------------------
DmCvD             0  --------------------------------------------------------------------------------
DmFabp            0  --------------------------------------------------------------------------------
DmGlaz            0  --------------------------------------------------------------------------------
DmMic26-27        0  --------------------------------------------------------------------------------
DmNlaz            0  --------------------------------------------------------------------------------
HsApoA1           0  --------------------------------------------------------------------------------
HsApoA2           0  --------------------------------------------------------------------------------
HsApoA4           0  --------------------------------------------------------------------------------
HsApoA5           0  --------------------------------------------------------------------------------
HsApoB48          0  --------------------------------------------------------------------------------
HsApoB52          1  -----------------------------------------------------------FDQYIKDSYDLHDLKIAIANI
HsApoC1           0  --------------------------------------------------------------------------------
HsApoC2           0  --------------------------------------------------------------------------------
HsApoC3           0  --------------------------------------------------------------------------------
HsApoC4           0  --------------------------------------------------------------------------------
HsApoD            0  --------------------------------------------------------------------------------
HsApoE2           0  --------------------------------------------------------------------------------
HsApoF            0  --------------------------------------------------------------------------------
HsApoH            0  --------------------------------------------------------------------------------
HsApoJ            0  --------------------------------------------------------------------------------
HsApoL1           0  --------------------------------------------------------------------------------
HsApoL2           0  --------------------------------------------------------------------------------
HsApoL3           0  --------------------------------------------------------------------------------
HsApoL4           0  --------------------------------------------------------------------------------
HsApoL5           0  --------------------------------------------------------------------------------
HsApoL6           0  --------------------------------------------------------------------------------
HsApoM            0  --------------------------------------------------------------------------------
HsApoO            0  --------------------------------------------------------------------------------
HsApoOL           0  --------------------------------------------------------------------------------
HsMTTP            0  --------------------------------------------------------------------------------
DmMtp             0  --------------------------------------------------------------------------------

DmApoLppI         2  IVDDVSKISKKYKMYGVKNVQDLNLDVSLKLFGSELAFLSLGDNIPSSLDDIINYFSTSFEKAKQELSSFEKQFSSHHLF
DmApoLppII        1  -MARMKYNIALIGILASVLLTIAVNAENACNLGCPKSDNGLLKYIPGNYYDYSFDSILTIGASSDVPNDSDDTSLKVSGS
DmApoLTPI        81  LVEVMALAAKFNPFQQAKNVLSGKEFTYTKSRVFLDASYTVPLAVGLPLAIHAFGASSIDLRVSGNLDEMDPPTDWHFDV
DmApoLTPII        1  ------MGGLKPQAAIWLLLLIAHTQAVRENPLKDPRICGRPQCDSKSAKFNYGEQLYKYQYTVAVRTEFAGSGDNSSDL
DmCG31659         0  --------------------------------------------------------------------------------
DmCvD             1  ------------------MRLLKCSVFVYLFLIFDAGHAFSIIGLNKQMLYEYEGNVLVGAKPQDEGHQAPPTTGWIVRG
DmFabp            1  ---------------------------------------------------------------------------MSFVG
DmGlaz            1  -------------------------------------------------------------------------MMSGQPL
DmMic26-27        1  --------------------------------------------------------------------MLRKTATMGIMA
DmNlaz            0  --------------------------------------------------------------------------------
HsApoA1           0  --------------------------------------------------------------------------------
HsApoA2           0  --------------------------------------------------------------------------------
HsApoA4           1  -----------------------------------------------------MFLKAVVLTLALVAVAGARAEVSADQV
HsApoA5           1  -----------------------------------------------------MASMAAVLTWALALLSAFSATQARKGF
HsApoB48          1  ------MDPPRPALLALLALPALLLLLLAGARAEEEMLENVSLVCPKDATRFKHLRKYTYNYEAESSSGVPGTADSRSAT
HsApoB52         22  IDEIIEKLKSLDEHYHIRVNLVKTIHDLHLFIENIDFNKSGSSTASWIQNVDTKYQIRIQIQEKLQQLKRHIQNIDIQHL
HsApoC1           0  --------------------------------------------------------------------------------
HsApoC2           0  --------------------------------------------------------------------------------
HsApoC3           0  --------------------------------------------------------------------------------
HsApoC4           1  -----------------------------------------------------------------------------MSL
HsApoD            1  -----------------------------------MVMLLLLLSALAGLFGAAEGQAFHLGKCPNPPVQENFDV------
HsApoE2           1  ---------------------------------MKVLWAALLVTFLAGCQAKVEQAVETEPEPELRQQTEWQSGQRWELA
HsApoF            1  ------------------------------------------------------------------MTGLCGYSAPDMRG
HsApoH            1  -----------------------MISPVLILFSSFLCHVAIAGRTCPKPDDLPFSTVVPLKTFYEPGEEITYSCKPGYVS
HsApoJ            1  --------------------------------MMKTLLLFVGLLLTWESGQVLGDQTVSDNELQEMSNQGSKYVNKEIQN
HsApoL1           1  ---------MEGAALLRVSVLCIWMSALFLGVGVRAEEAGARVQQNVPSGTDTGDPQSKPLGDWAAGTMDPESSIFIEDA
HsApoL2           1  --------------------------------------------------------------------MNPESSIFIEDY
HsApoL3           1  --------------------------------------------------------------------MDSEKKRFTEEA
HsApoL4           1  --------------------------------------------MGSWVQLITSVGVQQNHPGWTVAGQFQEKKRFTEEV
HsApoL5           1  ----------------------------------------------MPCGKQGNLQVPGSKVLPGLGEGCKEMWLRKVIY
HsApoL6           1  --------------------------------------------------------------------MDNQAERESEAG
HsApoM            0  --------------------------------------------------------------------------------
HsApoO            1  -------------------------------------------------------------------------------M
HsApoOL           1  -----------------------------------------------------------------------------MAA
HsMTTP            1  ----------------MILLAVLFLCFISSYSASVKGHTTGLSLNNDRLYKLTYSTEVLLDRGKGKLQDSVGYRISSNVD
DmMtp             1  -------------MENKNKKCLRTLLLLALFLGLLEDGKTALIAPNSQQIFKLQNQVILQELGRDSSSAETSYTFETDLK
```

```
DmApoLppI       82   LDTDLAYP--TSIGVPLELVAQGFAA------------------------------------------------------
DmApoLppII      80   AKIFAKGN--CGYTLQLSSVKVTNTK------------------------------------------------------
DmApoLTPI      161   EGQFKPSV---SVDVITTMQTDMFWEQSGIKVKSNLYSNSELVAKLKVRGRNLVSFSFDLPRDKNEIFSVRSELLVQKRE
DmApoLTPII      75   LLKSDLEI--FFPKPCEGYLRINDAK------------------------------------------------------
DmCG31659        0   --MIKWKI--FVPAILYLQSSMAMRA------------------------------------------------------
DmCvD           63   KLTLQRQS--ELVLAAALVIDDVTLN------------------------------------------------------
DmFabp           6   KKYKLDKS--ENFDEYMKELGVGLVT------------------------------------------------------
DmGlaz           8   GSRVWLLS--GVLLVTFAGTDAYGFG------------------------------------------------------
DmMic26-27      13   AVAVKAAP--EPQKPASSAADCSLVC------------------------------------------------------
DmNlaz           0   --MNHHSS--SHLLLLISVVFGAVWV------------------------------------------------------
HsApoA1          0   ---MSKDL--EEVKAKVQPYLDDFQK------------------------------------------------------
HsApoA2          0   MKLLAATV--LLLTIC----------------------------------------------------------------
HsApoA4         28   ATVMWDYF--SQLSNNAKEAVEHLQK------------------------------------------------------
HsApoA5         28   WDYFSQTS--GDKGRVEQIHQQKMAR------------------------------------------------------
HsApoB48        75   RINCKVEL--EVPQLCSFILKTSQCT------------------------------------------------------
HsApoB52       102   AGKLKQHI--EAIDVRVLLDQLGTTI------------------------------------------------------
HsApoC1          0   -MRLFLSL--PVLVVVLSIVLEGPAP------------------------------------------------------
HsApoC2          0   ---MGTRL--LPALFLVLLVLGFEVQ------------------------------------------------------
HsApoC3          0   -----MQP--RVLLVVALLALLASAR------------------------------------------------------
HsApoC4          4   LRNRLQAL--PALCLCVLVLACIGAC------------------------------------------------------
HsApoD          40   NKYLGRWY--EIEKIPTTFENGRCIQ------------------------------------------------------
HsApoE2         48   LGRFWDYL--RWVQTLSEQVQEELLS------------------------------------------------------
HsApoF          15   LRLIMIPV--ELLLCYLLLHPVDATS------------------------------------------------------
HsApoH          58   RGGMRKFI--CPLTGLWPINTLKCTP------------------------------------------------------
HsApoJ          49   AVNGVKQI--KTLIEKTNEERKTLLS------------------------------------------------------
HsApoL1         72   IKYFKEKV--STQNLLLLLTDNEAWN------------------------------------------------------
HsApoL2         13   LKYFQDQV--SRENLLQLLTDDEAWN------------------------------------------------------
HsApoL3         13   TKYFRERV--SPVHLQILLTNNEAWK------------------------------------------------------
HsApoL4         37   IEYFQKKV--SPVHLKILLTSDEAWK------------------------------------------------------
HsApoL5         35   GGEVWGKS--PEPEFPSLVNLCQSWK------------------------------------------------------
HsApoL6         13   VGLQRDED--DAPLCEDVELQDGDLS------------------------------------------------------
HsApoM           0   --MFHQIW--AALLYFYGIILNSIYQ------------------------------------------------------
HsApoO           2   FKVIQRSV--GPASLSLLTFKVYAAP------------------------------------------------------
HsApoOL          4   IRMGKLTT--MPAGLIYASVSVHAAK------------------------------------------------------
HsMTTP          65   VALLWRNP--DGDDDQLIQITMKDVN------------------------------------------------------
DmMtp           68   INSVWSGDEDQLLEVFISGSKVDASG------------------------------------------------------

DmApoLppI      106   ---------------------------------------------------------------------------TKVDL
DmApoLppII     104   ---------------------------------------------------------------------------ESVEK
DmApoLTPI      238   EQLPQAGIANRSANSTCTWPVLDQAIGLQMCSHYSVPDLSNATEIYPSLLLAGPLNFSLILKKSDLSAKKYVFEYKWDQQ
DmApoLTPII      99   ---------------------------------------------------------------------------LYDTL
DmCG31659       22   ---------------------------------------------------------------------------FHGAC
DmCvD           87   ---------------------------------------------------------------------------NSGEK
DmFabp          30   ---------------------------------------------------------------------------RKMGN
DmGlaz          32   ---------------------------------------------------------------------------RCPNY
DmMic26-27      37   ---------------------------------------------------------------------------RPSEL
DmNlaz          22   ---------------------------------------------------------------------------AHAQV
HsApoA1         21   ---------------------------------------------------------------------------KWQEE
HsApoA2         14   --------------------------------------------------------------------------------
HsApoA4         52   ---------------------------------------------------------------------------SELTQ
HsApoA5         52   ---------------------------------------------------------------------------EPATL
HsApoB48        99   ---------------------------------------------------------------------------LKEVY
HsApoB52       126   ---------------------------------------------------------------------------SFERI
HsApoC1         23   ---------------------------------------------------------------------------AQGTP
HsApoC2         21   ---------------------------------------------------------------------------GTQQ-
HsApoC3         19   ---------------------------------------------------------------------------ASEAE
HsApoC4         28   ---------------------------------------------------------------------------QPEAQ
HsApoD          64   ---------------------------------------------------------------------------ANYSL
HsApoE2         72   ---------------------------------------------------------------------------SQVTQ
HsApoF          39   ---------------------------------------------------------------------------YGKQT
HsApoH          82   ---------------------------------------------------------------------------RVCPF
HsApoJ          73   ---------------------------------------------------------------------------NLEEA
HsApoL1         96   ---------------------------------------------------------------------------GFVAA
HsApoL2         37   ---------------------------------------------------------------------------GFVAA
HsApoL3         37   ---------------------------------------------------------------------------RFVTA
HsApoL4         61   ---------------------------------------------------------------------------RFVRV
HsApoL5         59   ---------------------------------------------------------------------------INNLM
HsApoL6         37   ---------------------------------------------------------------------------PEEKI
HsApoM          22   ---------------------------------------------------------------------------CPEHS
HsApoO          26   ---------------------------------------------------------------------------KKDSP
HsApoOL         28   ---------------------------------------------------------------------------QEESK
HsMTTP          89   ---------------------------------------------------------------------------VENVN
DmMtp           94   ---------------------------------------------------------------------------KARSI
```

```
DmApoLppI        111   AVSLDINAILEQNWQKAKYRLKFVPSVDINANVQIGFNAQVLSTGLRVVSSAHSATGSDITVAVISDGEGFNVDLELPRE
DmApoLppII       109   KILNS---------------------------------------------------------------------------
DmApoLTPI        318   EEDNNFSLVFTTPGSKVPRVLVANVTKVPDAFNASVAFVNGPNRVSAGCSYDGNPDFRRLDIYLDTNGNRSLDLGMELRR
DmApoLTPII       104   DELND---------------------------------------------------------------------------
DmCG31659         27   PSNMT---------------------------------------------------------------------------
DmCvD             92   FLQNK---------------------------------------------------------------------------
DmFabp            35   SLSPT---------------------------------------------------------------------------
DmGlaz            37   PSMPK---------------------------------------------------------------------------
DmMic26-27        42   PIYGS---------------------------------------------------------------------------
DmNlaz            27   PFPGK---------------------------------------------------------------------------
HsApoA1           26   MELYR---------------------------------------------------------------------------
HsApoA2           14   --------------------------------------------------------------------------------
HsApoA4           57   QLNAL---------------------------------------------------------------------------
HsApoA5           57   KDSLE---------------------------------------------------------------------------
HsApoB48         104   GFNPE--GKALLKKTKNSEEFAAAMSRYELKLAIPEGKQVFLYPEKDEPTYILNIKRGIISALLVPPETEEAKQVLFLDT
HsApoB52         131   NDILEHVKHFVINLIGDFEVAEKINAFRAKVHELIERYEVDQQIQVLMDKLVELAHQYKLKETIQKLSNVLQQVKIKDYF
HsApoC1           28   DVSSA---------------------------------------------------------------------------
HsApoC2           25   --------------------------------------------------------------------------------
HsApoC3           24   DASLL---------------------------------------------------------------------------
HsApoC4           33   EGTLS---------------------------------------------------------------------------
HsApoD            69   MENGK---------------------------------------------------------------------------
HsApoE2           77   ELRAL---------------------------------------------------------------------------
HsApoF            44   NVLMH---------------------------------------------------------------------------
HsApoH            87   AGILE---------------------------------------------------------------------------
HsApoJ            78   KKKKE---------------------------------------------------------------------------
HsApoL1          101   AELPR---------------------------------------------------------------------------
HsApoL2           42   AELPR---------------------------------------------------------------------------
HsApoL3           42   AELPR---------------------------------------------------------------------------
HsApoL4           66   AELPR---------------------------------------------------------------------------
HsApoL5           64   STVHS---------------------------------------------------------------------------
HsApoL6           42   --------------------------------------------------------------------------------
HsApoM            27   QLTTL---------------------------------------------------------------------------
HsApoO            31   PKNSV---------------------------------------------------------------------------
HsApoOL           33   KQLVK---------------------------------------------------------------------------
HsMTTP            94   QQRGE---------------------------------------------------------------------------
DmMtp             99   TRIPD---------------------------------------------------------------------------

DmApoLppI        191   KLELINFNVDTELYVAEQDKQKAIALKGNKKNKNSQPSEICFNQLELVGLNICIKSSTSLSEVQAGNGNVAERGLSVSEK
DmApoLppII       114   --------------------------------------------------------------------------------
DmApoLTPI        398   HQDFTAWIYNPRMLLAINGVNITGLAGTVKVNEKNGIKQHDVDLSFETKKLQAVIKGNVVQSEITTSTNMTIKYRFQANK
DmApoLTPII       109   --------------------------------------------------------------------------------
DmCG31659         32   --------------------------------------------------------------------------------
DmCvD             97   -----------------------EMYPPYKPFKIALTKDGAISHVVFKEGDPIWSMNFKRAIASVLQFQMKSSGAFVVDE
DmFabp            40   --------------------------------------------------------------------------------
DmGlaz            42   --------------------------------------------------------------------------------
DmMic26-27        47   --------------------------------------------------------------------------------
DmNlaz            32   --------------------------------------------------------------------------------
HsApoA1           31   --------------------------------------------------------------------------------
HsApoA2           14   --------------------------------------------------------------------------------
HsApoA4           62   --------------------------------------------------------------------------------
HsApoA5           62   --------------------------------------------------------------------------------
HsApoB48         182   VYGNCSTHFTVKTRKGNVATEISTERDLGQCDRFKPIRTGISPLALIKGMTRPLSTLISSSQSCQYTLDAKRKHVAEAIC
HsApoB52         211   EKLVGFIDDAVKKLNELSFKTFIEDVNKFLDMLIKKLKSFDYHQFVDETNDKIREVTQRLNGEIQALELPQKAEALKLFL
HsApoC1           33   --------------------------------------------------------------------------------
HsApoC2           25   --------------------------------------------------------------------------------
HsApoC3           29   --------------------------------------------------------------------------------
HsApoC4           38   --------------------------------------------------------------------------------
HsApoD            74   --------------------------------------------------------------------------------
HsApoE2           82   --------------------------------------------------------------------------------
HsApoF            49   --------------------------------------------------------------------------------
HsApoH            92   --------------------------------------------------------------------------------
HsApoJ            83   --------------------------------------------------------------------------------
HsApoL1          106   --------------------------------------------------------------------------------
HsApoL2           47   --------------------------------------------------------------------------------
HsApoL3           47   --------------------------------------------------------------------------------
HsApoL4           71   --------------------------------------------------------------------------------
HsApoL5           69   --------------------------------------------------------------------------------
HsApoL6           42   --------------------------------------------------------------------------------
HsApoM            32   --------------------------------------------------------------------------------
HsApoO            36   --------------------------------------------------------------------------------
HsApoOL           38   --------------------------------------------------------------------------------
HsMTTP            99   --------------------------------------------------------------------------------
DmMtp            104   --------------------------------------------------------------------------------
```

```
DmApoLppI        271   FHLSRPFNFAVYLTTERKFTFKGIHTQEAFSQKWKLDYSTPGSKVSHDTTVVYELGNKPKTFSRLSFDNSQCHFAVEGGI
DmApoLppII       114   --------------------------------------------------------------------------------
DmApoLTPI        478   IEEINFAGKLVNNGDKSKTEYRGNMKLQTSAYPKLNFASESTWLSLQGHTEGMITYNNAPDYVNPNYTSLVRLIFARSHS
DmApoLTPII       109   --------------------------------------------------------------------------------
DmCG31659         32   --------------------------------------------------------------------------------
DmCvD            154   LGIHGTCRTEYFVSNRTNYISIRKTPEVKTCKPYSEAVHTTRSNVPPNTCEFDHQKSVIIGNEAIYGMSPHNETGYYLSM
DmFabp            40   --------------------------------------------------------------------------------
DmGlaz            42   --------------------------------------------------------------------------------
DmMic26-27        47   --------------------------------------------------------------------------------
DmNlaz            32   --------------------------------------------------------------------------------
HsApoA1           31   --------------------------------------------------------------------------------
HsApoA2           14   --------------------------------------------------------------------------------
HsApoA4           62   --------------------------------------------------------------------------------
HsApoA5           62   --------------------------------------------------------------------------------
HsApoB48         262   KEQHLFLPFSYKNKYGMVAQVTQTLKLEDTPKINSRFFGEGTKKMGLAFESTKSTSPPKQAEAVLKTLQELKKLTISEQN
HsApoB52         291   EETKATVAVYLESLQDTKITLIINWLQEALSSASLAHMKAKFRETLEDTRDRMYQMDIQQELQRYLSLVGQVYSTLVTYI
HsApoC1           33   --------------------------------------------------------------------------------
HsApoC2           25   --------------------------------------------------------------------------------
HsApoC3           29   --------------------------------------------------------------------------------
HsApoC4           38   --------------------------------------------------------------------------------
HsApoD            74   --------------------------------------------------------------------------------
HsApoE2           82   --------------------------------------------------------------------------------
HsApoF            49   --------------------------------------------------------------------------------
HsApoH            92   --------------------------------------------------------------------------------
HsApoJ            83   --------------------------------------------------------------------------------
HsApoL1          106   --------------------------------------------------------------------------------
HsApoL2           47   --------------------------------------------------------------------------------
HsApoL3           47   --------------------------------------------------------------------------------
HsApoL4           71   --------------------------------------------------------------------------------
HsApoL5           69   --------------------------------------------------------------------------------
HsApoL6           42   --------------------------------------------------------------------------------
HsApoM            32   --------------------------------------------------------------------------------
HsApoO            36   --------------------------------------------------------------------------------
HsApoOL           38   --------------------------------------------------------------------------------
HsMTTP            99   --------------------------------------------------------------------------------
DmMtp            104   --------------------------------------------------------------------------------

DmApoLppI        351   NNDKNELVVYGQYEQDKEIKKSKIGFSKNGNEYKPLIEIQDNNGISNSINGYHADGKIVVKKNS----NNIERYNFENFQ
DmApoLppII       114   --------------------------------------------------------------------------------
DmApoLTPI        558   EDSFLDGTQTRASLELKLPRSKIDYRILVKHKEHIKNGTEHNVIVGLKYTPEKEITGLFSVHLPRRNLFAIDAYMNVTVP
DmApoLTPII       109   --------------------------------------------------------------------------------
DmCG31659         32   --------------------------------------------------------------------------------
DmCvD            234   AHAKGTTLIHTFESTGEAQFINSELLLNFLNETPIDNPIDIETSMAAEPSNLELQRLDPNDPTGGRSPQQQETLIAQAGT
DmFabp            40   --------------------------------------------------------------------------------
DmGlaz            42   --------------------------------------------------------------------------------
DmMic26-27        47   --------------------------------------------------------------------------------
DmNlaz            32   --------------------------------------------------------------------------------
HsApoA1           31   --------------------------------------------------------------------------------
HsApoA2           14   --------------------------------------------------------------------------------
HsApoA4           62   --------------------------------------------------------------------------------
HsApoA5           62   --------------------------------------------------------------------------------
HsApoB48         342   IQRANLFNKLVTELRGLSDEAVTSLLPQLIEVSSPITLQALVQCGQPQCSTHILQWLKRVHANP----LLIDVVTYLVAL
HsApoB52         371   SDWWTLAAKNLTDFAEQYSIQDWAKRMKALVEQGFTVPEIKTILGTMPAFEVSLQALQKATFQTPDFIVPLTDLRIPSVQ
HsApoC1           33   --------------------------------------------------------------------------------
HsApoC2           25   --------------------------------------------------------------------------------
HsApoC3           29   --------------------------------------------------------------------------------
HsApoC4           38   --------------------------------------------------------------------------------
HsApoD            74   --------------------------------------------------------------------------------
HsApoE2           82   --------------------------------------------------------------------------------
HsApoF            49   --------------------------------------------------------------------------------
HsApoH            92   --------------------------------------------------------------------------------
HsApoJ            83   --------------------------------------------------------------------------------
HsApoL1          106   --------------------------------------------------------------------------------
HsApoL2           47   --------------------------------------------------------------------------------
HsApoL3           47   --------------------------------------------------------------------------------
HsApoL4           71   --------------------------------------------------------------------------------
HsApoL5           69   --------------------------------------------------------------------------------
HsApoL6           42   --------------------------------------------------------------------------------
HsApoM            32   --------------------------------------------------------------------------------
HsApoO            36   --------------------------------------------------------------------------------
HsApoOL           38   --------------------------------------------------------------------------------
HsMTTP            99   --------------------------------------------------------------------------------
DmMtp            104   --------------------------------------------------------------------------------
```

```
DmApoLppI     427  VSNSNNAHVAVNGWSDVGTNSLTSELRISLDHQTFLIKENLKLENGLYEAGFFINDEHSPENIYGSSIHLTIADQSYALK
DmApoLppII    114  --------------------------------------------------------------------------------
DmApoLTPI     638  EFNSCTASLKVNEKATKDYIIFINGSWFTGHSVAVKANYKDRSSRVQALHHLKMIVESPSFNITSLNIIYRRKQLLIFYD
DmApoLTPII    109  -----------------------------------------------NESDSSEKTKEYDYYDNLANEESESQNYDNMHP
DmCG31659      32  --------------------------------------------------------------------------------
DmCvD         314  LLDSLAEALETTEFKFSEPYDSTLSDVIKLLSEMDFDSLTKLYREVDIGTSYRQETIRNIFHEIIPRIGTKASVFLTHHL
DmFabp         40  --------------------------------------------------------------------------------
DmGlaz         42  --------------------------------------------------------------------------------
DmMic26-27     47  --------------------------------------------------------------------------------
DmNlaz         32  --------------------------------------------------------------------------------
HsApoA1        31  --------------------------------------------------------------------------------
HsApoA2        14  --------------------------------------------------------------------------------
HsApoA4        62  --------------------------------------------------------------------------------
HsApoA5        62  --------------------------------------------------------------------------------
HsApoB48      418  IPEPSAQQLREIFNMARDQRSRATLYALSHAVNNYHKTNPTGTQELLDIANYLMEQIQDDCTGDEDYTYLILRVIGNMGQ
HsApoB52      451  INFKDLKNIKIPSRFSTPEFTILNTFHIPSFTIDFVEMKVKIIRTIDQMLNSELQWPVPDIYLRDLKVEDIPLARITLPD
HsApoC1        33  --------------------------------------------------------------------------------
HsApoC2        25  --------------------------------------------------------------------------------
HsApoC3        29  --------------------------------------------------------------------------------
HsApoC4        38  --------------------------------------------------------------------------------
HsApoD         74  --------------------------------------------------------------------------------
HsApoE2        82  --------------------------------------------------------------------------------
HsApoF         49  --------------------------------------------------------------------------------
HsApoH         92  --------------------------------------------------------------------------------
HsApoJ         83  --------------------------------------------------------------------------------
HsApoL1       106  --------------------------------------------------------------------------------
HsApoL2        47  --------------------------------------------------------------------------------
HsApoL3        47  --------------------------------------------------------------------------------
HsApoL4        71  --------------------------------------------------------------------------------
HsApoL5        69  --------------------------------------------------------------------------------
HsApoL6        42  --------------------------------------------------------------------------------
HsApoM         32  --------------------------------------------------------------------------------
HsApoO         36  --------------------------------------------------------------------------------
HsApoOL        38  --------------------------------------------------------------------------------
HsMTTP         99  -------------------------------KSIFKGKSPSKIMGKENLEALQRPTLLHLIHGKVKEFYSYQNEAVAIEN
DmMtp         104  -----------------------------------------------------RPFYISLVRGQPDKVIAHTSKDQSLLN

DmApoLppI     507  TNGKAAAWSIGSDGSFNFQKLADSNSARAGSLVENVEIQYKNKQVGGIKIMSNFDVNKMDVDVEISREQKIGSIIVKYES
DmApoLppII    114  -------------IQKPVQFTLVSGILEPQICSDSSDLDYSLNIKRAVVSLLQSGIEAEHEVDVFGMCPTHTSTSKVGNA
DmApoLTPI     718  IQAKYDQDPYGLTIQYASNAHNRNTNAEVRLKVKERDYWINAKLLSEQPKLLQLEIHMDKIRDVHIQVGLLNVDKRKELS
DmApoLTPII    142  KSSDFNVDLTKNLLRFAFHDGLISEVCPQEQETPWVLNIKKGILSAFQNTMMRFDVDANTTETDVSGQCQVQYALEDTDS
DmCG31659      32  --------------------------------------------------------------------------------
DmCvD         394  VLNKLTKPQIAVQLLIPMPFHIFELSAELVQKCEDFLNIGPDRPDVRQAAILSFATLIHNVYVAKGIDKEKFEEYVQKYF
DmFabp         40  --------------------------------------------------------------------------------
DmGlaz         42  --------------------------------------------------------------------------------
DmMic26-27     47  --------------------------------------------------------------------------------
DmNlaz         32  --------------------------------------------------------------------------------
HsApoA1        31  --------------------------------------------------------------------------------
HsApoA2        14  --------------------------------------------------------------------------------
HsApoA4        62  --------------------------------------------------------------------------------
HsApoA5        62  --------------------------------------------------------------------------------
HsApoB48      498  TMEQLTPELKSSILKCVQSTKPSLMIQKAAIQALRKMEPKDKDQEVLLQTFLDDASPGDKRLAAYLMLMRSPSQADINKI
HsApoB52      531  FRLPEIAIPEFIIPTLNLNDFQVPDLHIPEFQLPHISHTIEVPTFGKLYSILKIQSPLFTLDANADIGNGTTSANEAGIA
HsApoC1        33  --------------------------------------------------------------------------------
HsApoC2        25  --------------------------------------------------------------------------------
HsApoC3        29  --------------------------------------------------------------------------------
HsApoC4        38  --------------------------------------------------------------------------------
HsApoD         74  --------------------------------------------------------------------------------
HsApoE2        82  --------------------------------------------------------------------------------
HsApoF         49  --------------------------------------------------------------------------------
HsApoH         92  --------------------------------------------------------------------------------
HsApoJ         83  --------------------------------------------------------------------------------
HsApoL1       106  --------------------------------------------------------------------------------
HsApoL2        47  --------------------------------------------------------------------------------
HsApoL3        47  --------------------------------------------------------------------------------
HsApoL4        71  --------------------------------------------------------------------------------
HsApoL5        69  --------------------------------------------------------------------------------
HsApoL6        42  --------------------------------------------------------------------------------
HsApoM         32  --------------------------------------------------------------------------------
HsApoO         36  --------------------------------------------------------------------------------
HsApoOL        38  --------------------------------------------------------------------------------
HsMTTP        148  IKRGLASLFQTQLSSGTTNEVDISGNCKVTYQAHQDKVIKIKALDSCKIARSGFTTPNQVLGVSSKATSVTTYKIEDSFV
DmMtp         131  LERGIASLLQLRLDASQEEELDVSGLCRVSYNVKSSTKVEKTKRDCSLWDLRVNYNPEEALGVTQQAQETVFYELSSEGT
```

```
DmApoLppI        587   NQRHAQDYSLEASAKINKHSIDVISKCDFNGNVYVVDNSLVTSWGTLLSAKGEIGQRYSAQDININIQGNVQISGKDKVT
DmApoLppII       181   NII-----------------------------------------------------------------------------
DmApoLTPI        798   LELKWDANRDPSQRLGLLAEYNSPGTKHYDGNLMITYPERTIHFGFNSFTGGPKYFGKVHASWSINEVIEFEYEAGILPG
DmApoLTPII       222   SYVTIRKTKDINSCRQRYATHSVLQTTPYTF-------------------------------------------------
DmCG31659         32   --------------------------------------------------------------------------------
DmCvD            474   NAYLSDRDFDQKMLYLQGLNNLQLGNVANYLEPIVQDPNEHEDLKFQAAWTTLALADRRAERIYEVYWPIFESRNASLEL
DmFabp            40   --------------------------------------------------------------------------------
DmGlaz            42   --------------------------------------------------------------------------------
DmMic26-27        47   --------------------------------------------------------------------------------
DmNlaz            32   --------------------------------------------------------------------------------
HsApoA1           31   --------------------------------------------------------------------------------
HsApoA2           14   --------------------------------------------------------------------------------
HsApoA4           62   --------------------------------------------------------------------------------
HsApoA5           62   --------------------------------------------------------------------------------
HsApoB48         578   VQILPWEQNEQVKNFVASHIANILNSEELDIQDLKKLVKEALKESQLPTVMDFRKFSRNYQLYKSVSLPSLDPASAKIEG
HsApoB52         611   ASITAKGESKLEVLNFDFQANAQLSNPKINPLALKESVKFSSKYLRTEHGSEMLFFGNAIEGKSNTVASLHTEKNTLELS
HsApoC1           33   --------------------------------------------------------------------------------
HsApoC2           25   --------------------------------------------------------------------------------
HsApoC3           29   --------------------------------------------------------------------------------
HsApoC4           38   --------------------------------------------------------------------------------
HsApoD            74   --------------------------------------------------------------------------------
HsApoE2           82   --------------------------------------------------------------------------------
HsApoF            49   --------------------------------------------------------------------------------
HsApoH            92   --------------------------------------------------------------------------------
HsApoJ            83   --------------------------------------------------------------------------------
HsApoL1          106   --------------------------------------------------------------------------------
HsApoL2           47   --------------------------------------------------------------------------------
HsApoL3           47   --------------------------------------------------------------------------------
HsApoL4           71   --------------------------------------------------------------------------------
HsApoL5           69   --------------------------------------------------------------------------------
HsApoL6           42   --------------------------------------------------------------------------------
HsApoM            32   --------------------------------------------------------------------------------
HsApoO            36   --------------------------------------------------------------------------------
HsApoOL           38   --------------------------------------------------------------------------------
HsMTTP           228   IAVLAEETHNFGLNFLQTIKGKIVSKQKLELKTTEAGPRLMSGKQAAAIIKAVDSKYTAIPIVGQVFQSHCKGCPSLSEL
DmMtp            211   LLHAESQENHRLNLAAKPDVGSFVKSSLILQHVSQGSEEVKQLQLGSLDKAIQSLLEWYRVFELESDVDGMISAIKEQ--

DmApoLppI        667   QWILKVIGTPDKTNSDFRISRDTSELIKLTSESQHPQDKISFAKLNLIVKNQLTAKGEFRVAKNGKGDFTASIDTLKTEP
DmApoLppII       184   --------------------------------------------------------------------------------
DmApoLTPI        878   HTLHNWVKAELRTPFDGWRVNSLDAGIYSLKNLILVNSTLFWADDQKLQVGYKSDYDVNDQLMSFDVRFGINSTIRDIPT
DmApoLTPII       253   --------------------------------------------------------------------------------
DmCG31659         32   --------------------------------------------------------------------------------
DmCvD            554   RVAAVTLLLISNPTAARLISIHRIIQSETDPHMINYYRTTVT--------------------------------------
DmFabp            40   --------------------------------------------------------------------------------
DmGlaz            42   --------------------------------------------------------------------------------
DmMic26-27        47   --------------------------------------------------------------------------------
DmNlaz            32   --------------------------------------------------------------------------------
HsApoA1           31   --------------------------------------------------------------------------------
HsApoA2           14   --------------------------------------------------------------------------------
HsApoA4           62   --------------------------------------------------------------------------------
HsApoA5           62   --------------------------------------------------------------------------------
HsApoB48         658   NLIFDPNNYLPKESMLKTTLTAFGFASADLIEIGLEGKGFEPTLEALFGKQGFFPDSVNKALYWVNGQVPDGVSKVLVDH
HsApoB52         691   NGVIVKINNQLTLDSNTKYFHKLNIPKLDFSSQADLRNEIKTLLKAGHIAWTSSGKGSWKWACPRFSDEGTHESQISFTI
HsApoC1           33   --------------------------------------------------------------------------------
HsApoC2           25   --------------------------------------------------------------------------------
HsApoC3           29   --------------------------------------------------------------------------------
HsApoC4           38   --------------------------------------------------------------------------------
HsApoD            74   --------------------------------------------------------------------------------
HsApoE2           82   --------------------------------------------------------------------------------
HsApoF            49   --------------------------------------------------------------------------------
HsApoH            92   --------------------------------------------------------------------------------
HsApoJ            83   --------------------------------------------------------------------------------
HsApoL1          106   --------------------------------------------------------------------------------
HsApoL2           47   --------------------------------------------------------------------------------
HsApoL3           47   --------------------------------------------------------------------------------
HsApoL4           71   --------------------------------------------------------------------------------
HsApoL5           69   --------------------------------------------------------------------------------
HsApoL6           42   --------------------------------------------------------------------------------
HsApoM            32   --------------------------------------------------------------------------------
HsApoO            36   --------------------------------------------------------------------------------
HsApoOL           38   --------------------------------------------------------------------------------
HsMTTP           308   WRSTRKYLQPDNLSKAEAVRNFLAFIQHLRTAKKEEILQILKMENKEVLPQLVDAVTSAQTS------------------
DmMtp            289   --------------------------------------------------------------------------------
```

```
DmApoLppI        747   KHKLEIESKFHIQSPKYDIDASLTLDGKRKVHLKSENTIEKLKFSTKNIGEANDKIIAFEANGSLKGELRGNGEIQGTFI
DmApoLppII       184   --------------------------------------------------------------------------------
DmApoLTPI        958   INVKVKHWMDVKKVDTELYLGYSGQNDTFNTYSMDSSWEIEKNQRYNNYSGLVHLVSPFKGYEKGGLVAHFSLSDQRVVS
DmApoLTPII       253   --------------------------------------------------------------------------------
DmCG31659         32   --------------------------------------------------------------------------------
DmCvD            596   --------------------------------------------------------------------------------
DmFabp            40   --------------------------------------------------------------------------------
DmGlaz            42   --------------------------------------------------------------------------------
DmMic26-27        47   --------------------------------------------------------------------------------
DmNlaz            32   --------------------------------------------------------------------------------
HsApoA1           31   --------------------------------------------------------------------------------
HsApoA2           14   --------------------------------------------------------------------------------
HsApoA4           62   --------------------------------------------------------------------------------
HsApoA5           62   --------------------------------------------------------------------------------
HsApoB48         738   FGYTKDDKHEQDMVNGIMLSVEKLIKDLKSKEV-----------------------------------------------
HsApoB52         771   EGPLTSFGLSNKINSKHLRVNQNLVYESGSLNFSKLEIQSQVDSQHVGHSVLTAKGMALFGEGKAEFTGRHDAHLNGKVI
HsApoC1           33   --------------------------------------------------------------------------------
HsApoC2           25   --------------------------------------------------------------------------------
HsApoC3           29   --------------------------------------------------------------------------------
HsApoC4           38   --------------------------------------------------------------------------------
HsApoD            74   --------------------------------------------------------------------------------
HsApoE2           82   --------------------------------------------------------------------------------
HsApoF            49   --------------------------------------------------------------------------------
HsApoH            92   --------------------------------------------------------------------------------
HsApoJ            83   --------------------------------------------------------------------------------
HsApoL1          106   --------------------------------------------------------------------------------
HsApoL2           47   --------------------------------------------------------------------------------
HsApoL3           47   --------------------------------------------------------------------------------
HsApoL4           71   --------------------------------------------------------------------------------
HsApoL5           69   --------------------------------------------------------------------------------
HsApoL6           42   --------------------------------------------------------------------------------
HsApoM            32   --------------------------------------------------------------------------------
HsApoO            36   --------------------------------------------------------------------------------
HsApoOL           38   --------------------------------------------------------------------------------
HsMTTP           370   --------------------------------------------------------------------------------
DmMtp            289   --------------------------------------------------------------------------------

DmApoLppI        827   FNAPDGRVIDGSINRKISTNAKSGLSQGNIDAQL----------------------------------------------
DmApoLppII       184   ----------TKARNLNSCSHREQINSGLVSGKV----------------------------------------------
DmApoLTPI       1038   GAASLNFDLREFTLTMNGYVKKFTDNMLTVNITTPLEKFGTINARFGLNEKKRHAVAEVRAPTAALGVEVLADIKNLLNF
DmApoLTPII       253   ----------RDDKTIWPILKSQSHCNLTIDNNV----------------------------------------------
DmCG31659         32   ------------------------------AVGD----------------------------------------------
DmCvD            596   ----------SISETTYPCYQHLRRLLSYMHRHL----------------------------------------------
DmFabp            40   --------------------------------------------------------------------------------
DmGlaz            42   --------------------------------------------------------------------------------
DmMic26-27        47   -----------------------LRKTEPKPERH----------------------------------------------
DmNlaz            32   -------------------------CPDVKLLDT----------------------------------------------
HsApoA1           31   --------------------------------------------------------------------------------
HsApoA2           14   --------------------------------------------------------------------------------
HsApoA4           62   ---------------FQDKLGEVNTYAGDLQKKL----------------------------------------------
HsApoA5           62   ----------QDLNNMNKFLEKLRPLSGSEAPRL----------------------------------------------
HsApoB48         771   ----------PEARAYLRILGEELGFASLHDLQL----------------------------------------------
HsApoB52         851   GTLKNSLFFSAQPFEITASTNNEGNLKVRFPLRL----------------------------------------------
HsApoC1           33   --------------------------------------------------------------------------------
HsApoC2           25   --------------------------------------------------------------------------------
HsApoC3           29   --------------------------------------------------------------------------------
HsApoC4           38   ---------------------------------P----------------------------------------------
HsApoD            74   ------IKVLNQELRADGTVNQIEGEATPVNLTE----------------------------------------------
HsApoE2           82   ---------------MDETMKELKAYKSELEEQL----------------------------------------------
HsApoF            49   ---------FPLSLESQTPSSDPLSCQFLHPKSL----------------------------------------------
HsApoH            92   --------------------NGAVRYTTFEYPNT----------------------------------------------
HsApoJ            83   ----------DALNETRESETKLKELPGVCNETM----------------------------------------------
HsApoL1          106   ----------NEADELRKALDNLARQMIMKDKNW----------------------------------------------
HsApoL2           47   ----------DEADELRKALNKLASHMVMKDKNR----------------------------------------------
HsApoL3           47   ----------DEADALYEALKKLRTYAAIEDEYV----------------------------------------------
HsApoL4           71   ----------EEADALYEALKNLTPYVAIEDKDM----------------------------------------------
HsApoL5           69   -----------DEAGMLSYFLFEELMRCDKDSMP----------------------------------------------
HsApoL6           42   --------------------------------------------------------------------------------
HsApoM            32   ----------------------------GVDGKE----------------------------------------------
HsApoO            36   ---------------------------KVDELSL----------------------------------------------
HsApoOL           38   ----------------------------PEQLPI----------------------------------------------
HsMTTP           370   ----------DSLEAILDFLDFKSDSSIILQERF----------------------------------------------
DmMtp            289   ----------TLEDQLKASLTELQSADVGKSSLA----------------------------------------------
```

```
DmApoLppI        861   -----------------------SDTPFGSNKK-----------------------------------------------
DmApoLppII       208   -----------------------NEKAGITSSL-----------------------------------------------
DmApoLTPI       1118   DVKLSVATPIESFQQAAIFALFNPERVDMRGLWNNVTLGFTGVWHMQNITDFEYSYHVFTPLAGFEENGFIVQLLKREKF
DmApoLTPII       277   -----------------------YKEIKCLETH-----------------------------------------------
DmCG31659         36   -----------------------LDMDRFKGKW-----------------------------------------------
DmCvD            620   -----------------------PQKPESRYWVT----------------------------------------------
DmFabp            40   --------------------------------------------------------------------------------
DmGlaz            42   -----------------------FNMSRVLGHW-----------------------------------------------
DmMic26-27        58   -----------------------PPQDSVLHKN-----------------------------------------------
DmNlaz            41   -----------------------FDAEAYMGVW-----------------------------------------------
HsApoA1           31   --------------------------------------------------------------------------------
HsApoA2           14   --------------------------------------------------------------------------------
HsApoA4           81   -----------------------VPFATELHER-----------------------------------------------
HsApoA5           86   -----------------------PQDPVGMRRQ-----------------------------------------------
HsApoB48         795   -----------------------LGKLLLMGAR-----------------------------------------------
HsApoB52         885   -----------------------TGKIDFLNNYA----------------------------------------------
HsApoC1           33   -----------------------LDKLKEFGNT-----------------------------------------------
HsApoC2           25   --------------------------------------------------------------------------------
HsApoC3           29   --------------------------------------------------------------------------------
HsApoC4           39   -----------------------PPKLKMSRWS-----------------------------------------------
HsApoD           102   -----------------------PAKLEVKFSW-----------------------------------------------
HsApoE2          101   -----------------------TPVAEETRAR-----------------------------------------------
HsApoF            74   -----------------------PGFSHMAPLP-----------------------------------------------
HsApoH           106   -----------------------ISFSCNTGFY-----------------------------------------------
HsApoJ           107   -----------------------MALWEECKPC-----------------------------------------------
HsApoL1          130   -----------------------HDKGQQYRNW-----------------------------------------------
HsApoL2           71   -----------------------HDKDQQHRQW-----------------------------------------------
HsApoL3           71   -----------------------QQKDEQFREW-----------------------------------------------
HsApoL4           95   -----------------------QQKEQQFREW-----------------------------------------------
HsApoL5           92   -----------------------DGNLSEEEKL-----------------------------------------------
HsApoL6           42   --------------------------------------------------------------------------------
HsApoM            38   -----------------------FPEVHLGQWY-----------------------------------------------
HsApoO            43   -----------------------YSVPEGQSKY-----------------------------------------------
HsApoOL           44   -----------------------YTAPPLQSKY-----------------------------------------------
HsMTTP           394   -----------------------LYACGFASHP-----------------------------------------------
DmMtp            313   -----------------------LAYVKLIPLAR----------------------------------------------

DmApoLppI        871   ---------------------------------------RSISLIGKLDR------------------------------
DmApoLppII       218   ---------------------------------------LLQANYIKESR------------------------------
DmApoLTPI       1198   VFQLHGKMSNYKLGVKINGEPKSDLVNQLGSNKMELEMLYDADFKPLNAETDYKPDADEEYFSYFTNFQVDTLVWPTIVG
DmApoLTPII       287   ---------------------------------------LLVPFSNASSG------------------------------
DmCG31659         46   --------------------------------------YTHSIYPHLSLR------------------------------
DmCvD            631   -------------------------------------GNYIFDYRDSKFG------------------------------
DmFabp            40   ----------------------------------------------VEVT------------------------------
DmGlaz            52   ---------------------------------------YEVERSFYLPE------------------------------
DmMic26-27        68   ---------------------------------------LEAGVRYVREE------------------------------
DmNlaz            51   ---------------------------------------YEYAAYPFAFE------------------------------
HsApoA1           31   --------------------------------------------------------------------------------
HsApoA2           14   --------------------------------------------------------------------------------
HsApoA4           91   ---------------------------------------LAKDSEKLKEE------------------------------
HsApoA5           96   ---------------------------------------LQEELEEVKAR------------------------------
HsApoB48         805   ---------------------------------------TLQGIPQMIGE------------------------------
HsApoB52         896   -------------------------------------LFLSPSAQQASWQ------------------------------
HsApoC1           43   ---------------------------------------LEDKARELISR------------------------------
HsApoC2           25   --------------------------------------------------------------------------------
HsApoC3           29   --------------------------------------------------------------------------------
HsApoC4           49   --------------------------------------------------------------------------------
HsApoD           112   ---------------------------------------FMPSAPYWILA------------------------------
HsApoE2          111   ---------------------------------------LSKELQAAQAR------------------------------
HsApoF            84   ---------------------------------------KFLVSLALRNA------------------------------
HsApoH           116   ---------------------------------------LNGADSAKCTE------------------------------
HsApoJ           117   ---------------------------------------LKQTCMKFYAR------------------------------
HsApoL1          140   ---------------------------------------FLKEFPRLKSE------------------------------
HsApoL2           81   ---------------------------------------FLKEFPRLKRE------------------------------
HsApoL3           81   ---------------------------------------FLKEFPQVKRK------------------------------
HsApoL4          105   ---------------------------------------FLKEFPQIRWK------------------------------
HsApoL5          102   ---------------------------------------FLSYFPLHKFE------------------------------
HsApoL6           42   ---------------------------------------FLREFPRLKED------------------------------
HsApoM            48   ---------------------------------------FIAGAAPTKEE------------------------------
HsApoO            53   -------------------------------------------VEEARSQ------------------------------
HsApoOL           54   ---------------------------------------VEEQPGHLQMG------------------------------
HsMTTP           404   ---------------------------------------NEELLRALISK------------------------------
DmMtp            324   -------------------------------------ITRQEQFEDLLTE------------------------------
```

```
DmApoLppI        882   --------------------------------------------------------------------------------
DmApoLppII       229   --------------------------------------------------------------------------------
DmApoLTPI       1278   NVDIQEIIDFYLVVGHVELPQGKVEFKDRLHYPDYINVHNLLTVTTPFAVAKNIKSIVEYHVDLNFNAFYERVKFIVNDD
DmApoLTPII       298   --------------------------------------------------------------------------------
DmCG31659         58   --------------------------------------------------------------------------------
DmCvD            644   --------------------------------------------------------------------------------
DmFabp            44   --------------------------------------------------------------------------------
DmGlaz            63   --------------------------------------------------------------------------------
DmMic26-27        79   --------------------------------------------------------------------------------
DmNlaz            62   --------------------------------------------------------------------------------
HsApoA1           31   --------------------------------------------------------------------------------
HsApoA2           14   --------------------------------------------------------------------------------
HsApoA4          102   --------------------------------------------------------------------------------
HsApoA5          107   --------------------------------------------------------------------------------
HsApoB48         816   --------------------------------------------------------------------------------
HsApoB52         909   --------------------------------------------------------------------------------
HsApoC1           54   --------------------------------------------------------------------------------
HsApoC2           25   --------------------------------------------------------------------------------
HsApoC3           29   --------------------------------------------------------------------------------
HsApoC4           49   --------------------------------------------------------------------------------
HsApoD           123   --------------------------------------------------------------------------------
HsApoE2          122   --------------------------------------------------------------------------------
HsApoF            95   --------------------------------------------------------------------------------
HsApoH           127   --------------------------------------------------------------------------------
HsApoJ           128   --------------------------------------------------------------------------------
HsApoL1          151   --------------------------------------------------------------------------------
HsApoL2           92   --------------------------------------------------------------------------------
HsApoL3           92   --------------------------------------------------------------------------------
HsApoL4          116   --------------------------------------------------------------------------------
HsApoL5          113   --------------------------------------------------------------------------------
HsApoL6           53   --------------------------------------------------------------------------------
HsApoM            59   --------------------------------------------------------------------------------
HsApoO            60   --------------------------------------------------------------------------------
HsApoOL           65   --------------------------------------------------------------------------------
HsMTTP           415   --------------------------------------------------------------------------------
DmMtp            337   --------------------------------------------------------------------------------

DmApoLppI        882   ------------------------------------------LNT--KTKEFSANSNLVYTAFNGEKS------------
DmApoLppII       229   ------------------------------------------IVN--HLIENVQLTETYKFIGNTKRNSDI---------
DmApoLTPI       1358   KDNTQELGFVFNYTALQDNVKPKAHDVQVTLLTPYEMLHEIYVHGHIELDDNAYKGNISAVTAHTHLSMAASIENEDNFL
DmApoLTPII       298   ------------------------------------------ALT--TSTSRLKLDGVESYSAGEFLEQNPELV------
DmCG31659         58   ------------------------------------------VEK-----------------------------------
DmCvD            644   ------------------------------------------IGA--MLQVFLVGDPKSDMPVVAFFKFDTEALG-----
DmFabp            44   ------------------------------------------LEG--D--------------------------------
DmGlaz            63   --------------------------------------------------------------------------------
DmMic26-27        79   ------------------------------------------VQS--GYKAVA---------------------------
DmNlaz            62   ------------------------------------------IGK-----------------------------------
HsApoA1           31   --------------------------------------------------------------------------------
HsApoA2           14   --------------------------------------------------------------------------------
HsApoA4          102   ------------------------------------------IGK--ELE------------------------------
HsApoA5          107   ------------------------------------------LQP--YMAEAHELVGWNLE-------------------
HsApoB48         816   ------------------------------------------VIR--KGSKNDFFLHYIFMENAFELPTGAGLQLQI---
HsApoB52         909   ------------------------------------------VSA--RFNQYKYNQNFSAGNNENIMEAHVGINGEANLD
HsApoC1           54   ------------------------------------------IKQ--S--------------------------------
HsApoC2           25   --------------------------------------------------------------------------------
HsApoC3           29   --------------------------------------------------------------------------------
HsApoC4           49   --------------------------------------------------------------------------------
HsApoD           123   ------------------------------------------T----DYENYALV-------------------------
HsApoE2          122   ------------------------------------------LGA--DME------------------------------
HsApoF            95   ------------------------------------------LEE--AGCQAD---------------------------
HsApoH           127   ------------------------------------------EGK--WSPEL----------------------------
HsApoJ           128   ------------------------------------------VCR--SGSGLVGRQLEEFL-------------------
HsApoL1          151   ------------------------------------------LED--NIRRLRALADGVQK-------------------
HsApoL2           92   ------------------------------------------LED--HIRKLRALAEEVEQ-------------------
HsApoL3           92   ------------------------------------------IQE--SIEKLRALANGIEE-------------------
HsApoL4          116   ------------------------------------------IQE--SIERLRVIANEIEK-------------------
HsApoL5          113   ------------------------------------------LEQ--NIKELNTLADQVDT-------------------
HsApoL6           53   ------------------------------------------LKG--NIDKLRALADDIDK-------------------
HsApoM            59   ------------------------------------------LAT--F--------------------------------
HsApoO            60   ------------------------------------------LEE--SISQLR---------------------------
HsApoOL           65   ------------------------------------------FASIR---------------------------------
HsMTTP           415   ------------------------------------------FKG--SIGSSDIRETVMIITGTLVRKLC----------
DmMtp            337   ------------------------------------------HAE--VLPQLVDLLGAVQTFD-----------------
```

```
DmApoLppI      906  -------------EISYQIKQQPN--GDAKNIDFSLKAYGNPLPQP--------------------------FEIAFALG
DmApoLppII     256  -------------SAKVVTILKLKNPSGTKANSPGTGSTVRSLIFQRPETYTSKNI----------------NALKTILS
DmApoLTPI     1438  ETSVGIQLETDAIPHYGCQVYFKK--DFSAVDKAIDIRFEVTDNGTLNQLHISTDWHTDPSYIVNANGRIKTTMLPLQMA
DmApoLTPII     328  -------------ERRATLVFDHT--PAVKPSHDEIKAARELLV----------------------------EMCRVGFP
DmCG31659       61  -------------CQSTDFIEKEE------------------------------------------------NKFSVVAR
DmCvD          675  -------------KFTGQLALYIKARGLPDTILNKMQSRNGSDPFTFKSIK---------------------ALLAMLQA
DmFabp          48  -------------TYTLTTTSTFK--TSAISFKLGVE-------------------------------------------
DmGlaz          63  -------------IASGCTTFQFE--PYNKGEQSKFS-------------------------------------------
DmMic26-27      88  -------------DQAGIVGHYVE--TAKAHTQSTI------------------------------------DMLNEPQN
DmNlaz          65  ----------------KCIYANYSLIDNSTV-----------------------------------------SVVNAAIN
HsApoA1         31  -------------QKVEPLRAELQ--EGARQKLHEL--------------------------------------------
HsApoA2         14  -------------SLEGALVR-----------------------------------------------------------
HsApoA4        108  -------------ELRARLLPHAN--EVSQKIGDNL------------------------------------RELQQRLE
HsApoA5        124  -------------GLRQQLKPYTM--DLMEQVALRV------------------------------------QELQEQLR
HsApoB48       849  -------------SSSGVIAPGAK--AGVKLEVANMQAELVAKPSVSVEFV---------------------TNMGIIIP
HsApoB52       945  FLNIPLTIPEMRLPYTIITTPPLK--DFSLWEKTGLKEFLKTTKQSFDLSVKAQYKKNKHRHSITNPLAVLCEFISQSIK
HsApoC1         58  -------------ELSAKMRLEPF--PG----------------------------------------------------
HsApoC2         25  --------------------------------------------------------------------------------
HsApoC3         29  -------------SFMQGYMKHAT--KTAKDALSSVQ-------------------------------------------
HsApoC4         49  -------------LVRGRM-------------------------------------------------------------
HsApoD         132  -------------YSCTCIIQLFH--VDFAWILARNP-----------------------------------NLPPETVD
HsApoE2        128  -------------DVCGRLVQYRG--EVQAMLGQST------------------------------------EELRVRLA
HsApoF         104  -------------VWALQLQLYRQ--GGVNATQVLIQ-----------------------------------HLRGLQKG
HsApoH         135  -------------PVCAPIICPPP--SIPTFATLRV------------------------------------YKPSAGNN
HsApoJ         145  -------------NQSSPFYFWMNGDRIDSLLENDRQQT---------------------------------HMLDVMQD
HsApoL1        168  -------------VHKGTTIANVV--SGSLSISSGIL-----------------------------------TLVGMGLA
HsApoL2        109  -------------VHRGTTIANVV--SNSVGTTSGIL-----------------------------------TLLGLGLA
HsApoL3        109  -------------VHRGCTISNVV--SSSTGAASGIM-----------------------------------SLAGLVLA
HsApoL4        133  -------------VHRGCVIANVV--SGSTGIL---------------------------------------SVIGVMLA
HsApoL5        130  -------------THELLTKTSLV--ASSSGAVSGVM-----------------------------------NILGLALA
HsApoL6         70  -------------THKKFTKANMV--ATSTAVISGVM-----------------------------------SLLGLALA
HsApoM          63  -------------DPVDNIVFNMA--AGSAPMQLHLRATI----------------------------------------
HsApoO          69  -------------HYCEPYTTWCQ--ETYSQTKPKMQ-----------------------------------SLVQWGLD
HsApoOL         70  -------------TATGCYIGWCK--GVYVFVKNGIM-----------------------------------DTVQFGKD
HsMTTP         441  -------------QNEGCKLKAVV--EAKKLILGGLEKAEKKEDTRMYLLALKN------------------ALLPEGIP
DmMtp          356  -------------AHNATFGFLYKESETTSEQLDLLEKYLQSLAVATHPDRKIVE-----------------HLFGLLEQ

DmApoLppI      945  --------------------------------------------------------------------------------
DmApoLppII     307  --------------------------------------------------------------------------------
DmApoLTPI     1516  STSVLVIQGQNPHLNFDLNLLSQNGQSIAYGARANKKKDVFNIEVWTPMKNFRNISMHGTAIRSPRDPGRYDVSGFLYRN
DmApoLTPII     365  --------------------------------------------------------------------------------
DmCG31659       80  --------------------------------------------------------------------------------
DmCvD          721  --------------------------------------------------------------------------------
DmFabp          70  --------------------------------------------------------------------------------
DmGlaz          85  --------------------------------------------------------------------------------
DmMic26-27     117  --------------------------------------------------------------------------------
DmNlaz          88  --------------------------------------------------------------------------------
HsApoA1         52  --------------------------------------------------------------------------------
HsApoA2         22  --------------------------------------------------------------------------------
HsApoA4        137  --------------------------------------------------------------------------------
HsApoA5        153  --------------------------------------------------------------------------------
HsApoB48       893  --------------------------------------------------------------------------------
HsApoB52      1023  --------------------------------------------------------------------------------
HsApoC1         71  --------------------------------------------------------------------------------
HsApoC2         25  --------------------------------------------------------------------------------
HsApoC3         51  --------------------------------------------------------------------------------
HsApoC4         55  --------------------------------------------------------------------------------
HsApoD         162  --------------------------------------------------------------------------------
HsApoE2        157  --------------------------------------------------------------------------------
HsApoF         134  --------------------------------------------------------------------------------
HsApoH         164  --------------------------------------------------------------------------------
HsApoJ         179  --------------------------------------------------------------------------------
HsApoL1        198  --------------------------------------------------------------------------------
HsApoL2        139  --------------------------------------------------------------------------------
HsApoL3        139  --------------------------------------------------------------------------------
HsApoL4        159  --------------------------------------------------------------------------------
HsApoL5        160  --------------------------------------------------------------------------------
HsApoL6        100  --------------------------------------------------------------------------------
HsApoM          88  --------------------------------------------------------------------------------
HsApoO          99  --------------------------------------------------------------------------------
HsApoOL        100  --------------------------------------------------------------------------------
HsMTTP         488  --------------------------------------------------------------------------------
DmMtp          406  --------------------------------------------------------------------------------
```

```
DmApoLppI        945   ----------------DYSA-QHAVVSITSKYGEIFSVSANGNYNNNQALEYGLQANIEIPKSTLKSLEINSHGKVLKSL
DmApoLppII       307   ----------------DLVD-------------------STGDYVKKETAKKFVEFIRLLRQSDSETLLELAAFPHPNKV
DmApoLTPI       1596   MATYEVTGAVRMTNSLPIDVVLRVQPKAGGRDGVIELNIHEAGPKKIRFSFSAIEDGKMCQMSGGYSVSKTNGAMDFSVL
DmApoLTPII       365   ----------------NIQR---------------------------EFIDVFTNFLQTSKSLDYKTLSVLLQRSASTCE
DmCG31659         80   --------------------------------------------------------------------------------
DmCvD            721   ----------------PIIN------------------------------SKDLHLEFILQMEGKTVLSYYLNQRMFRQL
DmFabp            70   --------------------------------------------------------------------------------
DmGlaz            85   --------------------------------------------------------------------------------
DmMic26-27       117   ----------------SLHR------------------------------------------------------------
DmNlaz            88   ----------------RFTG------------------------------------------------------------
HsApoA1           52   --------------------------------------------------------------------------------
HsApoA2           22   --------------------------------------------------------------------------------
HsApoA4          137   ----------------PYAD--------------------------------QLRTQVSTQAEQLRRQLTPYAQRMERVL
HsApoA5          153   ----------------VVGE------------------------------------DTKAQLLGGVDEAWALLQGLQSRV
HsApoB48         893   ----------------DFAR-------------------SGVQMNTNFFHESGLEAHVALKAGKLKFIIPSPKRPVKLLS
HsApoB52        1023   ----------------SFDRHFEKNRNNALDFVTKSYNETKIKFDKYKAEKSHDELPRTFQIPGYTVPVVNVEVSPFTIE
HsApoC1           71   --------------------------------------------------------------------------------
HsApoC2           25   --------------------------------------------------------------------------------
HsApoC3           51   --------------------------------------------------------------------------------
HsApoC4           55   --------------------------------------------------------------------------------
HsApoD           162   ----------------SLKN------------------------------------------------------------
HsApoE2          157   ----------------SHLR------------------------------------------------------KLRKRL
HsApoF           134   ----------------RSTE------------------------------------------------------------
HsApoH           164   ----------------SLYR------------------------------------------------------------
HsApoJ           179   ----------------HFSR------------------------------ASSIIDELFQDRFFTREPQDTYHYLPFSLP
HsApoL1          198   ----------------PFTE------------------------------------------------------------
HsApoL2          139   ----------------PFTE------------------------------------------------------------
HsApoL3          139   ----------------PFTA------------------------------------------------------------
HsApoL4          159   ----------------PFTA------------------------------------------------------------
HsApoL5          160   ----------------PVTA------------------------------------------------------------
HsApoL6          100   ----------------PATG------------------------------------------------------------
HsApoM            88   --------------------------------------------------------------------------------
HsApoO            99   ----------------SYDY------------------------------------------------------------
HsApoOL          100   ----------------AYVY------------------------------------------------------------
HsMTTP           488   ----------------SLLK-------------------------YAEAGEGPISHLATTALQRYDLPFITDEVKKTLNR
DmMtp            406   ----------------ESIK------------------------KHLKLRESVIQTVATLTRQSGLDVEDPLLKEVRSYL

DmApoLppI       1008   IGNENAAYNVEFFLDSKTSLGQYARVNTVWNGTANDGSYDFEAQTNNM----ESPLKFNGKY------------------
DmApoLppII       352   LARKVYLDGLFRTSTAESARVILKQLSKFDEKEKLLAILSLNIVKSVDKETLNQAASQLLPN-----APKELYIAVGNLV
DmApoLTPI       1676   VESTEPEIARINFYGNLSPNSEGSLVGDLSLETPWKALGIDTVHLHSDVGFLNKGGHIVGEYKIGQYIGRGSCLWSWILA
DmApoLTPII       402   QGRNHLLESLPFIGSTASYKVMRDQII-----NEKLTKQMAHDWMTALSFITRPDEETLETF------------------
DmCG31659         80   --------------------------------ELNTQTGTVKMRKADILNVEPEFGRYVLGT------------------
DmCvD            755   TYDNILERMQQIIRTDSHINMQTVRWPFMNRYTVPTVLGT----SSDVLLQTTVLTSLRGNI--TEQRNSPITKHTLEID
DmFabp            70   --------------------------------FDEETLDG------------RNVKSIITLD------------------
DmGlaz            85   --------------------------------NFKLAVAI------------KNINRITGNP------------------
DmMic26-27       121   --------------------------------SGAIVVGG----LAGF------IFAARGGF------------------
DmNlaz            92   --------------------------------QPSNVTGQ----AKVL-----GPGQLAVAF------------------
HsApoA1           52   --------------------------------QEKLSPLG----EEMR----DRARAHVDAL------------------
HsApoA2           22   --------------------------------RQAKEPCV----ESLV----SQYFQTVTDY------------------
HsApoA4          169   RENADSLQASLRPHADELKAKIDQNVEEL---KGRLTPYA----DEFK----VKIDQTVEEL------------------
HsApoA5          181   VHHTGRFKELFHPYAESLVSGIGRHVQEL---HRSVAPHA----PASP----ARLSRCVQVL------------------
HsApoB48         938   GGNTLHLVSTTKTEVIPPLIENRQSWSVCKQVFPGLNYCTSGAYSNASSTDSASYYPLTGDTRLELELRPTGEIEQYSVS
HsApoB52        1087   MSAFGYVFPKAVSMPSFSILGSDVRVPSYTLILPSLELPV----LHVP----RNLKLSLPDFKELCTISHIFIPAMGNIT
HsApoC1           71   --------------------------------HGRAGVCF----------------------------------------
HsApoC2           25   --------------------------------PQQDEMPS----PTFL----TQVKESLSSY------------------
HsApoC3           51   --------------------------------ESQVAQQA----RGWV----TDGFSSLKDY------------------
HsApoC4           55   ----------------------------------------------------KELLETVVNR------------------
HsApoD           166   ------ILTSNNIDVKKMTVTDQVNCPKL---SQAKEPCV----ESLV----SQYFQTVTDY------------------
HsApoE2          167   LRDADDLQKRLAVYQAGAREGAERGLSAI---RERLGPLV----EQGR----VRAATVGSLA------------------
HsApoF           138   --------------RNVSVEALASALQLLAREQQSTGRVG----RSLPTEDCENEKEQAVHN------------------
HsApoH           168   --------------------------------DTAVFECL----PQHAMFGNDTITCTTHGN------------------
HsApoJ           213   HRRPHFFFPKSRIVRSLMPFSPYEPLNFHAMFQPFLEMIHEAQQAMDIHFHSPAFQHPPTEF-------------IREGD
HsApoL1          202   ----------------------GGSLVLL---EPGMELGI----TAAL----TGITSSTMDY------------------
HsApoL2          143   ----------------------GISFVLL---DTGMGLGA----AAAV----AGITCSVVEL------------------
HsApoL3          143   ----------------------GTSLALT---AAGVGLGA----ASAV----TGITTSIVEH------------------
HsApoL4          163   ----------------------GLSLSIT---AAGVGLGI----ASAT----AGIASSIVEN------------------
HsApoL5          164   ----------------------GGSLMLS---ATGTGLGA----AAAI----TNIVTNVLEN------------------
HsApoL6          104   ----------------------GGSLLLS---TAGQGLAT----AAGV----TSIVSGTLER------------------
HsApoM            88   --------------------------------RMKDGLCV---PRKWI----YHLTEGSTDL------------------
HsApoO           103   ---------------------LQNAPPGF---FPRLGVIGF-----------AGLIGLLLAR------------------
HsApoOL          104   ---------------------LKNPPRDF---LPKMGVIT----VSGL----AGLVSARKGS------------------
HsMTTP           527   IYHQNRKVHEKTVRTAAAAIILNNNPSYM---DVKNILLSIGELPQEMNKYMLAIVQDILRF------------------
DmMtp            446   LQGLTSKEPTLYIRALQNLQDPATIEALLEHAQTGEAPNLSVAALQALKAFPLGSFNSSHRL------------------
```

```
DmApoLppI       1066   -HRKQ-------TGNIKDGDLTGKQTYVLNAQYGAQYVKMDASLGYGAEKVDIAYVIDSSFDSVKDIKVNIR-----TFK
DmApoLppII       427   AKYCL-------KNYCQGPE------------------------------------------------------------
DmApoLTPI       1756   EDMQLVLENYLERPNAKPRIVHASAKYQNPGQTFTQLQAGGRLSVDSKWNLDVNGSAEYKSVDDFKFRVITALPLPVGDR
DmApoLTPII       459   --HSI-------LEYAKNRL--------------------------------------------------DA-----EYT
DmCG31659        110   ------------TSTAFPEG------------------------------------------------------------
DmCvD            829   ARYSS-------YASVRSRSYNPFLNLDHEINREQGFLIYIPFSSELHLNESGSKCRRYSFSRPQNLTSGLS-----FKS
DmFabp            88   -GNKL-------TQEQKGDK------------------------------------------------------------
DmGlaz           103   -NVNI--------GYATPEN------------------------------------------------------------
DmMic26-27       141   -IKKV-------LYSGIGAG------------------------------------------------------------
DmNlaz           113   --YPT-------QPLTKANY------------------------------------------------------------
HsApoA1           74   -RTHL-------APYSDELR------------------------------------------------------------
HsApoA2           44   -GKDL-------MEKVKSPE------------------------------------------------------------
HsApoA4          220   -RRSL-------APYAQDTQ----------------------------------------------------------EK
HsApoA5          232   -SRKL-------TLKAKALH------------------------------------------------------------
HsApoB48        1018   ATYEL-------QREDRALVDTLKFVTQAEGAKQTEATMTFKYNRQSMTLSSEVQIPDFDVDLGTILRVNDE-----STE
HsApoB52        1159   YDFSF-------KSSVITLNTNAELFNQSDIVAHLLSSSSSVIDALQYKLEGTTRLTRKRGLKLATALSLSNKFVEGSHN
HsApoC1           79   -WVEP-------WQMVQDEQ------------------------------------------------------------
HsApoC2           47   ------------WESAKTAA------------------------------------------------------------
HsApoC3           73   ------------WSTVKDKF------------------------------------------------------------
HsApoC4           65   -TRDG-------WQWFWSPS------------------------------------------------------------
HsApoD           202   -GKDL-------MEKVKSPE------------------------------------------------------------
HsApoE2          218   -GQPL-------QERAQAWG------------------------------------------------------------
HsApoF           182   -VVQL-------LPGVGTFY------------------------------------------------------------
HsApoH           194   -WTKLPECREVKCPFPSRPD------------------------------------------------------------
HsApoJ           280   DDRTV-------CREIRHNS------------------------------------------------------------
HsApoL1          231   -GKKW-------WTQAQAHD------------------------------------------------------------
HsApoL2          172   -VNKL-------RARAQARN------------------------------------------------------------
HsApoL3          172   -SYTS-------SAEAEASR------------------------------------------------------------
HsApoL4          192   -TYTR-------SAELTASR------------------------------------------------------------
HsApoL5          193   -RSNS-------AARDKASR------------------------------------------------------------
HsApoL6          133   -SKNK-------EAQARAED------------------------------------------------------------
HsApoM           111   ------------RTEGRPDM------------------------------------------------------------
HsApoO           130   -GSKI-------KKLVYPPG------------------------------------------------------------
HsApoOL          134   -KFKK-------ITYPLGLA------------------------------------------------------------
HsMTTP           586   -EMPA-------SKIVRRVL------------------------------------------------------------
DmMtp            508   -QFES-------IFYQRKRRFDSSARTLALDIILSLRPTQEQLGNFLDYLASNDRQFEIKTYVLQKLRMLAEKCPRFRAL

DmApoLppI       1133   PLDDSTYVVTALFKQTDKSYGLDTTFYHSAHKKGVDIRLDLLKEKPIIISSIAELLGDRKGKVLFEILNLADLDIKINSE
DmApoLppII       440   ------------------IDAISKKFSDGLKH------------------------------------------------
DmApoLTPI       1836   HQLSASYQGNVISQQFNNPDFVLEASYESFEA------------QNKLLSRISYKNATNNLKGLGHVEWGKIQNLSVVEG
DmApoLTPII       475   LGATAVVHSFCKHHEACEENLRVQQIINLLET------------------------------------------------
DmCG31659        118   -----------------VLMYVLDTDYVNFAI------------------------------------------------
DmCvD            897   RAVTKTRGLITKTAAAPFEEIMVPEGRNDVVQ------------------------------------------------
DmFabp           100   ----------------------PTTIVREFTD------------------------------------------------
DmGlaz           114   ----------------------SRSSIMDFKF------------------------------------------------
DmMic26-27       153   ---------------------AVASMCYPRQA------------------------------------------------
DmNlaz           124   --------------------LVLGTDYESYAV------------------------------------------------
HsApoA1           86   --------------------QRLAARLEALKE------------------------------------------------
HsApoA2           56   ------------------LQAEAKSYFEKSKE------------------------------------------------
HsApoA4          234   LNHQLEGLTFQMKKNAEELKARISASAEELRQ------------------------------------------------
HsApoA5          244   --------------------ARIQQNLDQLRE------------------------------------------------
HsApoB48        1086   GKTSYRLTLDIQNKKITEVALMGHLSCDTKEE------------------------------------------------
HsApoB52        1232   STVSLTTKNMEVSVATTTKAQIPILRMNFKQE------------------------------------------------
HsApoC1           91   --------------------------IEKKTS------------------------------------------------
HsApoC2           55   ------------------------QNLYEKTY------------------------------------------------
HsApoC3           81   --------------------------------------------------------------------------------
HsApoC4           77   -----------------TFRGFMQTYYDDH--------------------------------------------------
HsApoD           223   ------------------LQAEAKSYFEKSKE------------------------------------------------
HsApoE2          230   --------------------ERLRARMEEMGS------------------------------------------------
HsApoF           194   --------------------NLGTALYYATQN------------------------------------------------
HsApoH           213   -------------NGFVNYPAKPTLYYKDKAT------------------------------------------------
HsApoJ           293   -----------------TGCLRMKDQCDKCRE------------------------------------------------
HsApoL1          243   ---------------------LVIKSLDKLKE------------------------------------------------
HsApoL2          184   ---------------------LDQSGTNVAKV------------------------------------------------
HsApoL3          184   ---------------------LTATSIDRLKV------------------------------------------------
HsApoL4          204   ---------------------LTATSTDQLEA------------------------------------------------
HsApoL5          205   -----------LGPLTTSHEAFGGINWSEIEA------------------------------------------------
HsApoL6          145   ---------------------ILPTYDQEDRE------------------------------------------------
HsApoM           119   ------------------KTELFSSSCPGGIM------------------------------------------------
HsApoO           142   ------------------FMGLAASLYYPQQA------------------------------------------------
HsApoOL          146   --------------------TLGATVCYPVQS------------------------------------------------
HsMTTP           598   -------------------KEMVAHNYDRFSR------------------------------------------------
DmMtp            580   FKSELVKRRHVNNYNVLGQKGLTTVLTRQLSQ------------------------------------------------
```

```
DmApoLppI       1213   ASYVSIDEFYIIVNWSSKKLKLDGYELEARAQSKNIKIQLKNENGIIFSGTATYALKKELNKTIIDGQGKVQYQGKALSG
DmApoLppII       454   --------------------------------------------------------------------------------
DmApoLTPI       1904   DFELLHKQGAQREFSAKIITPKFKNEHTFALTGSYDLEKSGHHNVVGSLDYPASRRITDLDVSVSSLSNMHGIFNSTLPT
DmApoLTPII       507   --------------------------------------------------------------------------------
DmCG31659        133   --------------------------------------------------------------------------------
DmCvD            929   --------------------------------------------------------------------------------
DmFabp           110   --------------------------------------------------------------------------------
DmGlaz           124   --------------------------------------------------------------------------------
DmMic26-27       164   --------------------------------------------------------------------------------
DmNlaz           136   --------------------------------------------------------------------------------
HsApoA1           98   --------------------------------------------------------------------------------
HsApoA2           70   --------------------------------------------------------------------------------
HsApoA4          266   --------------------------------------------------------------------------------
HsApoA5          256   --------------------------------------------------------------------------------
HsApoB48        1118   ---------------------------------------------RKIKGVISIPRLQAEARSEILAHWSPAKLLLQMDS
HsApoB52        1264   --------LNGNTKSKPTVSSSMEFKYDFNSSMLYSTAKGAVDHKLSLESLTSYFSIESSTKGDVKGSVLSREYSGTIAS
HsApoC1           97   --------------------------------------------------------------------------------
HsApoC2           63   --------------------------------------------------------------------------------
HsApoC3           81   --------------------------------------------------------------------------------
HsApoC4           90   --------------------------------------------------------------------------------
HsApoD           237   --------------------------------------------------------------------------------
HsApoE2          242   --------------------------------------------------------------------------------
HsApoF           206   --------------------------------------------------------------------------------
HsApoH           232   --------------------------------------------------------------------------------
HsApoJ           308   --------------------------------------------------------------------------------
HsApoL1          254   --------------------------------------------------------------------------------
HsApoL2          195   --------------------------------------------------------------------------------
HsApoL3          195   --------------------------------------------------------------------------------
HsApoL4          215   --------------------------------------------------------------------------------
HsApoL5          226   --------------------------------------------------------------------------------
HsApoL6          156   --------------------------------------------------------------------------------
HsApoM           133   --------------------------------------------------------------------------------
HsApoO           156   --------------------------------------------------------------------------------
HsApoOL          158   --------------------------------------------------------------------------------
HsMTTP           611   --------------------------------------------------------------------------------
DmMtp            612   --------------------------------------------------------------------------------

DmApoLppI       1293   NFKLTRQHFDFGTDREVGFSYTFMGNLGSKNGLGTLKITNKEFNTKFSVCEEKRQCTNLIVQSIVSIDEQKLDAVEHTTL
DmApoLppII       454   --------------------------------------------------------------------------------
DmApoLTPI       1984   FLNVSWLKTDFNFTTNNGKSYRYCRCFWPQDTAYFKLNSNYDSDSSNFNHNLNGNVEIEVPLATRHRADIVYGLQKRRNQ
DmApoLTPII       507   --------------------------------------------------------------------------------
DmCG31659        133   --------------------------------------------------------------------------------
DmCvD            929   --------------------------------------------------------------------------------
DmFabp           110   --------------------------------------------------------------------------------
DmGlaz           124   --------------------------------------------------------------------------------
DmMic26-27       164   --------------------------------------------------------------------------------
DmNlaz           136   --------------------------------------------------------------------------------
HsApoA1           98   --------------------------------------------------------------------------------
HsApoA2           70   --------------------------------------------------------------------------------
HsApoA4          266   --------------------------------------------------------------------------------
HsApoA5          256   --------------------------------------------------------------------------------
HsApoB48        1153   SATAYGSTVSKRVAWHYDEEKIEFEWNTGTNVDTKKMTSNFPVDLSDYPKSLHMYANRLLDHRVPQTDMTFRHVGSKLIV
HsApoB52        1336   EANTYLNSKSTRSSVKLQGTSKIDDIWNLEVKENFAGEATLQRIYSLWEHSTKNHLQLEGLFFTNGEHTSKATLELSPWQ
HsApoC1           97   --------------------------------------------------------------------------------
HsApoC2           63   --------------------------------------------------------------------------------
HsApoC3           81   --------------------------------------------------------------------------------
HsApoC4           90   --------------------------------------------------------------------------------
HsApoD           237   --------------------------------------------------------------------------------
HsApoE2          242   --------------------------------------------------------------------------------
HsApoF           206   --------------------------------------------------------------------------------
HsApoH           232   --------------------------------------------------------------------------------
HsApoJ           308   --------------------------------------------------------------------------------
HsApoL1          254   --------------------------------------------------------------------------------
HsApoL2          195   --------------------------------------------------------------------------------
HsApoL3          195   --------------------------------------------------------------------------------
HsApoL4          215   --------------------------------------------------------------------------------
HsApoL5          226   --------------------------------------------------------------------------------
HsApoL6          156   --------------------------------------------------------------------------------
HsApoM           133   --------------------------------------------------------------------------------
HsApoO           156   --------------------------------------------------------------------------------
HsApoOL          158   --------------------------------------------------------------------------------
HsMTTP           611   --------------------------------------------------------------------------------
DmMtp            612   --------------------------------------------------------------------------------
```

```
DmApoLppI      1373   IIVDLRDFGYPYEFELKSQNTRQGLKYQYHLDSFIITGNNFKYQFTANVQPTSSTIKLALPKRQILFETTQKIPADGSLF
DmApoLppII      454   --------------------------------------------------------------------------------
DmApoLTPI      2064   DAGNVKVVYNEKQVLDGKYKRLEQAKAPIYKETTDISLENEVKPLGIHFVSTRDASDPAGSQDVKHIEIYELRNTQNFNL
DmApoLTPII      507   --------------------------------------------------------------------------------
DmCG31659       133   --------------------------------------------------------------------------------
DmCvD           929   --------------------------------------------------------------------------------
DmFabp          110   --------------------------------------------------------------------------------
DmGlaz          124   --------------------------------------------------------------------------------
DmMic26-27      164   --------------------------------------------------------------------------------
DmNlaz          136   --------------------------------------------------------------------------------
HsApoA1          98   --------------------------------------------------------------------------------
HsApoA2          70   --------------------------------------------------------------------------------
HsApoA4         266   --------------------------------------------------------------------------------
HsApoA5         256   --------------------------------------------------------------------------------
HsApoB48       1233   AMSSWLQKASGSLPYTQTLQDHLNSLKEFNLQNMGLPDFHIPENLFLKSDGRVKYTLNKNSLKIEIPLPFGGKSSRDLKM
HsApoB52       1416   MSALVQVHASQPSSFHDFPDLGQEVALNANTKNQKIRWKNEVRIHSGSFQSQVELSNDQEKAHLDIAGSLEGHLRFLKNI
HsApoC1          97   --------------------------------------------------------------------------------
HsApoC2          63   --------------------------------------------------------------------------------
HsApoC3          81   --------------------------------------------------------------------------------
HsApoC4          90   --------------------------------------------------------------------------------
HsApoD          237   --------------------------------------------------------------------------------
HsApoE2         242   --------------------------------------------------------------------------------
HsApoF          206   --------------------------------------------------------------------------------
HsApoH          232   --------------------------------------------------------------------------------
HsApoJ          308   --------------------------------------------------------------------------------
HsApoL1         254   --------------------------------------------------------------------------------
HsApoL2         195   --------------------------------------------------------------------------------
HsApoL3         195   --------------------------------------------------------------------------------
HsApoL4         215   --------------------------------------------------------------------------------
HsApoL5         226   --------------------------------------------------------------------------------
HsApoL6         156   --------------------------------------------------------------------------------
HsApoM          133   --------------------------------------------------------------------------------
HsApoO          156   --------------------------------------------------------------------------------
HsApoOL         158   --------------------------------------------------------------------------------
HsMTTP          611   --------------------------------------------------------------------------------
DmMtp           612   --------------------------------------------------------------------------------

DmApoLppI      1453   GRYEQTASFFIDKLQKPDDVARFSAIVDVT--------------------------------------------------
DmApoLppII      454   --------------------------------------------------------------------------------
DmApoLTPI      2144   TGELHSRATLKAQDFKVVAIHPNRAVVLSTKYEDVSPEVVRHHSKLELSETAWIGYNLELGNFSKVGNESQSFALEIFYP
DmApoLTPII      507   --------------------------------------------------------------------------------
DmCG31659       133   --------------------------------------------------------------------------------
DmCvD           929   --------------------------------------------------------------------------------
DmFabp          110   --------------------------------------------------------------------------------
DmGlaz          124   --------------------------------------------------------------------------------
DmMic26-27      164   --------------------------------------------------------------------------------
DmNlaz          136   --------------------------------------------------------------------------------
HsApoA1          98   --------------------------------------------------------------------------------
HsApoA2          70   --------------------------------------------------------------------------------
HsApoA4         266   --------------------------------------------------------------------------------
HsApoA5         256   --------------------------------------------------------------------------------
HsApoB48       1313   LETVRTPALHFKSVGFHLPSREFQVPTFTI--------------------------------------------------
HsApoB52       1496   ILPVYDKSLWDFLKLDVTTSIGRRQHLRVS--------------------------------------------------
HsApoC1          97   --------------------------------------------------------------------------------
HsApoC2          63   --------------------------------------------------------------------------------
HsApoC3          81   --------------------------------------------------------------------------------
HsApoC4          90   --------------------------------------------------------------------------------
HsApoD          237   --------------------------------------------------------------------------------
HsApoE2         242   --------------------------------------------------------------------------------
HsApoF          206   --------------------------------------------------------------------------------
HsApoH          232   --------------------------------------------------------------------------------
HsApoJ          308   --------------------------------------------------------------------------------
HsApoL1         254   --------------------------------------------------------------------------------
HsApoL2         195   --------------------------------------------------------------------------------
HsApoL3         195   --------------------------------------------------------------------------------
HsApoL4         215   --------------------------------------------------------------------------------
HsApoL5         226   --------------------------------------------------------------------------------
HsApoL6         156   --------------------------------------------------------------------------------
HsApoM          133   --------------------------------------------------------------------------------
HsApoO          156   --------------------------------------------------------------------------------
HsApoOL         158   --------------------------------------------------------------------------------
HsMTTP          611   --------------------------------------------------------------------------------
DmMtp           612   --------------------------------------------------------------------------------
```

```
DmApoLppI      1483   --------------------------------------------------------------------------GTERVA
DmApoLppII      454   --------------------------------------------------------------------------------
DmApoLTPI      2224   KRNLSSSGQYYMTDTNFNSDLSFQWLGGNYDQQPKIIHSNLQWKAEPLHRGDREHRTIALTVAHPLLEKDINCKATYYRG
DmApoLTPII      507   --------------------------------------------------------------------------------
DmCG31659       133   --------------------------------------------------------------------------------
DmCvD           929   --------------------------------------------------------------------------------
DmFabp          110   --------------------------------------------------------------------------------
DmGlaz          124   --------------------------------------------------------------------------------
DmMic26-27      164   --------------------------------------------------------------------------------
DmNlaz          136   --------------------------------------------------------------------------------
HsApoA1          98   --------------------------------------------------------------------------------
HsApoA2          70   --------------------------------------------------------------------------------
HsApoA4         266   --------------------------------------------------------------------------------
HsApoA5         256   --------------------------------------------------------------------------------
HsApoB48       1343   --------------------------------------------------------------------------PKLYQL
HsApoB52       1526   --------------------------------------------------------------------------TAFVYT
HsApoC1          97   --------------------------------------------------------------------------------
HsApoC2          63   --------------------------------------------------------------------------------
HsApoC3          81   --------------------------------------------------------------------------------
HsApoC4          90   --------------------------------------------------------------------------------
HsApoD          237   --------------------------------------------------------------------------------
HsApoE2         242   --------------------------------------------------------------------------------
HsApoF          206   --------------------------------------------------------------------------------
HsApoH          232   --------------------------------------------------------------------------------
HsApoJ          308   --------------------------------------------------------------------------------
HsApoL1         254   --------------------------------------------------------------------------------
HsApoL2         195   --------------------------------------------------------------------------------
HsApoL3         195   --------------------------------------------------------------------------------
HsApoL4         215   --------------------------------------------------------------------------------
HsApoL5         226   --------------------------------------------------------------------------------
HsApoL6         156   --------------------------------------------------------------------------------
HsApoM          133   --------------------------------------------------------------------------------
HsApoO          156   --------------------------------------------------------------------------------
HsApoOL         158   --------------------------------------------------------------------------------
HsMTTP          611   --------------------------------------------------------------------------------
DmMtp           612   --------------------------------------------------------------------------------

DmApoLppI      1489   FNANGKLKFEHPTIRPLSISGQLNGDVNQQIASAEVIFDIFRLPEQKVVGNSELRNSRSQNGFNIAYITTVKSAGLQFQY
DmApoLppII      454   --------------------------------------------------------------------------------
DmApoLTPI      2304   LRDLLRTHLTIDYSEYPDQLIELGAQLTDRYSELGHTNYTFHVYGKHIASELDVQLNGTLAAMNSYYKTESTAHYKRDIF
DmApoLTPII      507   --------------------------------------------------------------------------------
DmCG31659       133   --------------------------------------------------------------------------------
DmCvD           929   --------------------------------------------------------------------------------
DmFabp          110   --------------------------------------------------------------------------------
DmGlaz          124   --------------------------------------------------------------------------------
DmMic26-27      164   --------------------------------------------------------------------------------
DmNlaz          136   --------------------------------------------------------------------------------
HsApoA1          98   --------------------------------------------------------------------------------
HsApoA2          70   --------------------------------------------------------------------------------
HsApoA4         266   --------------------------------------------------------------------------------
HsApoA5         256   --------------------------------------------------------------------------------
HsApoB48       1349   QVPLLGVLDLSTNVYSNLYNWSASYSGGNTSTDHFSLRARYHMKADSVVDLLSYNVQGSGETTYDHKNTFTLSCDGSLRH
HsApoB52       1532   KNPNGYSFSIPVKVLADKFIIPGLKLNDLNSVLVMPTFHVPFTDLQVPSCKLDFREIQIYKKLRTSSFALNLPTLPEVKF
HsApoC1          97   --------------------------------------------------------------------------------
HsApoC2          63   --------------------------------------------------------------------------------
HsApoC3          81   --------------------------------------------------------------------------------
HsApoC4          90   --------------------------------------------------------------------------------
HsApoD          237   --------------------------------------------------------------------------------
HsApoE2         242   --------------------------------------------------------------------------------
HsApoF          206   --------------------------------------------------------------------------------
HsApoH          232   --------------------------------------------------------------------------------
HsApoJ          308   --------------------------------------------------------------------------------
HsApoL1         254   --------------------------------------------------------------------------------
HsApoL2         195   --------------------------------------------------------------------------------
HsApoL3         195   --------------------------------------------------------------------------------
HsApoL4         215   --------------------------------------------------------------------------------
HsApoL5         226   --------------------------------------------------------------------------------
HsApoL6         156   --------------------------------------------------------------------------------
HsApoM          133   --------------------------------------------------------------------------------
HsApoO          156   --------------------------------------------------------------------------------
HsApoOL         158   --------------------------------------------------------------------------------
HsMTTP          611   --------------------------------------------------------------------------------
DmMtp           612   --------------------------------------------------------------------------------
```

```
DmApoLppI      1569   QINSNAAVDIEAHEYNIGLELNNGEIDVKAISFLNKEKFEISLSESNKHIIYIVGDFSKQNHYAKLNTKVQILDKNPIEI
DmApoLppII      454   --------------------------------------------------------------------------------
DmApoLTPI      2384   PARYGKFLALLDVNKRELEYERQSPFHAVRLHLLPTIRYPIYGLNATIWDTPDTNHSGYIYMDILERYARMDFNLTEDAS
DmApoLTPII      507   --------------------------------------------------------------------------------
DmCG31659       133   --------------------------------------------------------------------------------
DmCvD           929   --------------------------------------------------------------------------------
DmFabp          110   --------------------------------------------------------------------------------
DmGlaz          124   --------------------------------------------------------------------------------
DmMic26-27      164   --------------------------------------------------------------------------------
DmNlaz          136   --------------------------------------------------------------------------------
HsApoA1          98   --------------------------------------------------------------------------------
HsApoA2          70   --------------------------------------------------------------------------------
HsApoA4         266   --------------------------------------------------------------------------------
HsApoA5         256   --------------------------------------------------------------------------------
HsApoB48       1429   KFLDSNIKFSHVEKLGNNPVSKGLLIFDASSSWGPQMSASVHLDSKKKQHLFVKEVKIDGQFRVSSFYAKGTYGLSCQRD
HsApoB52       1612   PEVDVLTKYSQPEDSLIPFFEITVPESQLTVSQFTLPKSVSDGIAALDLNAVANKIADFELPTIIVPEQTIEIPSIKFSV
HsApoC1          97   --------------------------------------------------------------------------------
HsApoC2          63   --------------------------------------------------------------------------------
HsApoC3          81   --------------------------------------------------------------------------------
HsApoC4          90   --------------------------------------------------------------------------------
HsApoD          237   --------------------------------------------------------------------------------
HsApoE2         242   --------------------------------------------------------------------------------
HsApoF          206   --------------------------------------------------------------------------------
HsApoH          232   --------------------------------------------------------------------------------
HsApoJ          308   --------------------------------------------------------------------------------
HsApoL1         254   --------------------------------------------------------------------------------
HsApoL2         195   --------------------------------------------------------------------------------
HsApoL3         195   --------------------------------------------------------------------------------
HsApoL4         215   --------------------------------------------------------------------------------
HsApoL5         226   --------------------------------------------------------------------------------
HsApoL6         156   --------------------------------------------------------------------------------
HsApoM          133   --------------------------------------------------------------------------------
HsApoO          156   --------------------------------------------------------------------------------
HsApoOL         158   --------------------------------------------------------------------------------
HsMTTP          611   --------------------------------------------------------------------------------
DmMtp           612   --------------------------------------------------------------------------------

DmApoLppI      1649   TSEVQPNSAKIILKRQDFIDGTAEVKLGKEFKVDVIGSGKQLFNGRVALDATNFLQTNYFINEDHLNGFWHIVESEINKD
DmApoLppII      454   --------------------------------------------------------------------------------
DmApoLTPI      2464   QNLQMVGYIPDTRSAFLDIWRNYEEIRVIDVSSYLKMNHSRLITGRFHWRPSIRQEVREKIQAVGKSVYSSFSEGIDFWI
DmApoLTPII      507   --------------------------------------------------------------------------------
DmCG31659       133   --------------------------------------------------------------------------------
DmCvD           929   -------------------------------------------------------------------LFSYPMTDLGVRL
DmFabp          110   --------------------------------------------------------------------------------
DmGlaz          124   --------------------------------------------------------------------------------
DmMic26-27      164   --------------------------------------------------------------------------------
DmNlaz          136   --------------------------------------------------------------------------------
HsApoA1          98   --------------------------------------------------------------------------------
HsApoA2          70   --------------------------------------------------------------------------------
HsApoA4         266   --------------------------------------------------------------------------------
HsApoA5         256   --------------------------------------------------------------------------------
HsApoB48       1509   PNTGRLNGESNLRFNSSYLQGTNQITGRYEDGTLSLTSTSDLQSGIIKNTASLKYENYELTLKSDTNGKYKNF-------
HsApoB52       1692   PAGIVIPSFQALTARFEVDSPVYNATWSASLKNKADYVETVLDSTCSSTVQFLEYELNVLGTHKIEDGTLASKTKGTFAH
HsApoC1          97   --------------------------------------------------------------------------------
HsApoC2          63   --------------------------------------------------------------------------------
HsApoC3          81   --------------------------------------------------------------------------------
HsApoC4          90   --------------------------------------------------------------------------------
HsApoD          237   --------------------------------------------------------------------------------
HsApoE2         242   --------------------------------------------------------------------------------
HsApoF          206   --------------------------------------------------------------------------------
HsApoH          232   --------------------------------------------------------------------------------
HsApoJ          308   --------------------------------------------------------------------------------
HsApoL1         254   --------------------------------------------------------------------------------
HsApoL2         195   --------------------------------------------------------------------------------
HsApoL3         195   --------------------------------------------------------------------------------
HsApoL4         215   --------------------------------------------------------------------------------
HsApoL5         226   --------------------------------------------------------------------------------
HsApoL6         156   --------------------------------------------------------------------------------
HsApoM          133   --------------------------------------------------------------------------------
HsApoO          156   --------------------------------------------------------------------------------
HsApoOL         158   --------------------------------------------------------------------------------
HsMTTP          611   --------------------------------------------------------------------------------
DmMtp           612   --------------------------------------------------------------------------------
```

```
DmApoLppI       1729   SEYISENIKERLKKSRQVTDKIVKLAKEAGPDFSKLQGKLLDYKNDIVQELEADQSIAPIIDGIRTLFKKIAGIVDDINK
DmApoLppII       454   --------------------------------------------------------------------------------
DmApoLTPI       2544   KSIYTETTESMGVVWNTAKEYNRDFIDDIGQLSVLEEDLADLRLFVNQSYEANDFYIKNVVNFTLTILDELAIRDHIESL
DmApoLTPII       507   --------------------------------------------------------------------------------
DmCG31659        133   --------------------------------------------------------------------------------
DmCvD            942   SMTTNLNELIKYRGMLLKSEFTENGFSGNMVVNALMYIFGFTQLSSIHLGHDRNFTMLMYNEKNTRIEGNFCAEDVLKTS
DmFabp           110   --------------------------------------------------------------------------------
DmGlaz           124   --------------------------------------------------------------------------------
DmMic26-27       164   --------------------------------------------------------------------------------
DmNlaz           136   --------------------------------------------------------------------------------
HsApoA1           98   --------------------------------------------------------------------------------
HsApoA2           70   --------------------------------------------------------------------------------
HsApoA4          266   --------------------------------------------------------------------------------
HsApoA5          256   --------------------------------------------------------------------------------
HsApoB48        1582   ---------------------ATSNKMDMTFSKQNALLRSEYQADYESLRFFSLLSGSLNSHGLELNADILGTDKINSGA
HsApoB52        1772   RDFSAEYEEDGKYEGLQEWEGKAHLNIKSPAFTDLHLRYQKDKKGISTSAASPAVGTVGMDMDEDDDFSKWNFYYSPQSS
HsApoC1           97   --------------------------------------------------------------------------------
HsApoC2           63   --------------------------------------------------------------------------------
HsApoC3           81   --------------------------------------------------------------------------------
HsApoC4           90   --------------------------------------------------------------------------------
HsApoD           237   --------------------------------------------------------------------------------
HsApoE2          242   --------------------------------------------------------------------------------
HsApoF           206   --------------------------------------------------------------------------------
HsApoH           232   --------------------------------------------------------------------------------
HsApoJ           308   --------------------------------------------------------------------------------
HsApoL1          254   --------------------------------------------------------------------------------
HsApoL2          195   --------------------------------------------------------------------------------
HsApoL3          195   --------------------------------------------------------------------------------
HsApoL4          215   --------------------------------------------------------------------------------
HsApoL5          226   --------------------------------------------------------------------------------
HsApoL6          156   --------------------------------------------------------------------------------
HsApoM           133   --------------------------------------------------------------------------------
HsApoO           156   --------------------------------------------------------------------------------
HsApoOL          158   --------------------------------------------------------------------------------
HsMTTP           611   --------------------------------------------------------------------------------
DmMtp            612   ----------------------------------------APAFNETLLSTQEVYQGILKRGSVEFLLHAGRSQASSFKL

DmApoLppI       1809   AISEILEKAQKSIVDIYDKLQALWKDSLLKAWEDFIITVQKLISTLKTEFIKICTQSFKDLLSALEKYGPALKNYGKAIG
DmApoLppII       454   ---------------------CKPNTKREEERIVYILKGLGNAKSLSGNTVAA---------------------------
DmApoLTPI       2624   PKIFSELWQAMGDSGKALRNSIVWLIETIKTTYNNLLDAVARFFHGESLVYISGLLEKGIAKYDSFIKDLHIKFIKYIEN
DmApoLTPII       507   ---------------------EFLNLYNLFKGERRTRERMVILLKGLGNIGVV---------------------------
DmCG31659        133   ----------------------------------------------RFMCFDA---------------------------
DmCvD           1022   DMKGKQIGLTLEHTDHMNENHAADALHRWNITLDVLASTKSNWFKLTGQVQRN---------------------------
DmFabp           110   --------------------------------------------NELITLIPH---------------------------
DmGlaz           124   ---------------------------------------TTRFPDVIARLLPG---------------------------
DmMic26-27       164   ------------------------------------------EENCRVVLYEG---------------------------
DmNlaz           136   ----------------------------------------------VYSCTSV---------------------------
HsApoA1           98   --------------------------------------------NGGARLAEY---------------------------
HsApoA2           70   ------------------------------------------------QLTPL---------------------------
HsApoA4          266   --------------------------RLAPLAEDVRGNLRGNTEGLQKSLAEL---------------------------
HsApoA5          256   -------------------------------ELSRAFAGTGTEEGAGPDPQML---------------------------
HsApoB48        1641   HKATLRIGQDGISTSATTNLKCSLLVLENELNAELGLSGASMKLTTNGRFREH---------------------------
HsApoB52        1852   PDKKLTIFKTELRVRESDEETQIKVNWEEEAASGLLTSLKDNVPKATGVLYDYVNKYH----------------------
HsApoC1           97   --------------------------------------------PGEADNIPL---------------------------
HsApoC2           63   --------------------------------------------------------------------------------
HsApoC3           81   --------------------------------------------------------------------------------
HsApoC4           90   --------------------------------------------------------------------------------
HsApoD           237   ------------------------------------------------QLTPL---------------------------
HsApoE2          242   -------------------------------------RTRDRLDEVKEQVAEV---------------------------
HsApoF           206   ------------------------------CLGKARERGRDGAIDLGYDLLMT---------------------------
HsApoH           232   ----------------------------FGCHDGYSLDGPEEIECTKLGNWSA---------------------------
HsApoJ           308   ---------------------------ILSVDCSTNNPSQAKLRRELDESLQV---------------------------
HsApoL1          254   -------------------------------VREFLGENISNFLSLAGNTYQL---------------------------
HsApoL2          195   -------------------------------MKEFVGGNTPNVLTLVDNWYQV---------------------------
HsApoL3          195   -------------------------------FKEVMRDITPNLLSLLNNYYEA---------------------------
HsApoL4          215   -------------------------------LRDILHDITPNVLSFALDFDEA---------------------------
HsApoL5          226   ---------------------------AGFCVNKCVKAIQGIKDLHAYQMAKS---------------------------
HsApoL6          156   -------------------------------DEEEKADYVTAAGKIIYNLRNT---------------------------
HsApoM           133   --------------------------------------------------------------------------------
HsApoO           156   --------------------------------------------------IVF---------------------------
HsApoOL          158   ---------------------------------------VIIAKVTAKKVYAT---------------------------
HsMTTP           611   ----------SGSSSAYTGYIERSPRSASTYSLDILYSGSGILRRSNLNIFQY---------------------------
DmMtp            652   GIYTAGLGSLVGDGDSGDGNDAIPADDEFSEDEAVTAGMEISVQGAQLRPLVFF--------------------------
```

```
DmApoLppI       1889   EIVKPINDAAQEVIKIVVNAAE----------------------------------------------------------
DmApoLppII       486   -----LSECASTGRSNRIRVAA----------------------------------------------------------
DmApoLTPI       2704   LWHKTWTLAENHWKAVLKRFEPHLFKMISFIETTAWNLSKEVFDFIYKRTNELAESPYFNKVSSFTADAERLYRDFKAND
DmApoLTPII       539   -----SSAFAEQLQWIIREDEA----------------------------------------------------------
DmCG31659        140   -----SKIFSFHWAVIQTRKRL----------------------------------------------------------
DmCvD           1075   -----SKDDEDDWKACTKLTYE----------------------------------------------------------
DmFabp           119   -----LTPSQHGSLRLRVPCQP----------------------------------------------------------
DmGlaz           138   ---------SGKYQVLYTDYEN----------------------------------------------------------
DmMic26-27       175   -----RKIFAVAYNFIKGVKPG----------------------------------------------------------
DmNlaz           143   -----TPLANFKIVWILTRQRE----------------------------------------------------------
HsApoA1          107   -----HAKATEHLSTLSEKAKP----------------------------------------------------------
HsApoA2           75   -----IKKAGTELVNFLSYFVE----------------------------------------------------------
HsApoA4          293   -----GGHLDQQVEEFRRRVEP----------------------------------------------------------
HsApoA5          278   -----SEEVRQRLQAFRQDTYL----------------------------------------------------------
HsApoB48        1694   -----NAKFSLDGKAALTELSL----------------------------------------------------------
HsApoB52        1910   -----WEHTGLTLREVSSKLRR----------------------------------------------------------
HsApoC1          106   -----VTQLDLKGVVFRDISES----------------------------------------------------------
HsApoC2           63   -----LPAVDEKLRDLYSKSTA----------------------------------------------------------
HsApoC3           81   -----SEFWDLDPEVRPTSAVA----------------------------------------------------------
HsApoC4           90   -----LRDLGPLTKAWFLESKD----------------------------------------------------------
HsApoD           242   -----IKKAGTELVNFLSYFVE----------------------------------------------------------
HsApoE2          258   -----RAKLEEQAQQIRLQAEA----------------------------------------------------------
HsApoF           229   -----MAGMSGGPMGLAISAAL----------------------------------------------------------
HsApoH           257   -----MPSCKASCKVPVKKATV----------------------------------------------------------
HsApoJ           334   -----AERLTRKYNELLKSYQW----------------------------------------------------------
HsApoL1          276   -----TRGIGKDIRALRRARAN----------------------------------------------------------
HsApoL2          217   -----TQGIGRNIRAIRRARAN----------------------------------------------------------
HsApoL3          217   -----TQTIGSEIRAIRQARAR----------------------------------------------------------
HsApoL4          237   -----TKMIANDVHTLRRSKAT----------------------------------------------------------
HsApoL5          252   -----NSGFMAMVKNFVAKRHI----------------------------------------------------------
HsApoL6          178   -----LKYAKKNVRAFWKLRAN----------------------------------------------------------
HsApoM           133   -----LNETGQGYQRFLLYNRS----------------------------------------------------------
HsApoO           159   -----AQVSGERLYDWGLRGYI----------------------------------------------------------
HsApoOL          172   -----SQQIFGAVKSLWTKSSK----------------------------------------------------------
HsMTTP           654   -----IGKAGLHGSQVVIEAQG----------------------------------------------------------
DmMtp            706   -----SGQTELMGHVWGGSASD----------------------------------------------------------

DmApoLppI       1911   -----------------GVTHEFKQYVASLPSFESIRNEFNDKVKVLKLFEKATELTNSLFDQINILPQTPETSEFLQKL
DmApoLppII       503   -----------------L--------------------------------------------------------------
DmApoLTPI       2784   AITNIKKYSTIAWNFVKEKYFKLVPFGAELNEVLTEIWQEIKELEKIDQVQIMVQKYYEVMAKVDWVADELQLEHRLHQV
DmApoLTPII       556   -----------------P--------------------------------------------------------------
DmCG31659        157   -----------------P--------------------------------------------------------------
DmCvD           1092   -----------------PLVFTKRPHTLNGDVVFGLATEESECPEKGSTVQFAARAGPSEHARAFLRSDKISLTDTDFCP
DmFabp           136   -----------------V--------------------------------------------------------------
DmGlaz           151   -----------------F--------------------------------------------------------------
DmMic26-27       192   -----------------E--------------------------------------------------------------
DmNlaz           160   -----------------P--------------------------------------------------------------
HsApoA1          124   -----------------A--------------------------------------------------------------
HsApoA2           92   -----------------L--------------------------------------------------------------
HsApoA4          310   -----------------Y--------------------------------------------------------------
HsApoA5          295   -----------------Q--------------------------------------------------------------
HsApoB48        1711   -----------------GSAYQAMILGVDSKNIFNFKVSQEGLKLSNDMMGSYAEMKFDHTNSLNIAGLSLDFSSKLDNI
HsApoB52        1927   -----------------N-----------------------------------------------------------LQN
HsApoC1          123   -----------------E--------------------------------------------------------------
HsApoC2           80   -----------------A--------------------------------------------------------------
HsApoC3           98   -----------------A--------------------------------------------------------------
HsApoC4          107   -----------------S--------------------------------------------------------------
HsApoD           259   -----------------L--------------------------------------------------------------
HsApoE2          275   -----------------F--------------------------------------------------------------
HsApoF           246   -----------------K--------------------------------------------------------------
HsApoH           274   -----------------V--------------------------------------------------------------
HsApoJ           351   -----------------K--------------------------------------------------------------
HsApoL1          293   -----------------L--------------------------------------------------------------
HsApoL2          234   -----------------P--------------------------------------------------------------
HsApoL3          234   -----------------A--------------------------------------------------------------
HsApoL4          254   -----------------V--------------------------------------------------------------
HsApoL5          269   -----------------P--------------------------------------------------------------
HsApoL6          195   -----------------P--------------------------------------------------------------
HsApoM           150   -----------------P--------------------------------------------------------------
HsApoO           176   -----------------V--------------------------------------------------------------
HsApoOL          189   -----------------E--------------------------------------------------------------
HsMTTP           671   -----------------L--------------------------------------------------------------
DmMtp            723   -----------------S--------------------------------------------------------------
```

```
DmApoLppI       1974   HDYLIAKLKQEHIDNEKYIEELGQLLIKAVRSIWVSIRSTYPGSSDHVIDFQSWIGSLTHSFDSLA--------------
DmApoLppII       504   --------------------------------------------------------------------------------
DmApoLTPI       2864   YGLVRNKFRNYAMNALETADMYREAKTKFVFDPEVGIIDLEQKLPMSWHAFNETPRFEEIPEYQVLAKAQSFFSETNSSI
DmApoLTPII       557   -------------------------------------------------------------------------VDIRLHG
DmCG31659        158   --------------------------------------------------------------------------------
DmCvD           1155   KEVLKFSPIPTSRYCKRSNFENFTSITQYDMDLKFDNMPAWFELWSNRLDHLVSALSADKVDSLHMSQEINISMQTPQDQ
DmFabp           137   --------------------------------------------------------------------------------
DmGlaz           152   --------------------------------------------------------------------------------
DmMic26-27       193   --------------------------------------------------------------------------------
DmNlaz           161   --------------------------------------------------------------------------------
HsApoA1          125   --------------------------------------------------------------------------------
HsApoA2           93   --------------------------------------------------------------------------------
HsApoA4          311   --------------------------------------------------------------------------------
HsApoA5          296   --------------------------------------------------------------------------------
HsApoB48        1774   YSSDKFYKQTVNLQLQPYSLVTTLNSDLKYNALDLTNNGKLRLEPLKLHVAGNLKGAYQNNEIKHI--------------
HsApoB52        1931   NAEWVYQGAIRQIDDIDVRFQKAASGTTGTYQEWKDKAQNLYQELLTQEGQASFQGLKDNVFDGLVRVTQEFHMKVKHLI
HsApoC1          124   --------------------------------------------------------------------------------
HsApoC2           81   --------------------------------------------------------------------------------
HsApoC3           99   --------------------------------------------------------------------------------
HsApoC4          108   --------------------------------------------------------------------------------
HsApoD           260   --------------------------------------------------------------------------------
HsApoE2          276   --------------------------------------------------------------------------------
HsApoF           247   --------------------------------------------------------------------------------
HsApoH           275   --------------------------------------------------------------------------------
HsApoJ           352   --------------------------------------------------------------------------------
HsApoL1          294   --------------------------------------------------------------------------------
HsApoL2          235   --------------------------------------------------------------------------------
HsApoL3          235   --------------------------------------------------------------------------------
HsApoL4          255   --------------------------------------------------------------------------------
HsApoL5          270   --------------------------------------------------------------------------------
HsApoL6          196   --------------------------------------------------------------------------------
HsApoM           151   --------------------------------------------------------------------------------
HsApoO           177   --------------------------------------------------------------------------------
HsApoOL          190   --------------------------------------------------------------------------------
HsMTTP           672   --------------------EALIAATPDEGEENLDSYAGMSAILFDVQLRPVTFFNGYSDLMSKMLSASGD--------
DmMtp            724   --------------------------------------------------------------------------------

DmApoLppI       2040   VLPSILSFRSSILNCLLNENWDVVFNKKLLYSWIFFNDFELRGHVVDGKHIFTFDGLNFAYPGNCKYILAQDSVDNNFTI
DmApoLppII       504   HAFSKVKCEETLQSKSLELLKNRNEDSELRIEAYLSAISCPNAEVANQISEIVNS-------------------------
DmApoLTPI       2944   VMKLYNMRTHLDPKTWLPPYYSRALLIDSRHYMTFDQRYVGLNLNFDELGNGRSTSQCSYLLAHDFFKRNFTLLLEPASK
DmApoLTPII       564   ILAFRRVDCARHRSYFLDNYGNYTLNSELRIYSYLQAMRCPDYISVGVIKSILEH-------------------------
DmCG31659        158   --------------------------------------------------------------------------------
DmCvD           1235   FRLAVEVNGVKWRFHQIPFFYKLDSKFDASHELTFDSGLKRSCSVINGIVNTFDD-------------------------
DmFabp           137   --------------------------------------------------------------------------------
DmGlaz           152   --------------------------------------------------------------------------------
DmMic26-27       193   --------------------------------------------------------------------------------
DmNlaz           161   --------------------------------------------------------------------------------
HsApoA1          125   --------------------------------------------------------------------------------
HsApoA2           93   --------------------------------------------------------------------------------
HsApoA4          311   --------------------------------------------------------------------------------
HsApoA5          296   --------------------------------------------------------------------------------
HsApoB48        1840   YAISSAALSASYKADTVAKVQGVEFSHRLNTDIAGLASAIDMSTNYNSDSLHFSN-VFRSVMAPFTMTIDAHTNGNGKLA
HsApoB52        2011   DSLIDFLNFPRFQFPGKPGIYTREELCTMFIREVGTVLSQVYSKVHNGSEILFSYFQDLVITLPFELRKHKLIDVISMYR
HsApoC1          124   --------------------------------------------------------------------------------
HsApoC2           81   --------------------------------------------------------------------------------
HsApoC3           99   --------------------------------------------------------------------------------
HsApoC4          108   --------------------------------------------------------------------------------
HsApoD           260   --------------------------------------------------------------------------------
HsApoE2          276   --------------------------------------------------------------------------------
HsApoF           247   --------------------------------------------------------------------------------
HsApoH           275   ---------------------------------------------YQGERVKIQE-------------------------
HsApoJ           352   --------------------------------------------------------------------------------
HsApoL1          294   -----------------QSVPHASASRPRVTEPISAESGEQVERVNEPSILEMSR-------------------------
HsApoL2          235   -------------------QLGAYAPPPHVIGRISAEGGEQVERVVEGPAQAMSR-------------------------
HsApoL3          235   -------------------------RLPVTTWRISAGSGGQAERTIAGTTRAVSR-------------------------
HsApoL4          255   -------------------------GRPLIAWRYVPINVVETLRTRGAPTRIVRK-------------------------
HsApoL5          270   -----------------------------------FWTARGVQRAFEGTTLAMTN-------------------------
HsApoL6          196   -------------------RLANATKRLLTTGQVSSRSRVQVQKAFAGTTLAMTK-------------------------
HsApoM           151   --------------------------------------------------------------------------------
HsApoO           177   --------------------------------------------------------------------------------
HsApoOL          190   --------------------------------------------------------------------------------
HsMTTP           724   ---------------PISVVKGLILLIDHSQELQLQSGLKANIEVQGGLAIDISG-------------------------
DmMtp            724   ----------------TPAYQATTLSQDNEHYIILTSGATLHWRVLGARSVDLNG-------------------------
```

```
DmApoLppI      2120   IGQLTNGKLKSITLIDREGSYFEVADNLALKLNGNLVEYPQHLSGLHAWRRFYTIHLYSEYGVGIVCTSDLKVCHINING
DmApoLppII      559   -----------------------------------------ETVNQVGGFISSNLKAIRDSTDVSRDQQKYHLANIRVTK
DmApoLTPI      3024   SLAGQGLTRKLSFIANGQLIEIDLETDHISINGNPQPILPLKLGDVNIHRDLDVLSITSDTEFSLHCNVQFDLCWFEVSG
DmApoLTPII      619   -----------------------------------------EEINQVGSFVWSHLTNLAKSNSPVRIEAQGLLLNDELSE
DmCG31659       158   ------------------------------------------------------------------------STQVIHMA
DmCvD          1290   -----------------------------------------------YLINLREIAVRPDCLTLLVADCSPLPQIAVFVT
DmFabp          137   -------------------------------------------------------------------------------P
DmGlaz          152   --------------------------------------------------------------AILWSCGSIGSLGHSDQI
DmMic26-27      193   -------------------------------------------------------------DVPVVPFPTSLEDLKYMAS
DmNlaz          161   --------------------------------------------------------SAEAVDAARKILEDNDVSQAFLID
HsApoA1         125   --------------------------------------------------------------------------------
HsApoA2          93   --------------------------------------------------------------------------------
HsApoA4         311   --------------------------------------------------------GENFNKALVQQMEQLRQKLGPHAG
HsApoA5         296   -----------------------------------------------------------IAAFTRAIDQETEEVQQQLAP
HsApoB48       1919   LWGEHTGQLYSKFLLKAEPLAFTFSHDYKGSTSHHLVSRKSISAALEHKVSALLTPAEQTGTWKLKTQFNNNEYSQDLDA
HsApoB52       2091   ELLKDLSKEAQEVFKAIQSLKTTEVLRNLQDLLQFIFQLIEDNIKQLKEMKFTYLINYIQDEINTIFSDYIPYVFKLLKE
HsApoC1         124   --------------------------------------------------------------------------------
HsApoC2          81   --------------------------------------------------------------------------MSTYTG
HsApoC3          99   --------------------------------------------------------------------------------
HsApoC4         108   -----------------------------------------------------------------------------LLK
HsApoD          260   --------------------------------------------------------------------------------
HsApoE2         276   -------------------------------------------------------------------QARLKSWFEPLVE
HsApoF          247   -------------------------------------------------------------PALRSGVQQLIQYYQDQKD
HsApoH          285   ----------------------------------------------------------KFKNGMLHGDKVSFFCKNKEKK
HsApoJ          352   ---------------------------------------------------------MLNTSSLLEQLNEQFNWVSRLAN
HsApoL1         332   -------------------------------------------------------------GVKLTDVAPVSFFLVLDVV
HsApoL2         271   -------------------------------------------------------------GTMIVGAATGGILLLLDVV
HsApoL3         265   -------------------------------------------------------------GARILSATTSGIFLALDVV
HsApoL4         285   -------------------------------------------------------------VARNLGKATSGVLVVLDVV
HsApoL5         290   -------------------------------------------------------------GAWVMGAAGAGFLLMKDMS
HsApoL6         232   -------------------------------------------------------------NARVLGGVMSAFSLGYDLA
HsApoM          151   -------------------------------------------------------------------HPPEKCVEEFKSL
HsApoO          177   ------------------------------------------------------------------------------IE
HsApoOL         190   --------------------------------------------------------------ESLPKPKEKTKLGSSSEI
HsMTTP          764   --------------------------------------------------------AMEFSLWYRESKTRVKNRVTVVIT
DmMtp           763   ------------------------------------------KVGFSLWNRNAQTEIQQNTGSAVLGHLAVGFTYAKLVQ

DmApoLppI      2200   FYTSKTR---GLLGNGNAEPYDDFLLIDG----------------------TLAENSAALGNDYGVGKCTAIEFDNNQFK
DmApoLppII      598   TFPVDYR---RYSFNNEVSYKLESLGVGA----------------------STDYQIIYSQHGFLPRSSRINVTTEFFGT
DmApoLTPI      3104   WYFGRTA---GLLGTLNNEPYDEYTMSSG-------------VISNETQLFTDSWSLKQCRQNKLAQTQEVSQEVSDACT
DmApoLTPII      658   RFKMDIR---KFSRNYEHSLFFDEYNFGT----------------------TTDANVIFGTDSYLPRIASVNFTADLFGQ
DmCG31659       166   QYFGKSA---GLVIGDMSKVPQESCPYDT---------------------------------------------------
DmCvD          1323   PSPVQGL---STNYGLRVHIGQNYFNFRA----------------------RTDNSSLPTDEPVLIYLNQDQTPHNVRKK
DmFabp          138   PTSVDDG---ARSGGCGQRPGGPG--------------------------------------------------------
DmGlaz          170   WILGRDR---DFEVDIRSKVYDVLKRLSL----------------------DPERLIISKNKQCPEAL------------
DmMic26-27      212   DLYDEAK---DLIFPKKK--------------------------------------------------------------
DmNlaz          185   TVQKNCP---RLDGNGTGLAGEDGLDVDD----------------------FVSTTVPNAIEKA?EWLRRLYERLYDIFM
HsApoA1         125   -----LE---DLRQGLLPVLESFKVSFLS----------------------ALEEYTKKLNTQ-----------------
HsApoA2          93   --------------GTQPATQ-----------------------------------------------------------
HsApoA4         335   DVEGHLS---FLEKDLRDKVNSFFSTFKE----------------------KESQDKTLSLPELEQQQEQQQEQQQEQVQ
HsApoA5         317   PPPGHSA---FAPEFQQTDSGKVLSKLQA----------------------RLDDLWEDITHSLHDQGHSHLGDP-----
HsApoB48       1999   YNTKDKI---GVELTGRTLADLTLLDSPI----------------------KVPLLLSEPINIIDALEMRDAVEKPQEFT
HsApoB52       2171   NLCLNLHKFNEFIQNELQEASQELQQIHQYIMALREEYFDPSIVGWTVKYYELEEKIVSLIKNLLVALKDFHSEYIVSAS
HsApoC1         124   --------------GETQD-------------------------------------------------------------
HsApoC2          87   IFTDQVL---SVLKGEE---------------------------------------------------------------
HsApoC3          99   --------------------------------------------------------------------------------
HsApoC4         111   KTHSLCP---RLVCGDKDQG------------------------------------------------------------
HsApoD          260   --------------GTQPATQ-----------------------------------------------------------
HsApoE2         289   DMQRQWA---GLVEKVQAAVGTSAAPVPS----------------------DNH--------------------------
HsApoF          266   ANISQPE---TTKEGLRAISDVSDLEETT----------------------TLASFISEVVSSAPYWGWAIIKSYDLDPG
HsApoH          307   CSYTEDA---QCIDGTIEVPKCFKEHSSL----------------------AFWKTDASDVKPC----------------
HsApoJ          375   LTQGEDQ---YYLRVTTVASHTSDSDVPS----------------------GVTEVVVKLFDSDPITVTVPVEVSRKNPK
HsApoL1         351   YLVYESK---HLHEGAKSETAEELKKVAQ----------------------ELEEKLNILNNNYKILQADQEL-------
HsApoL2         290   SLAYESK---HLLEGAKSESAEELKKRAQ----------------------ELEGKLNFLTKIHEMLQPGQDQ-------
HsApoL3         284   NLVYESK---HLHEGAKSASAEELRRQAQ----------------------ELEENLMELTQIYQRLNPCHTH-------
HsApoL4         304   NLVQDSL---DLHKGEKSESAELLRQWAQ----------------------ELEENLNELTHIHQSLKAG----------
HsApoL5         309   SFLQSWK---HLEDGARTETAEELRALAK----------------------KLEQELDRLTQHHRHLPQKASQTCSSSRG
HsApoL6         251   TLSKEWK---HLKEGARTKFAEELRAKAL----------------------ELERKLTELTQLYKSLQQKVRSRARGVGK
HsApoM          164   TSCLDSK---AFLLTPRNQEACELSNN-----------------------------------------------------
HsApoO          179   DLWKENF---QKPGNVKNSPGTK---------------------------------------------------------
HsApoOL         208   EVPAKTT---HVLKHSVPLPTELSSEAKT----------------------KSESTSGATQFMPDPKLMDHGQSHPEDID
HsMTTP          788   TDITVDS---SFVKAGLETSTETEAGLEF----------------------ISTVQFSQYPFLVCMQMDKDEAPFRQFEK
DmMtp           801   DFSITHEPKLSLNADLDFYSGIKLCMQLQ----------------------RPEQLLKQTNVRSVFLQSVDRPYAKHVRS
```

```
DmApoLppI       2255   SSKRQEMCSELFGIESTLAFNFITLDSRPYRKACDIALAKVAEKEKEATACTFALAYGSAVKQINKWVLLPPRCIKCAGP
DmApoLppII       653   NYNVFEASVRQENVEDVLEYYLGPKGLVNKDFDEIVKLIEVGNNGVAAGGRARR--------------------------
DmApoLTPI       3168   SFFRTGILATCSAVLDPTPFYEMCMDLGMKSPPIRKGHPAVKGACAAALAYIEACTALKVPMRVPSQCVFCQLSNGSYVP
DmApoLTPII       713   SVNFFEFTARAEGLEELAANAFGPKGPLSGQLLRKKLSFLNRWLGNESAEEDDTLENLLSLDNLRLK-------------
DmCG31659        192   --------------------------------------------------------------------------------
DmCvD           1378   PYQWPIETSDYDFRVELNEQNILIVECTQLSSTIQFDLYNILNFEIYGVYKHQMCGLCSKPLNRMQNYTICELEANTPTP
DmFabp           159   --------------------------------------------------------------------------------
DmGlaz           213   --------------------------------------------------------------------------------
DmMic26-27       227   --------------------------------------------------------------------------------
DmNlaz           240   NFLSY---------------------------------------------------------------------------
HsApoA1          158   --------------------------------------------------------------------------------
HsApoA2          100   --------------------------------------------------------------------------------
HsApoA4          390   MLAPLES-------------------------------------------------------------------------
HsApoA5          367   --------------------------------------------------------------------------------
HsApoB48        2054   IVAFVKYDKNQDVHSINLPFFETLQEYFERNRQTIIVVLENVQRNLKHINIDQFVRKYRAALGKLPQQANDYLNSFNWER
HsApoB52        2251   NFTSQLSSQVEQFLHRNIQEYLSILTDPDGKGKEKIAELSATAQEIIKSQAIATKKIISDYHQQFRYKLQDFSDQLSDYY
HsApoC1          129   --------------------------------------------------------------------------------
HsApoC2          101   --------------------------------------------------------------------------------
HsApoC3           99   --------------------------------------------------------------------------------
HsApoC4          128   --------------------------------------------------------------------------------
HsApoD           266   --------------------------------------------------------------------------------
HsApoE2          318   --------------------------------------------------------------------------------
HsApoF           321   AGSLEI--------------------------------------------------------------------------
HsApoH           346   --------------------------------------------------------------------------------
HsApoJ           430   FMETVAEKALQEYRKKHREE------------------------------------------------------------
HsApoL1          399   --------------------------------------------------------------------------------
HsApoL2          338   --------------------------------------------------------------------------------
HsApoL3          332   --------------------------------------------------------------------------------
HsApoL4          349   --------------------------------------------------------------------------------
HsApoL5          364   RAVRGSRVVKPEGSRSPLPWPVVEHQPRLGPGVALRTPKRTVSAPRMLGHQPAPPAPARKGRQAPGRHRQ----------
HsApoL6          306   DLTGTCETEAYWKELREHVWMWLWLCVCLCVCVYVQFT------------------------------------------
HsApoM           188   --------------------------------------------------------------------------------
HsApoO           199   --------------------------------------------------------------------------------
HsApoOL          263   MYSTRS--------------------------------------------------------------------------
HsMTTP           843   KYERLSTGRGYVSQKRKESVLAGCEFPLHQENSEMCKVVFAPQPDSTSSGWF----------------------------
DmMtp            859   TLSHKTAGCTFALNQKNNEMCNLIFRDL----------------------------------------------------

DmApoLppI       2335   AGQHDFGDEFTVKLPNNKVDVVFVVDINVTPGVLSNLIAPAINDIRESLRSRGFSDVQVGVIVFEETKRYPALLTSDGGK
DmApoLppII       707   --------------------------------------------------------------------------------
DmApoLTPI       3248   EGTFMELSGPEIPKSSDVVFIVEAKECNANLKTSKNIMTVVSSIEEQLQAAKITNNRYAVVAFGGVSPYDKARSVIYEHN
DmApoLTPII       780   --------------------------------------------------------------------------------
DmCG31659        192   --------------------------------------------------------------------------------
DmCvD           1458   VPLQNSSDVVVVA-------------------------------------------------------------------
DmFabp           159   --------------------------------------------------------------------------------
DmGlaz           213   --------------------------------------------------------------------------------
DmMic26-27       227   --------------------------------------------------------------------------------
DmNlaz           245   --------------------------------------------------------------------------------
HsApoA1          158   --------------------------------------------------------------------------------
HsApoA2          100   --------------------------------------------------------------------------------
HsApoA4          397   --------------------------------------------------------------------------------
HsApoA5          367   --------------------------------------------------------------------------------
HsApoB48        2134   QVSHAKEKLTALTKKYRITENDIQIALDDAKINFNEKLSQLQTYMIQ---------------------------------
HsApoB52        2331   EKFIAESKRLIDLSIQNYHTFLIYITELLKKLQSTTVMNPYMKLAPGELTIIL---------------------------
HsApoC1          129   --------------------------------------------------------------------------------
HsApoC2          101   --------------------------------------------------------------------------------
HsApoC3           99   --------------------------------------------------------------------------------
HsApoC4          128   --------------------------------------------------------------------------------
HsApoD           266   --------------------------------------------------------------------------------
HsApoE2          318   --------------------------------------------------------------------------------
HsApoF           327   --------------------------------------------------------------------------------
HsApoH           346   --------------------------------------------------------------------------------
HsApoJ           450   --------------------------------------------------------------------------------
HsApoL1          399   --------------------------------------------------------------------------------
HsApoL2          338   --------------------------------------------------------------------------------
HsApoL3          332   --------------------------------------------------------------------------------
HsApoL4          349   --------------------------------------------------------------------------------
HsApoL5          434   --------------------------------------------------------------------------------
HsApoL6          344   --------------------------------------------------------------------------------
HsApoM           188   --------------------------------------------------------------------------------
HsApoO           199   --------------------------------------------------------------------------------
HsApoOL          269   --------------------------------------------------------------------------------
HsMTTP           895   --------------------------------------------------------------------------------
DmMtp            887   --------------------------------------------------------------------------------
```

```
DmApoLppI       2415   INYKGNVADVKLAGIKSFCDNCVEQIITEKRILDIYNSLKEIVKGIAPQADEKAFQLALDYPFRAGAAKSIIGVRSDSLE
DmApoLppII       707   --------------------------------------------------------------------------------
DmApoLTPI       3328   EFTSKPEQLADYFGHINTGNGSSNDILMAISAAAKLNFRPGVSKTFILLSCSKCAARDMRFDYTSILQYLLEEGVNLHIL
DmApoLTPII       780   --------------------------------------------------------------------------------
DmCG31659        192   --------------------------------------------------------------------------------
DmCvD           1471   --------------------------------------------------------------------------------
DmFabp           159   --------------------------------------------------------------------------------
DmGlaz           213   --------------------------------------------------------------------------------
DmMic26-27       227   --------------------------------------------------------------------------------
DmNlaz           245   --------------------------------------------------------------------------------
HsApoA1          158   --------------------------------------------------------------------------------
HsApoA2          100   --------------------------------------------------------------------------------
HsApoA4          397   --------------------------------------------------------------------------------
HsApoA5          367   --------------------------------------------------------------------------------
HsApoB48        2181   --------------------------------------------------------------------------------
HsApoB52        2384   --------------------------------------------------------------------------------
HsApoC1          129   --------------------------------------------------------------------------------
HsApoC2          101   --------------------------------------------------------------------------------
HsApoC3           99   --------------------------------------------------------------------------------
HsApoC4          128   --------------------------------------------------------------------------------
HsApoD           266   --------------------------------------------------------------------------------
HsApoE2          318   --------------------------------------------------------------------------------
HsApoF           327   --------------------------------------------------------------------------------
HsApoH           346   --------------------------------------------------------------------------------
HsApoJ           450   --------------------------------------------------------------------------------
HsApoL1          399   --------------------------------------------------------------------------------
HsApoL2          338   --------------------------------------------------------------------------------
HsApoL3          332   --------------------------------------------------------------------------------
HsApoL4          349   --------------------------------------------------------------------------------
HsApoL5          434   --------------------------------------------------------------------------------
HsApoL6          344   --------------------------------------------------------------------------------
HsApoM           188   --------------------------------------------------------------------------------
HsApoO           199   --------------------------------------------------------------------------------
HsApoOL          269   --------------------------------------------------------------------------------
HsMTTP           895   --------------------------------------------------------------------------------
DmMtp            887   --------------------------------------------------------------------------------

DmApoLppI       2495   YKNWWKFVRAQLTGSITKFDGALIHLIAPVKGLSLEGVLSEKLIGFNSRLVATVDGKDSKKRTKLQFDNDMGIDFVLNNG
DmApoLppII       707   --------------------------------------------------------------------------------
DmApoLTPI       3408   ADTEFDFERNKKLRHFFGLDSKLVYSKRFPEGDAETRNTTHIPKSNLGICTTLAVETQGSVFSARKLQPERKYPIKRFAT
DmApoLTPII       780   --------------------------------------------------------------------------------
DmCG31659        192   --------------------------------------------------------------------------------
DmCvD           1471   --------------------------------------------------------------------------------
DmFabp           159   --------------------------------------------------------------------------------
DmGlaz           213   --------------------------------------------------------------------------------
DmMic26-27       227   --------------------------------------------------------------------------------
DmNlaz           245   --------------------------------------------------------------------------------
HsApoA1          158   --------------------------------------------------------------------------------
HsApoA2          100   --------------------------------------------------------------------------------
HsApoA4          397   --------------------------------------------------------------------------------
HsApoA5          367   --------------------------------------------------------------------------------
HsApoB48        2181   --------------------------------------------------------------------------------
HsApoB52        2384   --------------------------------------------------------------------------------
HsApoC1          129   --------------------------------------------------------------------------------
HsApoC2          101   --------------------------------------------------------------------------------
HsApoC3           99   --------------------------------------------------------------------------------
HsApoC4          128   --------------------------------------------------------------------------------
HsApoD           266   --------------------------------------------------------------------------------
HsApoE2          318   --------------------------------------------------------------------------------
HsApoF           327   --------------------------------------------------------------------------------
HsApoH           346   --------------------------------------------------------------------------------
HsApoJ           450   --------------------------------------------------------------------------------
HsApoL1          399   --------------------------------------------------------------------------------
HsApoL2          338   --------------------------------------------------------------------------------
HsApoL3          332   --------------------------------------------------------------------------------
HsApoL4          349   --------------------------------------------------------------------------------
HsApoL5          434   --------------------------------------------------------------------------------
HsApoL6          344   --------------------------------------------------------------------------------
HsApoM           188   --------------------------------------------------------------------------------
HsApoO           199   --------------------------------------------------------------------------------
HsApoOL          269   --------------------------------------------------------------------------------
HsMTTP           895   --------------------------------------------------------------------------------
DmMtp            887   --------------------------------------------------------------------------------
```

```
DmApoLppI       2575   GWVFATQNFEKLKASDQKKMLNQITSSLADTLFKTEIVSDCRCLPIHGLHGQHKCVIKSSTFVANKKAKSA
DmApoLppII       707   -----------------------------------------------------------------------
DmApoLTPI       3488   IFAKRVALSATPIQSQTCECSAHNTGVSYMACSPQALPEEKYDLDDYDSFNNWDWGDEPESETNVMS----
DmApoLTPII       780   -----------------------------------------------------------------------
DmCG31659        192   -----------------------------------------------------------------------
DmCvD           1471   -----------------------------------------------------------------------
DmFabp           159   -----------------------------------------------------------------------
DmGlaz           213   -----------------------------------------------------------------------
DmMic26-27       227   -----------------------------------------------------------------------
DmNlaz           245   -----------------------------------------------------------------------
HsApoA1          158   -----------------------------------------------------------------------
HsApoA2          100   -----------------------------------------------------------------------
HsApoA4          397   -----------------------------------------------------------------------
HsApoA5          367   -----------------------------------------------------------------------
HsApoB48        2181   -----------------------------------------------------------------------
HsApoB52        2384   -----------------------------------------------------------------------
HsApoC1          129   -----------------------------------------------------------------------
HsApoC2          101   -----------------------------------------------------------------------
HsApoC3           99   -----------------------------------------------------------------------
HsApoC4          128   -----------------------------------------------------------------------
HsApoD           266   -----------------------------------------------------------------------
HsApoE2          318   -----------------------------------------------------------------------
HsApoF           327   -----------------------------------------------------------------------
HsApoH           346   -----------------------------------------------------------------------
HsApoJ           450   -----------------------------------------------------------------------
HsApoL1          399   -----------------------------------------------------------------------
HsApoL2          338   -----------------------------------------------------------------------
HsApoL3          332   -----------------------------------------------------------------------
HsApoL4          349   -----------------------------------------------------------------------
HsApoL5          434   -----------------------------------------------------------------------
HsApoL6          344   -----------------------------------------------------------------------
HsApoM           188   -----------------------------------------------------------------------
HsApoO           199   -----------------------------------------------------------------------
HsApoOL          269   -----------------------------------------------------------------------
HsMTTP           895   -----------------------------------------------------------------------
DmMtp            887   -----------------------------------------------------------------------
```

Fig. S11. Alignment of 14 nematode and 11 fly sequences with Vit1-6, ApoLpp and ApoLTP separated plus both outgroups.

```
CeApoL1           0  --------------------------------------------------------------------------------
CeApoL3-Like      0  --------------------------------------------------------------------------------
CeMoma1           0  --------------------------------------------------------------------------------
CeVit1I           0  --------------------------------------------------------------------------------
CeVit1II          0  --------------------------------------------------------------------------------
CeVit2I           0  --------------------------------------------------------------------------------
CeVit2II          0  --------------------------------------------------------------------------------
CeVit3I           0  --------------------------------------------------------------------------------
CeVit3II          0  --------------------------------------------------------------------------------
CeVit4I           0  --------------------------------------------------------------------------------
CeVit4II          0  --------------------------------------------------------------------------------
CeVit5I           0  --------------------------------------------------------------------------------
CeVit5II          0  --------------------------------------------------------------------------------
CeVit6I           0  --------------------------------------------------------------------------------
CeVit6II          0  --------------------------------------------------------------------------------
DmApoLppI         1  SIVDDVSKISKKYKMYGVKNVQDLNLDVSLKLFGSELAFLSLGDNIPSSLDDIINYFSTSFEKAKQELSSFEKQFSSHHL
DmApoLppII        0  --------------------------------------------------------------------------------
DmApoLTPI         1  -----------------------------------------------------------------------------RKA
DmApoLTPII        0  --------------------------------------------------------------------------------
DmCG31659         0  --------------------------------------------------------------------------------
DmCvD             0  --------------------------------------------------------------------------------
DmFabp            0  --------------------------------------------------------------------------------
DmGlaz            0  --------------------------------------------------------------------------------
DmMic26-27        0  --------------------------------------------------------------------------------
DmNlaz            0  --------------------------------------------------------------------------------
DmMtp             0  --------------------------------------------------------------------------------
CeDSc4            0  --------------------------------------------------------------------------------

CeApoL1           0  --------------------------------------------------------------------------------
CeApoL3-Like      0  --------------------------------------------------------------------------------
CeMoma1           0  --------------------------------------------------------------------------------
CeVit1I           1  ---------------------------------------------------------RIQTGITL----------LKEL-
CeVit1II          1  ---------------------------------------------------------------------------MRSI-
CeVit2I           1  ---------------------------------------------------------RIQTGIKL----------LKEL-
CeVit2II          1  ---------------------------------------------------------------------------MRSI-
CeVit3I           1  ---------------------------------------------------------RVQSGIQM----------LQEI-
CeVit3II          1  ---------------------------------------------------------------------------MKSI-
CeVit4I           1  ---------------------------------------------------------RVQSGIQM----------LQEI-
CeVit4II          1  ---------------------------------------------------------------------------MKSI-
CeVit5I           1  ---------------------------------------------------------RVQSGIQM----------LQEI-
CeVit5II          1  ---------------------------------------------------------------------------MKSI-
CeVit6I           1  ---------------------------------------------------------RASGSFQ-----------PTEF-
CeVit6II          1  ---------------------------------------------------------------------------MKFF-
DmApoLppI        81  FLDTDLAYPTSIGVPLELVAQGFAATKVDLAVSLDINAILEQNWQKAKYRLKFVPSVDINANVQIGFNAQVLSTGLRVVS
DmApoLppII        1  -------------------------------------------------------MARMKYNIA-----------LIGI-
DmApoLTPI         4  QEQARASEEEVDEDYDFEESLEGNKRQKRDVSTTRKQEIDRNVDSLGYKLKYDYNNPRAQFGLRVFGNDLRYFN-VESL-
DmApoLTPII        1  -----------------------------------------------------MGGLKPQAAIWL----------LL---
DmCG31659         0  --------------------------------------------------------------------------------
DmCvD             1  -------------------------------------------------------MRLLKCSVFVYLFLIFDAGHAFSII
DmFabp            0  --------------------------------------------------------------------------------
DmGlaz            0  --------------------------------------------------------------------------------
DmMic26-27        0  --------------------------------------------------------------------------------
DmNlaz            0  --------------------------------------------------------------------------------
DmMtp             1  ---------------------------------------------------------------------------MENKN
CeDSc4            1  ---------------------------------------------------------MFSSRIWL---------------

CeApoL1           0  --------------------------------------------------------------------------------
CeApoL3-Like      0  --------------------------------------------------------------------------------
CeMoma1           0  --------------------------------------------------------------------------------
CeVit1I          13  ---------------------------------ALK----------MNIRAR--PANYNEKDA-----------------
CeVit1II          5  ---------------------------------IIA----------SIVALA--IA------F-----------------
CeVit2I          13  ---------------------------------AQK----------MNIRAR--PATYTEKDA-----------------
CeVit2II          5  ---------------------------------IIA----------SLVALA--LA------S-----------------
CeVit3I          13  ---------------------------------VKK----------MNIRPR--VQQTDSQNA-----------------
CeVit3II          5  ---------------------------------IIA----------SLVALA--IA------A-----------------
CeVit4I          13  ---------------------------------VKK----------MNIRPR--VQQTDSQNA-----------------
CeVit4II          5  ---------------------------------IIA----------SLVALA--IA------A-----------------
CeVit5I          13  ---------------------------------VKK----------MNIRPR--VQQTDSQNA-----------------
CeVit5II          5  ---------------------------------IIA----------SLVALA--IA------A-----------------
CeVit6I          12  ---------------------------------LSN----------LLEKLRITRRQSSEQDP-----------------
CeVit6II          5  ---------------------------------IAL----------ALLGAALASTHLDRYNSI----------------
DmApoLppI       161  SAHSATGSDITVAVISDGEGFNVDLELPREKLELIN----------FNVDTELYVAEQDKQKAIALKGNKKNKNSQPSEI
DmApoLppII       14  ---------------------------------LAS----------VLLTIAVNAENACNLGCP----------------
DmApoLTPI        82  ---------------------------------VEV----------MALAAKFNPFQQAKNVL-----------------
DmApoLTPII       15  ---------------------------------LIA----------HTQAVRENPLKDPRICG-----------------
DmCG31659         0  ---------------------------------MIK--------------------------------------------
DmCvD            26  GLNKQMLYEYEGNVLVGAKPQDEGHQAPPTTGWIVRGKLTLQRQSELVLAAALVIDDVTLNNS-----------------
DmFabp            0  --------------------------------------------------------------------------------
DmGlaz            0  --------------------------------------------------------------------------------
DmMic26-27        0  --------------------------------------------------------------------------------
DmNlaz            0  --------------------------------------------------------------------------------
DmMtp             6  KK-------------------------------CLR----------TLLLLALFLGLLEDGKT-----------------
CeDSc4            9  ---------------------------------LLA----------VTVGVCLAVPDLDEIKK-----------------
```

```
CeApoL1          0  --------------------------------------------------------------------------------
CeApoL3-Like     0  --------------------------------------------------------------------------------
CeMoma1          0  --------------------------------------------------------------------------------
CeVit1I         31  --------------------------------------------------------------------------------
CeVit1II        17  --------------------------------------------------------------------------------
CeVit2I         31  --------------------------------------------------------------------------------
CeVit2II        17  --------------------------------------------------------------------------------
CeVit3I         31  --------------------------------------------------------------------------------
CeVit3II        17  --------------------------------------------------------------------------------
CeVit4I         31  --------------------------------------------------------------------------------
CeVit4II        17  --------------------------------------------------------------------------------
CeVit5I         31  --------------------------------------------------------------------------------
CeVit5II        17  --------------------------------------------------------------------------------
CeVit6I         32  --------------------------------------------------------------------------------
CeVit6II        26  --------------------------------------------------------------------------------
DmApoLppI      231  CFNQLELVGLNICIKSSTSLSEVQAGNGNVAERGLSVSEKFHLSRPFNFAVYLTTERKFTFKGIHTQEAFSQKWKLDYST
DmApoLppII      35  --------------------------------------------------------------------------------
DmApoLTPI      102  --------------------------------------------------------------------------------
DmApoLTPII      35  --------------------------------------------------------------------------------
DmCG31659        3  --------------------------------------------------------------------------------
DmCvD           89  ------------------------------------------------------GEKFLQNKEMYPPYKPFKIALTKDGA
DmFabp           0  --------------------------------------------------------------------------------
DmGlaz           0  --------------------------------------------------------------------------------
DmMic26-27       0  --------------------------------------------------------------------------------
DmNlaz           0  --------------------------------------------------------------------------------
DmMtp           28  --------------------------------------------------------------------------------
CeDSc4          29  --------------------------------------------------------------------------------

CeApoL1          0  --------------------------------------------------------------------------------
CeApoL3-Like     0  --------------------------MQIKDD------------------------------------------------
CeMoma1          0  ----------------------------MTQD------------------------------------------------
CeVit1I         31  ---------FAMVYLRYKDMDY----AILPVD------------------------------------------------
CeVit1II        17  ---------SPAFERTFEPKID----YHYKFD------------------------------------------------
CeVit2I         31  ---------FAMVYLRYKDMDY----AFLPID------------------------------------------------
CeVit2II        17  ---------SPAFERTFEPKTD----YHYKFD------------------------------------------------
CeVit3I         31  ---------HAVFYLRYKEMDY----IVLPID------------------------------------------------
CeVit3II        17  ---------SPALDRTFSPKSE----YVYKFD------------------------------------------------
CeVit4I         31  ---------HAVFYLRYKEMDY----IVLPID------------------------------------------------
CeVit4II        17  ---------SPALDRTFSPKSE----YVYKFD------------------------------------------------
CeVit5I         31  ---------HAVFYLRYKEMDY----IVLPID------------------------------------------------
CeVit5II        17  ---------SPALDRTFSPKSE----YVYKFD------------------------------------------------
CeVit6I         32  ---------HAFVYIRHRDMDY----AFLPID------------------------------------------------
CeVit6II        26  --------ERNIQESSFRAGRE----YRYLFN------------------------------------------------
DmApoLppI      311  PGSKVSHDTTVVYELGNKPKTF----SRLSFDNSQCHFAVEGGINNDKNELVVYGQYEQDKEIKKSKIGFSKNGNEYKPL
DmApoLppII      35  --------KSDNGLLKYIPGNY----YDYSFD------------------------------------------------
DmApoLTPI      102  ---------SGKEFTYTKSRVF----LDASYT------------------------------------------------
DmApoLTPII      35  ---------RPQCDSKSAKFNYGEQLYKYQYT------------------------------------------------
DmCG31659        3  -------------WKIFVPA------ILYLQS------------------------------------------------
DmCvD          115  ISHVVFKEGDPIWSMNFKRAIA----SVLQFQ------------------------------------------------
DmFabp           0  --------------MSFVGKKY----------------------------------------------------------
DmGlaz           0  -------------MMSGQPLGS----RVWLLS------------------------------------------------
DmMic26-27       0  --------------------------------------------------------------------------------
DmNlaz           0  --------------------------MNHHSS------------------------------------------------
DmMtp           28  --------------ALIAPNSQ----QIFKLQ------------------------------------------------
CeDSc4          29  -----NLRKHGPDYYKNQPKMNENTVRLLKVD------------------------------------------------

CeApoL1          0  --------------------------------------------------------------------------------
CeApoL3-Like     6  --------------------------------------------------------------------------------
CeMoma1          4  --------------------------------------------------------------------------------
CeVit1I         50  --------------------------------------------------------------------------------
CeVit1II        36  --------------------------------------------------------------------------------
CeVit2I         50  --------------------------------------------------------------------------------
CeVit2II        36  --------------------------------------------------------------------------------
CeVit3I         50  --------------------------------------------------------------------------------
CeVit3II        36  --------------------------------------------------------------------------------
CeVit4I         50  --------------------------------------------------------------------------------
CeVit4II        36  --------------------------------------------------------------------------------
CeVit5I         50  --------------------------------------------------------------------------------
CeVit5II        36  --------------------------------------------------------------------------------
CeVit6I         51  --------------------------------------------------------------------------------
CeVit6II        46  --------------------------------------------------------------------------------
DmApoLppI      387  IEIQDNNGISNSINGYHADGKIVVKKNSNNIERYNFENFQVSNSNNAHVAVNGWSDVGTNSLTSELRISLDHQTFLIKEN
DmApoLppII      55  --------------------------------------------------------------------------------
DmApoLTPI      121  --------------------------------------------------------------------------------
DmApoLTPII      58  --------------------------------------------------------------------------------
DmCG31659       16  --------------------------------------------------------------------------------
DmCvD          143  --------------------------------------------------------------------------------
DmFabp           8  --------------------------------------------------------------------------------
DmGlaz          15  --------------------------------------------------------------------------------
DmMic26-27       0  --------------------------------------------------------------------------------
DmNlaz           6  --------------------------------------------------------------------------------
DmMtp           42  --------------------------------------------------------------------------------
CeDSc4          56  --------------------------------------------------------------------------------
```

```
CeApoL1            0  -----MSIYESLKSTLMNGEE-----------------------------------------------------------
CeApoL3-Like       6  --------HDDAG.QSEDNGVPIYDSLESLLFIP----------------------------------------------
CeMoma1            4  -----KP.V.TI--------------------------------------------------------------------
CeVit1I           50  -----TQLI.K.IEKYISNGKVQFSEIRRLLNQE---------------------HEFETHHAAYFYEAI----------
CeVit1II          36  -----GLVLSG.PTASSELSQSRFSARVRIQAVD---------------------DRHIHLQLVNIHMAA----------
CeVit2I           50  -----RQLV.N.IEKFTSNGKVQFSEIRRLLNQE---------------------LEFETHHAAYFYEAI----------
CeVit2II          36  -----GLVLSG.P.ASSELSQSRISARARIQAVD---------------------DRYIHLQLVNIRMAA----------
CeVit3I           50  -----.ETIDNVVEKYVRNG.FDIKSLLTFLTND---------------------SKFELHRALFFYEAE----------
CeVit3II          36  -----GLLLSG.PT.FSDASQTLISCRTRLQAVD---------------------DRYIHLQLIDIQYSA----------
CeVit4I           50  -----.ETIDNVVEKYVRNG.FDIKSLLTFLTND---------------------SKFELHRALFFYEAE----------
CeVit4II          36  -----GLLLSG.PT.FSDASQTLISCRTRLQAVD---------------------DRYIHLQLIDIQYSA----------
CeVit5I           50  -----.ETIDT.VEKYVRNG.FDIKSLLTFLTND---------------------SKFELHRALFFYEAE----------
CeVit5II          36  -----GLLLSG.PTASSDASQTLISCRTRLQAVD---------------------DRYIHLQLTDIQYSA----------
CeVit6I           51  -----ADSIPEVVRSMIQ.GRLEIGDIERVLAQG---------------------IHFSASNAAFLYETV----------
CeVit6II          46  -----GQLSAG.PVPSTPQGISRLQSQVTLQWTD---------------------GNTVRMQLQKTRFATSQ--------
DmApoLppI        467  LKLENGLYEAGFFINDEHSP.NIYGSSIHLTIADQSYALKTNGKAAAWSIGSDGSFNFQKLADSNSARAGSLVENVEIQY
DmApoLppII        55  -----SILTIGAS.DVP.DSD----------------------------------DTSLKVSGSAKIFAK----------
DmApoLTPI        121  -----VPLAVG.PLAIHAFGASSIDLRVSGNLDEMDPPTDWHFDVEGQFKPSVSVDVITTMQTDMFWEQSGIKVKSNLYS
DmApoLTPII        58  -----VAVRTEFAGSGD.SSDLLLKSDLEIFFPKPC-------------------EGYLRINDAKLYDTL----------
DmCG31659         16  -----SMAMRAF--------------------------------------------------------------------
DmCvD            143  MKSSGAFVVDE.GIHGTCRT.YFVSNRTNYISIR---------------------KTPEVKTCKPYSEAVHTTRS-----
DmFabp             8  --------------------------------------------------------------------------------
DmGlaz            15  -----GVLLVTFAG.DAY.-------------------------------------------------------------
DmMic26-27         0  --------------------------------------------------------------------------------
DmNlaz             6  --------------------------------------------------------------------------------
DmMtp             42  -----NQVILQELGRDSSSA.TSYTFETDLKINSV--------------------WSGDEDQLLEVFISG----------
CeDSc4            56  YWFRTE.MIYDDIDNKEKDPSTVIAGNFSFETLH---------------------HDVEGGMLGRFTLT-----------

CeApoL1           16  --------------------------------------------------------------------------------
CeApoL3-Like      32  --------------------------------------------------------------------------------
CeMoma1           11  --------------------------------------------------------------------------------
CeVit1I           94  --------------------------------------------------------------------------------
CeVit1II          80  --------------------------------------------------------------------------------
CeVit2I           94  --------------------------------------------------------------------------------
CeVit2II          80  --------------------------------------------------------------------------------
CeVit3I           94  --------------------------------------------------------------------------------
CeVit3II          80  --------------------------------------------------------------------------------
CeVit4I           94  --------------------------------------------------------------------------------
CeVit4II          80  --------------------------------------------------------------------------------
CeVit5I           94  --------------------------------------------------------------------------------
CeVit5II          80  --------------------------------------------------------------------------------
CeVit6I           95  --------------------------------------------------------------------------------
CeVit6II          92  --------------------------------------------------------------------------------
DmApoLppI        547  KNKQVGGIKIMSNFDVNKMDVDVEISREQKIGSIIVKYESNQRHAQDYSLEASAKINKHSIDVISKCDFNGNVYVVDNSL
DmApoLppII        86  --------------------------------------------------------------------------------
DmApoLTPI        196  NSELVAKLKVRGRNLVSFSFDLPRDKNEIFSVRSELLVQKREEQLPQAGIANRSANSTCTWPVLDQAIGLQMCSHYSVPD
DmApoLTPII       104  --------------------------------------------------------------------------------
DmCG31659         23  --------------------------------------------------------------------------------
DmCvD            197  --------------------------------------------------------------------------------
DmFabp             8  --------------------------------------------------------------------------------
DmGlaz            29  --------------------------------------------------------------------------------
DmMic26-27         0  --------------------------------------------------------------------------------
DmNlaz             6  --------------------------------------------------------------------------------
DmMtp             87  --------------------------------------------------------------------------------
CeDSc4           104  --------------------------------------------------------------------------------

CeApoL1           16  --------------------------------------------------------------------------------
CeApoL3-Like      32  --------------------------------------------------------------------------------
CeMoma1           11  --------------------------------------------------------------------------------
CeVit1I           94  -------------------------------------------------------------------RKFPTTLGLPLIV
CeVit1II          80  -------------------------------------------------------------------SHLPESEQIPSLN
CeVit2I           94  -------------------------------------------------------------------RKFPTTLGLPLTI
CeVit2II          80  -------------------------------------------------------------------SHLPESEQMPSLN
CeVit3I           94  -------------------------------------------------------------------RRIPTTIGMPLTI
CeVit3II          80  -------------------------------------------------------------------SHIPQSEQWPKIK
CeVit4I           94  -------------------------------------------------------------------RRIPTTIGMPLTI
CeVit4II          80  -------------------------------------------------------------------SHIPQSEQWPKIE
CeVit5I           94  -------------------------------------------------------------------RRIPTTIGMPLTI
CeVit5II          80  -------------------------------------------------------------------SHIPQSEQWPKIE
CeVit6I           95  -------------------------------------------------------------------RRVPTPMGLPVQF
CeVit6II          92  -------------------------------------------------------------------QESNSMKMLPFER
DmApoLppI        627  VTSWGTLLSAKGEIGQRYSAQDININIQGNVQISGKDKVTQWILKVIGTPDKTNSDFRISRDTSELIKLTSESQHPQDKI
DmApoLppII        86  -------------------------------------------------------------------GNCGYTLQLSSVK
DmApoLTPI        276  LSNATEIYPSLLLAGPLNFSLILKKSDLSAKKYVFEYKWDQQEEDNNFSLVFTTPGSKVPRVLVANVTKVPDAFNASVAF
DmApoLTPII       104  -----------------------------------------------DELNDNESDSSEKTKEYDYYDNLANEESESQNY
DmCG31659         23  --------------------------------------------------------------------------------
DmCvD            197  -------------------------------------------------------------------NVPPNTCEFDHQK
DmFabp             8  --------------------------------------------------------------------------------
DmGlaz            29  --------------------------------------------------------------------------------
DmMic26-27         0  --------------------------------------------------------------------------------
DmNlaz             6  --------------------------------------------------------------------------------
DmMtp             87  -------------------------------------------------------------------SKVDA--------
CeDSc4           104  -------------------------------------------------------------------QCNTDNCGNPSPI
```

```
CeApoL1          16  --------------------------------------------------------------------------------
CeApoL3-Like     32  -------------------------------------------------------------------------RSMSKEL
CeMoma1          11  ---------------------------------------------------------------------SNAGEQVTNVF
CeVit1I         107  ---------------------------------------------------------------------SGKIPTVFSAE
CeVit1II         93  ---------------------------------------------------------------------SMEQRELSEEY
CeVit2I         107  ---------------------------------------------------------------------SGKIPTVISAE
CeVit2II         93  ---------------------------------------------------------------------SMEQRELSEEY
CeVit3I         107  ---------------------------------------------------------------------SGKMPTILSIN
CeVit3II         93  ---------------------------------------------------------------------SLEQRELSDEL
CeVit4I         107  ---------------------------------------------------------------------SGKMPTILSIN
CeVit4II         93  ---------------------------------------------------------------------SLEQRELSDEL
CeVit5I         107  ---------------------------------------------------------------------SGKMPTILSIN
CeVit5II         93  ---------------------------------------------------------------------SLEQRELSDEF
CeVit6I         108  ---------------------------------------------------------------------TSKMPTISSIR
CeVit6II        105  ---------------------------------------------------------------------FEEVERMNREH
DmApoLppI       707  SFAKLNLIVKNQLTAKGEFRVAKNGKGDF----------------------------------------TASIDTLKTEP
DmApoLppII       99  V--------------------------------------------------------------------TNTKESVEKKI
DmApoLTPI       356  VNGPNRVSAGCSYDGNPDFRRLDIYLDTNGNRSLDLGMELRRHQDFTAWIYNPRMLLAINGVNITGLAGTVKVNEKNGIK
DmApoLTPII      137  ---------------------------------------------------------------------DNMHPKSSDFN
DmCG31659        23  ---------------------------------------------------------------------HGACPSNMTAV
DmCvD           210  SVIIGNEAIYGMSPHNE----------------------------------------------------TGYYLSMAHAK
DmFabp            8  ---------------------------------------------------------------------KLDKSENFDEY
DmGlaz           29  ---------------------------------------------------------------------FGRCPNYP---
DmMic26-27        0  ---------------------------------------------------------------------MLRKTATMGIM
DmNlaz            6  ---------------------------------------------------------------------SHLLLLISVVF
DmMtp            92  ---------------------------------------------------------------------SGKARSITRIP
CeDSc4          117  ---------------------------------------------------------------------YIAFRQGGNNA

CeApoL1          16  ---------------------MPKELQVKAWGAQWH--------------------------------------------
CeApoL3-Like     39  SET---ALRTQFL-------------------------------------------------------------------
CeMoma1          22  GQF-----------------------------------------------------------------------------
CeVit1I         118  GQF---SLGLEETEL------RLTVEARPSVA.THV--------------------------------------------
CeVit1II        104  KQMLKLPLRAQLRNG------LIA...FDKED.E.S--------------------------------------------
CeVit2I         118  GQF---SLELEGTEL------RLTVEARPSVA.THV--------------------------------------------
CeVit2II        104  KQMLELPLRAQLRNG------LIS...FDKED.E.S--------------------------------------------
CeVit3I         118  GKV---SIELEKLGA------RLVLDI.PTVATTHV--------------------------------------------
CeVit3II        104  KELLELPFRAQIRNG------LVS.I.FSSED.E.S--------------------------------------------
CeVit4I         118  GKV---SIELEKLGA------RLVLDI.PTVATTHV--------------------------------------------
CeVit4II        104  KELLELPFRAQIRNG------LVS.I.FSSED.E.S--------------------------------------------
CeVit5I         118  GKV---SIELEKLGA------RLVLDI.PTVATTHV--------------------------------------------
CeVit5II        104  KELLELPFRAQIRNG------LIS.I.FSSED.E.S--------------------------------------------
CeVit6I         119  GQV---TFELEPKNGKSFDGLRLRVQAGPRVASTHV--------------------------------------------
CeVit6II        116  QELLSMPVEFDYEHG------LVR.IRFAENDQP.S--------------------------------------------
DmApoLppI       747  KHKLEIESKFHIQSPKYDIDASLTLDGKRKVHLKSENTIEKLKFSTKNIGEANDKIIAFEANGSLKGELRGNGEIQGTFI
DmApoLppII      111  LNSIQKPVQFTLVSG-----ILEPQICSDSSDLDYS--------------------------------------------
DmApoLTPI       436  QHDVDLSFETKKLQA------VI.GNV.QSEITTSTNMTIKYRFQANKIEEINFAGKLVNNGDKSKTEYRGNMKLQTSAY
DmApoLTPII      148  VDLTKNLLRFAFHDG------LIS.VCPQEQETP.V--------------------------------------------
DmCG31659        34  GDL-----------------------------------------------------------------------------
DmCvD           238  GTTLIHTFESTGEAQFINSELLLNF.NETPIDNPID--------------------------------------------
DmFabp           19  MKELGVGLVTRKMGN------SLSP-------------------------------------------------------
DmGlaz           37  --------------------------------------------------------------------------------
DmMic26-27       11  AAV-----------------------------------------------------------------------------
DmNlaz           17  GAV-------------------------------.V--------------------------------------------
DmMtp           103  DR----PFYISLVRG------Q.DKVIAHTSKD.SL--------------------------------------------
CeDSc4          128  EHILKASDESDATWN-----FLYAIVNTIYTP.EYG--------------------------------------------

CeApoL1          31  ------------------------------------------------------------LDRWSSNRQEIMNRME----
CeApoL3-Like     49  ------------------------------------------------------------..K..N..RK.VR-------
CeMoma1          25  -----------------------------------------------------------WQLVT.K.-------------
CeVit1I         145  ------------------------YEMR------MFTPLFEQGVKSVQSVRAYTPIKIQAVVGMKR.-F..VYKVVVPEN
CeVit1II        134  ------------------------KNMK------------------RAVVNMISFNPIAPRNEIEKI-ESSYDKE.QSEE
CeVit2I         145  ------------------------YEMR------MFTPLFEQGVKSVQSVRAYTPIKIQAVAGMKR.-F..VYKVVVPEN
CeVit2II        134  ------------------------KNMK------------------RAVVNMISFNPIAPRNEIEKI-ESSYDKE.QSEE
CeVit3I         145  ------------------------TEMR------FWYPVIEQGVKSLQSARLHTPLRFESTVELKK.TL..THKFVVPEN
CeVit3II        134  ------------------------KNAK------------------RSILNLFSLRKSAPV.EM.QD-.----KDMESDK
CeVit4I         145  ------------------------TEMR------FWYPVIEQGVKSLQSARLHTPLRFESTVELKK.TL..THKFVVPEN
CeVit4II        134  ------------------------KNAK------------------RSILNLFSLRKSAPV.EM.QD-.----KDMESDK
CeVit5I         145  ------------------------TEMR------FWYPVIEQGVKSLQSARLHTPLRFESTVELKK.TL..THKFVVPEN
CeVit5II        134  ------------------------KNAK------------------RSILNLFSLRKSAPV.EMNQD-----QKDMESDK
CeVit6I         152  ------------------------LSLR------VICPIAEVGTKFLHQAVLNTPVDTEIRMN.EDK-VV.RAIYNTPSE
CeVit6II        146  ------------------------ENIK------------------RAVINMLQ------VNILKKEKY.GAEKSDNQEP
DmApoLppI       827  FNAPDGRVIDGSINRKISTNAK--SGLSQGNIDAQLSDTPFGSNKKRSISLIGKLDRLNTKTKEF.ANSNLVYTAFNGEK
DmApoLppII      142  ------------------------LNIK---------------------------------------------.AVVSLL
DmApoLTPI       510  PKLNFASESTWLSLQGHTEGMITYNNAPDYVNPNYTSLVRLIFARSHSEDSFLDGTQTRASLELKLP.SK.DY.ILVKHK
DmApoLTPII      178  ------------------------LNIK------------------KGILSAFQNTMMRFDVD-----------------
DmCG31659        37  --------------------------------------------------------------------------------
DmCvD           274  -------------------IETSMAAEPSNLELQRLDPNDPTGGRSPQQQETLIAQAGTL..SLAEALETTEFKFSEPYD
DmFabp           38  --------------------------------------------------------------------------------
DmGlaz           37  --------------------------------------------------------------------------------
DmMic26-27       14  --------------------------------------------------------------------------------
DmNlaz           22  --------------------------------------------------------------------------------
DmMtp           129  ------------------------LNLE------------------RGIASLLQLR----..ASQEEELDVSGLCRVSYN
CeDSc4          159  ------------------------EGDEQTVDTIYGRCFVNFG---RPEDKRFRRIIEKCDLGYGT.FTKFEGIESVQYD
```

```
CeApoL1         47  -----------EIAKD---------------------------------------------------LDGLEKA------
CeApoL3-Like    62  -----------QMEGI--------------------------------------------------------.EKLDNW
CeMoma1         33  --TTNNGDSK-P.KIE------------------------------------QLPIYAEDNA----PLKQKFL------
CeVit1I        194  QKSIISLTTR-PVVFL------------------------------------RFPGFSKFEY----IEAE.RTVVVPQW
CeVit1II       171  NTSFFT-NEK-TLEG.------------------------------------CQVAYTVIR------EQKKTIITKSIN
CeVit2I        194  QKSIVSLTTR-PVVFL------------------------------------RFPGFSKFEY----IEAE.RTVVVPQW
CeVit2II       171  NTSFFT-NEK-TLEG.------------------------------------CQVAYTVIR------EQKKTIITKSIN
CeVit3I        195  KKTTVSVHTR-PV.FI------------------------------------RVPKNQDSEY----VETE..TISHSQY
CeVit3II       167  DSLFFNVHEK-TMEG.------------------------------------CEVAYTIVQ------E.EKTIYTKSVN
CeVit4I        195  KKTTVSVHTR-PV.FI------------------------------------RVPKNQDSEY----VEAE..TISHSQY
CeVit4II       167  DSLFFNVHEK-TMEG.------------------------------------CEVAYTIVQ------E.GKTIYTKSVN
CeVit5I        195  KKTTVSVHTR-PV.FI------------------------------------RVPKNQDSEY----VEAE..TISHSQY
CeVit5II       167  DSLFFNVHEK-TMEG.------------------------------------CEVAYTIVQ------E.EKTIYTKSVN
CeVit6I        201  EKRIAMIQSR-PVTFT------------------------------------RTVAPDARQY----PEPI.MTYMLPAH
CeVit6II       178  TFSFTNV-ER-TLEGE------------------------------------CEVLYTVEEI----KKED.QRWAKSIN
DmApoLppI      905  SEISYQIKQQ-PNGDA------------------------------------KNIDFSLKAYGNPLPQPF.I.FALGDY
DmApoLppII     153  QSGIEAEHEV-DVFGM------------------------------------CPTHTSTSK------V.NANIITKARN
DmApoLTPI      590  EHIKNGTEHN-V.VGLKYTPEKEITGLFSVHLPRRNLFAIDAYMNVTVPEFNSCTASLKVNEK----ATKDYIIFINGSW
DmApoLTPII     199  ----ANTTET-DVSGQ------------------------------------CQVQYALED-----T.SSYVTIRKTKD
DmCG31659       37  -----------DMDRF-----------------------------------------------------------KGKW
DmCvD          335  STLSDVIKLLS.MDF.-----------------------------------SLTKLYREVDI----GTSYRQETIRNIF
DmFabp          38  -----------TVEVT----------------------------------------------------.E.DTYTLTTTST
DmGlaz          37  ----------------------------------------------------SMPKFNMSRV-------------LGHW
DmMic26-27      14  -----------AVKAA---------------------------------------------------PEPQKP.SSAADC
DmNlaz          22  ------AHAQVPFPGK------------------------------------CPDVKL---------..TFDAEAYMGVW
DmMtp          163  VKSSTKVEKT---KR.------------------------------------CSLWDLRVNYNPEEAL.VTQQAQETVF
CeDSc4         212  QDVWYTQNTKVDADII------------------------------------MVDAIEMLAFKSPLHEKYGFTLESRTH

CeApoL1         59  -------------------------------------------------------SAIATTVGSSVDIASGLAVFGGLF
CeApoL3-Like    74  EKGCA--------------------------------------------------------.S.A...T.G....I..I...I
CeMoma1         63  -------------------------------------------------------PEEPLPLQREFATIRIACEQE---
CeVit1I        232  QQKTQEI------------------------------------------------EKVFNFL.LE.STRGNILNQHT.E
CeVit1II       206  FDKCT--------------------------------------------------ERSEIAY.LRYSSECPECEKDTVL
CeVit2I        232  QQKTQEI------------------------------------------------EKVFNFL.LE.STRGNILNQHT.E
CeVit2II       206  FDKCT--------------------------------------------------ERSEIAY.LRYSSECPECEKDTEL
CeVit3I        233  QMSTEEI------------------------------------------------DRQYE.F.LRINAQGNVLSQWT.P
CeVit3II       203  FDKCI--------------------------------------------------TRPE.AY.LRFGSECKECEKE.Q.
CeVit4I        233  QMSTEEI------------------------------------------------DRQYE.F.LRINAQGNVLSQWT.P
CeVit4II       203  FDKCI--------------------------------------------------TRPE.AY.LRFGSECKECEKE.Q.
CeVit5I        233  QMSTEEI------------------------------------------------DRQYE.F.LRINAQGNVLSQWT.P
CeVit5II       203  FDKCI--------------------------------------------------TRPE.AY.LRFGSECKECEKE.Q.
CeVit6I        239  KQLSQSL------------------------------------------------DREYPQIRVQGTLNRPTS.RIPQW
CeVit6II       215  FDKCT--------------------------------------------------RRPYIHHVQT--PVCKDCQQTLEQ
DmApoLppI      947  SAQHAVVSITSKYGEIFSVSANGNYN-----------------------------NNQ.LEY.LQAN.EIPKSTLKS.E
DmApoLppII     189  LNSCS--------------------------------------------------HREQINS.L---VSGKVNEKA.IT
DmApoLTPI      665  FTGHSVAVKANYKDRSSRVQALHHLKMIVESPSFNITSLNIIYRRKQLLIFYDIQAKYDQDPY.LTIQY..NAHNRNTNA
DmApoLTPII     232  INSC---------------------------------------------------RQRYA.HSVLQTTPYTFRDDKTIW
DmCG31659       46  YT-----------------------------------------------------HS.YPHLSLR.----EKCQSTDFI
DmCvD          375  HEIIPRIGTK---------------------------------------------ASVFL.HHLVLNKLTKP-QIAVQL
DmFabp          56  FKT----------------------------------------------------...SFKL.VEF.EETLDGRNVKSI
DmGlaz          51  Y------------------------------------------------------EVERSFYLPEIASGCTT-------
DmMic26-27      32  SLVCR--------------------------------------------------PSELPIY..LRKTEPKPERHPPQD
DmNlaz          51  Y------------------------------------------------------EYA.YPFAFEIGKKCIY.NYSLID
DmMtp          203  YELSSEGTLLH--------------------------------------------AESQENHRLNLAAKPDVGS.VKSS
CeDSc4         255  VEITNR-------------------------------------------------TRVFV.SYCNDTVP.AKCAEQAFG

CeApoL1         83  FMPP----------------------------------------------------------------------------
CeApoL3-Like   101  L...----------------------------------------------------------------------------
CeMoma1         84  --------------------------------------------------------------------------------
CeVit1I        263  NWLL------------------------------------------AEQDFEVSVENKYRPAE----------------
CeVit1II       235  IR.Q------------------------------------------TVYTYILENEELKESEVRSL-------------
CeVit2I        263  NWLL------------------------------------------AEQDFEVSVENKNRPAE----------------
CeVit2II       235  IR.Q------------------------------------------TVYTYVLENEELKESEVRSL-------------
CeVit3I        264  MVLM------------------------------------------TEQDFEFTLENKNRPVE----------------
CeVit3II       232  VK.Q------------------------------------------TVYTYTFKNEKLQESEVHSI-------------
CeVit4I        264  MVLM------------------------------------------TEQDFEFTLENKNRPVE----------------
CeVit4II       232  VQ.Q------------------------------------------TVYTYTFKNEKLQESEVNSI-------------
CeVit5I        264  MVLM------------------------------------------TEQDFEYTLENKNRPVE----------------
CeVit5II       232  VK.Q------------------------------------------TVYTYTFKNEKLQESEVHSV-------------
CeVit6I        270  IVDS------------------------------------------NVEVYYKPNVEQYEAI-----------------
CeVit6II       242  DKMS------------------------------------------STVLNYNITGTSSSFLINSVELRSQHL------
DmApoLppI      997  INSHGKVLKSLIGNENAAYNVEFFLDSKTSLGQYARVNTVWNGTANDGSYDFEAQTNNMESPLKFNGKYHRKQTGNIKDG
DmApoLppII     215  SSLL------------------------------------------LQANYI---------------------------
DmApoLTPI      745  EVRL------------------------------------------KVKERDYWINAKLLSEQPKLLQLEIHMDKIRDV
DmApoLTPII     260  PILK------------------------------------------SQSHCNLTIDNNVYKEI----------------
DmCG31659       68  EKEE----------------------------------------------------------------------------
DmCvD          408  LI.M------------------------------------------PFHIFELSAELVQKCEDF---------------
DmFabp          83  ITLD----------------------------------------------------------------------------
DmGlaz          69  --------------------------------------------------------------------------------
DmMic26-27      61  SVLH----------------------------------------------------------------------------
DmNlaz          76  NSTV----------------------------------------------------------------------------
DmMtp          238  LILQ-------------------------------------HVSQGSEEVKQLQLGSLDKAIQSLLEWYRV--------
CeDSc4         285  AVRV----------------------------------------GGKLYEHVKIAQEQSNKLTKLI------------
```

```
CeApoL1            87  --------------------------------------------------------------------------------
CeApoL3-Like      105  --------------------------------------------------------------------------------
CeMoma1            84  --------------------------------------------------------------------------------
CeVit1I           284  --------------------------------------------------------------------------------
CeVit1II          259  --------------------------------------------------------------------------------
CeVit2I           284  --------------------------------------------------------------------------------
CeVit2II          259  --------------------------------------------------------------------------------
CeVit3I           285  --------------------------------------------------------------------------------
CeVit3II          256  --------------------------------------------------------------------------------
CeVit4I           285  --------------------------------------------------------------------------------
CeVit4II          256  --------------------------------------------------------------------------------
CeVit5I           285  --------------------------------------------------------------------------------
CeVit5II          256  --------------------------------------------------------------------------------
CeVit6I           290  --------------------------------------------------------------------------------
CeVit6II          273  --------------------------------------------------------------------------------
DmApoLppI        1077  DLTGKQTYVLNAQYGAQYVKMDASLGY-----------------------------------------------------
DmApoLppII        225  --------------------------------------------------------------------------------
DmApoLTPI         782  HIQVGLLNVDKRKELSLELKWDANRDPSQRLGLLAEYNSPGTKHYDGNLMITYPERTIHFGFNSFTGGPKYFGKVHASWS
DmApoLTPII        281  --------------------------------------------------------------------------------
DmCG31659          72  --------------------------------------------------------------------------------
DmCvD             430  --------------------------------------------------------------------------------
DmFabp             87  --------------------------------------------------------------------------------
DmGlaz             69  --------------------------------------------------------------------------------
DmMic26-27         65  --------------------------------------------------------------------------------
DmNlaz             80  --------------------------------------------------------------------------------
DmMtp             272  --------------------------------------------------------------------------------
CeDSc4            311  --------------------------------------------------------------------------------

CeApoL1            87  --------------------------------------------------------------------------------
CeApoL3-Like      105  --------------------------------------------------------------------------------
CeMoma1            84  --------------------------------------------------------------------------------
CeVit1I           284  --------------------------------------------------------------------------------
CeVit1II          259  --------------------------------------------------------------------------------
CeVit2I           284  --------------------------------------------------------------------------------
CeVit2II          259  --------------------------------------------------------------------------------
CeVit3I           285  --------------------------------------------------------------------------------
CeVit3II          256  --------------------------------------------------------------------------------
CeVit4I           285  --------------------------------------------------------------------------------
CeVit4II          256  --------------------------------------------------------------------------------
CeVit5I           285  --------------------------------------------------------------------------------
CeVit5II          256  --------------------------------------------------------------------------------
CeVit6I           290  --------------------------------------------------------------------------------
CeVit6II          273  --------------------------------------------------------------------------------
DmApoLppI        1104  --------------------------------------------------------------------------------
DmApoLppII        225  --------------------------------------------------------------------------------
DmApoLTPI         862  INEVIEFEYEAGILPGHTLHNWVKAELRTPFDGWRVNSLDAGIYSLKNLILVNSTLFWADDQKLQVGYKSDYDVNDQLMS
DmApoLTPII        281  --------------------------------------------------------------------------------
DmCG31659          72  --------------------------------------------------------------------------------
DmCvD             430  --------------------------------------------------------------------------------
DmFabp             87  --------------------------------------------------------------------------------
DmGlaz             69  --------------------------------------------------------------------------------
DmMic26-27         65  --------------------------------------------------------------------------------
DmNlaz             80  --------------------------------------------------------------------------------
DmMtp             272  --------------------------------------------------------------------------------
CeDSc4            311  --------------------------------------------------------------------------------

CeApoL1            87  ---------------------------VAIAGLIVGAASGVSNVA-----------------------TGVNKKLAT---
CeApoL3-Like      105  ---------------------------.........T......L.-----------------------..IT.FFH.---
CeMoma1            84  -----------------------YDRVAERFKVVDC.MTQTKKA.-----------------------.KC.AY.TE---
CeVit1I           284  ----FTARLTVGQLEKTELSQIKYNKIFEKEFELEQENTESRREY-FTK---MVKSIQKEQGYK----SV.SLR.EA-PR
CeVit1II          259  ----YTVNVNGQEVMKTETRSKLVLEENHSIKSHIEKVN.EKESI-IYS---SRWEQLVEDFFK----N.DKAEF.P-FE
CeVit2I           284  ----FTARLTVGQLEKTELSQIKYNKIFEKEFELEQENTESRREY-FNK---MVKNIQKEQGYK----SVISL..EA-PR
CeVit2II          259  ----YTVNVNGQEVMKTETRSKLVLEENHSIKSHIKKVN.EKESI-IYS---SRWEQLVEDFFK----N.DKAEF.P-FE
CeVit3I           285  ----FTARVTIGNLEKTDLSEIKFDKIFEKEFDLENNE.ENRRQY-FHK---MIREIQSEQGFK----NLITL..EA-PQ
CeVit3II          256  ----YTLNVNGQEVVKSETRSKVTFVEESKINREIKKV..PKEEI-VYS---MENEKLIEQFYQ----Q.DQAEVNP-FK
CeVit4I           285  ----FTARVTIGNLEKTDLSEIKFDKIFEKEFDLENNE.ENRRQY-FHK---MIREIQSEQGFK----NLITL..EA-PQ
CeVit4II          256  ----YTLNVNGQEVVKSETRAKVTFVEESKINREIKKV..PKEEI-VYS---MENEKLIEQFYK----Q.DKAEVNP-FK
CeVit5I           285  ----FTARVTIGNLEKTDLSEIKFDKIFEKEFDLENNE.ENRRQY-FHK---MIREIQSEQGFK----NLITL..EA-PQ
CeVit5II          256  ----YTLNVNGQEVVKSETRAKVTFVEESKINREIKKV..PKEEI-VYS---MENEKLIEQFYQ----Q.DKAEVNP-FK
CeVit6I           290  ----FELNLYNNYKMEKNYEKVYKKHNGRRYLEAEPEYDEEEHREQITK---KFEWLQNEKVYQ----HVAKFEIKP--E
CeVit6II          273  ----FAPISEKHQLVSAFTLNTMELIYAGEKKTEIKQVRNEKTSELVYN---QESEWAEQQWAQ----..EE.Y.RQ-LP
DmApoLppI        1104  ----GAEKVDIAYVIDSSFDSVKDIKVNIRTFKPLDDSTY.VTALFKQT---DKSYGLDTTFYHSAHKK..DIR.DL-LK
DmApoLppII        225  ----KESRIVNHLIENVQLTETYKFIGNTKRNSDIS.KVVTILKL-KNP---SGTKANSPGTGS----.VRSLIFQR-PE
DmApoLTPI         942  FDVRFGINSTIRDIPTINVKVKHWMDVKKVDTELYLGY..QNDTFNTYS---MDSSWEIEKNQRYNNYS.LVHLVSP-FK
DmApoLTPII        281  ------------KCLETHLLVPFSNASSGALTTSTSRLKLDGVES-YSA---GEFLEQNPELVE----RRATLVFDHTPA
DmCG31659          72  -------------------------NKFSVVARELNTQT.TVKMR-----------------------KADILNVEPEFG
DmCvD             430  ----LNIGPDRPDVRQAAILSFATLIHNVYVAKGIDKEKFEEY.QKYFNAYLSDRDFDQKMLYLQGLNNLQLGNV.NYLE
DmFabp             87  ------------------------------GNKLTQEQK.DKP-------------------------.TIVREFTD---
DmGlaz             69  -------------------------------FQFEPYNK.EQSKF-----------------------SNFKLAV.I---
DmMic26-27         65  -------------------------------KNLEAGVRY.REEVQ----------------------S.YKAVADQ---
DmNlaz             80  ----------------------------SVVNAAINRFT.QPSNV-----------------------..QA.V.GP---
DmMtp             272  ----FELESDVDGMISAIKEQTLEDQLK.SLTELQS.DV.K.SL.LAYVKLIPLARITRQEQFE----DLLTEHAEVLPQ
CeDSc4            311  ----GTYRRHLQDMGDSHICEKHSLLYSQ..QEARL.KRQDWEA.-IQY---PENDHVLSLIASALGGV.TAESIT.-AR
```

```
CeApoL1         114  --------------------------------------------------------------------------------
CeApoL3-Like    132  --------------------------------------------------------------------------------
CeMoma1         115  --------------------------------------------------------------------------------
CeVit1I         351  DYTMN---------------------------------------------------------------------------
CeVit1II        326  KFPLD---------------------------------------------------------------------------
CeVit2I         351  DYTMN---------------------------------------------------------------------------
CeVit2II        326  KFPLD---------------------------------------------------------------------------
CeVit3I         352  QMYWN---------------------------------------------------------------------------
CeVit3II        323  AIEME---------------------------------------------------------------------------
CeVit4I         352  QMYWN---------------------------------------------------------------------------
CeVit4II        323  AIEIE---------------------------------------------------------------------------
CeVit5I         352  QMYWN---------------------------------------------------------------------------
CeVit5II        323  AIEME---------------------------------------------------------------------------
CeVit6I         357  VVKME---------------------------------------------------------------------------
CeVit6II        341  QWTEN---------------------------------------------------------------------------
DmApoLppI      1176  EKPII---------------------------------------------------------------------------
DmApoLppII      292  TYTSK---------------------------------------------------------------------------
DmApoLTPI      1018  GYEKGGLVAHFSLSDQRVVSGAASLNFDLREFTLTMNGYVKKFTDNMLTVNITTPLEKFGTINARFGLNEKKRHAVAEVR
DmApoLTPII      341  VKPSH---------------------------------------------------------------------------
DmCG31659       104  RYVLG---------------------------------------------------------------------------
DmCvD           506  PIVQD---------------------------------------------------------------------------
DmFabp          109  --------------------------------------------------------------------------------
DmGlaz           92  --------------------------------------------------------------------------------
DmMic26-27       89  --------------------------------------------------------------------------------
DmNlaz          106  --------------------------------------------------------------------------------
DmMtp           344  LVDLL---------------------------------------------------------------------------
CeDSc4          382  EVLLT---------------------------------------------------------------------------

CeApoL1         114  -----------------------------------------------------------------------DHKIREINR
CeApoL3-Like    132  -----------------------------------------------------------------------KGQHK.VAA
CeMoma1         115  --------------------------------------------------------------------------------
CeVit1I         356  ---------------------------------------------------------------------TEVTTVCDKQV
CeVit1II        331  ---------------------------------------------------------------------KKM.L.KT.TE
CeVit2I         356  ---------------------------------------------------------------------TELTTVCDKQV
CeVit2II        331  ---------------------------------------------------------------------KKM.L.KT.TE
CeVit3I         357  ---------------------------------------------------------------------TELRTVCDKWI
CeVit3II        328  ---------------------------------------------------------------------QKVEQLD..F.
CeVit4I         357  ---------------------------------------------------------------------TELRTVCDKWI
CeVit4II        328  ---------------------------------------------------------------------QKVEQLE..F.
CeVit5I         357  ---------------------------------------------------------------------TELRTVCDKWI
CeVit5II        328  ---------------------------------------------------------------------QKVEQLQ..F.
CeVit6I         362  -----------------------------------------------------------------------VEAVCNNDF
CeVit6II        346  ----------------------------------------------------------------------KVEM.KKMFS
DmApoLppI      1181  -----------------------------------------------------ISSIAELLGDRKGKVLFEILNLADLDI
DmApoLppII      297  ----------------------------------------------------------------------NINALKT.LS
DmApoLTPI      1098  APTAALGVEVLADIKNLLNFDVKLSVATPIESFQQAAIFALFNPERVDMRGLWNNVTLGFTGVWHMQNITDFEYSYHVFT
DmApoLTPII      346  ---------------------------------------------------------------------DEIKAA..LLV
DmCG31659       109  --------------------------------------------------------------------------------
DmCvD           511  ------------------------------------------------------------------PNEHE.L.FQAAWT
DmFabp          109  --------------------------------------------------------------------------------
DmGlaz           92  ---------------------------------------------------------------------KNINR.-----
DmMic26-27       89  --------------------------------------------------------------------------------
DmNlaz          106  --------------------------------------------------------------------------------
DmMtp           349  ------------------------------------------------------------------GAVQTFDAHNATFG
CeDSc4          387  ---------------------------------------------------------------------ASPDYLDDLLF

CeApoL1         123  MLAED---------------------------------------------------------------------------
CeApoL3-Like    141  .I..----------------------------------------------------------------------------
CeMoma1         115  --------------------------------------------------------------------------------
CeVit1I         367  RMCQWEVEIRRSPILEETK-------------------------------------------------------------
CeVit1II        342  QIQ.VENNIPETSHFL----------------------------------------------------------------
CeVit2I         367  RMCQWEVEIRRSPILEETK-------------------------------------------------------------
CeVit2II        342  QIQ.VENNMPETSHFL----------------------------------------------------------------
CeVit3I         368  RMCKVEMDARRSPMEHENK-------------------------------------------------------------
CeVit3II        339  QIQ.HEQNTPETVHLI----------------------------------------------------------------
CeVit4I         368  RMCKVEMDARRSPIEHENK-------------------------------------------------------------
CeVit4II        339  QIQ.HEQNTPETVHLI----------------------------------------------------------------
CeVit5I         368  RMCKVEMDARRSPMEHENK-------------------------------------------------------------
CeVit5II        339  QIQ.HEQNTPETVHLI----------------------------------------------------------------
CeVit6I         371  HFCKTQIR------------------------------------------------------------------------
CeVit6II        356  LM.KQIEQGEAELEAAHTV-------------------------------------------------------------
DmApoLppI      1208  KINSEASYVSIDEFYIIV--------------------------------------------------------------
DmApoLppII      307  D.VDSTGDYVKKET------------------------------------------------------------------
DmApoLTPI      1178  P..GFEENGFIVQLLKRKEFVFQLHGKMSNYKLGVKINGEPKSDLVNQLGSNKMELEMLYDADFKPLNAETDYKPDADEE
DmApoLTPII      357  EMCRVGFPNIQREFIDVF--------------------------------------------------------------
DmCG31659       109  --------------------------------------------------------------------------------
DmCvD           525  T..LADRRAERIYEV-----------------------------------------------------------------
DmFabp          109  --------------------------------------------------------------------------------
DmGlaz           98  --------------------------------------------------------------------------------
DmMic26-27       89  --------------------------------------------------------------------------------
DmNlaz          106  --------------------------------------------------------------------------------
DmMtp           363  F.YKESETTSEQLDLL----------------------------------------------------------------
CeDSc4          398  GISQSSSNNE----------------------------------------------------------------------
```

```
CeApoL1          128  ------------------------AKFFEELLHSRND-------------------------------------------
CeApoL3-Like     145  ------------------------DGVLF.E.LKSRE-------------------------------------------
CeMoma1          115  ------------------------EWTA----------------------------------------------------
CeVit1I          386  ------------------------EWTLRSQ.LVVRP-------------------------------------------
CeVit1II         358  ------------------------.RLVRIFRTTSTS-------------------------------------------
CeVit2I          386  ------------------------EWTLRSQ.LVVRP-------------------------------------------
CeVit2II         358  ------------------------.RLVRIFRTTSTS-------------------------------------------
CeVit3I          387  ------------------------EWTLRTE.LAARP-------------------------------------------
CeVit3II         355  ------------------------.RAVRMFRMCTIE-------------------------------------------
CeVit4I          387  ------------------------EWTLRTE.LAARP-------------------------------------------
CeVit4II         355  ------------------------.RAVRMFRMCTIE-------------------------------------------
CeVit5I          387  ------------------------EWTLRTE.LAARP-------------------------------------------
CeVit5II         355  ------------------------.RAVRMFRMCTIE-------------------------------------------
CeVit6I          379  ------------------------GEELKATIQYVYP-------------------------------------------
CeVit6II         375  ------------------------.RIVKV.RECNEE-------------------------------------------
DmApoLppI       1226  ------------------------NWSSKK.KLDGYELEARAQSKNIKIQLKNENGIIFSGTATYALKKELNKTIIDGQG
DmApoLppII       321  ------------------------..K.V.FIRLLRQ-------------------------------------------
DmApoLTPI       1258  YFSYFTNFQVDTLVWPTIVGNVDIQEIIDFY.VVGHV-------------------------------------------
DmApoLTPII       375  ------------------------TN.LQTSKSLDYK-------------------------------------------
DmCG31659        109  --------------------------------------------------------------------------------
DmCvD            540  ------------------------YWPIF.SRNASLELRVAAVTLLLISNPTAA--------------------------
DmFabp           109  --------------------------------------------------------------------------------
DmGlaz            98  --------------------------------------------------------------------------------
DmMic26-27        89  ------------------------.GIVGHYVETAKA-------------------------------------------
DmNlaz           106  --------------------------------------------------------------------------------
DmMtp            379  ------------------------E.YLQS.AVATHPDRKIVE-------------------------------------
CeDSc4           408  ------------------------KWHKQLMYWLG---------------------------------------------

CeApoL1          141  -------------LLNEVRRFVEDKQSSKIF---------------------------------KNFDDVQSHLKTLF--
CeApoL3-Like     158  -------------E.M.AV.KIVEDEEFFKHF--------------------------------..DG.IENK...V.--
CeMoma1          119  --------------------------------------------------------------------------------
CeVit1I          399  -------------EMPSSL.QLH.QPHREVQLSLTSTWGSQK----------------------.SEVT.NAQ.QQSK--
CeVit1II         371  -------------Q.K.IHETLYV.ADK..QSLMEHALAI------------------------AGTKNTIQ.ILVHI--
CeVit2I          399  -------------EMPSSL.QLR.QPHREVQLSLTSTWGSQK----------------------.SEVT.NAQ.QQSK--
CeVit2II         371  -------------Q.K.IHETLYV.ADK..QSLMEHALAI------------------------AGTKNTIQ.ILVHM--
CeVit3I          400  -------------QMPSSL.QLREQPHREVQLALNAKWGSSK----------------------.SEITFNAQ.EQST--
CeVit3II         368  -------------E.KK.HTTIYT.AEK.VQLVIETTLAV------------------------AGTKNTIQ..IHH.--
CeVit4I          400  -------------QMPSSL.QLREQPHREVQLALNAKWGSSK----------------------.SEITFNAQ.EQST--
CeVit4II         368  -------------E.KK.HTTIYT.AEK.VQLVIETTLAV------------------------AGTKNTIQ..IHH.--
CeVit5I          400  -------------QMPSSL.QLREQPHREVQLAFNAKWGSSK----------------------.SEIT.NAQ.EQST--
CeVit5II         368  -------------E.KK.HTTIYT.AEK.VQLVIETTLAV------------------------AGTKNTIQ..IHH.--
CeVit6I          392  -------------QTPRTVEELKEQKYRQLVVMGEMNYG-------------------------E.TIHININGQQSQ--
CeVit6II         388  -------------Q.EQIY.H.AEHKDE..AEQLRSIYFNTLAL--------------------AGTRVTIQQFVDKV--
DmApoLppI       1282  KVQYQGKALSGNFK.TRQHFDFGTDREVGFSYTFMGNLGSKNGLGTLKITNKEFNTKFSVCEEKRQCTNLIVQSIVSIDE
DmApoLppII       334  -------------SDS.TLLELAAFPHPNKVLARKVYLDGLF----------------------RTSTAESARVILKQ--
DmApoLTPI       1295  -------------E.PQGKVEFK.RLHYPDYINVHNLLTVTTPF--------------------AVAKNIK.IVEYHVDL
DmApoLTPII       388  -------------T.SVLLQRSASTCEQGRNHLLESLPF-------------------------IGSTASYKVMRDQI--
DmCG31659        109  ----------------------------------------------------------------TTSTAFPEGVLMYV--
DmCvD            570  -------------R.ISIH.IIQSETDPHMINYYRTTVTSIS----------------------ETTYPCYQ..RR.LSY
DmFabp           109  ----------------------------------------------------------------NELITLIP..TPSQ--
DmGlaz            98  ----------------------------------------------------------------TGNPN.NIGYA.PE--
DmMic26-27       102  -------------HTQSTIDMLNEP.N.L-----------------------------------HRSGAIVVGGLAG.--
DmNlaz           106  -----------------------------------------------------------------GQLA.AFYPTQPL--
DmMtp            398  -------------H.FGLLEQESI.KHL.LRESVIQTVATLTRQSGLDVEDPLL----------.EVRSYLLQGL.SK--
CeDSc4           419  -------------S.DKKSEEYWKVANTIATVLNKRCEAS------------------------TSSLNSCNKG.ETI--

CeApoL1          173  -----------------------GVSLAGI-TGFGIKMAASSMGHLTNA-------------------------------
CeApoL3-Like     191  -----------------------.G.VT..-..I.TRF.IT...R.SSS-------------------------------
CeMoma1          119  --------------------------------------------------------------------------------
CeVit1I          442  --------------EQKKYERNMDRHFN.M-PEYELLIK.ARLNQINAV---AEYKLTRE--------------------
CeVit1II         412  --------------E-NEDIVPLEAAQLLK-SIQETPFPSQTIAEALIK---FAESRVSK--------------------
CeVit2I          442  --------------EQKKYERNMDRQFN.M-PEYELLIK.ARLNQINAV---AEYKLTRE--------------------
CeVit2II         412  --------------E-NEDILPL--GQILK-.IQETPFPSQ.IAEALIK---FAESRVAK--------------------
CeVit3I          443  --------------EQKKFLRNIEREYK..-PEYELLIK.ARLNQVNVV---SEYKLTPE--------------------
CeVit3II         409  --------------E-KKSITPLRAAELLK-SVQETLYPSEHIAD.LIQ---LAQSPLSE--------------------
CeVit4I          443  --------------EQKKFLRNIEREYK..-PEYELLIK.ARLNQVNVV---SEYKLTPE--------------------
CeVit4II         409  --------------E-KKSITPLRAAELLK-SVQETLYPSEHIAD.LIQ---LAQSPLSE--------------------
CeVit5I          443  --------------EQKKFIRNIEREYK..-PEYELLIK.ARLNQVNVV---SEYKLTPQ--------------------
CeVit5II         409  --------------E-KKSITPLRAAELLK-SVQETLYPSEHIAD.LIQ---LAQSPLSE--------------------
CeVit6I          432  --------------EQKKFVKQIEQA-----PEHETLLE..RLDQYQTV---VEYEFEPK--------------------
CeVit6II         433  --------------QSRKNIAPLKA.V.IK-.LVDMRYPSLAIAEDIAR---LCESDVSS--------------------
DmApoLppI       1362  QKLDAVEHTTLIIVDLRDFGYPYEFE.KSQ-NTRQGLKYQYHLDSFIITGNNFKYQFTAN--------------------
DmApoLppII       377  -------------------LSKFDEKEKLL-AILSLNIVK.VDKETL.Q---AASQLLPN--------------------
DmApoLTPI       1342  NFNAFYERVKFIVNDDKDNTQEL.FVFNYT-ALQDNVKPKAHDVQV.LL---TPYEMLHEIYVHGHIELDDNAYKGNISA
DmApoLTPII       428  --------------INEKLTKQMAHDWMTA-LS.ITRPDEETLETFHSI---LEY-------------------------
DmCG31659        123  ----------------------LDTDYVNF-AIRFMCFD..KIFSFHW.-------------------------------
DmCvD            615  MHRHLPQKPESRYWVTGNYIFDYRD.KF..GAMLQVFLVGDPKSDMPVV---AFFKFDTE--------------------
DmFabp           123  --------------------------------------------------------------------------------
DmGlaz           112  --------------NSRSSIMDFKFTTRFP-DVIARLLPG.GKYQVLYT-------------------------------
DmMic26-27       132  -----------IFAARGGFIKKVLY.GI.AGAVASMCYPRQAEENCRVV-------------------------------
DmNlaz           119  -------------------------------.KANYLVLGTDYESYAVV---YSCTSVTP--------------------
DmMtp            453  --------------EPTLYIRALQNLQDPA-.I-EALLEHAQT.EAP.LSVAALQALKAF--------------------
CeDSc4           460  ---------------VNKFITDLTAGGVEV-RVLEVLENIPIF.SY.F.---KKFICETE--------------------
```

```
CeApoL1          198  --------------------------------------------------------------------------------
CeApoL3-Like     216  --------------------------------------------------------------------------------
CeMoma1          119  --------------------------------------------------------------------------------
CeVit1I          484  --------------------------------------------------------------------------------
CeVit1II         453  --------------------------------------------------------------------------------
CeVit2I          484  --------------------------------------------------------------------------------
CeVit2II         451  --------------------------------------------------------------------------------
CeVit3I          485  --------------------------------------------------------------------------------
CeVit3II         450  --------------------------------------------------------------------------------
CeVit4I          485  --------------------------------------------------------------------------------
CeVit4II         450  --------------------------------------------------------------------------------
CeVit5I          485  --------------------------------------------------------------------------------
CeVit5II         450  --------------------------------------------------------------------------------
CeVit6I          470  --------------------------------------------------------------------------------
CeVit6II         475  --------------------------------------------------------------------------------
DmApoLppI       1421  --------------------------------------------------------------------------------
DmApoLppII       414  --------------------------------------------------------------------------------
DmApoLTPI       1418  VTAHTHLSMAASIENEDNFLETSVGIQLETDAIPHYGCQVYFKKDFSAVDKAIDIRFEVTDNGTLNQLHISTDWHTDPSY
DmApoLTPII       465  --------------------------------------------------------------------------------
DmCG31659        149  --------------------------------------------------------------------------------
DmCvD            672  --------------------------------------------------------------------------------
DmFabp           123  --------------------------------------------------------------------------------
DmGlaz           146  --------------------------------------------------------------------------------
DmMic26-27       170  --------------------------------------------------------------------------------
DmNlaz           145  --------------------------------------------------------------------------------
DmMtp            497  --------------------------------------------------------------------------------
CeDSc4           501  --------------------------------------------------------------------------------

CeApoL1          198  --------------------------------------------------------------------------------
CeApoL3-Like     216  --------------------------------------------------------------------------------
CeMoma1          119  --------------------------------------------------------------------------------
CeVit1I          484  ------------------------------------------------------TEQVLARYFD----------------
CeVit1II         453  ------------------------------------------------------NNQVVRQSAW----------------
CeVit2I          484  ------------------------------------------------------TEQVLARYFD----------------
CeVit2II         451  ------------------------------------------------------NNLVVRQAAW----------------
CeVit3I          485  ------------------------------------------------------SEYTFSRIFD----------------
CeVit3II         450  ------------------------------------------------------KYEPLRQSAW----------------
CeVit4I          485  ------------------------------------------------------SEYTFSRIFD----------------
CeVit4II         450  ------------------------------------------------------KYEPLRQSAW----------------
CeVit5I          485  ------------------------------------------------------SEYTFSRIFD----------------
CeVit5II         450  ------------------------------------------------------KYEPLRQSAW----------------
CeVit6I          470  ------------------------------------------------------PAQYFARYWN----------------
CeVit6II         475  ------------------------------------------------------SFPALRQSCW----------------
DmApoLppI       1421  ---------------------------------------------VQPTSSTIKLALPKRQILFETTQKIPADGS-----
DmApoLppII       414  -----------------------------------------------------APKELYIAVGN----------------
DmApoLTPI       1498  IVNANGRIKTTMLPLQMASTSVLVIQGQNPHLNFDLNLLSQNGQSIAYGARANKKKDVFNIEVWTPMKNFRNISMHGTAI
DmApoLTPII       465  ------------------------------------------------------AKNRLDAEYT----------------
DmCG31659        149  --------------------------------------------------------------------------------
DmCvD            672  -------------------ALGKFTGQLALYIKARGLPDTILNKMQSRNGSDPFTFKSIKALLA----------------
DmFabp           123  --------------------------------------------------------------------------------
DmGlaz           146  ------------------------------------------------------DYENF---------------------
DmMic26-27       170  --------------------------------------------------------------------------------
DmNlaz           145  --------------------------------------------------------------------------------
DmMtp            497  ------------------------------------------------------PLGSFNSSHR----------------
CeDSc4           501  ------------------------------------------------------SEDVQKAALNVI--------------

CeApoL1          198  --------------------------------------------------------------------------------
CeApoL3-Like     216  --------------------------------------------------------------------------------
CeMoma1          119  --------------------------------------------------------------------------------
CeVit1I          494  --------------------------------------------------------------------------------
CeVit1II         463  --------------------------------------------------------------------------------
CeVit2I          494  --------------------------------------------------------------------------------
CeVit2II         461  --------------------------------------------------------------------------------
CeVit3I          495  --------------------------------------------------------------------------------
CeVit3II         460  --------------------------------------------------------------------------------
CeVit4I          495  --------------------------------------------------------------------------------
CeVit4II         460  --------------------------------------------------------------------------------
CeVit5I          495  --------------------------------------------------------------------------------
CeVit5II         460  --------------------------------------------------------------------------------
CeVit6I          480  --------------------------------------------------------------------------------
CeVit6II         485  --------------------------------------------------------------------------------
DmApoLppI       1451  --------------------------------------------------------------------------------
DmApoLppII       425  --------------------------------------------------------------------------------
DmApoLTPI       1578  RSPRDPGRYDVSGFLYRNMATYEVTGAVRMTNSLPIDVVLRVQPKAGGRDGVIELNIHEAGPKKIRFSFSAIEDGKMCQM
DmApoLTPII       475  --------------------------------------------------------------------------------
DmCG31659        149  --------------------------------------------------------------------------------
DmCvD            717  --------------------------------------------------------------------------------
DmFabp           123  --------------------------------------------------------------------------------
DmGlaz           151  --------------------------------------------------------------------------------
DmMic26-27       170  --------------------------------------------------------------------------------
DmNlaz           145  --------------------------------------------------------------------------------
DmMtp            507  --------------------------------------------------------------------------------
CeDSc4           513  --------------------------------------------------------------------------------
```

```
CeApoL1         198  --------------------------------------------------------------------------------
CeApoL3-Like    216  --------------------------------------------------------------------------------
CeMoma1         119  --------------------------------------------------------------------------------
CeVit1I         494  --------------------------------------------------------------------------------
CeVit1II        463  --------------------------------------------------------------------------------
CeVit2I         494  --------------------------------------------------------------------------------
CeVit2II        461  --------------------------------------------------------------------------------
CeVit3I         495  --------------------------------------------------------------------------------
CeVit3II        460  --------------------------------------------------------------------------------
CeVit4I         495  --------------------------------------------------------------------------------
CeVit4II        460  --------------------------------------------------------------------------------
CeVit5I         495  --------------------------------------------------------------------------------
CeVit5II        460  --------------------------------------------------------------------------------
CeVit6I         480  --------------------------------------------------------------------------------
CeVit6II        485  --------------------------------------------------------------------------------
DmApoLppI      1451  --------------------------------------------------------------------------------
DmApoLppII      425  --------------------------------------------------------------------------------
DmApoLTPI      1658  SGGYSVSKTNGAMDFSVLVESTEPEIARINFYGNLSPNSEGSLVGDLSLETPWKALGIDTVHLHSDVGFLNKGGHIVGEY
DmApoLTPII      475  --------------------------------------------------------------------------------
DmCG31659       149  --------------------------------------------------------------------------------
DmCvD           717  --------------------------------------------------------------------------------
DmFabp          123  --------------------------------------------------------------------------------
DmGlaz          151  --------------------------------------------------------------------------------
DmMic26-27      170  --------------------------------------------------------------------------------
DmNlaz          145  --------------------------------------------------------------------------------
DmMtp           507  --------------------------------------------------------------------------------
CeDSc4          513  --------------------------------------------------------------------------------

CeApoL1         198  -----LMKG-----------------------------------------------------------------------
CeApoL3-Like    216  -----.V..-----------------------------------------------------------------------
CeMoma1         119  -----.P.AAAITV------------------------------------------------------------------
CeVit1I         494  -----.V.AYNYWT------------------------------------------------------------------
CeVit1II        463  -----.AA.SVVRG------------------------------------------------------------------
CeVit2I         494  -----.V.TYNYWT------------------------------------------------------------------
CeVit2II        461  -----.AA.SVVRG------------------------------------------------------------------
CeVit3I         495  -----.I.AYNFWT------------------------------------------------------------------
CeVit3II        460  -----.AA.SVVRG------------------------------------------------------------------
CeVit4I         495  -----.I.AYNFWT------------------------------------------------------------------
CeVit4II        460  -----.AA.SVVRG------------------------------------------------------------------
CeVit5I         495  -----.I.AYNFWT------------------------------------------------------------------
CeVit5II        460  -----.AA.SVVRG------------------------------------------------------------------
CeVit6I         480  -----MVQAYLRTQ------------------------------------------------------------------
CeVit6II        485  -----.TY.AIVNG------------------------------------------------------------------
DmApoLppI      1451  -----.FGRYEQTA------------------------------------------------------------------
DmApoLppII      425  -----.VAKYCLKN------------------------------------------------------------------
DmApoLTPI      1738  KIGQYIGR.SCLWSWILAEDMQLVLENYLERPNAKPRIVHASAKYQNPGQTFTQLQAGGRLSVDSKWNLDVNGSAEYKSV
DmApoLTPII      475  -----.GATAVVHS------------------------------------------------------------------
DmCG31659       149  --------------------------------------------------------------------------------
DmCvD           717  -----MLQAPIINS------------------------------------------------------------------
DmFabp          123  --------------------------------------------------------------------------------
DmGlaz          151  ---------AILWS------------------------------------------------------------------
DmMic26-27      170  -----.YE.RKIFA------------------------------------------------------------------
DmNlaz          145  -----.ANFKIVWI------------------------------------------------------------------
DmMtp           507  -----.QFESIFYQ------------------------------------------------------------------
CeDSc4          513  -----.AASKNLYE------------------------------------------------------------------

CeApoL1         202  --------------------------------------------------------------------------------
CeApoL3-Like    220  --------------------------------------------------------------------------------
CeMoma1         128  --------------------------------------------------------------------------------
CeVit1I         503  --------------------------------------------------------------------------------
CeVit1II        472  --------------------------------------------------------------------------------
CeVit2I         503  --------------------------------------------------------------------------------
CeVit2II        470  --------------------------------------------------------------------------------
CeVit3I         504  --------------------------------------------------------------------------------
CeVit3II        469  --------------------------------------------------------------------------------
CeVit4I         504  --------------------------------------------------------------------------------
CeVit4II        469  --------------------------------------------------------------------------------
CeVit5I         504  --------------------------------------------------------------------------------
CeVit5II        469  --------------------------------------------------------------------------------
CeVit6I         489  --------------------------------------------------------------------------------
CeVit6II        494  --------------------------------------------------------------------------------
DmApoLppI      1460  --------------------------------------------------------------------------------
DmApoLppII      434  --------------------------------------------------------------------------------
DmApoLTPI      1818  DDFKFRVITALPLPVGDRHQLSASYQGNVISQQFNNPDFVLEASYESFEAQNKLLSRISYKNATNNLKGLGHVEWGKIQN
DmApoLTPII      484  --------------------------------------------------------------------------------
DmCG31659       149  --------------------------------------------------------------------------------
DmCvD           726  --------------------------------------------------------------------------------
DmFabp          123  --------------------------------------------------------------------------------
DmGlaz          156  --------------------------------------------------------------------------------
DmMic26-27      179  --------------------------------------------------------------------------------
DmNlaz          154  --------------------------------------------------------------------------------
DmMtp           516  --------------------------------------------------------------------------------
CeDSc4          522  --------------------------------------------------------------------------------
```

```
CeApoL1         202  --------------------------------------------------------------------------------
CeApoL3-Like    220  --------------------------------------------------------------------------------
CeMoma1         128  --------------------------------------------------------------------------------
CeVit1I         503  ------------------------------------V-----SSRPENNE------------------------------
CeVit1II        472  ------------------------------------I-----VDYKNIRPL------------VR---------------
CeVit2I         503  ------------------------------------V-----SSRPENNE------------------------------
CeVit2II        470  ------------------------------------I-----VDYKNIRPL------------VR---------------
CeVit3I         504  ------------------------------------V-----SEKRVQNE------------------------------
CeVit3II        469  ------------------------------------F-----ASKTQDLPL------------IR---------------
CeVit4I         504  ------------------------------------V-----SEKRVQNE------------------------------
CeVit4II        469  ------------------------------------F-----ASKTQDLPL------------IR---------------
CeVit5I         504  ------------------------------------V-----SEKRVQNE------------------------------
CeVit5II        469  ------------------------------------F-----ASKTQDLPL------------IR---------------
CeVit6I         489  ------------------------------------YPWTSRIETREEPSR-----------------------------
CeVit6II        494  ------------------------------------V-----CGQTPRVFV-----------------------------
DmApoLppI      1460  ------------------------------------SFFIDKLQKPDDVARFSAIVDVTGTERVAFNA------------
DmApoLppII      434  ------------------------------------Y-----CQGPEID-------------------------------
DmApoLTPI      1898  LSVVEGDFELLHKQGAQREFSAKIITPKFKNEHTFALTGSYDLEKSGHHNVVGSLDYPASRRITDLDVSVSSLSNMHGIF
DmApoLTPII      484  ------------------------------------F-----CKHHEACE------------------------------
DmCG31659       149  --------------------------------------------------------------------------------
DmCvD           726  ----------------------KDLHLEFILQMEGKTVLSYYLNQRMFRQLTYDNILERMQQIIRTDSHINMQTVRWPFM
DmFabp          123  --------------------------------------------------------------------------------
DmGlaz          156  ------------------------------------------CG------------------------------------
DmMic26-27      179  --------------------------------------------------------------------------------
DmNlaz          154  --------------------------------------------------------------------------------
DmMtp           516  ------------------------------------------RKRRFD--------------------------------
CeDSc4          522  ------------------------------------T-------------------------------------------

CeApoL1         202  --------------------------------------------------------------------------------
CeApoL3-Like    220  --------------------------------------------------------------------------------
CeMoma1         128  --------------------------------------------------------------------------------
CeVit1I         512  --------------------------------------------------------------------------------
CeVit1II        484  --------------------------------------------------------------------------------
CeVit2I         512  --------------------------------------------------------------------------------
CeVit2II        482  --------------------------------------------------------------------------------
CeVit3I         513  --------------------------------------------------------------------------------
CeVit3II        481  --------------------------------------------------------------------------------
CeVit4I         513  --------------------------------------------------------------------------------
CeVit4II        481  --------------------------------------------------------------------------------
CeVit5I         513  --------------------------------------------------------------------------------
CeVit5II        481  --------------------------------------------------------------------------------
CeVit6I         504  --------------------------------------------------------------------------------
CeVit6II        504  --------------------------------------------------------------------------------
DmApoLppI      1492  --------------------------------------------------------------------------------
DmApoLppII      442  --------------------------------------------------------------------------------
DmApoLTPI      1978  NSTLPTFLNVSWLKTDFNFTTNNGKSYRYCRCFWPQDTAYFKLNSNYDSDSSNFNHNLNGNVEIEVPLATRHRADIVYGL
DmApoLTPII      493  --------------------------------------------------------------------------------
DmCG31659       149  --------------------------------------------------------------------------------
DmCvD           784  NRYTV---------------------------------------------------------------------------
DmFabp          123  --------------------------------------------------------------------------------
DmGlaz          158  --------------------------------------------------------------------------------
DmMic26-27      179  --------------------------------------------------------------------------------
DmNlaz          154  --------------------------------------------------------------------------------
DmMtp           522  --------------------------------------------------------------------------------
CeDSc4          523  --------------------------------------------------------------------------------

CeApoL1         202  ----------------------------------------------------MLQ---------SVA-------------
CeApoL3-Like    220  ----------------------------------------------------V.H---------...-------------
CeMoma1         128  -------------------------------------------GGMAGFVL-G.K---------RGP-------------
CeVit1I         512  ----------------NDRV------VVQLTVEPMSRQYVNITMQSPIERV-E.K---------N.Q-------------
CeVit1II        484  ----------------EDKR------ELKEKFLRVFMQQYKDAETTYEKIL-A.K---------.IG-------------
CeVit2I         512  ----------------NDRV------VVQLTVEPMSRQYVNITMQSPMERI-E.K---------N.Q-------------
CeVit2II        482  ----------------EDKR------ELKEKFLRVFMQQYKDAETTYEKIL-A.K---------TIG-------------
CeVit3I         513  ----------------DRRV------VLQLSVEPLSRQYMNMTIQTPEQEV-E.K---------N.R-------------
CeVit3II        481  ----------------PASR------QTKEKYVRVFMQHFRNADSTYEKVL-A.K---------TLG-------------
CeVit4I         513  ----------------DRRV------VLQLSVEPLSRQYMNMTIQTPEQEV-E.K---------N.R-------------
CeVit4II        481  ----------------PASR------QTKEKYVRVFMQHFRNADSTYEKVL-A.K---------TLG-------------
CeVit5I         513  ----------------NRRV------VLQLSVEPLSRQYMNMTIQTPEQEV-E.K---------N.R-------------
CeVit5II        481  ----------------PASR------QTKEKYVRVFMQHFRNADSTYEKVL-A.K---------TLG-------------
CeVit6I         504  ----------------KNMI------RATINVEPRQRLTVNMTIETPMETT-V.E---------R.E-------------
CeVit6II        504  ----------------QKNGVKMCPRDAKQRIVDKLVQQFESASTRYEKVL-A.K---------TL.-------------
DmApoLppI      1492  ----------------NGKLKFEHPTIRPLSISGQLNGDVNQQIASAEVIF-DIF---------RLPEQKVVGNSELRNS
DmApoLppII      442  --------------------------AISKKFSDGLKHCKPNTKREEERIVYI.K---------GLG-------------
DmApoLTPI      2058  QKRRNQDAGNVKVVYNEKQVLDGKYKRLEQAKAPIYKETTDISLENEVKPL-GIH---------F.STRDASDPAGSQDV
DmApoLTPII      493  ----------------ENLRVQQIINLLETEFLNLYNLFKGERRTRERMVI-L.K---------GLG-------------
DmCG31659       149  ----------------------------------------------------VI.-------------------------
DmCvD           789  ----------------PTVLGTSSDVLLQTTVLTSLRGNITEQRNSPITKH-T.EIDARYSSYA..R-------------
DmFabp          123  ----------------------------------------------------------------HGS-------------
DmGlaz          158  ----------------------------------------------------SIG---------.LG-------------
DmMic26-27      179  --------------------------------------------VAYNFIK-GVK-------------------------
DmNlaz          154  --------------------------------LTRQREPSAEAVDAARKIL-EDN---------D.S-------------
DmMtp           522  ----------------SSARTLALDIILSLRPTQEQLGNFLDYLASNDRQF-EIK---------TYV-------------
CeDSc4          523  --------------------------QLTHKLIKLFRNTCSQETPTSHSQL-AID---------ILLKCVPDHQN-----
```

```
CeApoL1          208  --------------------------------------A-----------------------------------------
CeApoL3-Like     226  --------------------------------------V-----------------------------------------
CeMoma1          142  --------------------------------------V-----------------------------------------
CeVit1I          547  --------------------------------------V-----------------------------------------
CeVit1II         519  --------------------------------------N-----------------------------------------
CeVit2I          547  --------------------------------------V-----------------------------------------
CeVit2II         517  --------------------------------------N-----------------------------------------
CeVit3I          548  --------------------------------------I-----------------------------------------
CeVit3II         516  --------------------------------------N-----------------------------------------
CeVit4I          548  --------------------------------------I-----------------------------------------
CeVit4II         516  --------------------------------------N-----------------------------------------
CeVit5I          548  --------------------------------------I-----------------------------------------
CeVit5II         516  --------------------------------------N-----------------------------------------
CeVit6I          539  --------------------------------------------------------------------------------
CeVit6II         545  --------------------------------------N-----------------------------------------
DmApoLppI       1546  RSQNGFNIAYITTVKSAGLQFQYQINSNAAVDIEAHEYN-----------------------------------------
DmApoLppII       474  --------------------------------------------------------------------------------
DmApoLTPI       2128  KHIEIYELRNTQNFNLTGELHSRATLKAQDFKVVAIHPNRAVVLSTKYEDVSPEVVRHHSKLELSETAWIGYNLELGNFS
DmApoLTPII       534  --------------------------------------N-----------------------------------------
DmCG31659        152  --------------------------------------------------------------------------------
DmCvD            839  --------------------------------------------------------------------------------
DmFabp           126  --------------------------------------------------------------------------------
DmGlaz           164  --------------------------------------------------------------------------------
DmMic26-27       189  --------------------------------------------------------------------------------
DmNlaz           179  --------------------------------------------------------------------------------
DmMtp            563  --------------------------------------L-----------------------------------------
CeDSc4           562  --------------------------------------V-----------------------------------------

CeApoL1          209  --------------------------------------------------------------------------------
CeApoL3-Like     227  --------------------------------------------------------------------------------
CeMoma1          143  --------------------------------------------------------------------------------
CeVit1I          548  --------------------------------------------------------------------------------
CeVit1II         520  --------------------------------------------------------------------------------
CeVit2I          548  --------------------------------------------------------------------------------
CeVit2II         518  --------------------------------------------------------------------------------
CeVit3I          549  --------------------------------------------------------------------------------
CeVit3II         517  --------------------------------------------------------------------------------
CeVit4I          549  --------------------------------------------------------------------------------
CeVit4II         517  --------------------------------------------------------------------------------
CeVit5I          549  --------------------------------------------------------------------------------
CeVit5II         517  --------------------------------------------------------------------------------
CeVit6I          539  --------------------------------------------------------------------------------
CeVit6II         546  --------------------------------------------------------------------------------
DmApoLppI       1585  --------------------------------------------------------------------------------
DmApoLppII       474  --------------------------------------------------------------------------------
DmApoLTPI       2208  KVGNESQSFALEIFYPKRNLSSSGQYYMTDTNFNSDLSFQWLGGNYDQQPKIIHSNLQWKAEPLHRGDREHRTIALTVAH
DmApoLTPII       535  --------------------------------------------------------------------------------
DmCG31659        152  --------------------------------------------------------------------------------
DmCvD            839  --------------------------------------------------------------------------------
DmFabp           126  --------------------------------------------------------------------------------
DmGlaz           164  --------------------------------------------------------------------------------
DmMic26-27       189  --------------------------------------------------------------------------------
DmNlaz           179  --------------------------------------------------------------------------------
DmMtp            564  --------------------------------------------------------------------------------
CeDSc4           563  --------------------------------------------------------------------------------

CeApoL1          209  --------------------------------------------------------------------------------
CeApoL3-Like     227  --------------------------------------------------------------------------------
CeMoma1          143  --------------------------------------------------------------------------------
CeVit1I          548  --------------------------------------------------------------------------------
CeVit1II         520  --------------------------------------------------------------------------------
CeVit2I          548  --------------------------------------------------------------------------------
CeVit2II         518  --------------------------------------------------------------------------------
CeVit3I          549  --------------------------------------------------------------------------------
CeVit3II         517  --------------------------------------------------------------------------------
CeVit4I          549  --------------------------------------------------------------------------------
CeVit4II         517  --------------------------------------------------------------------------------
CeVit5I          549  --------------------------------------------------------------------------------
CeVit5II         517  --------------------------------------------------------------------------------
CeVit6I          539  --------------------------------------------------------------------------------
CeVit6II         546  --------------------------------------------------------------------------------
DmApoLppI       1585  --------------------------------------------------------------------------------
DmApoLppII       474  --------------------------------------------------------------------------------
DmApoLTPI       2288  PLLEKDINCKATYYRGLRDLLRTHLTIDYSEYPDQLIELGAQLTDRYSELGHTNYTFHVYGKHIASELDVQLNGTLAAMN
DmApoLTPII       535  --------------------------------------------------------------------------------
DmCG31659        152  --------------------------------------------------------------------------------
DmCvD            839  --------------------------------------------------------------------------------
DmFabp           126  --------------------------------------------------------------------------------
DmGlaz           164  --------------------------------------------------------------------------------
DmMic26-27       189  --------------------------------------------------------------------------------
DmNlaz           179  --------------------------------------------------------------------------------
DmMtp            564  --------------------------------------------------------------------------------
CeDSc4           563  --------------------------------------------------------------------------------
```

```
CeApoL1          209  --------------------------------------------------IGIVLDGVTLA-------------------
CeApoL3-Like     227  --------------------------------------------------......S....-------------------
CeMoma1          143  --------------------------------------------------GRLLTTTIG..-------------------
CeVit1I          548  --------------------------------------------------PRVY.PSIAQR-------------------
CeVit1II         520  --------------------------------------------------A.LDISVNQ.N-------------------
CeVit2I          548  --------------------------------------------------PRVY.PSIAQR-------------------
CeVit2II         518  --------------------------------------------------A.LDISVNQ.N-------------------
CeVit3I          549  --------------------------------------------------PRV..PTIARR-------------------
CeVit3II         517  --------------------------------------------------A..D.SVYE.V-------------------
CeVit4I          549  --------------------------------------------------PRV..PTIARR-------------------
CeVit4II         517  --------------------------------------------------A..D.SVYE.V-------------------
CeVit5I          549  --------------------------------------------------PRV..PTIARS-------------------
CeVit5II         517  --------------------------------------------------A..D.SVYE.V-------------------
CeVit6I          539  --------------------------------------------------LPFR.PTAQIH-------------------
CeVit6II         546  --------------------------------------------------A.LD.SVYP.E-------------------
DmApoLppI       1585  --------------------------------------------------..LE.NNGEID------------------V
DmApoLppII       474  --------------------------------------------------NAKS.S.N.V.-------------------
DmApoLTPI       2368  SYYKTESTAHYKRDIFPARYGKFLALLDVNKRELEYERQSPFHAVRLHLLPT.RYPIYG.NATIWDTPDTNHSGYIYMDI
DmApoLTPII       535  --------------------------------------------------..V.SSAFAEQ-------------------
DmCG31659        152  --------------------------------------------------TRKR.PSTQVI-------------------
DmCvD            839  --------------------------------------------------SRSYNPFLN.D-------------------
DmFabp           126  --------------------------------------------------LRLRVPCQPVP-------------------
DmGlaz           164  --------------------------------------------------HSDQIWILGRD-------------------
DmMic26-27       189  --------------------------------------------------P.EDVPV.PFP-------------------
DmNlaz           179  --------------------------------------------------QAFLI.T.Q---------------------
DmMtp            564  --------------------------------------------------QKLRMLAEKCP-------------------
CeDSc4           563  --------------------------------------------------ATLI.RTE..N-------------------

CeApoL1          220  --------------------------------------------------------------------------------
CeApoL3-Like     238  --------------------------------------------------------------------------------
CeMoma1          154  --------------------------------------------------------------------------------
CeVit1I          559  --------------------------------------------------------------------------------
CeVit1II         531  --------------------------------------------------------------------------------
CeVit2I          559  --------------------------------------------------------------------------------
CeVit2II         529  --------------------------------------------------------------------------------
CeVit3I          560  --------------------------------------------------------------------------------
CeVit3II         528  --------------------------------------------------------------------------------
CeVit4I          560  --------------------------------------------------------------------------------
CeVit4II         528  --------------------------------------------------------------------------------
CeVit5I          560  --------------------------------------------------------------------------------
CeVit5II         528  --------------------------------------------------------------------------------
CeVit6I          550  --------------------------------------------------------------------------------
CeVit6II         557  --------------------------------------------------------------------------------
DmApoLppI       1597  KAISFLNKEKFEISLSESNKHIIYIVGDFSKQNHYAKLNTKVQILDKNPIEITSEVQPNSAKIILKRQDFIDGTAEVKLG
DmApoLppII       485  --------------------------------------------------------------------------------
DmApoLTPI       2448  LERYARMDFNLTEDASQNLQMVGYIPDTRSAFLDIWRNYEEIRVIDVSSYLKMNHSRLITGRFHWRPSIRQEVREKIQAV
DmApoLTPII       546  --------------------------------------------------------------------------------
DmCG31659        163  --------------------------------------------------------------------------------
DmCvD            850  --------------------------------------------------------------------------------
DmFabp           137  --------------------------------------------------------------------------------
DmGlaz           175  --------------------------------------------------------------------------------
DmMic26-27       200  --------------------------------------------------------------------------------
DmNlaz           188  --------------------------------------------------------------------------------
DmMtp            575  --------------------------------------------------------------------------------
CeDSc4           574  --------------------------------------------------------------------------------

CeApoL1          220  --------------------------------------------------------------------------------
CeApoL3-Like     238  --------------------------------------------------------------------------------
CeMoma1          154  --------------------------------------------------------------------------------
CeVit1I          559  --------------------------------------------------------------------------------
CeVit1II         531  --------------------------------------------------------------------------------
CeVit2I          559  --------------------------------------------------------------------------------
CeVit2II         529  --------------------------------------------------------------------------------
CeVit3I          560  --------------------------------------------------------------------------------
CeVit3II         528  --------------------------------------------------------------------------------
CeVit4I          560  --------------------------------------------------------------------------------
CeVit4II         528  --------------------------------------------------------------------------------
CeVit5I          560  --------------------------------------------------------------------------------
CeVit5II         528  --------------------------------------------------------------------------------
CeVit6I          550  --------------------------------------------------------------------------------
CeVit6II         557  --------------------------------------------------------------------------------
DmApoLppI       1677  KEFKVDVIGSGKQLFNGRVALDATNFLQTNYFINEDHLNGFWHIVESEINKDSEYISENIKERLKKSRQVTDKIVKLAKE
DmApoLppII       485  --------------------------------------------------------------------------------
DmApoLTPI       2528  GKSVYSSFSEGIDFWIKSIYTETTESMGVVWNTAKEYNRDFIDDIGQLSVLEEDLADLRLFVNQSYEANDFYIKNVVNFT
DmApoLTPII       546  --------------------------------------------------------------------------------
DmCG31659        163  --------------------------------------------------------------------------------
DmCvD            850  ----------------------------------------------------------------HEINREQGFLIYIPFS
DmFabp           137  --------------------------------------------------------------------------------
DmGlaz           175  --------------------------------------------------------------------------------
DmMic26-27       200  --------------------------------------------------------------------------------
DmNlaz           188  --------------------------------------------------------------------------------
DmMtp            575  --------------------------------------------------------------------------------
CeDSc4           574  --------------------------------------------------------------------------------
```

```
CeApoL1          220  --------------------------------------------------------------------------------
CeApoL3-Like     238  --------------------------------------------------------------------------------
CeMoma1          154  --------------------------------------------------------------------------------
CeVit1I          559  -----------------SVKHLLNEASGSVCKVQ----------------------------------------------
CeVit1II         531  -----------------EIIVDKRQLLPVRKEAI----------------------------------------------
CeVit2I          559  -----------------SVKHQLTEASGSVCKVQ----------------------------------------------
CeVit2II         529  -----------------EIIVDKRQPLPVRKEAI----------------------------------------------
CeVit3I          560  -----------------AMFQQTWEKTGATCKVG----------------------------------------------
CeVit3II         528  -----------------QLIQDPRQPLSIRTEAV----------------------------------------------
CeVit4I          560  -----------------AMFQQTWEKTGATCKVD----------------------------------------------
CeVit4II         528  -----------------QLIQDPRQPLSIRTEAV----------------------------------------------
CeVit5I          560  -----------------AMFQQTWEKTGATCKVD----------------------------------------------
CeVit5II         528  -----------------QIIQDPRQPLSIRTEAV----------------------------------------------
CeVit6I          550  ------YQPRNSRYEQKPVMEKIAHHASKQANCV----------------------------------------------
CeVit6II         557  -----------------KIILNEQHETTIRTQAI----------------------------------------------
DmApoLppI       1757  AGPDFSKLQGKLLDYKNDIVQELEADQSIAPIID----------------------------------------------
DmApoLppII       485  -----------------ALSECASTGRSNRIRVA----------------------------------------------
DmApoLTPI       2608  LTILDELAIRDHIESLPKIFSELWQAMGDSGKALRNSIVWLIETIKTTYNNLLDAVARFFHGESLVYISGLLEKGIAKYD
DmApoLTPII       546  ---------------LQWIIREDEAPVDIRLHGI----------------------------------------------
DmCG31659        163  --------------------------------------------------------------------------------
DmCvD            866  SELHLNESGSKCRRYSFSRPQNLTSGLSFKSRAV----------------------------------------------
DmFabp           137  --------------------------------------------------------------------------------
DmGlaz           175  --------------------------------------------------------------------------------
DmMic26-27       200  --------------------------------------------------------------------------------
DmNlaz           188  --------------------------------------------------------------------------------
DmMtp            575  -----------------------RFRALFKSELV----------------------------------------------
CeDSc4           574  -----------------PDDQEKWHYLYKAIEAS----------------------------------------------

CeApoL1          220  --------------------------------------------------------------------------------
CeApoL3-Like     238  --------------------------------------------------------------------------------
CeMoma1          154  --------------------------------------------------------------------------------
CeVit1I          576  --------------------------------------------------------------------------------
CeVit1II         548  --------------------------------------------------------------------------------
CeVit2I          576  --------------------------------------------------------------------------------
CeVit2II         546  --------------------------------------------------------------------------------
CeVit3I          577  --------------------------------------------------------------------------------
CeVit3II         545  --------------------------------------------------------------------------------
CeVit4I          577  --------------------------------------------------------------------------------
CeVit4II         545  --------------------------------------------------------------------------------
CeVit5I          577  --------------------------------------------------------------------------------
CeVit5II         545  --------------------------------------------------------------------------------
CeVit6I          578  --------------------------------------------------------------------------------
CeVit6II         574  --------------------------------------------------------------------------------
DmApoLppI       1791  --------------------------------------------------------------------------------
DmApoLppII       502  --------------------------------------------------------------------------------
DmApoLTPI       2688  SFIKDLHIKFIKYIENLWHKTWTLAENHWKAVLKRFEPHLFKMISFIETTAWNLSKEVFDFIYKRTNELAESPYFNKVSS
DmApoLTPII       565  --------------------------------------------------------------------------------
DmCG31659        163  --------------------------------------------------------------------------------
DmCvD            900  --------------------------------------------------------------------------------
DmFabp           137  --------------------------------------------------------------------------------
DmGlaz           175  --------------------------------------------------------------------------------
DmMic26-27       200  --------------------------------------------------------------------------------
DmNlaz           188  --------------------------------------------------------------------------------
DmMtp            586  --------------------------------------------------------------------------------
CeDSc4           591  --------------------------------------------------------------------------------

CeApoL1          220  ---------------MSANTLKN---------------------------------------------------------
CeApoL3-Like     238  ---------------L..K..GE---------------------------------------------------------
CeMoma1          154  --------------------------------------------------------------------------------
CeVit1I          576  --------------KNQIR.FDDVL---YNTPLTTCYSLIA---KDC---SEEPTFAVLSKKTEKNSEE-----------
CeVit1II         548  ---------------DALRL..DTMPRKIQKVLLPIYK--N---RQY---EPEIRMLALWRMMHTRPEE-----------
CeVit2I          576  --------------KNQIR.FDDVL---YNTPLTTCYSLIA---KDC---SEEPTFAVLSKKTEKNSEE-----------
CeVit2II         546  ---------------DALRL..DTMPRKIQKVLLPIYK--N---RQY---EPEIRMLALWRMMHTRPEE-----------
CeVit3I          577  --------------QSEVS.FD.VI---YRAPLTTCYSLVA---KDC---SEQPRFAVLAKKINKNSEE-----------
CeVit3II         545  ---------------DALRL..DVMPRKIQKVLLPVYK--N---RQN---KPELRMAALWRMMHTIPEE-----------
CeVit4I          577  --------------QSEVS.FD.VI---YRAPLTTCYSLVA---KDC---SEQPRFAVLAKKINKNSEE-----------
CeVit4II         545  ---------------DALRL..DVMPRKIQKVLLPVYK--N---RQN---KPELRMAALWRMMHTIPEE-----------
CeVit5I          577  --------------QSEVS.FD.VI---YRAPLTTCYSLVA---KDC---SEQPRFAVLAKKINKNSEE-----------
CeVit5II         545  ---------------DALRL..DVMPRKIQKVLLPVYK--N---RQN---KPELRMAALWRMMHTIPEE-----------
CeVit6I          578  ------------VKSTKI..FDQVA---YRNQFTPCYSVLA---KDCGSEKSEPRFVVLMKKINEKKEW-----------
CeVit6II         574  ---------------E.FRR.RTQMPTKIQRVLMPVYL--N---RQQ---PQHIRMSALHQIIYTQPEW-----------
DmApoLppI       1791  --GIRTLFKKIAGIVDDI.KAISEILEKAQKSIVDIYDKLQALWKDSLLKAWEDFIITVQKLISTLKTEFIKICTQSFKD
DmApoLppII       502  ---------------ALHAFS.VKCEETLQSKSLELLK--N---RNE---DSELRIEAYLSAI-SCPNA-----------
DmApoLTPI       2768  FTADAERLYRDFKANDAITNI.KYSTIAWNFVKEKYFKLVP---FGA---ELNEVLTEIWQEIKELEKI-----------
DmApoLTPII       565  ---------------LAFRRVDCARHRSYFLDNYGNYTL-----------NSELRIYSYLQAM-RCPDY-----------
DmCG31659        163  -------------------------------HMAQYFG------------------------------------------
DmCvD            900  ------TKTRGLITKTA.APFEEIMVPEGRNDVVQLFSYPMTDLGVRLSMTTNLNELIKYRGMLLKSEFTENGFSGNMVV
DmFabp           137  --------------------------------------------------------------------------------
DmGlaz           175  --------------------------------------------RDF---EVDIR-------------------------
DmMic26-27       200  ---------------T.L--------------------------------------------------------------
DmNlaz           188  --------------------------------------------KNC---------------------------------
DmMtp            586  -------------KRRHV.NYNVLGQKGLTTVLTRQLS------QAPAFNETLLSTQEVYQGILKRGSV-----------
CeDSc4           591  ---------------GNKDE..AEFWSRMRK--FKVFR------PNFLHRALQADSHVHWQEIADASNF-----------
```

```
CeApoL1         228  -------------------------------GSSSELAGKIREASGK----------------MERMRH--------NVV
CeApoL3-Like    246  -------------------------------..V...GSS.L...S.----------------..M..Q--------K..
CeMoma1         154  --------------------------------------------------------------T.AAFCY--------PIE
CeVit1I         622  -------------------------------MIIKVIR.EQEIVAQL---------------QN.EI.V--------K.D
CeVit1II        594  -------------------------------SLLVQVVSQMEKETNQ---------------QVAALT.--------QMI
CeVit2I         622  -------------------------------MIIKVIR.EQEIVAQL---------------QN.EI.V--------K.D
CeVit2II        592  -------------------------------SLLVQVVSQMEKETNQ---------------QVAALT.--------QMI
CeVit3I         623  -------------------------------LLVKVVRREEEIVVK.---------------SDDKFLV--------K.D
CeVit3II        591  -------------------------------PVLAHIVSQMENE.NQ---------------HVAAFTY--------..L
CeVit4I         623  -------------------------------LLVKVVRREEEIVVK.---------------SDDKFLV--------K.D
CeVit4II        591  -------------------------------PVLAHIVSQMENE.NQ---------------HVAAFTY--------..L
CeVit5I         623  -------------------------------LLVKVVRREEEIVVK.---------------SDDKFLV--------K.D
CeVit5II        591  -------------------------------PVLAHIVSQMENE.NQ---------------HVAAFTY--------..L
CeVit6I         629  -------------------------------KNVKVVY.ENEIEMY.---------------TE.GLIC--------R.N
CeVit6II        620  -------------------------------SVL.QIGNQL.QERNQ---------------QVRAFTL--------SLL
DmApoLppI      1869  LLSALEKYGPALKNYGKAIGEIVKPINDAAQEVIKIVVNAAEGVTHEFKQYVASLPSFESIRNEFNDKV--------K.L
DmApoLppII      547  -------------------------------EVANQISEIVNSETVN---------------QVGGFIS--------SNL
DmApoLTPI      2831  -------------------------------DQVQIMVQ.YY.VMA.VDWVADELQLEHRLHQVYGLVR--------.KF
DmApoLTPII      607  -------------------------------I.VGVIKSILEHEEIN---------------QVGSFVW--------SHL
DmCG31659       170  -------------------------------K.AGLVI.DMSKVPQE---------------------------------
DmCvD           974  -------------------------------NALMYIF.FTQLS.IH-------------LGHDRNFTMLMYNEKNTRIE
DmFabp          137  --------------------------------------------------------------------------------
DmGlaz          183  -------------------------------SKVYDVLKRLSLDPER---------------------------------
DmMic26-27      203  -------------------------------EDLKYM.SDLYDEAKD---------------------------------
DmNlaz          191  -------------------------------PRLDGNGTGLAGED.L---------------DVDDFVS-----------
DmMtp           636  -------------------------------EFLLHAGRSQASSFKL---------------GIYTAGLGSLVGDGDSGD
CeDSc4          637  -------------------------------QLF.TANTEFLQK.F.RSIFELSMKKGRKEHNLFSLSI--------DTE

CeApoL1         253  TNFLNYD-------------------------------------------------------------------------
CeApoL3-Like    271  KH...E.V---------WKDDDDVLSDVGS----------IEMVQPESP-------------------------------
CeMoma1         164  AVDVAKTG------------------------------------------------------------------------
CeVit1I         648  GKKILSED---------YSAHQIERLGESD----------IVIELPEGE-------------------------------
CeVit1II        620  RH.AKSTNPC-------YQRVAIVCSKVLS----------FTRYQPQEQ-------------------------------
CeVit2I         648  GKKIQSED---------YSAYQIERLGESA----------IVIELPEGE-------------------------------
CeVit2II        618  RH.AMSTNPC-------YQRVAIVCSKVLS----------FTRYQPQEQ-------------------------------
CeVit3I         649  EKKV.PTE---------LEQYNIEILGDNL----------IVIRLPHGE-------------------------------
CeVit3II        617  RQ.SKSTNPC-------YQQLAVRCSKVLL----------FTRYQPQEQ-------------------------------
CeVit4I         649  GKKV.PTE---------LEQYNIEILGDNL----------IVIRLPHGE-------------------------------
CeVit4II        617  RQ.SKSTNPC-------YQQLAVRCSKVLL----------FTRYQPQEQ-------------------------------
CeVit5I         649  GKKV.PTE---------LEQYNIEILGDNL----------IVIRLPQGE-------------------------------
CeVit5II        617  RQ.YKSTNPC-------YQQLAVRCSKILL----------FTRYQPQEQ-------------------------------
CeVit6I         655  GEEIE.QPESEIE----KKQYNIIWLNKNT----------LKFDSDDVT-------------------------------
CeVit6II        646  RSYA.NESPC-------EQTFSSRVQSLLN----------NIPFSSQEI-------------------------------
DmApoLppI      1941  KL.EKATELTNSL----FDQINILPQTPETSEFLQKLHDYLIAKLKQEHIDNEKYIEELGQLLIKAVRSIWVSIRSTYPG
DmApoLppII      573  KAIRDSTDVSRDQQKYHLANIRVTKTFPVD----------YRRYSFNNE-------------------------------
DmApoLTPI      2872  R.YAMNALETADM----YREAKTKFVFDPE----------VGIIDLEQKLPMSWHAFNETPRFEEIPEYQVLAKAQSFFS
DmApoLTPII      633  ..LAKSNSPVRIEAQGLLLNDELSERFKMD----------IRKFSRNYE-------------------------------
DmCG31659       186  --------------------------------------------------------------------------------
DmCvD          1010  G..CAE.VLKTSDMKGKQIGLTLEHTDHMNENHAADALHRWNITLDVLASTKSNWFKLTGQVQRNSKDDEDDWKACTKLT
DmFabp          137  --------------------------------------------------------------------------------
DmGlaz          199  --------------------------------------------------------------------------------
DmMic26-27      219  --------------------------------------------------------------------------------
DmNlaz          214  --------------------------------------------------------------------------------
DmMtp           670  G.DAIPADDEFSEDEAVTAGMEISVQGAQL----------RPLVFFSGQ-------------------------------
CeDSc4          678  HLEQFVTGSASSRSGAPQGSVRIGVAGHKL----------PTHHIFKGS-------------------------------

CeApoL1         260  --------------------------------------------------------------------------------
CeApoL3-Like    301  --------------------------------------------------------------------NQ----------
CeMoma1         172  --------------------------------------------------------------------------------
CeVit1I         678  -----VRFDGYTIKTQL-----------PSYSRKNQLCG-------------------LCGNNDDESTNEFYTSDNTETK
CeVit1II        652  -----MIASSYAQ---------------LPLFLQNSFSG-----------------------------AQFDFAAIFEKN
CeVit2I         678  -----VRFDGYTIKTQL-----------PSYSRKNQLCG-------------------LCGNNDDESTNEFYTSDNTETE
CeVit2II        650  -----MIASSYAQ---------------LPLFLQNSFSG-----------------------------AQFDFAAIFEKN
CeVit3I         679  -----VRFDGYTVKTNM-----------PSVASQNQLCG-------------------LCGNNDGERDNEFMTADNYETE
CeVit3II        649  -----ML-STYSQ---------------LPLFNSEWLSG-----------------------------VQFDFATIFEKN
CeVit4I         679  -----VRFDGYTVKTNM-----------PSVASQNQLCG-------------------LCGNNDGERDNEFMTADNYETE
CeVit4II        649  -----ML-STYSQ---------------LPLFNSEWLSG-----------------------------VQFDFATIFEKN
CeVit5I         679  -----VRFDGYTVKTNM-----------PSVASQNQLCG-------------------LCGNNDGERDNEFMTADNYETE
CeVit5II        649  -----ML-STYSQ---------------LPLFNSEWLSG-----------------------------VQFDFATIFEKN
CeVit6I         690  -----VQFDGVNARIH------------LSALYRNQQCG-------------------LCGHYDNEKETEFYDAENQENT
CeVit6II        678  -----DRFESVYGK--------------WSTYSRRHQSG-----------------------------FEANFASLFTTE
DmApoLppI      2017  SSDHVIDFQSWIGSLTHSFDSLAVLPSILSFRSSILNCL--LNENWDVVFNKKLLYSWIFFNDFELRGHVVDGKHIFTFD
DmApoLppII      612  ----------------------------VSYKLESLGVG-----------------------------ASTDYQIIYSQH
DmApoLTPI      2938  ETNSSIVMKLYNMRTHLDPKTWL-----PPYYSRALLIDSRHYMTFDQRYVGLNLNFDELGNGRSTSQCSYLLAHDFFKR
DmApoLTPII      672  ----------------------------HSLFFDEYNFG-----------------------------TTTDANVIFGTD
DmCG31659       186  --------------------------------------------------------------------------------
DmCvD          1090  YEPLVFTKRPHTLNGDVVFGLATEESECPEKGSTVQFAA---------------------RAGPSEHARAFLRSDKISLT
DmFabp          137  ----------------------------PTSVDDGARSG-----------------------------------------
DmGlaz          199  --------------------------------------------------------------------------------
DmMic26-27      219  --------------------------------------------------------------------------------
DmNlaz          214  --------------------------------------------------------------------------------
DmMtp           709  ----------------------------TELMGHVWGGS------------------------ASDSTPAYQATTLSQDN
CeDSc4          717  ----------------------------TDLLSTVWEAD--------------------------GRTHKAFEGHVPVRD
```

```
CeApoL1          260  --------------------------------------------------------------------------------
CeApoL3-Like     303  ----------------------SLIMDPHQQESSVQH-------------------------------------------
CeMoma1          172  -------------------------------RAHAEQ-------------------------------------------
CeVit1I          723  DIEEFHR---------------SYLLKNE--ECEAEEERLSEKK-------------------------------------
CeVit1II         683  ----------------------SFLLKDL--HASLDAVFGG---------------------------------------
CeVit2I          723  DIEEFHR---------------SYLLKNE--ECEAEEERLSEKKNYR---------------------------------
CeVit2II         681  ----------------------SFLPKDL--HASLDAVFGG---------------------------------------
CeVit3I          724  DVEEFHR---------------SYLLKNE--ECEVENDRISEKKNYRN--------------------------------
CeVit3II         679  ----------------------AFLPKEV--QASFETVFGG---------------------------------------
CeVit4I          724  DVEEFHR---------------SYLLKNE--ECEVEKDRISEKKNYKN--------------------------------
CeVit4II         679  ----------------------AFLPKEV--QASFETVFGG---------------------------------------
CeVit5I          724  DVEEFHR---------------SYLLKNE--ECEVENDRISEKKNYRN--------------------------------
CeVit5II         679  ----------------------AFLPKEV--QASFETVFGG---------------------------------------
CeVit6I          734  IPKFAK----------------SYLYKDS--KCNYEREMFEKEE------------------------------------
CeVit6II         710  ----------------------SVLPTEM--MASIEGVLSG---------------------------------------
DmApoLppI       2095  GLNFAYPGNC------------KYILAQD--SVDNNFTIIGQLTNGKLKSITLIDREGSYFEVADNLALKLNGNLVEYPQ
DmApoLppII       635  ----------------------GFLPRSS--RINVTTEFFGT--------------------------------------
DmApoLTPI       3013  NFTLLLEPASKSLAGQGLTRKLSFIANGQLIEIDLETDHISINGNPQPILPLKLGDVNIHRDLDVLSITSDTEFSLHCNV
DmApoLTPII       695  ----------------------SYLPRIA--SVNFTADLFGQSV------------------------------------
DmCG31659        186  -------------------------------SCPYDT-------------------------------------------
DmCvD           1149  DT--------------------DFCPKEVLKFSPIPTSRYCKRSNFENFTSITQYDMDLKFDNMPAWFE-----------
DmFabp           148  -------------------------------GCGQRPGGPGS--------------------------------------
DmGlaz           199  ----------------------LIISKNK--QCPEAL-------------------------------------------
DmMic26-27       219  ----------------------LIFPKK----------------------------------------------------
DmNlaz           214  ----------------------TTVPNAI--EKA?EWLRR-----------------------------------------
DmMtp            737  E---------------------HYIILTS--GATLHWRVLGARSVDLNGKVGFS--------------------------
CeDSc4           743  ----------------------VRLSVPLLSGLTLDVDSVGAISMRVLASAEVS--------------------------

CeApoL1          260  ------AWNSDI--------------------------------------------------------------------
CeApoL3-Like     318  --------------------------------------------------------------------------------
CeMoma1          178  ------T.Y.FQESP-----------------------------------------------------------------
CeVit1I          750  ------NYRKYDERK-----------------------------------------------------------------
CeVit1II         700  ------N..KYFAQI-----------------------------------------------------------------
CeVit2I          753  ------KYER.EEQS-----------------------------------------------------------------
CeVit2II         698  ------N..KYFAQI-----------------------------------------------------------------
CeVit3I          755  ------K..REEKKS-----------------------------------------------------------------
CeVit3II         696  ------N..KYFAQV-----------------------------------------------------------------
CeVit4I          755  ------K..REEKKS-----------------------------------------------------------------
CeVit4II         696  ------N..KYFAQV-----------------------------------------------------------------
CeVit5I          755  ------K..REEKKS-----------------------------------------------------------------
CeVit5II         696  ------N..KYFAQV-----------------------------------------------------------------
CeVit6I          760  ------NFQRIEKNQ-----------------------------------------------------------------
CeVit6II         727  ------E..QYFAQI-----------------------------------------------------------------
DmApoLppI       2161  HLSGLH..RRFYTIH---------------------------------------------------LYSEYGVGIVCTSD
DmApoLppII       653  ------NY.VFEASV-----------------------------------------------------------------
DmApoLTPI       3093  QFDL--C.FEVSGWYFGRTAGLLGTLNNEPYDEYTMSSGVISNETQLFTDSWSLKQCRQNKLAQTQEVSQEVSDACTSFF
DmApoLTPII       715  ------NFFEFTARA-----------------------------------------------------------------
DmCG31659        192  --------------------------------------------------------------------------------
DmCvD           1198  ------L.SNRLDHL--------------------------------------------------------VSALSADKV
DmFabp           159  --------------------------------------------------------------------------------
DmGlaz           212  --------------------------------------------------------------------------------
DmMic26-27       225  --------------------------------------------------------------------------------
DmNlaz           230  ------LYERLYDIF-----------------------------------------------------------------
DmMtp            768  ------L..RNAQTE-----------------------------------------------------------------
CeDSc4           775  ------L..QRSNAK-----------------------------------------------------------------

CeApoL1          266  --------------------------------------------------------------------------------
CeApoL3-Like     318  --------------------------------------------------------------------------------
CeMoma1          187  --------------------------------------------------------------------------------
CeVit1I          759  --------------------------------------------------------------------------------
CeVit1II         709  --------------------------------------------------------------------------------
CeVit2I          762  --------------------------------------------------------------------------------
CeVit2II         707  --------------------------------------------------------------------------------
CeVit3I          764  --------------------------------------------------------------------------------
CeVit3II         705  --------------------------------------------------------------------------------
CeVit4I          764  --------------------------------------------------------------------------------
CeVit4II         705  --------------------------------------------------------------------------------
CeVit5I          764  --------------------------------------------------------------------------------
CeVit5II         705  --------------------------------------------------------------------------------
CeVit6I          769  --------------------------------------------------------------------------------
CeVit6II         736  --------------------------------------------------------------------------------
DmApoLppI       2190  LKVCHININGFYTSKTRGLLGNGNAEPYDDFLLIDGTLAENSAALGNDYGVGKCTAIEFDNNQFKSSKRQEMCSELFGIE
DmApoLppII       662  --------------------------------------------------------------------------------
DmApoLTPI       3171  RTGILATCSAVLDPTPFYEMCMDLGMKSPPIRKGHPAVKGACAAALAYIEACTALKVPMRVPSQCVFCQLSNGSYVPEGT
DmApoLTPII       724  --------------------------------------------------------------------------------
DmCG31659        192  --------------------------------------------------------------------------------
DmCvD           1216  DSLHMSQEINISMQTPQDQFRLAVEVNGVKWRFHQIPFFYKLDSKFDASHELTFDSGLKRSCSVINGIVNTFDDYLINLR
DmFabp           159  --------------------------------------------------------------------------------
DmGlaz           212  --------------------------------------------------------------------------------
DmMic26-27       225  --------------------------------------------------------------------------------
DmNlaz           239  --------------------------------------------------------------------------------
DmMtp            777  --------------------------------------------------------------------------------
CeDSc4           784  --------------------------------------------------------------------------------
```

```
CeApoL1          266   --------------------------------------------------------------------------------
CeApoL3-Like     318   --------------------------------------------------------------------------------
CeMoma1          187   --------------------------------------------------------------------------------
CeVit1I          759   ----------------------------------------------------YESEEYSFEETYDYEQENTNKKQKNQRS
CeVit1II         709   --------------------------------------------------------------------------------
CeVit2I          762   -------------------------------------------------------DEYSSEETYDYEQENTKKSQKNQRS
CeVit2II         707   --------------------------------------------------------------------------------
CeVit3I          764   ----------------------------------------------------------DYVSSSDYENNYDEKETENQ--
CeVit3II         705   --------------------------------------------------------------------------------
CeVit4I          764   ----------------------------------------------------------DYESSSDYESNYDEKETEKE--
CeVit4II         705   --------------------------------------------------------------------------------
CeVit5I          764   ----------------------------------------------------------DYESSSDYESNYDEKETEKE--
CeVit5II         705   --------------------------------------------------------------------------------
CeVit6I          769   -----------------------------------------------------------------EEEKDQEMNYEESRR
CeVit6II         736   --------------------------------------------------------------------------------
DmApoLppI       2270   STLAFNFITLDSRPYRKACDIALAKVAEKEKEATACTFALAYGSAVKQINKWVLLPPRCIKCAGPAGQHDFGDEFTVKLP
DmApoLppII       662   --------------------------------------------------------------------------------
DmApoLTPI       3251   FMELSGPEIPKSSDVVFIVEAKECNANLKTSKNIMTVVSSIEEQLQAAKITNNRYAVVAFGGVSPYDKARSVIYEHNEFT
DmApoLTPII       724   --------------------------------------------------------------------------------
DmCG31659        192   --------------------------------------------------------------------------------
DmCvD           1296   EIAVRPDCLTLLVADCSPLPQIAVFVTPSPVQGLSTNYGLRVHIGQNYFNFRARTDNSSLPTDEPVLIYLNQDQTPHNVR
DmFabp           159   --------------------------------------------------------------------------------
DmGlaz           212   --------------------------------------------------------------------------------
DmMic26-27       225   --------------------------------------------------------------------------------
DmNlaz           239   --------------------------------------------------------------------------------
DmMtp            777   ----------------------------------------------------------IQQNTGSAVLGHLAVGFTYAKL
CeDSc4           784   ----------------------------------------------------------AEAYTSGSLHLTASLYHHSEPV

CeApoL1          266   --------------------------------------------------------------------------------
CeApoL3-Like     318   --------------------------------------------------------------------------------
CeMoma1          187   --------------------------------------------------------------------------------
CeVit1I          787   QKKSDLVEKTQIKEFSHRICFSVEPVAECRRRGYEAVEQQQRKVRFTCLPRHSSEARRLV--------------------
CeVit1II         709   --------------------------------------------------------------------------------
CeVit2I          787   QKKSDLVEKTQIKEFSHRICFSVEPVAEC-RRGYEVEQQQQRKIRFTCLQRHNRDASRL--------------------L
CeVit2II         707   --------------------------------------------------------------------------------
CeVit3I          784   -----LFKKTLIKEFSNRVCFSIEPVSEC-RRGLESEKTSNEKIRFTCMPRHSKNARRF--------------------L
CeVit3II         705   --------------------------------------------------------------------------------
CeVit4I          784   -----LVKKTLIKEFSNRVCFSIEPVSEC-RRGLESEKTSNKKIRFTCMPRHSKNARRF--------------------L
CeVit4II         705   --------------------------------------------------------------------------------
CeVit5I          784   -----LVKKTLIKEFSNRVCFSIEPVSEC-RRGLESEKTSNKKIRFTCMPRHSKNARRF--------------------L
CeVit5II         705   --------------------------------------------------------------------------------
CeVit6I          784   EQDDEPTEQVAIVERQHEICFTQKPVLRC--QNGKSQESKKQKTSVYCLPSSNSWARRQ--------------------M
CeVit6II         736   --------------------------------------------------------------------------------
DmApoLppI       2350   NNKVDVVFVVDINVTPGVLSNLIAPAINDIRESLRSRGFSDVQVGVIVFEETKRYPALLTSDGGKINYKGNVADVKLAGI
DmApoLppII       662   --------------------------------------------------------------------------------
DmApoLTPI       3331   SKPEQLADYFGHINTGNGSSNDILMAISAAAKLNFRPGVSKTFILLSCSKCAARDMRFD--------------------Y
DmApoLTPII       724   -----------------------EGLEELAANAFGPKGPLSGQLLRKKLSFLNRWLGNE---------------------
DmCG31659        192   --------------------------------------------------------------------------------
DmCvD           1376   KKPYQWPIETSDYDFRVELNEQNILIVECTQLSSTIQFDLYNILNFEIYGVYKHQ------------------------M
DmFabp           159   --------------------------------------------------------------------------------
DmGlaz           212   --------------------------------------------------------------------------------
DmMic26-27       225   --------------------------------------------------------------------------------
DmNlaz           239   --------------------------------------------------------------------------------
DmMtp            799   VQDFSITHEPKLSLNADLDFYSGIKLCMQLQRPEQLLKQTNVRSVFL---------------------------------
CeDSc4           806   RHVESTISALSTFTTDTRAIFETLPYDFCLRTSNSNVDINQKTVVQDQIGKHKKK-------------------------

CeApoL1          266   --------------------------------------------------------------------------------
CeApoL3-Like     318   --------------------------------------------------------------------------------
CeMoma1          187   ------TPSAIVK--------------------------------TN---------------------------------
CeVit1I          847   -KEARQGTVQLDD--------------------------------HK---------------------------------
CeVit1II         709   -GFSQQHMDKYVQ--------------------------------MA---------------------------------
CeVit2I          846   KESRQQPL-QLDD--------------------------------YP---------------------------------
CeVit2II         707   -GFSQQHMDKYVQ--------------------------------MA---------------------------------
CeVit3I          838   KEAREQTVADLVD--------------------------------FP---------------------------------
CeVit3II         705   -GFSQQNFEQVIL--------------------------------KT---------------------------------
CeVit4I          838   KEAREQTVADLVD--------------------------------FP---------------------------------
CeVit4II         705   -GFSQQNFEQVIL--------------------------------KT---------------------------------
CeVit5I          838   KEAREQTVAELVD--------------------------------FP---------------------------------
CeVit5II         705   -GFSQQNFEQVIL--------------------------------KT---------------------------------
CeVit6I          842   REIRREPLAQWPE--------------------------------HK---------------------------------
CeVit6II         736   -GFTQKNMEKIIK--------------------------------KL---------------------------------
DmApoLppI       2430   KSFCDNCVEQIITEKRILDIYNSLKEIVKGIAPQADEKAFQLALDYPFRAGAAKSIIGVRSDSLEYKNWWKFVRAQLTGS
DmApoLppII       662   ---RQENVEDVLE--------------------------------YY---------------------------------
DmApoLTPI       3391   TSILQYLLEEGVNLHILADTEFDFERNKKLRHFFGLDSKLVYSKRFP---------------------------------
DmApoLTPII       760   SAEEDDTLENLLS-------------------------------------------------------------------
DmCG31659        192   --------------------------------------------------------------------------------
DmCvD           1432   CGLCSKPLNRMQN--------------------------------YT---------------------------------
DmFabp           159   --------------------------------------------------------------------------------
DmGlaz           212   --------------------------------------------------------------------------------
DmMic26-27       225   ---------------------------------------------KP---------------------------------
DmNlaz           239   --------------------------------------------------------------------------------
DmMtp            846   -QSVDRPYAKHVR--------------------------------ST---------------------------------
CeDSc4           861   -TLNRKRVHPGVT--------------------------------YR---------------------------------
```

```
CeApoL1          266  --------------------------------------------------------------------------------
CeApoL3-Like     318  --------------------------------------------------------------------------------
CeMoma1          196  -------------------LSPPKS-------------------------------------------------------
CeVit1I          861  -------------------ISFVHS----------VQVPVACVAY-----------------------------------
CeVit1II         723  -------------------LEKLES----------IEKESTTVVRGR---------------------------------
CeVit2I          860  -------------------VSFVES----------VKVPTACVAYI----------------------------------
CeVit2II         721  -------------------LEKLES----------LEKESTTVVRGRI--------------------------------
CeVit3I          853  -------------------VSFVES----------VKIPTACVAY-----------------------------------
CeVit3II         719  -------------------LEKLSL----------YGKQSDE-LRSR---------------------------------
CeVit4I          853  -------------------VSFVES----------VKIPTACVAYP----------------------------------
CeVit4II         719  -------------------LEKLSL----------YGKQSDE-LRSRE--------------------------------
CeVit5I          853  -------------------VSFVES----------VKIPTACVAYP----------------------------------
CeVit5II         719  -------------------LEKLSL----------YGKQSDE-LRSRN--------------------------------
CeVit6I          857  -------------------LRNLRDQPQMEERTVRVAVDQKCDKFDYS--------------------------------
CeVit6II         750  -------------------LSNVQ-----------EKGLEQIVVRGKV--------------------------------
DmApoLppI       2510  ITKFDGALIHLIAPVKGLSLEGVLSEKLIGFNSRLVATVDGKDSKKRTKLQFDNDMGIDFVLNNGGWVFATQNFEKLKAS
DmApoLppII       674  -------------------LGPKGL----------VNKDFDEIVKLIEVGNNGVAAGGRARR------------------
DmApoLTPI       3438  -------------------EGDAETRNTTHIPKSNLGICTTLAVETQGSVFSARKLQPERKYPIKRFATIFAKRVALSAT
DmApoLTPII       773  -------------------LDNLRLK------------------------------------------------------
DmCG31659        192  --------------------------------------------------------------------------------
DmCvD           1447  -------------------ICELEANTPTPVP---LQNSSDVVVVA----------------------------------
DmFabp           159  --------------------------------------------------------------------------------
DmGlaz           212  --------------------------------------------------------------------------------
DmMic26-27       227  --------------------------------------------------------------------------------
DmNlaz           239  -------------------MNFLSY-------------------------------------------------------
DmMtp            860  -------------------LSHKTAGCTFALN---QKNNEMCNLIFRDL-------------------------------
CeDSc4           875  -------------------LDDST-----------IRQCNSYLEQFRL--------------------------------

CeApoL1          266  --------------------------------------------------------
CeApoL3-Like     318  --------------------------------------------------------
CeMoma1          202  --------------------------------------------------------
CeVit1I          877  --------------------------------------------------------
CeVit1II         741  --------------------------------------------------------
CeVit2I          877  --------------------------------------------------------
CeVit2II         740  --------------------------------------------------------
CeVit3I          869  --------------------------------------------------------
CeVit3II         736  --------------------------------------------------------
CeVit4I          870  --------------------------------------------------------
CeVit4II         737  --------------------------------------------------------
CeVit5I          870  --------------------------------------------------------
CeVit5II         737  --------------------------------------------------------
CeVit6I          886  --------------------------------------------------------
CeVit6II         768  --------------------------------------------------------
DmApoLppI       2590  DQKKMLNQITSSLADTLFKTEIVSDCRCLPIHGLHGQHKCVIKSSTFVANKKAKSA
DmApoLppII       707  --------------------------------------------------------
DmApoLTPI       3499  PIQSQTCECSAHNTGVSYMACSPQALPEEKYDLDDYDSFNNWDWGDEPESETNVMS
DmApoLTPII       780  --------------------------------------------------------
DmCG31659        192  --------------------------------------------------------
DmCvD           1471  --------------------------------------------------------
DmFabp           159  --------------------------------------------------------
DmGlaz           212  --------------------------------------------------------
DmMic26-27       227  --------------------------------------------------------
DmNlaz           245  --------------------------------------------------------
DmMtp            887  --------------------------------------------------------
CeDSc4           893  --------------------------------------------------------
```

Fig. S12. Alignment of 25 human, 11 fly and 14 nematode sequences with ApoB, ApoLpp and ApoLTP separated plus all outgroups.

```
HsApoA1          0   ----------------------------------------------------------------------
HsApoA2          0   ----------------------------------------------------------------------
HsApoA4          0   ----------------------------------------------------------------------
HsApoA5          0   ----------------------------------------------------------------------
HsApoB48         0   ----------------------------------------------------------------------
HsApoB52         0   ----------------------------------------------------------------------
HsApoC1          0   ----------------------------------------------------------------------
HsApoC2          0   ----------------------------------------------------------------------
HsApoC3          0   ----------------------------------------------------------------------
HsApoC4          0   ----------------------------------------------------------------------
HsApoD           0   ----------------------------------------------------------------------
HsApoE2          0   ----------------------------------------------------------------------
HsApoF           0   ----------------------------------------------------------------------
HsApoH           0   ----------------------------------------------------------------------
HsApoJ           0   ----------------------------------------------------------------------
HsApoL1          0   ----------------------------------------------------------------------
HsApoL2          0   ----------------------------------------------------------------------
HsApoL3          0   ----------------------------------------------------------------------
HsApoL4          0   ----------------------------------------------------------------------
HsApoOL(2)       0   ----------------------------------------------------------------------
HsApoL6          0   ----------------------------------------------------------------------
HsApoM           0   ----------------------------------------------------------------------
HsApoO           0   ----------------------------------------------------------------------
HsApoOL          0   ----------------------------------------------------------------------
HsMTTP           0   ----------------------------------------------------------------------
DmCG31659        0   ----------------------------------------------------------------------
DmMtp            0   ----------------------------------------------------------------------
DmCvD            0   ----------------------------------------------------------------------
DmGlaz           0   ----------------------------------------------------------------------
DmFabp           0   ----------------------------------------------------------------------
DmApoLTPII       0   ----------------------------------------------------------------------
DmApoLTPI        1   RKAQEQARASEEEVDEDYDFEESLEGNKRQKRDVSTTRKQEIDRNVDSLGYKLKYDYNNPRAQFGLRVFG
DmNlaz           0   ----------------------------------------------------------------------
DmApoLII         0   ----------------------------------------------------------------------
DmApoLI          0   ----------------------------------------------------------------------
DmMICOS_A        0   ----------------------------------------------------------------------
Cel_MICOS        0   ----------------------------------------------------------------------
Cel_Vit_2        0   ----------------------------------------------------------------------
Cel_Vit_4        0   ----------------------------------------------------------------------
Cel_Vit_5        0   ----------------------------------------------------------------------
Cel_ApoL_1       0   ----------------------------------------------------------------------
Cel_MTTP         0   ----------------------------------------------------------------------
Cel_Vit_6        0   ----------------------------------------------------------------------
Cel_Vit_3        0   ----------------------------------------------------------------------
Cel_Vit-1        0   ----------------------------------------------------------------------
Cel_Apo_L3_Like  0   ----------------------------------------------------------------------

HsApoA1          0   ----------------------------------------------------------------------
HsApoA2          0   ----------------------------------------------------------------------
HsApoA4          1   ----------------------------------------------MFLKAVVLTLALVAVAGARAEVSA
HsApoA5          1   --------------------------------------------------MASMAAVLTWALALLSAFSA
HsApoB48         1   ----------------------------------MDPPRPALLALLALPALLLLLLAGARAEEEMLENVS
HsApoB52         1   ---------------------------------------FDQYIKDSYDLHDLKIAIANIIDEIIEKLKS
HsApoC1          0   ----------------------------------------------------------------------
HsApoC2          0   ----------------------------------------------------------------------
HsApoC3          0   ----------------------------------------------------------------------
HsApoC4          0   ----------------------------------------------------------------------
HsApoD           0   ----------------------------------------------------------------------
HsApoE2          1   ----------------------------------------------------MKVLWAALLVTFLAGCQA
HsApoF           1   -------------------------------------------------------------MTGLCGYSA
HsApoH           0   ----------------------------------------------------------------------
HsApoJ           1   ---------------------------------------------MMKTLLLFVGLLLTWESGQVLGDQT
HsApoL1          1   ---------------------------------------MEGAALLRVSVLCIWMSALFLGVGVRAEEAG
HsApoL2          0   ----------------------------------------------------------------------
HsApoL3          0   ----------------------------------------------------------------------
HsApoL4          1   ----------------------------------------------------------MGSWVQLITSVG
HsApoOL(2)       1   ----------------------------------------------MPCGKQGNLQVPGSKVLPGLGEGC
HsApoL6          0   ----------------------------------------------------------------------
HsApoM           0   ----------------------------------------------------------------------
HsApoO           0   ----------------------------------------------------------------------
HsApoOL          0   ----------------------------------------------------------------------
HsMTTP           1   ------------------------------------------MILLAVLFLCFISSYSASVKGHTTGLSL
DmCG31659        0   ----------------------------------------------------------------------
DmMtp            1   --------------------------------------------MENKNKKCLRTLLLLALFLGLLEDGK
DmCvD            1   -------------------------------------------MRLLKCSVFVYLFLIFDAGHAFSIIGL
DmGlaz           0   ----------------------------------------------------------------------
DmFabp           0   ----------------------------------------------------------------------
DmApoLTPII       1   -------------------------------------------MGGLKPQAAIWLLLLIAHTQAVRENPL
DmApoLTPI        71  NDLRYFNVESLVEVMALAAKFNPFQQAKNVLSGKEFTYTKSRVFLDASYTVPLAVGLPLAIHAFGASSID
DmNlaz           0   ----------------------------------------------------------------------
DmApoLII         1   -------------------------------------MARMKYNIALIGILASVLLTIAVNAENACNLGC
DmApoLI          1   -----------------------------------------SIVDDVSKISKKYKMYGVKNVQDLNLDVS
DmMICOS_A        0   ----------------------------------------------------------------------
Cel_MICOS        0   ----------------------------------------------------------------------
Cel_Vit_2        1   ----------------------------------------------MRSIIIASLVALALASSP-----A
Cel_Vit_4        1   ----------------------------------------------MKSIIIASLVALAIAASP-----A
Cel_Vit_5        1   ----------------------------------------------MKSIIIASLVALAIAASP-----A
Cel_ApoL_1       0   ----------------------------------------------------------------------
Cel_MTTP         1   --------------------------------------------------MFSSRIWLLLAVTVGVCLAV
Cel_Vit_6        1   ----------------------------------------------MKFFIALALLGAALASTHLDRYNS
Cel_Vit_3        1   ----------------------------------------------MKSIIIASLVALAIAASP-----A
Cel_Vit-1        1   ----------------------------------------------MRSIIIASIVALAIAFSP-----A
Cel_Apo_L3_Like  0   ----------------------------------------------------------------------
```

```
HsApoA1          0    ----------------------------------------------------------------------
HsApoA2          0    ----------------------------------------------------------------------
HsApoA4          25   DQVATVMWDYFSQLSNNAK---------------------------------------------------
HsApoA5          21   TQARKGFWDYFSQTSGDKGRVEQIH---------------------------------------------
HsApoB48         37   LVCPKDATRFKHLRKYTYNYEAESSSGVPGTADSRSATRINCK---------------------------
HsApoB52         32   LDEHYHIRVNLVKTIHDLHLFIENIDFNKSGSSTASWIQNVDTKYQIRIQIQEKLQQLKRHIQNIDI---
HsApoC1          0    ----------------------------------------------------------------------
HsApoC2          0    ----------------------------------------------------------------------
HsApoC3          0    ----------------------------------------------------------------------
HsApoC4          0    ----------------------------------------------------------------------
HsApoD           0    ----------------------------------------------------------------------
HsApoE2          19   KVEQAVETEPEPELRQQTEWQS------------------------------------------------
HsApoF           10   PDMRGLRLIMIP----------------------------------------------------------
HsApoH           0    ----------------------------------------------------------------------
HsApoJ           26   VSDNELQEMSNQGSKYVNKEIQNAVNGV------------------------------------------
HsApoL1          32   ARVQQNVPSGTDTGDPQSKPLGDWAAGT------------------------------------------
HsApoL2          0    ----------------------------------------------------------------------
HsApoL3          0    ----------------------------------------------------------------------
HsApoL4          13   VQQNHPGWTVAGQFQEKKRFTEEVIE--------------------------------------------
HsApoOL(2)       25   KEMWLRKVIYGGEVWGKSPEPE------------------------------------------------
HsApoL6          0    ----------------------------------------------------------------------
HsApoM           0    ----------------------------------------------------------------------
HsApoO           0    ----------------------------------------------------------------------
HsApoOL          0    ----------------------------------------------------------------------
HsMTTP           29   NNDRLYKLTYSTEVLLDRGKGKLQDSVGYRISSNVDVALLW-----------------------------
DmCG31659        0    ----------------------------------------------------------------------
DmMtp            27   TALIAPNSQQIFKLQNQVILQELGRD--------------------------------------------
DmCvD            28   NKQMLYEYEGNVLVGAKPQDEGHQ----------------------------------------------
DmGlaz           0    ----------------------------------------------------------------------
DmFabp           0    ----------------------------------------------------------------------
DmApoLTPII       28   KDPRICGRPQCDSKSAKFNYGEQLYKYQYTVAVRTEFAGSGDNSSDLLLKSDLEIFFPKPCEGYLRINDA
DmApoLTPI        141  LRVSGNLDEMDPPTDWHFDVEGQFKPSVSVDVITTMQTDMFWEQSGIKVKSNLYSNSELVAKLKVRGRNL
DmNlaz           0    ----------------------------------------------------------------------
DmApoLII         34   PKSDNGLLKYIPGNYYDYSFDSILTIGASSD---------------------------------------
DmApoLI          30   LKLFGSELAFLSLGDNIPSSLDDIINYFSTSFEKAKQELSSFEKQFSSHHLFLDTDLAYPTSIGVPLELV
DmMICOS_A        0    ----------------------------------------------------------------------
Cel_MICOS        0    ----------------------------------------------------------------------
Cel_Vit_2        20   FER-----TFEPKTDYHYKFDGLVLSGLPSASSELSQSRISARAR-------------------------
Cel_Vit_4        20   LDR-----TFSPKSEYVYKFDGLLLSGLPTTFSDASQTLISCRTR-------------------------
Cel_Vit_5        20   LDR-----TFSPKSEYVYKFDGLLLSGLPTASSDASQTLISCRTR-------------------------
Cel_ApoL_1       0    ----------------------------------------------------------------------
Cel_MTTP         21   PDLDEIKKNLRKHGPDYYKNQPKMNENTVRLLKVDYWFRTESM---------------------------
Cel_Vit_6        25   IERNIQESSFRAGREYRYLFNGQLSAGLPVPSTPQGISRLQSQVT-------------------------
Cel_Vit_3        20   LDR-----TFSPKSEYVYKFDGLLLSGLPTTFSDASQTLISCRTR-------------------------
Cel_Vit-1        20   FER-----TFEPKIDYHYKFDGLVLSGLPTASSELSQSRFSARVR-------------------------
Cel_Apo_L3_Like  0    ----------------------------------------------------------------------

HsApoA1          0    ----------------------------------------------------------------------
HsApoA2          0    ----------------------------------------------------------------------
HsApoA4          43   ----------------------------------------------------------------------
HsApoA5          45   ----------------------------------------------------------------------
HsApoB48         79   -----------VELEVPQLCSFILKTSQCTLKEVYGFNPEGKALLKKTKNSEEFAAAMSRYELKLAIPEG
HsApoB52         98   --------QHLAGKLKQHIEAIDVRVLLDQLGTTISFERINDILEHVKHFVINLIGDFEVAEKINAFRAK
HsApoC1          0    ----------------------------------------------------------------------
HsApoC2          0    ----------------------------------------------------------------------
HsApoC3          0    ----------------------------------------------------------------------
HsApoC4          0    ----------------------------------------------------------------------
HsApoD           0    ----------------------------------------------------------------------
HsApoE2          40   -------------------------------------------------------------------GQR
HsApoF           21   ----------------------------------------------------------------------
HsApoH           1    -----------------------------------------------------MISPVLILFSSFLCHVA
HsApoJ           53   -------------------------------------------KQIKTLIEKTNEERKTLLSNLEEAKKK
HsApoL1          59   --------------------------------------------MDPESSIFIEDAIKYFKEKVSTQNLL
HsApoL2          1    --------------------------------------------MNPESSIFIEDYLKYFQDQVSRENLL
HsApoL3          1    --------------------------------------------MDSEKKRFTEEATKYFRERVSPVHLQ
HsApoL4          38   ----------------------------------------------------------YFQKKVSPVHLK
HsApoOL(2)       46   -------------------------------------------------------------------FPS
HsApoL6          0    ----------------------------------------------------------------------
HsApoM           0    ----------------------------------------------------------------------
HsApoO           0    ----------------------------------------------------------------------
HsApoOL          0    ----------------------------------------------------------------------
HsMTTP           69   --------RNPDGDDDQLIQITMKDVNVENVNQQRGEKSIFKGKSPSKIMGKENLEALQRPTLLHLIHGK
DmCG31659        0    ----------------------------------------------------------------------
DmMtp            52   --------SSSAETSYTFETDLKINSVWSGDEDQLLEVFISGSKVDASGKARSITRIPDRPFYISLVRGQ
DmCvD            51   --------APPTTGWIVRGKLTLQRQSELVLAAALVIDDVTLNNSGEKFLQNKEMYPPYKPFKIALTKDG
DmGlaz           0    ----------------------------------------------------------------------
DmFabp           0    ----------------------------------------------------------------------
DmApoLTPII       98   KLY-----DTLDELNDNESDSSEKTKEYDYYDNLANEESESQNYDNMHPKSSDFNVDLTKNLLRFAFHDG
DmApoLTPI        211  VSFSFDLPRDKNEIFSVRSELLVQKREEQLPQAGIANRSANSTCTWPVLDQAIGLQMCSHYSVPDLSNAT
DmNlaz           0    ----------------------------------------------------------------------
DmApoLII         64   --------VPNDSDDTSLKVSGSAKIFAKGNCGYTLQLSSVKVTNTKESVEKKILNSIQKPVQFTLVSGI
DmApoLI          100  AQGFAATKVDLAVSLDINAILEQNWQKAKYRLKFVPSVDINANVQIGFNAQVLSTGLRVVSSAHSATGSD
DmMICOS_A        0    ----------------------------------------------------------------------
Cel_MICOS        0    ----------------------------------------------------------------------
Cel_Vit_2        59   --------IQAVDDRYIHLQLVNIRMAASHL-PESEQMPSLNSMEQRE-LSEEYKQMLELPLRAQLRNGL
Cel_Vit_4        59   --------LQAVDDRYIHLQLIDIQYSASHI-PQSEQWPKIESLEQRE-LSDELKELLELPFRAQIRNGL
Cel_Vit_5        59   --------LQAVDDRYIHLQLTDIQYSASHI-PQSEQWPKIESLEQRE-LSDEFKELLELPFRAQIRNGL
Cel_ApoL_1       0    ----------------------------------------------------------------------
Cel_MTTP         63   --------IYDDIDNKEKDPSTVIAGNFSFETLHHDVEGGMLGRFTLTQCNTDNCGNPSPIYIAFRQGGN
Cel_Vit_6        69   --------LQWTDGNTVRMQLQKTRFATSQQESNSMKMLPFERFEEVERMNREHQELLSMPVEFDYEHGL
Cel_Vit_3        59   --------LQAVDDRYIHLQLIDIQYSASHI-PQSEQWPKIKSLEQRE-LSDELKELLELPFRAQIRNGL
Cel_Vit-1        59   --------IQAVDDRHIHLQLVNIHMAASHL-PESEQIPSLNSMEQRE-LSEEYKQMLKLPLRAQLRNGL
Cel_Apo_L3_Like  0    ----------------------------------------------------------------------
```

```
HsApoA2          1    ------------------MKLLA-----------------------------------------------
HsApoA4          43   ---EAVEHLQKSELTQQLNALFQDKLGEVNTYAGDL----------------------------------
HsApoA5          45   ---QQKMAREPATLKDSLEQDLNNMNKF------------------------------------------
HsApoB48         139  KQVFLYPEKDEPTYILNIKRGIISALLVPPETEEAKQVLFLD----------------------------
HsApoB52         161  VHELIERYEVDQQIQVLMDKLVELAHQYKLKETIQKLSNVLQ----------------------------
HsApoC1          1    -----------MRLFLSLPVLVVVLSIV------------------------------------------
HsApoC2          1    -------------MGTRLLPALFLVL--------------------------------------------
HsApoC3          1    ----------------MQPRVLL-----------------------------------------------
HsApoC4          1    -------MSLLRNRLQALPALCLCVLVL------------------------------------------
HsApoD           0    ----------------------------------------------------------------------
HsApoE2          44   WELALGRFWDYLRWVQTLSEQVQEELLSSQV---------------------------------------
HsApoF           22   VELLLCYLLLHPVDATSYGKQTNVLMHFPLS---------------------------------------
HsApoH           18   IAGRTCPKPDDLPFSTVVPLKTFYE---------------------------------------------
HsApoJ           81   KEDALNETRESETKLKELPGVCNETMMALWEECKPCLKQT------------------------------
HsApoL1          86   LLLTDNEAWNGFVAAAELPRNEADELRKA-----------------------------------------
HsApoL2          27   QLLTDDEAWNGFVAAAELPRDEADELRKA-----------------------------------------
HsApoL3          27   ILLTNNEAWKRFVTAAELPRDEADALYEA-----------------------------------------
HsApoL4          51   ILLTSDEAWKRFVRVAELPREEADALYEA-----------------------------------------
HsApoOL(2)       50   LV-NLCQSWKINNLMSTVHSDEAGMLSY------------------------------------------
HsApoL6          1    -------------MDNQAERESEAGVGLQ-----------------------------------------
HsApoM           1    ----------------MFHQIWAALLYFYG----------------------------------------
HsApoO           1    -------------MFKVIQRSVGPASLS------------------------------------------
HsApoOL          1    ---------MAAIRMGKLTTMPAGLIYAS-----------------------------------------
HsMTTP           132  VK-EFYSYQNEAVAIENIKRGLASLFQTQLSSGTTNEVDISG----------------------------
DmCG31659        1    ----------MIKWKIFVPAILYLQSSM------------------------------------------
DmMtp            115  PD-KVIAHTSKDQSLLNLERGIASLLQLRLDASQEEELDVSG----------------------------
DmCvD            114  AISHVVFKEGDPIWSMNFKRAIASVLQFQMKSSGAFVVDELG----------------------------
DmGlaz           1    ----MMSGQPLGSRVWLLSGVLLVTFAG------------------------------------------
DmFabp           0    ----------------------------------------------------------------------
DmApoLTPII       163  LISEVCPQEQETPWVLNIKKGILSAFQNTMMRFDVDANT-------------------------------
DmApoLTPI        281  EIYPSLLLAGPLNFSLILKKSDLSAKKYVFEYKWDQQEEDNNFSLVFTTPGSKVPRVLVANVTKVPDAFN
DmNlaz           1    ------MNHHSSSHLLLLISVVFGAVWVAH----------------------------------------
DmApoLII         127  LEPQICSDSSDLDYSLNIKRAVVSLLQSGIEAE-------------------------------------
DmApoLI          170  ITVAVISDGEGFNVDLELPREKLELINFNVDTELYVAEQDKQ----------------------------
DmMICOS_A        1    ----------------MLRKTAT-----------------------------------------------
Cel_MICOS        0    ----------------------------------------------------------------------
Cel_Vit_2        120  IS-ELQFDKEDAEWSKNMKRAVVNMISFNPIAPRNEIEKIES----------------------------
Cel_Vit_4        120  VS-EIQFSSEDAEWSKNAKRSILNLFSLRKSAPVDEMSQDQ-----------------------------
Cel_Vit_5        120  IS-EIQFSSEDAEWSKNAKRSILNLFSLRKSAPVDEMNQDQ-----------------------------
Cel_ApoL_1       1    ---------------MSI----------------------------------------------------
Cel_MTTP         126  NAEHILKASDESDATWNFLYAIVNTIYTPAEYGEGDEQTVDT----------------------------
Cel_Vit_6        132  VR-EIRFAENDQPWSENIKRAVINMLQ------VNILKKEKY----------------------------
Cel_Vit_3        120  VS-EIQFSSEDAEWSKNAKRSILNLFSLRKSAPVDEMSQDQ-----------------------------
Cel_Vit-1        120  IA-ELQFDKEDAEWSKNMKRAVVNMISFNPIAPRNEIEKIES----------------------------
Cel_Apo_L3_Like  1    ---MQIKDDHDDAGSQSEDNGVPI----------------------------------------------

HsApoA1          8    ----------------------------------------------------------------------
HsApoA2          5    ----------------------------------------------------------------------
HsApoA4          76   ----------------------------------------------------------------------
HsApoA5          70   ----------------------------------------------------------------------
HsApoB48         180  ----------------------------------------------------------------------
HsApoB52         202  ----------------------------------------------------------------------
HsApoC1          17   ----------------------------------------------------------------------
HsApoC2          13   ----------------------------------------------------------------------
HsApoC3          7    ----------------------------------------------------------------------
HsApoC4          21   ----------------------------------------------------------------------
HsApoD           0    ----------------------------------------------------------------------
HsApoE2          74   ----------------------------------------------------------------------
HsApoF           52   ----------------------------------------------------------------------
HsApoH           42   ----------------------------------------------------------------------
HsApoJ           120  ----------------------------------------------------------------------
HsApoL1          114  ----------------------------------------------------------------------
HsApoL2          55   ----------------------------------------------------------------------
HsApoL3          55   ----------------------------------------------------------------------
HsApoL4          79   ----------------------------------------------------------------------
HsApoOL(2)       76   ----------------------------------------------------------------------
HsApoL6          16   ----------------------------------------------------------------------
HsApoM           14   ----------------------------------------------------------------------
HsApoO           15   ----------------------------------------------------------------------
HsApoOL          20   ----------------------------------------------------------------------
HsMTTP           172  ----------------------------------------------------------------------
DmCG31659        18   ----------------------------------------------------------------------
DmMtp            155  ----------------------------------------------------------------------
DmCvD            155  ----------------------------------------------------------------------
DmGlaz           24   ----------------------------------------------------------------------
DmFabp           0    ----------------------------------------------------------------------
DmApoLTPII       201  ----------------------------------------------------------------------
DmApoLTPI        351  ASVAFVNGPNRVSAGCSYDGNPDFRRLDIYLDTNGNRSLDLGMELRRHQDFTAWIYNPRMLLAINGVNIT
DmNlaz           24   ----------------------------------------------------------------------
DmApoLII         159  ----------------------------------------------------------------------
DmApoLI          211  ----------------------------------------------------------------------
DmMICOS_A        7    ----------------------------------------------------------------------
Cel_MICOS        0    ----------------------------------------------------------------------
Cel_Vit_2        160  ----------------------------------------------------------------------
Cel_Vit_4        159  ----------------------------------------------------------------------
Cel_Vit_5        159  ----------------------------------------------------------------------
Cel_ApoL_1       3    ----------------------------------------------------------------------
Cel_MTTP         167  ----------------------------------------------------------------------
Cel_Vit_6        166  ----------------------------------------------------------------------
Cel_Vit_3        159  ----------------------------------------------------------------------
Cel_Vit-1        160  ----------------------------------------------------------------------
Cel_Apo_L3_Like  21   ----------------------------------------------------------------------
```

```
HsApoA2        5    ----------------------------------------------------------------------
HsApoA4        76   ----------------------------------------------------------------------
HsApoA5        70   ----------------------------------------------------------------------
HsApoB48       181  TVYGNCSTHFTVKTRKGNVATEISTERDLGQCDRFKPIRTGISPLALIKGMTRPLSTLISSSQSCQYTLD
HsApoB52       202  ------QVKIKDYFEKLVGFIDDAVKKLNELSFKTFIEDVNKFLDMLIKKLKSFDYHQFVDETNDKIREV
HsApoC1        17   ----------------------------------------------------------------------
HsApoC2        13   ----------------------------------------------------------------------
HsApoC3        7    ----------------------------------------------------------------------
HsApoC4        21   ----------------------------------------------------------------------
HsApoD         0    ----------------------------------------------------------------------
HsApoE2        74   ----------------------------------------------------------------------
HsApoF         52   ----------------------------------------------------------------------
HsApoH         42   ----------------------------------------------------------------------
HsApoJ         120  ----------------------------------------------------------------------
HsApoL1        114  ----------------------------------------------------------------------
HsApoL2        55   ----------------------------------------------------------------------
HsApoL3        55   ----------------------------------------------------------------------
HsApoL4        79   ----------------------------------------------------------------------
HsApoOL(2)     76   ----------------------------------------------------------------------
HsApoL6        16   ----------------------------------------------------------------------
HsApoM         14   ----------------------------------------------------------------------
HsApoO         15   ----------------------------------------------------------------------
HsApoOL        20   ----------------------------------------------------------------------
HsMTTP         172  ------NCKVTYQAHQDKV---------------------------------------------------
DmCG31659      18   ----------------------------------------------------------------------
DmMtp          155  ------LCRVSYNVKSSTKVEKTKRDCSLWD---------------------------------------
DmCvD          155  ------IHGTCRTEYFVSNRTNYISIRKTPEVKTCKPYSEAVHTT-------------------------
DmGlaz         24   ----------------------------------------------------------------------
DmFabp         0    ----------------------------------------------------------------------
DmApoLTPII     201  ----------------------------------------------------------------------
DmApoLTPI      421  GLAGTVKVNEKNGIKQHDVDLSFETKKLQAVIKGNVVQSEITTSTNMTIKYRFQANKIEEINFAGKLVNN
DmNlaz         24   ----------------------------------------------------------------------
DmApoLII       159  ----------------------------------------------------------------------
DmApoLI        211  ------KAIALKGNKKNKNSQPSEICFNQLELVGLNICIKSSTSLSEVQAGNGNVAERGLSVSEKFHLSR
DmMICOS_A      7    ----------------------------------------------------------------------
Cel_MICOS      0    ----------------------------------------------------------------------
Cel_Vit_2      160  ------SYDKEEQSEENTSFFT-NEKTLEGDCQVAYTV--IREQKKTIITKSINFDKCTERSEIAYGL--
Cel_Vit_4      159  ---------KDMESDKDSLFFNVHEKTMEGDCEVAYTI--VQEGGKTIYTKSVNFDKCITRPETAYGL--
Cel_Vit_5      159  ---------KDMESDKDSLFFNVHEKTMEGDCEVAYTI--VQEGEKTIYTKSVNFDKCITRPETAYGL--
Cel_ApoL_1     3    ----------------------------------------------------------------------
Cel_MTTP       167  ------IYGRCFVNFGRPEDKRFRRIIEKCD---------------------------------------
Cel_Vit_6      166  ------EGAEKSDNQEPTFSFTNVERTLEGECEVLYTVEEIKKEDEQRWAKSINFDKCTRRPYIHH----
Cel_Vit_3      159  ---------KDMESDKDSLFFNVHEKTMEGDCEVAYTI--VQEGEKTIYTKSVNFDKCITRPETAYGL--
Cel_Vit-1      160  ------SYDKEEQSEENTSFFT-NEKTLEGDCQVAYTV--IREQKKTIITKSINFDKCTERSEIAYGL--
Cel_Apo_L3_Like 21  ----------------------------------------------------------------------

HsApoA1        8    ----------------------------------------------------------------------
HsApoA2        5    ----------------------------------------------------------------------
HsApoA4        76   ----------------------------------------------------------------------
HsApoA5        70   ----------------------------------------------------------------------
HsApoB48       251  AK--------------------------------------------------------------------
HsApoB52       267  TQRLNGEIQALELPQKAEALKLFLEETKATVAVYLESLQDTKITLIINWLQEALS---------------
HsApoC1        17   ----------------------------------------------------------------------
HsApoC2        13   ----------------------------------------------------------------------
HsApoC3        7    ----------------------------------------------------------------------
HsApoC4        21   ----------------------------------------------------------------------
HsApoD         0    ----------------------------------------------------------------------
HsApoE2        74   ----------------------------------------------------------------------
HsApoF         52   ----------------------------------------------------------------------
HsApoH         42   ----------------------------------------------------------------------
HsApoJ         120  ----------------------------------------------------------------------
HsApoL1        114  ----------------------------------------------------------------------
HsApoL2        55   ----------------------------------------------------------------------
HsApoL3        55   ----------------------------------------------------------------------
HsApoL4        79   ----------------------------------------------------------------------
HsApoOL(2)     76   ----------------------------------------------------------------------
HsApoL6        16   ----------------------------------------------------------------------
HsApoM         14   ----------------------------------------------------------------------
HsApoO         15   ----------------------------------------------------------------------
HsApoOL        20   ----------------------------------------------------------------------
HsMTTP         185  ----------------------------------------------------------------------
DmCG31659      18   ----------------------------------------------------------------------
DmMtp          180  ----------------------------------------------------------------------
DmCvD          194  ----------------------------------------------------------------------
DmGlaz         24   ----------------------------------------------------------------------
DmFabp         1    ---------------------------------------------------------------------M
DmApoLTPII     201  ----------------------------------------------------------------------
DmApoLTPI      491  GDKSKTEYRGNMKLQTSAYPKLNFASESTWLSLQGHTEGMITYNNAPDYVNPNYTSLVRLIFARSHSEDS
DmNlaz         24   ----------------------------------------------------------------------
DmApoLII       159  ----------------------------------------------------------------------
DmApoLI        276  PFNFAVYLTTERKFTFKGIHTQEAFSQKWKLDYSTPGSKVSHDTTVVYELGNKPKTFSRLS---------
DmMICOS_A      7    ----------------------------------------------------------------------
Cel_MICOS      0    ----------------------------------------------------------------------
Cel_Vit_2      219  ----------------------------------------------------------------------
Cel_Vit_4      216  ----------------------------------------------------------------------
Cel_Vit_5      216  ----------------------------------------------------------------------
Cel_ApoL_1     3    ----------------------------------------------------------------------
Cel_MTTP       192  ----------------------------------------------------------------------
Cel_Vit_6      226  ----------------------------------------------------------------------
Cel_Vit_3      216  ----------------------------------------------------------------------
Cel_Vit-1      219  ----------------------------------------------------------------------
Cel_Apo_L3_Like 21  ----------------------------------------------------------------------
```

```
HsApoA2           6    ATVLLLTICSLEGALVR-----------------------------------------------------
HsApoA4           77   QKKLVPFATELHERLAK-DSEKLKE---------------------------------------------
HsApoA5           71   LEKLRPLSGSEAPRLPQDPVGMRRQ---------------------------------------------
HsApoB48          253  RKHVAEAICKEQHLFLP-FSYKNKY--------------------------------------------G
HsApoB52          322  SASLAHMKAKFRETLEDTRDRMYQM---------------------------------------------
HsApoC1           18   LEGPAPAQGTPDVSSALDKLKEFGN---------------------------------------------
HsApoC2           14   LVLGFEVQGTQQPQQDEMPSPTFLT---------------------------------------------
HsApoC3           8    VVALLALLASARASEAE-DASLLSF---------------------------------------------
HsApoC4           22   ACIGACQPEAQEGTLSPPPKLKMSR---------------------------------------------
HsApoD            1    -QAFHLGKCPNPPVQEN-FDVNKYL---------------------------------------------
HsApoE2           75   TQELRALMDETMKELKA-YKSELEE---------------------------------------------
HsApoF            53   LESQTPSSDPLSCQFLH-PKSLPGF---------------------------------------------
HsApoH            43   PGEEITYSCKPGYVSRG-GMRKFIC---------------------------------------------
HsApoJ            121  CMKFYARVCRSGSGLVGRQLEEFLN---------------------------------------------
HsApoL1           115  LDNLARQMIMKDKNWHD-KGQQYRN---------------------------------------------
HsApoL2           56   LNKLASHMVMKDKNRHD-KDQQHRQ---------------------------------------------
HsApoL3           56   LKKLRTYAAIEDEYVQQ-KDEQFRE---------------------------------------------
HsApoL4           80   LKNLTPYVAIEDKDMQQ-KEQQFRE---------------------------------------------
HsApoOL(2)        77   FLFEELMRCDKDSMPDG-NLSEEEK---------------------------------------------
HsApoL6           17   RDEDDAPLCEDVELQDG-DLSPEEK---------------------------------------------
HsApoM            15   IILNSIYQCPEHSQLTT-LGVDGKE---------------------------------------------
HsApoO            16   LLTFKVYAAPKKDSPPK-NSVKVDE---------------------------------------------
HsApoOL           21   VSVHAAKQEESKKQLVK-PEQLPIY---------------------------------------------
HsMTTP            186  IKIKALDSCKIARSGFTTPNQVLGV---------------------------------------------
DmCG31659         19   AMRAFHGACPSNMTAVG-DLDMDRF---------------------------------------------
DmMtp             181  LRVNYNPEEALGVTQQA-QETVFYE---------------------------------------------
DmCvD             195  RSNVPPNTCEFDHQKSVIIGNEAIY---------------------------------------------
DmGlaz            25   TDAYGFGRCPNYPSMPK-FNMSRVL---------------------------------------------
DmFabp            2    SFVGKKYKLDKSENFDE-YMKELGV---------------------------------------------
DmApoLTPII        202  TETDVSGQCQVQYALED-TDSSYVT---------------------------------------------
DmApoLTPI         561  FLDGTQTRASLELKLPR-SKIDYRILVKHKEHIKNGTEHNVIVGLKYTPEKEITGLFSVHLPRRNLFAID
DmNlaz            25   AQVPFPGKCPDVKLLDT-FDAEAYM---------------------------------------------
DmApoLII          160  HEVDVFGMCPTHTSTSKVGNANIIT---------------------------------------------
DmApoLI           337  FDNSQCHFAVEGGINND-KNELVVYGQYEQDKEIKKSKIGFSKNGNEYKPLIEIQDNNGISNSINGYHAD
DmMICOS_A         8    MGIMAAVAVKAAPEPQK-PASSAAD---------------------------------------------
Cel_MICOS         1    MTQDKPIVETISNAGEQ-VTNVFGQ---------------------------------------------
Cel_Vit_2         220  RYSSECPECEKDTELIR-PQTVYTY---------------------------------------------
Cel_Vit_4         217  RFGSECKECEKEGQFVQ-PQTVYTY---------------------------------------------
Cel_Vit_5         217  RFGSECKECEKEGQFVK-PQTVYTY---------------------------------------------
Cel_ApoL_1        4    YESLKSTLMNGEEMPKE-LQVKAWG---------------------------------------------
Cel_MTTP          193  LGYGTNFTKFEGIESVQ-YDQDVWY---------------------------------------------
Cel_Vit_6         227  VQTPVCKDCQQTLEQDKMSSTVLNY---------------------------------------------
Cel_Vit_3         217  RFGSECKECEKEGQFVK-PQTVYTY---------------------------------------------
Cel_Vit-1         220  RYSSECPECEKDTVLIR-PQTVYTY---------------------------------------------
Cel_Apo_L3_Like   22   YDSLESLLFIPRSMSKELSETALRT---------------------------------------------

HsApoA1           32   --------------------------------------KVEPLRAE------------------------
HsApoA2           22   ----------------------------------------------------------------------
HsApoA4           100  ----------------------------------EIGKELEELRAR------------------------
HsApoA5           95   -----------------------------------LQEELEEVKAR------------------------
HsApoB48          278  MVAQVTQTLKLEDTPKINSRFFGEGTKKMGLAFESTKSTSPPKQAE------------------------
HsApoB52          346  -------DIQQELQRYLSLVGQVYSTLVTYISDWWTLAAKNLTDFA------------------------
HsApoC1           42   ----------------------------------------------------------------------
HsApoC2           38   ----------------------------------------------------------------------
HsApoC3           31   ----------------------------------------------------------------------
HsApoC4           46   ----------------------------------WSL---------------------------------
HsApoD            23   --------------------------------GRWYEIEKIPTTFE------------------------
HsApoE2           98   ----------------------------------QLTPVAEETRAR------------------------
HsApoF            76   ----------------------------------SHMAPLPKFLVS------------------------
HsApoH            66   ----------------------------------PLTGLWPINTLK------------------------
HsApoJ            145  -----------------------------QSSPFYFWMNGDRIDSL------------------------
HsApoL1           138  ----------------------------------WFLKEFPRLKSE------------------------
HsApoL2           79   ----------------------------------WFLKEFPRLKRE------------------------
HsApoL3           79   ----------------------------------WFLKEFPQVKRK------------------------
HsApoL4           103  ----------------------------------WFLKEFPQIRWK------------------------
HsApoOL(2)        100  ----------------------------------LFLSYFPLHKFE------------------------
HsApoL6           40   ----------------------------------IFLREFPRLKED------------------------
HsApoM            38   ---------------------------------------FPEVHLG------------------------
HsApoO            39   ----------------------------------LSLYSVPEGQSK------------------------
HsApoOL           44   --------------------------------------TAPPLQSK------------------------
HsMTTP            210  -SSKATSVTTYKIEDSFVIAVLAEETHNFGLNFLQTIKGKIVSKQK------------------------
DmCG31659         42   -------------------------------KGKWYTHSIYPHLSL------------------------
DmMtp             204  -----------LSSEGTLLHAESQENHRLNLAAKPDVGSFVKSSLI------------------------
DmCvD             219  --------GMSPHNETGYYLSMAHAKGTTLIHTFESTGEAQFINSE------------------------
DmGlaz            48   --------------------------------GHWYEVERSFY---------------------------
DmFabp            25   ----------------------------------------------------------------------
DmApoLTPII        225  ------IRKTKDINSCRQRYATHSVLQTTPYTFRDDKTIWPILKSQ------------------------
DmApoLTPI         630  AYMNVTVPEFNSCTASLKVNEKATKDYIIFINGSWFTGHSVAVKANYKDRSSRVQALHHLKMIVESPSFN
DmNlaz            48   --------------------------------GVWYEYAAYPFAFE------------------------
DmApoLII          184  --KARNLNSCSHREQINSGLVSGKVNEKAGITSSLLLQANYIKESR------------------------
DmApoLI           406  GKIVVKKNSNNIERYNFENFQVSNSNNAHVAVNGWSDVGTNSLTSE------------------------
DmMICOS_A         31   ----------------------------------------------------------------------
Cel_MICOS         24   --------------------------------------FWQLVTSK------------------------
Cel_Vit_2         243  -----VLENEE----LKESEVRS--LYTVNVNGQEVMKTETRSKLV------------------------
Cel_Vit_4         240  -----TFKNEK----LQESEVNS--IYTLNVNGQEVVKSETRAKVT------------------------
Cel_Vit_5         240  -----TFKNEK----LQESEVHS--VYTLNVNGQEVVKSETRAKVT------------------------
Cel_ApoL_1        27   --------------------------------AQWHLDRWSSNRQE------------------------
Cel_MTTP          216  ------------------TQNTKVDADIIMVDAIEMLAFKSPLHEK------------------------
Cel_Vit_6         251  -----NITGTSSSFLINSVELRSQHLFAPISEKHQLVSAFTLNTME------------------------
Cel_Vit_3         240  -----TFKNEK----LQESEVHS--IYTLNVNGQEVVKSETRSKVT------------------------
Cel_Vit-1         243  -----ILENEE----LKESEVRS--LYTVNVNGQEVMKTETRSKLV------------------------
Cel_Apo_L3_Like   46   ---------------------------------QFLLDKWSNNRRK------------------------
```

```
HsApoA2          22    ----------------------------------------------------------------------
HsApoA4          112   ----------------------------------------------------------------------
HsApoA5          106   ----------------------------------------------------------------------
HsApoB48         323   ----------------------------------------------------------------------
HsApoB52         385   ----------------------------------------------------------------------
HsApoC1          42    ----------------------------------------------------------------------
HsApoC2          38    ----------------------------------------------------------------------
HsApoC3          31    ----------------------------------------------------------------------
HsApoC4          49    ----------------------------------------------------------------------
HsApoD           37    ----------------------------------------------------------------------
HsApoE2          110   ----------------------------------------------------------------------
HsApoF           88    ----------------------------------------------------------------------
HsApoH           78    ----------------------------------------------------------------------
HsApoJ           162   ----------------------------------------------------------------------
HsApoL1          150   ----------------------------------------------------------------------
HsApoL2          91    ----------------------------------------------------------------------
HsApoL3          91    ----------------------------------------------------------------------
HsApoL4          115   ----------------------------------------------------------------------
HsApoOL(2)       112   ----------------------------------------------------------------------
HsApoL6          52    ----------------------------------------------------------------------
HsApoM           45    ----------------------------------------------------------------------
HsApoO           51    ----------------------------------------------------------------------
HsApoOL          52    ----------------------------------------------------------------------
HsMTTP           255   ----------------------------------------------------------------------
DmCG31659        57    ----------------------------------------------------------------------
DmMtp            239   ----------------------------------------------------------------------
DmCvD            257   ----------------------------------------------------------------------
DmGlaz           59    ----------------------------------------------------------------------
DmFabp           25    ----------------------------------------------------------------------
DmApoLTPII       265   ----------------------------------------------------------------------
DmApoLTPI        700   ITSLNIIYRRKQLLIFYDIQAKYDQDPYGLTIQYASNAHNRNTNAEVRLKVKERDYWINAKLLSEQPKLL
DmNlaz           62    ----------------------------------------------------------------------
DmApoLII         228   ----------------------------------------------------------------------
DmApoLI          451   ----------------------------------------------------------------------
DmMICOS_A        31    ----------------------------------------------------------------------
Cel_MICOS        32    ----------------------------------------------------------------------
Cel_Vit_2        278   ----------------------------------------------------------------------
Cel_Vit_4        275   ----------------------------------------------------------------------
Cel_Vit_5        275   ----------------------------------------------------------------------
Cel_ApoL_1       41    ----------------------------------------------------------------------
Cel_MTTP         244   ----------------------------------------------------------------------
Cel_Vit_6        292   ----------------------------------------------------------------------
Cel_Vit_3        275   ----------------------------------------------------------------------
Cel_Vit-1        278   ----------------------------------------------------------------------
Cel_Apo_L3_Like  59    ----------------------------------------------------------------------

HsApoA1          40    ----------------------------------------------------------------------
HsApoA2          22    ----------------------------------------------------------------------
HsApoA4          112   ----------------------------------------------------------------------
HsApoA5          106   ----------------------------------------------------------------------
HsApoB48         323   ----------------------------------------------------------------------
HsApoB52         385   ----------------------------------------------------------------------
HsApoC1          42    ----------------------------------------------------------------------
HsApoC2          38    ----------------------------------------------------------------------
HsApoC3          31    ----------------------------------------------------------------------
HsApoC4          49    ----------------------------------------------------------------------
HsApoD           37    ----------------------------------------------------------------------
HsApoE2          110   ----------------------------------------------------------------------
HsApoF           88    ----------------------------------------------------------------------
HsApoH           78    ----------------------------------------------------------------------
HsApoJ           162   ----------------------------------------------------------------------
HsApoL1          150   ----------------------------------------------------------------------
HsApoL2          91    ----------------------------------------------------------------------
HsApoL3          91    ----------------------------------------------------------------------
HsApoL4          115   ----------------------------------------------------------------------
HsApoOL(2)       112   ----------------------------------------------------------------------
HsApoL6          52    ----------------------------------------------------------------------
HsApoM           45    ----------------------------------------------------------------------
HsApoO           51    ----------------------------------------------------------------------
HsApoOL          52    ----------------------------------------------------------------------
HsMTTP           255   ----------------------------------------------------------------------
DmCG31659        57    ----------------------------------------------------------------------
DmMtp            239   ----------------------------------------------------------------------
DmCvD            257   ----------------------------------------------------------------------
DmGlaz           59    ----------------------------------------------------------------------
DmFabp           25    ----------------------------------------------------------------------
DmApoLTPII       265   ----------------------------------------------------------------------
DmApoLTPI        770   QLEIHMDKIRDVHIQVGLLNVDKRKELSLELKWDANRDPSQRLGLLAEYNSPGTKHYDGNLMITYPERTI
DmNlaz           62    ----------------------------------------------------------------------
DmApoLII         228   ----------------------------------------------------------------------
DmApoLI          451   ----------------------------------------------------------------------
DmMICOS_A        31    ----------------------------------------------------------------------
Cel_MICOS        32    ----------------------------------------------------------------------
Cel_Vit_2        278   ----------------------------------------------------------------------
Cel_Vit_4        275   ----------------------------------------------------------------------
Cel_Vit_5        275   ----------------------------------------------------------------------
Cel_ApoL_1       41    ----------------------------------------------------------------------
Cel_MTTP         244   ----------------------------------------------------------------------
Cel_Vit_6        292   ----------------------------------------------------------------------
Cel_Vit_3        275   ----------------------------------------------------------------------
Cel_Vit-1        278   ----------------------------------------------------------------------
Cel_Apo_L3_Like  59    ----------------------------------------------------------------------
```

```
HsApoA2         22    ----------------------------------------------------------------------
HsApoA4         112   ----------------------------------------------------------------------
HsApoA5         106   ----------------------------------------------------------------------
HsApoB48        323   ----------------------------------------------------------------------
HsApoB52        385   ----------------------------------------------------------------------
HsApoC1         42    ----------------------------------------------------------------------
HsApoC2         38    ----------------------------------------------------------------------
HsApoC3         31    ----------------------------------------------------------------------
HsApoC4         49    ----------------------------------------------------------------------
HsApoD          37    ----------------------------------------------------------------------
HsApoE2         110   ----------------------------------------------------------------------
HsApoF          88    ----------------------------------------------------------------------
HsApoH          78    ----------------------------------------------------------------------
HsApoJ          162   ----------------------------------------------------------------------
HsApoL1         150   ----------------------------------------------------------------------
HsApoL2         91    ----------------------------------------------------------------------
HsApoL3         91    ----------------------------------------------------------------------
HsApoL4         115   ----------------------------------------------------------------------
HsApoOL(2)      112   ----------------------------------------------------------------------
HsApoL6         52    ----------------------------------------------------------------------
HsApoM          45    ----------------------------------------------------------------------
HsApoO          51    ----------------------------------------------------------------------
HsApoOL         52    ----------------------------------------------------------------------
HsMTTP          255   ----------------------------------------------------------------------
DmCG31659       57    ----------------------------------------------------------------------
DmMtp           239   ----------------------------------------------------------------------
DmCvD           257   ----------------------------------------------------------------------
DmGlaz          59    ----------------------------------------------------------------------
DmFabp          25    ----------------------------------------------------------------------
DmApoLTPII      265   ----------------------------------------------------------------------
DmApoLTPI       840   HFGFNSFTGGPKYFGKVHASWSINEVIEFEYEAGILPGHTLHNWVKAELRTPFDGWRVNSLDAGIYSLKN
DmNlaz          62    ----------------------------------------------------------------------
DmApoLII        228   ----------------------------------------------------------------------
DmApoLI         451   ----------------------------------------------------------------------
DmMICOS_A       31    ----------------------------------------------------------------------
Cel_MICOS       32    ----------------------------------------------------------------------
Cel_Vit_2       278   ----------------------------------------------------------------------
Cel_Vit_4       275   ----------------------------------------------------------------------
Cel_Vit_5       275   ----------------------------------------------------------------------
Cel_ApoL_1      41    ----------------------------------------------------------------------
Cel_MTTP        244   ----------------------------------------------------------------------
Cel_Vit_6       292   ----------------------------------------------------------------------
Cel_Vit_3       275   ----------------------------------------------------------------------
Cel_Vit-1       278   ----------------------------------------------------------------------
Cel_Apo_L3_Like 59    ----------------------------------------------------------------------

HsApoA1         40    ----------------------------------------------------------------------
HsApoA2         22    ----------------------------------------------------------------------
HsApoA4         112   ----------------------------------------------------------------------
HsApoA5         106   ----------------------------------------------------------------------
HsApoB48        323   ----------------------------------------------------------------------
HsApoB52        385   ----------------------------------------------------------------------
HsApoC1         42    ----------------------------------------------------------------------
HsApoC2         38    ----------------------------------------------------------------------
HsApoC3         31    ----------------------------------------------------------------------
HsApoC4         49    ----------------------------------------------------------------------
HsApoD          37    ----------------------------------------------------------------------
HsApoE2         110   ----------------------------------------------------------------------
HsApoF          88    ----------------------------------------------------------------------
HsApoH          78    ----------------------------------------------------------------------
HsApoJ          162   ----------------------------------------------------------------------
HsApoL1         150   ----------------------------------------------------------------------
HsApoL2         91    ----------------------------------------------------------------------
HsApoL3         91    ----------------------------------------------------------------------
HsApoL4         115   ----------------------------------------------------------------------
HsApoOL(2)      112   ----------------------------------------------------------------------
HsApoL6         52    ----------------------------------------------------------------------
HsApoM          45    ----------------------------------------------------------------------
HsApoO          51    ----------------------------------------------------------------------
HsApoOL         52    ----------------------------------------------------------------------
HsMTTP          255   ----------------------------------------------------------------------
DmCG31659       57    ----------------------------------------------------------------------
DmMtp           239   ----------------------------------------------------------------------
DmCvD           257   ----------------------------------------------------------------------
DmGlaz          59    ----------------------------------------------------------------------
DmFabp          25    ----------------------------------------------------------------------
DmApoLTPII      265   ----------------------------------------------------------------------
DmApoLTPI       910   LILVNSTLFWADDQKLQVGYKSDYDVNDQLMSFDVRFGINSTIRDIPTINVKVKHWMDVKKVDTELYLGY
DmNlaz          62    ----------------------------------------------------------------------
DmApoLII        228   ----------------------------------------------------------------------
DmApoLI         451   ----------------------------------------------------------------------
DmMICOS_A       31    ----------------------------------------------------------------------
Cel_MICOS       32    ----------------------------------------------------------------------
Cel_Vit_2       278   ----------------------------------------------------------------------
Cel_Vit_4       275   ----------------------------------------------------------------------
Cel_Vit_5       275   ----------------------------------------------------------------------
Cel_ApoL_1      41    ----------------------------------------------------------------------
Cel_MTTP        244   ----------------------------------------------------------------------
Cel_Vit_6       292   ----------------------------------------------------------------------
Cel_Vit_3       275   ----------------------------------------------------------------------
Cel_Vit-1       278   ----------------------------------------------------------------------
Cel_Apo_L3_Like 59    ----------------------------------------------------------------------
```

```
HsApoA2         22   ----------------------------------------------------------------------
HsApoA4         112  ----------------------------------------------------------------------
HsApoA5         106  ----------------------------------------------------------------------
HsApoB48        323  ---------------------------------------------------------AVLKTLQELKKLT
HsApoB52        385  ----------------------------------------------------------------------
HsApoC1         42   ----------------------------------------------------------------------
HsApoC2         38   ----------------------------------------------------------------------
HsApoC3         31   ----------------------------------------------------------------------
HsApoC4         49   ----------------------------------------------------------------------
HsApoD          37   ----------------------------------------------------------------------
HsApoE2         110  ----------------------------------------------------------------------
HsApoF          88   ----------------------------------------------------------------------
HsApoH          78   ----------------------------------------------------------------------
HsApoJ          162  ----------------------------------------------------------------------
HsApoL1         150  ----------------------------------------------------------------------
HsApoL2         91   ----------------------------------------------------------------------
HsApoL3         91   ----------------------------------------------------------------------
HsApoL4         115  ----------------------------------------------------------------------
HsApoOL(2)      112  ----------------------------------------------------------------------
HsApoL6         52   ----------------------------------------------------------------------
HsApoM          45   ----------------------------------------------------------------------
HsApoO          51   ----------------------------------------------------------------------
HsApoOL         52   ----------------------------------------------------------------------
HsMTTP          255  ----------------------------------------------------------------------
DmCG31659       57   ----------------------------------------------------------------------
DmMtp           239  ----------------------------------------------------------------------
DmCvD           257  ----------------------------------------------------------------------
DmGlaz          59   ----------------------------------------------------------------------
DmFabp          25   ----------------------------------------------------------------------
DmApoLTPII      265  ----------------------------------------------------------------------
DmApoLTPI       980  SGQNDTFNTYSMDSSWEIEKNQRYNNYSGLVHLVSPFKGYEKGGLVAHFSLSDQRVVSGAASLNFDLREF
DmNlaz          62   ----------------------------------------------------------------------
DmApoLII        228  ----------------------------------------------------------------------
DmApoLI         451  ----------------------------------------------------------------------
DmMICOS_A       31   ----------------------------------------------------------------------
Cel_MICOS       32   ----------------------------------------------------------------------
Cel_Vit_2       278  ----------------------------------------------------------------------
Cel_Vit_4       275  ----------------------------------------------------------------------
Cel_Vit_5       275  ----------------------------------------------------------------------
Cel_ApoL_1      41   ----------------------------------------------------------------------
Cel_MTTP        244  ----------------------------------------------------------------------
Cel_Vit_6       292  ----------------------------------------------------------------------
Cel_Vit_3       275  ----------------------------------------------------------------------
Cel_Vit-1       278  ----------------------------------------------------------------------
Cel_Apo_L3_Like 59   ----------------------------------------------------------------------

HsApoA1         40   ---------------------------------LQEGARQKLHE--------------------------
HsApoA2         22   ---------------------------------RQAKEPCVESL--------------------------
HsApoA4         112  --------------------------------LLPHANEVSQKIG-------------------------
HsApoA5         106  ---------------------------------LQPYMAEAHELV-------------------------
HsApoB48        337  ISEQNIQRANLFNKLVTELRGLSDEAVTSLLPQLIEVSSPITLQALVQCGQPQCSTHILQWLKRVHANPL
HsApoB52        385  -----------------------------EQYSIQDWAKRMKALVEQGFTVPEIKTILGTMPAFEVSLQA
HsApoC1         42   --------------------------------TLEDKARELIS---------------------------
HsApoC2         38   ----------------------------------------------------------------------
HsApoC3         31   ----------------------------------------------------------------------
HsApoC4         49   ----------------------------------------------------------------------
HsApoD          37   -----------------------------NGRCIQANYSLMENGK-------------------------
HsApoE2         110  -----------------------------LSKELQAAQARLGADM-------------------------
HsApoF          88   ---------------------------LALRNALEEAGCQADVWA-------------------------
HsApoH          78   -----------------------------CTPRVCPFAGILENGA-------------------------
HsApoJ          162  ---------------------------------LENDRQQTHMLD-------------------------
HsApoL1         150  ---------------------------------LEDNIRRLRALA-------------------------
HsApoL2         91   ---------------------------------LEDHIRKLRALA-------------------------
HsApoL3         91   ---------------------------------IQESIEKLRALA-------------------------
HsApoL4         115  ---------------------------------IQESIERLRVIA-------------------------
HsApoOL(2)      112  ---------------------------------LEQNIKELNTLA-------------------------
HsApoL6         52   ---------------------------------LKGNIDKLRALA-------------------------
HsApoM          45   -----------------------------QWYFIAGAAPTKEELATFD----------------------
HsApoO          51   ---------------------------------YVEEARSQLEES-------------------------
HsApoOL         52   ---------------------------------YVEEQPGHLQMG-------------------------
HsMTTP          255  -----------------------------LELKTTEAGPRLMSGK-------------------------
DmCG31659       57   -----------------------------RVEKCQSTDFIEKEEN-------------------------
DmMtp           239  ---------------------------------LQHVSQGSEEVKQLQLGSLDKAIQSL-----------
DmCvD           257  -----------------------------LLLNFLNETPIDNPIDIETSMAAEPSNLELQRLDPNDPTG-
DmGlaz          59   ----------------------------------------------------------------------
DmFabp          25   ----------------------------------------------------------------------
DmApoLTPII      265  -----------------------------SHCNLTIDNNVYKEIK-------------------------
DmApoLTPI       1050 TLTMNGYVKKFTDNMLTVNITTPLEKFGTINARFGLNEKKRHAVAEVRAPTAALGVEVLADIKNLLNFDV
DmNlaz          62   -----------------------------IGKKCIYANYSLIDNS-------------------------
DmApoLII        228  -----------------------------IVNHLIENVQLTETYK-------------------------
DmApoLI         451  -----------------------------LRISLDHQTFLIKENLKLENGLYEAGFFINDEHSPENIYGS
DmMICOS_A       31   ----------------------------------CSLVCRPSELP-------------------------
Cel_MICOS       32   ---------------------------------NTTNNGDSKPIK-------------------------
Cel_Vit_2       278  ---------------------------------LEENHSIKSHIKKVNGEK-ESIIYSSRWEQLVEDF--
Cel_Vit_4       275  ---------------------------------FVEESKINREIKKVSGPK-EEIVYSMENEKLIEQF--
Cel_Vit_5       275  ---------------------------------FVEESKINREIKKVSGPK-EEIVYSMENEKLIEQF--
Cel_ApoL_1      41   -----------------------------IMNRMEEIAKDLDGLE-------------------------
Cel_MTTP        244  ---------------------------------YGFTLESRTHVEITNRTRVFVTSYCNDTVPSAKCAE-
Cel_Vit_6       292  ---------------------------------LIYAGEKKTEIKQVRNEKTSELVYNQESEWAEQQW--
Cel_Vit_3       275  ---------------------------------FVEESKINREIKKVSGPK-EEIVYSMENEKLIEQF--
Cel_Vit-1       278  ---------------------------------LEENHSIKSHIEKVNGEK-ESIIYSSRWEQLVEDF--
Cel_Apo_L3_Like 59   -----------------------------IVRQMEGIAEKLDNWE-------------------------
```

```
HsApoA2           33   ----------------------------------------------------------------------
HsApoA4           125  ----------------------------------------------------------------------
HsApoA5           118  ----------------------------------------------------------------------
HsApoB48          407  LIDVVTYLVALIPEPSAQQLREIFNMARDQRSRATLYALSHAVNNYHKTNPTGTQELLDIANYLMEQIQD
HsApoB52          427  LQKATFQTPDFIVPLTDLRIPSVQINFKDLKNIKIPSRFSTPEFTILNTFHIPSFTIDFVEMKVKIIRTI
HsApoC1           53   ----------------------------------------------------------------------
HsApoC2           38   ----------------------------------------------------------------------
HsApoC3           31   ----------------------------------------------------------------------
HsApoC4           49   ----------------------------------------------------------------------
HsApoD            53   ----------------------------------------------------------------------
HsApoE2           126  ----------------------------------------------------------------------
HsApoF            106  ----------------------------------------------------------------------
HsApoH            94   ----------------------------------------------------------------------
HsApoJ            174  ----------------------------------------------------------------------
HsApoL1           162  ----------------------------------------------------------------------
HsApoL2           103  ----------------------------------------------------------------------
HsApoL3           103  ----------------------------------------------------------------------
HsApoL4           127  ----------------------------------------------------------------------
HsApoOL(2)        124  ----------------------------------------------------------------------
HsApoL6           64   ----------------------------------------------------------------------
HsApoM            64   ----------------------------------------------------------------------
HsApoO            63   ----------------------------------------------------------------------
HsApoOL           64   ----------------------------------------------------------------------
HsMTTP            271  ----------------------------------------------------------QAAAIIKAVDSK
DmCG31659         73   ----------------------------------------------------------------------
DmMtp             265  -----------------------------------------LEWYRVFELESDVDGMISAIKEQTLEDQL
DmCvD             297  -------------------------------------------GRSPQQQETLIAQAGTLLDSLAEALET
DmGlaz            59   ----------------------------------------------------------------------
DmFabp            25   ----------------------------------------------------------------------
DmApoLTPII        281  ----------------------------------------------------------------------
DmApoLTPI         1120 KLSVATPIESFQQAAIFALFNPERVDMRGLWNNVTLGFTGVWHMQNITDFEYSYHVFTPLAGFEENGFIV
DmNlaz            78   ----------------------------------------------------------------------
DmApoLII          244  ----------------------------------------------------------------------
DmApoLI           493  SIHLTIADQSYALKTNGKAAAWSIGSDGSFNFQKLADSNSARAGSLVENVEIQYKNKQVGGIKIMSNFDV
DmMICOS_A         42   ----------------------------------------------------------------------
Cel_MICOS         44   ----------------------------------------------------------------------
Cel_Vit_2         312  -------------------------------------------FKNGDKAEFAPFEKFPLDKKMHLIKTI
Cel_Vit_4         309  -------------------------------------------YKQGDKAEVNPFKAIEIEQKVEQLEEI
Cel_Vit_5         309  -------------------------------------------YQQGDKAEVNPFKAIEMEQKVEQLQEI
Cel_ApoL_1        57   ----------------------------------------------------------------------
Cel_MTTP          280  ----------------------------------------------------------------------
Cel_Vit_6         327  -------------------------------------------AQTGEEKYLRQLPQWT-ENKVEMIKKM
Cel_Vit_3         309  -------------------------------------------YQQGDQAEVNPFKAIEMEQKVEQLDEI
Cel_Vit-1         312  -------------------------------------------FKNGDKAEFAPFEKFPLDKKMHLIKTI
Cel_Apo_L3_Like   75   ----------------------------------------------------------------------

HsApoA1           51   ----------------------------------------------------------------------
HsApoA2           33   ----------------------------------------------------------------------
HsApoA4           125  ----------------------------------------------------------------------
HsApoA5           118  ----------------------------------------------------------------------
HsApoB48          477  DCTGDEDYTYLILRVIGNMGQTMEQLTPELKSSILKCVQSTKPSLMIQKAAIQALRKMEPKDKDQEVLLQ
HsApoB52          497  DQMLNSELQWPVPDIYLRDLKVEDIPLARITLPDFRLPEIAIPEFIIPTLNLNDFQVPDLHIPEFQLPHI
HsApoC1           53   ----------------------------------------------------------------------
HsApoC2           38   ----------------------------------------------------------------------
HsApoC3           31   ----------------------------------------------------------------------
HsApoC4           49   ----------------------------------------------------------------------
HsApoD            53   ----------------------------------------------------------------------
HsApoE2           126  ----------------------------------------------------------------------
HsApoF            106  ----------------------------------------------------------------------
HsApoH            94   ----------------------------------------------------------------------
HsApoJ            174  ----------------------------------------------------------------------
HsApoL1           162  ----------------------------------------------------------------------
HsApoL2           103  ----------------------------------------------------------------------
HsApoL3           103  ----------------------------------------------------------------------
HsApoL4           127  ----------------------------------------------------------------------
HsApoOL(2)        124  ----------------------------------------------------------------------
HsApoL6           64   ----------------------------------------------------------------------
HsApoM            64   ----------------------------------------------------------------------
HsApoO            63   ----------------------------------------------------------------------
HsApoOL           64   ----------------------------------------------------------------------
HsMTTP            284  YTAIPIVGQVFQSHCKGCPSLSELWRSTRK---------------YLQPDNLSKAEAVRNFLAFIQHLRT
DmCG31659         73   ----------------------------------------------------------------------
DmMtp             295  K----ASLTELQSADVGKSSLALAYVKLIP---------------LARITRQEQFEDLLTEHAEVLPQLV
DmCvD             325  TEFKFSEPYDSTLSDVIKLLSEMDFDSLTK---------------LYREVDIGTSYRQETIRNIFHEIIP
DmGlaz            59   ----------------------------------------------------------------------
DmFabp            25   ----------------------------------------------------------------------
DmApoLTPII        281  ----------------------------------------------------------------------
DmApoLTPI         1190 QLLKRKEFVFQLHGKMSNYKLGVKINGEPKSDLVNQLGSNKMELEMLYDADFKPLNAETDYKPDADEEYF
DmNlaz            78   ----------------------------------------------------------------------
DmApoLII          244  ----------------------------------------------------------------------
DmApoLI           563  NKMDVDVEISREQKIGSIIVKYESNQRHAQ---------------DYSLEASAKINKHSIDVISKCDFNG
DmMICOS_A         42   ----------------------------------------------------------------------
Cel_MICOS         44   ----------------------------------------------------------------------
Cel_Vit_2         340  T----EQIQEVENNMPETSHFLARLVRIFR---------------TTSTSQLKEIHETLYVKADKKI---
Cel_Vit_4         337  F----RQIQEHEQNTPETVHLIARAVRMFR---------------MCTIEELKKVHTTIYTKAEKKV---
Cel_Vit_5         337  F----RQIQEHEQNTPETVHLIARAVRMFR---------------MCTIEELKKVHTTIYTKAEKKV---
Cel_ApoL_1        57   ----------------------------------------------------------------------
Cel_MTTP          280  -----QAFGAVRVGGKLYEHVKIAQEQSNK---------------LTKLIGTYRRHLQDMGDSHICEKHS
Cel_Vit_6         354  FSLMAKQIEQGEAEL-EAAHTVARIVKVLR---------------ECNEEQLEQIYRHVAEHKDEKIAEQ
Cel_Vit_3         337  F----RQIQEHEQNTPETVHLIARAVRMFR---------------MCTIEELKKVHTTIYTKAEKKV---
Cel_Vit-1         340  T----EQIQEVENNIPETSHFLARLVRIFR---------------TTSTSQLKEIHETLYVKADKKI---
Cel_Apo_L3_Like   75   ----------------------------------------------------------------------
```

```
HsApoA2          33   ----------------------------------------------------------------------
HsApoA4          125  -----------------------------------------DNLRELQQRLEPYADQLRTQVSTQAEQLR
HsApoA5          118  -----------------------------------------------GWNLEGLRQQLKPYTMDLMEQVA
HsApoB48         547  TFLDDASPGDKRLAAYLMLMRSPSQADINKIVQILPWEQNEQVKNFVASHIANILNSEELDIQDLKKLVK
HsApoB52         567  SHTIEVPTFGKLYSILKIQSPLFTLDANADIGNGTTSANEAGIAASITAKGESKLEVLNFDFQANAQLSN
HsApoC1          53   ----------------------------------------------------------------------
HsApoC2          38   ----------------------------------------------------------------------
HsApoC3          31   ----------------------------------------------------------------------
HsApoC4          49   ----------------------------------------------------------------------
HsApoD           53   ----------------------------------------------------IKVLNQELRADGTVNQIE
HsApoE2          126  --------------------------------------------------------------EDVCGRLV
HsApoF           106  ----------------------------------------LQLQLYRQGGVNATQVLIQHLRGLQKGRST
HsApoH           94   ---------------------------------------VRYTTFEYPNTISFSCNTGFYLNGADSAKCT
HsApoJ           174  ----------------------------------------------VMQDHFSRASSIIDELFQDRFFTR
HsApoL1          162  -----------------------------------------------------DGVQKVHKGTTIANVVS
HsApoL2          103  -----------------------------------------------------EEVEQVHRGTTIANVVS
HsApoL3          103  -----------------------------------------------------NGIEEVHRGCTISNVVS
HsApoL4          127  -----------------------------------------------------NEIEKVHRGCVIANVVS
HsApoOL(2)       124  -----------------------------------------------------DQVDTTHELLTKTSLVA
HsApoL6          64   -----------------------------------------------------DDIDKTHKKFTKANMVA
HsApoM           64   ----------------------------------------------------------------------
HsApoO           63   -------------------------------------------------------------ISQLRHYCE
HsApoOL          64   -------------------------------------------------------------FASIRTATG
HsMTTP           339  AKKEEILQILKM--------------------------ENKEVLPQLVDAVTSAQTSDSLEAILDFLDFK
DmCG31659        73   ----------------------------------------------------------------KFSVVA
DmMtp            346  DLLGAVQTFDAH--------------------------NATFGFLYKESETTSEQLDLLEKYLQSLAVAT
DmCvD            380  RIGTKASVFLTH--------------------------HLVLNKLTKPQIAVQLLIPMPFHIFELSAELV
DmGlaz           59   ----------------------------------------------------------------------
DmFabp           25   ----------------------------------------------------------------------
DmApoLTPII       281  ----------------------------------------------------CLETHLLVPFSNASSGAL
DmApoLTPI        1260 SYFTNFQVDTLVWPTIVGNVDIQEIIDFYLVVGHVELPQGKVEFKDRLHYPDYINVHNLLTVTTPFAVAK
DmNlaz           78   --------------------------------------------------TVSVVNAAINRFTGQPSNVT
DmApoLII         244  -----------------------------------------FIGNTKRNSDISAKVVTILKLKNPSGTKA
DmApoLI          618  NVYVVDNSLVTSWGTLLSAKGEIGQRYSAQDININIQGNVQISGKDKVTQWILKVIGTPDKTNSDFRISR
DmMICOS_A        42   -----------------------------------------------------------------IYGSL
Cel_MICOS        44   --------------------------------------------------------------IEQLPIYA
Cel_Vit_2        387  -QSLMEHALAIA--------------------------GTKNTIQHILVHME-NEDILPL--GQILKTIQ
Cel_Vit_4        384  -QLVIETTLAVA--------------------------GTKNTIQHLIHHFE-KKSITPLRAAELLKSVQ
Cel_Vit_5        384  -QLVIETTLAVA--------------------------GTKNTIQHLIHHFE-KKSITPLRAAELLKSVQ
Cel_ApoL_1       57   -------------------------------------------------------------KASAIATTV
Cel_MTTP         331  LLYSQIAQEARL--------------------------AKRQDWEAAIQYPENDHVLSLIASALGGVGTA
Cel_Vit_6        408  LRSIYFNTLALA--------------------------GTRVTIQQFVDKVQSRKNIAPLKASVAIKTLV
Cel_Vit_3        384  -QLVIETTLAVA--------------------------GTKNTIQHLIHHFE-KKSITPLRAAELLKSVQ
Cel_Vit-1        387  -QSLMEHALAIA--------------------------GTKNTIQHILVHIE-NEDIVPLEAAQLLKSIQ
Cel_Apo_L3_Like  75   ------------------------------------------------------------KGCAISTAVG

HsApoA1          51   ----------------------------------------------------------------------
HsApoA2          33   ----------------------------------------------------------------------
HsApoA4          154  ----------------------------------------------------------------------
HsApoA5          141  ----------------------------------------------------------------------
HsApoB48         617  EALKESQLPTVMDFRKFSRNYQLYKSVSL-----------------------------------------
HsApoB52         636  ----------------------------------------------------------------------
HsApoC1          53   ----------------------------------------------------------------------
HsApoC2          38   ----------------------------------------------------------------------
HsApoC3          31   ----------------------------------------------------------------------
HsApoC4          49   ----------------------------------------------------------------------
HsApoD           71   ----------------------------------------------------------------------
HsApoE2          134  ----------------------------------------------------------------------
HsApoF           136  ----------------------------------------------------------------------
HsApoH           125  ----------------------------------------------------------------------
HsApoJ           198  ----------------------------------------------------------------------
HsApoL1          179  ----------------------------------------------------------------------
HsApoL2          120  ----------------------------------------------------------------------
HsApoL3          120  ----------------------------------------------------------------------
HsApoL4          144  ----------------------------------------------------------------------
HsApoOL(2)       141  ----------------------------------------------------------------------
HsApoL6          81   ----------------------------------------------------------------------
HsApoM           64   ----------------------------------------------------------------------
HsApoO           72   ----------------------------------------------------------------------
HsApoOL          73   ----------------------------------------------------------------------
HsMTTP           382  ----------------------------------------------------------------------
DmCG31659        79   ----------------------------------------------------------------------
DmMtp            389  ----------------------------------------------------------------------
DmCvD            423  ----------------------------------------------------------------------
DmGlaz           59   ----------------------------------------------------------------------
DmFabp           25   ----------------------------------------------------------------------
DmApoLTPII       299  ----------------------------------------------------------------------
DmApoLTPI        1330 NIKSIVEYHVDLNFNAFYERVKFIVNDDKDNTQELGFVFNYTALQDNVKPKAHDVQVTLLTPYEMLHEIY
DmNlaz           98   ----------------------------------------------------------------------
DmApoLII         273  ----------------------------------------------------------------------
DmApoLI          687  ----------------------------------------------------------------------
DmMICOS_A        47   ----------------------------------------------------------------------
Cel_MICOS        52   ----------------------------------------------------------------------
Cel_Vit_2        427  ----------------------------------------------------------------------
Cel_Vit_4        426  ----------------------------------------------------------------------
Cel_Vit_5        426  ----------------------------------------------------------------------
Cel_ApoL_1       66   ----------------------------------------------------------------------
Cel_MTTP         374  ----------------------------------------------------------------------
Cel_Vit_6        451  ----------------------------------------------------------------------
Cel_Vit_3        426  ----------------------------------------------------------------------
Cel_Vit-1        429  ----------------------------------------------------------------------
Cel_Apo_L3_Like  85   ----------------------------------------------------------------------
```

```
HsApoA2          33   ------------------------VSQYFQTVTDYGKDLMEKVKSP------------------------
HsApoA4          154  ----------------RQLTP---YAQRMERVLRENADSLQASLRP------------------------
HsApoA5          141  ----------------LRVQE---LQEQLRVVGEDTKAQLLGGVDE------------------------
HsApoB48         645  ----------------PSLDPASAKIEGNLIFDPNNYLPKESMLKT------------------------
HsApoB52         636  ----------------PKINPLALKESVKFSSKYLRTEHGSEMLFFGNAIEGKSNTVASLHTEKNTLELS
HsApoC1          53   ------------------------RIKQSELSAKMRLEPFPGHGRA------------------------
HsApoC2          38   -----------------------QVKESLSSYWESAKTAAQNLYEK------------------------
HsApoC3          31   ------------------------MQGYMKHATKTAKDALSSVQES------------------------
HsApoC4          49   ------------------------VRGRMKELLETVVNRTRDGWQW------------------------
HsApoD           71   ----------------GEATP---VNLTEPAKLEVKFSWFMPSAPY------------------------
HsApoE2          134  ----------------QYRGE---VQAMLGQSTEELRVRLASHLRK------------------------
HsApoF           136  ----------------ERNVSVEALASALQLLAREQQSTGRVGRSL------------------------
HsApoH           125  ----------------EEGKWSPELPVCAPIICPPPSIPTFATLRV------------------------
HsApoJ           198  ----------------EPQDTYHYLPFSLPHRRPHFFFPKSRIVRS------------------------
HsApoL1          179  ----------------GSLS----ISSGILTLVGMGLAPFTEGGSL------------------------
HsApoL2          120  ----------------NSVG----TTSGILTLLGLGLAPFTEGISF------------------------
HsApoL3          120  ----------------SSTG----AASGIMSLAGLVLAPFTAGTSL------------------------
HsApoL4          144  ------------------------GSTGILSVIGVMLAPFTAGLSL------------------------
HsApoOL(2)       141  ----------------SSSGA---VSGVMNIL-GLALAPVTAGGSL------------------------
HsApoL6          81   ----------------TSTAV---ISGVMSLL-GLALAPATGGGSL------------------------
HsApoM           64   ------------------------PVDNIVFNMAAGSAPMQLHLRA------------------------
HsApoO           72   ----------------PYTTW---CQETYSQTKPKMQSLVQWGLDS------------------------
HsApoOL          73   ----------------CYIGW---CKGVYVFVKNGIMDTVQFGKDA------------------------
HsMTTP           382  ----------------SDSSII--LQERFLYACGFASHPNEELLRA------------------------
DmCG31659        79   ----------------RELNT---QTGTVKMRKADILNVEPEFGRY------------------------
DmMtp            389  ----------------HPDRK---IVEHLFGLLEQESIKKHLKLRE------------------------
DmCvD            423  ----------------QKCEDFLNIGPDRPDVRQAAILSFATLIHN------------------------
DmGlaz           59   ------------------------LPEIASGCTTFQFEPYNKGEQS------------------------
DmFabp           25   --------------------------GLVTRKMGNSLSPTVEVTLE------------------------
DmApoLTPII       299  ----------------TTSTSRLKLDGVESYSAGEFLEQNPELVER------------------------
DmApoLTPI        1400 VHGHIELDDNAYKGNISAVTAHTHLSMAASIENEDNFLETSVGIQLETDAIPHYGCQVYFKKDFSAVDKA
DmNlaz           98   ----------------GQAKV---LGPGQLAVAFYPTQPLTKANYL------------------------
DmApoLII         273  ----------------NSPGTGSTVRSLIFQRPETYTSKNINALKT------------------------
DmApoLI          687  ----------------DTSELIKLTSESQHPQDKISFAKLNLIVKN------------------------
DmMICOS_A        47   ----------------RKTEP---KPERHPPQDSVLHKNLEAGVRY------------------------
Cel_MICOS        52   ----------------EDNAP---LKQKFLPEEPLPLQREFATIRI------------------------
Cel_Vit_2        427  ----------------ETPFPSQSIAEALIKFAESRVAKNNLVVRQ------------------------
Cel_Vit_4        426  ----------------ETLYPSEHIADLLIQLAQSPLSEKYEPLRQ------------------------
Cel_Vit_5        426  ----------------ETLYPSEHIADLLIQLAQSPLSEKYEPLRQ------------------------
Cel_ApoL_1       66   ----------------GSSVD---IASGLAVFGGLFFMPPVAIAGL------------------------
Cel_MTTP         374  ----------------ESITT---AREVLLTASPDYLDDLLFGISQ------------------------
Cel_Vit_6        451  ----------------DMRYPSLAIAEDIARLCESDVSSSFPALRQ------------------------
Cel_Vit_3        426  ----------------ETLYPSEHIADLLIQLAQSPLSEKYEPLRQ------------------------
Cel_Vit-1        429  ----------------ETPFPSQTIAEALIKFAESRVSKNNQVVRQ------------------------
Cel_Apo_L3_Like  85   ----------------STVG----IASGIAVIGGLILMPPVAIAGL------------------------

HsApoA1          72   ----------------------------------------------------------------------
HsApoA2          55   ----------------------------------------------------------------------
HsApoA4          181  ----------------------------------------------------------------------
HsApoA5          168  ----------------------------------------------------------------------
HsApoB48         675  -----------TLTAFGFASADLIEIGLEGKGFEPTLEALFGKQGFFPDSVNKALYWVNGQVPDGVSKVL
HsApoB52         691  NGVIVKINNQLTLDSNTKYFHKLNIPKLDFSSQADLRNEIKTLLKAGHIAWTSSGKGSWKWACPRFSDEG
HsApoC1          75   ----------------------------------------------------------------------
HsApoC2          61   ----------------------------------------------------------------------
HsApoC3          53   ----------------------------------------------------------------------
HsApoC4          71   ----------------------------------------------------------------------
HsApoD           98   ----------------------------------------------------------------------
HsApoE2          161  ----------------------------------------------------------------------
HsApoF           166  ----------------------------------------------------------------------
HsApoH           155  ----------------------------------------------------------------------
HsApoJ           228  ----------------------------------------------------------------------
HsApoL1          205  ----------------------------------------------------------------------
HsApoL2          146  ----------------------------------------------------------------------
HsApoL3          146  ----------------------------------------------------------------------
HsApoL4          166  ----------------------------------------------------------------------
HsApoOL(2)       167  ----------------------------------------------------------------------
HsApoL6          107  ----------------------------------------------------------------------
HsApoM           86   ----------------------------------------------------------------------
HsApoO           99   ----------------------------------------------------------------------
HsApoOL          100  ----------------------------------------------------------------------
HsMTTP           410  ---------------------------------------------------------------------L
DmCG31659        106  ----------------------------------------------------------------------
DmMtp            416  ---------------------------------------------------------------------S
DmCvD            453  ----------------------------------------------------------------------
DmGlaz           81   ----------------------------------------------------------------------
DmFabp           45   ----------------------------------------------------------------------
DmApoLTPII       329  -------------------------------------------------RATLVFDHTPAVKPSHDEIKA
DmApoLTPI        1470 IDIRFEVTDNGTLNQLHISTDWHTDPSYIVNANGRIKTTMLPLQMASTSVLVIQGQNPHLNFDLNLLSQN
DmNlaz           125  ----------------------------------------------------------------------
DmApoLII         303  ----------------------------------------------------------------------
DmApoLI          717  ----------QLTAKGEFRVAKNGKGDFTASIDTLKTEPKHKLEIESKFHIQSPKYDIDASLTLDGKRKV
DmMICOS_A        74   ----------------------------------------------------------------------
Cel_MICOS        79   ----------------------------------------------------------------------
Cel_Vit_2        457  ----------------------------------------------------AAWLAAGSVVRGIVDYKN
Cel_Vit_4        456  ----------------------------------------------------SAWLAAGSVVRGFASKTQ
Cel_Vit_5        456  ----------------------------------------------------SAWLAAGSVVRGFASKTQ
Cel_ApoL_1       93   ----------------------------------------------------------------------
Cel_MTTP         401  ----------------------------------------------------------------------
Cel_Vit_6        481  ----------------------------------------------------SCWLTYGAIVNGVCGQTP
Cel_Vit_3        456  ----------------------------------------------------SAWLAAGSVVRGFASKTQ
Cel_Vit-1        459  ----------------------------------------------------SAWLAAGSVVRGIVDYKN
Cel_Apo_L3_Like  111  ----------------------------------------------------------------------
```

```
HsApoA2           55   ----------------------------------------------------------------------
HsApoA4           181  --------------------------HADELKAKIDQ-------------------------NVEELKGR
HsApoA5           168  -----------------------AWALLQGLQSRVVH-------------------------HTGRFKEL
HsApoB48          735  VDHFGYTKDDKHEQDMVNGIMLSVEKLIKDLKSKEVP-------------------------EARAYLRI
HsApoB52          761  THESQISFTIEGPLTSFGLSNKINSKHLRVNQNLVYESGSLNFSKLEIQSQVDSQHVGHSVLTAKGMALF
HsApoC1           75   ----------------------------------------------------------------------
HsApoC2           61   ----------------------------------TYL-------------------------P-------
HsApoC3           53   ----------------------------------------------------------------------
HsApoC4           71   -----------------------------------FW-------------------------SPSTFRGF
HsApoD            98   --------------------------WILATDYENYA-------------------------LVYSCTCI
HsApoE2           161  ----------------------LRKRLLRDAD--DLQ-------------------------KRLAVYQA
HsApoF            166  --------------------------PTEDCENEKEQ-------------------------AVHNVVQL
HsApoH            155  ------------------------YKPSAGNNS-LYR-------------------------DTAVFECL
HsApoJ            228  -----LMPFSPYEPLNFHAMFQPFLEMIHEAQQ-AMD-------------------------IHFHSPAF
HsApoL1           205  --------------------------VLLEPGM-ELG-------------------------ITAALTGI
HsApoL2           146  --------------------------VLLDTGM-GLG-------------------------AAAAVAGI
HsApoL3           146  --------------------------ALTAAGV-GLG-------------------------AASAVTGI
HsApoL4           166  --------------------------SITAAGV-GLG-------------------------IASATAGI
HsApoOL(2)        167  --------------------------MLSATGT-GLG-------------------------AAAAITNI
HsApoL6           107  --------------------------LLSTAGQ-GLA-------------------------TAAGVTSI
HsApoM            86   --------------------------TIRMKDG-LCV-------------------------PRKWIYHL
HsApoO            99   ------------------------YDYLQNAPP-GFF-------------------------PRLGVIGF
HsApoOL           100  ------------------------YVYLKNPPR-DFL-------------------------PKMGVITV
HsMTTP            412  ISKFKGSIGSSDIRETVMIITGTLVRKLCQNEGCKLK-------------------------AVVEAKKL
DmCG31659         106  --------------------------VLGTTST-AFP-------------------------EGVLMYVL
DmMtp             418  VIQTVATLTRQSGLDVEDPLLKEVRSYLLQGLT-SKE-------------------------PTLYIRAL
DmCvD             453  -----VYVAKGIDKEKFEEYVQKYFNAYLSDRD-FDQ-------------------------KMLYLQGL
DmGlaz            81   ------------------------------KFS-NFK-------------------------LAVAIKNI
DmFabp            45   ----------------------------------------------------------------GDTYTL
DmApoLTPII        351  ARELLVEMCRVGFPNIQREFIDVFTNFLQTSKSLDYK-------------------------TLSVLLQR
DmApoLTPI         1540 GQSIAYGARANKKKDVFNIEVWTPMKNFRNISMHGTA-----IRSPRDPGRYDVSGFLYRNMATYEVTGA
DmNlaz            125  --------------------------VLGTDYE-SYA-------------------------VVYSCTSV
DmApoLII          303  ---ILSDLVDSTGDYVKKETAKKFVEFIRLLRQSDSE-------------------------TLLELAAF
DmApoLI           778  HLKSENTIEKLKFSTKNIGEANDKIIAFEANGS-LKG-------------------------ELRGNGEI
DmMICOS_A         74   --------------------------VREEVQS-GYK-------------------------AVADQAGI
Cel_MICOS         79   -----------------------------ACEQ-EYD-------------------------RVAERFKV
Cel_Vit_2         476  IRPL----VREDKRELKEKFLRVFMQQYKDAET-TYE-------------------------KILALKTI
Cel_Vit_4         475  DLPL----IRPASRQTKEKYVRVFMQHFRNADS-TYE-------------------------KVLALKTL
Cel_Vit_5         475  DLPL----IRPASRQTKEKYVRVFMQHFRNADS-TYE-------------------------KVLALKTL
Cel_ApoL_1        93   --------------------------IVGAASG-VSN-------------------------VATGVNKK
Cel_MTTP          401  ---------SSSNNEKWHKQLMYWLGSLDKKSE-EYW-------------------------KVANTIAT
Cel_Vit_6         500  RVFVQKNGVKMCPRDAKQRIVDKLVQQFESAST-RYE-------------------------KVLALKTL
Cel_Vit_3         475  DLPL----IRPASRQTKEKYVRVFMQHFRNADS-TYE-------------------------KVLALKTL
Cel_Vit-1         478  IRPL----VREDKRELKEKFLRVFMQQYKDAET-TYE-------------------------KILALKSI
Cel_Apo_L3_Like   111  --------------------------IVGTASG-VSN-------------------------LATGITKF

HsApoA1           72   ----------------------------------------------------------------------
HsApoA2           55   ----------------------------------------------------------------------
HsApoA4           201  LTPYADEFKVKIDQTVEELRR-------------------------------------------------
HsApoA5           191  FHPYAESLVSGI----------------------------------------------------------
HsApoB48          780  LGEELGFASLHDLQLLGKLLLMGARTLQGIPQMIGEVIRKGSKNDFFLHYIFMENAFELPTGAGLQLQIS
HsApoB52          831  GEGKAEFTGRHDAHLNGKVIGTLKNSLFFSAQPFEITASTNNEGNLKVRFPLRLTGKIDFLNNYALFLSP
HsApoC1           75   ----------------------------------------------------------------------
HsApoC2           65   ----------------------------------------------------------------------
HsApoC3           53   ----------------------------------------------------------------------
HsApoC4           82   MQTYYDDHLR------------------------------------------------------------
HsApoD            118  IQLFHVDFAWILAR--------------------------------------------------------
HsApoE2           183  GAREGAERGLS-----------------------------------------------------------
HsApoF            186  LPGVGTFYNLGT----------------------------------------------------------
HsApoH            176  PQHAMFGNDTITCTTHGNWTK-------------------------------------------------
HsApoJ            268  QHPPTEFIREGDDDRTVCREIRHNS---------------------------------------------
HsApoL1           224  TSSTMDYGKKWWTQ--------------------------------------------------------
HsApoL2           165  TCSVVELVN-------------------------------------------------------------
HsApoL3           165  TTSIVEHSYTSSAE--------------------------------------------------------
HsApoL4           185  ASSIVENTYTRSAE--------------------------------------------------------
HsApoOL(2)        186  VTNVLENRS-------------------------------------------------------------
HsApoL6           126  VSGTLERSKNK-----------------------------------------------------------
HsApoM            105  TEGSTDLRT-------------------------------------------------------------
HsApoO            120  AGLIGLLLAR------------------------------------------------------------
HsApoOL           121  SGLAGLVSARK-----------------------------------------------------------
HsMTTP            457  ILGGLEKAEKKEDTRMYLLALKNALLPEGIPS--------------------------------------
DmCG31659         125  DTDYVNFAIR------------------------------------------------------------
DmMtp             462  QNLQDPATIEALLEHAQTGEAPNL----------------------------------------------
DmCvD             493  NNLQLGNVANYLEPIVQDPNEHEDLKFQAAWT--------------------------------------
DmGlaz            96   NRITGNPNVN------------------------------------------------------------
DmFabp            52   TTTSTFKTS-------------------------------------------------------------
DmApoLTPII        396  SASTCEQGRNHLLESLPFIGS-------------------------------------------------
DmApoLTPI         1605 VRMTNSLPIDVVLRVQPKAGGRDGVIELNIHEAGPKKIRFSFSAIEDGKMCQMSGGYSVSKTNGAMDFSV
DmNlaz            144  TPLANFKIVWILTRQREP----------------------------------------------------
DmApoLII          346  PHPNKVLARKVYLDGLFR----------------------------------------------------
DmApoLI           822  QGTFIFNAPDGRVIDGSINRKISTNAKSGLSQGNIDAQLSDTPFGSNKKRSISLIGKLDRLNTKTKEFSA
DmMICOS_A         93   VGHYVETAK-------------------------------------------------------------
Cel_MICOS         95   VDCAMTQTK-------------------------------------------------------------
Cel_Vit_2         516  GNAGLDISVNQLNEIIVDKRQPLPVRKEAIDA--------------------------------------
Cel_Vit_4         515  GNAGIDLSVYELVQLIQDPRQPLSIRTEAVDA--------------------------------------
Cel_Vit_5         515  GNAGIDLSVYELVQIIQDPRQPLSIRTEAVDA--------------------------------------
Cel_ApoL_1        112  LATDHKIRE-------------------------------------------------------------
Cel_MTTP          437  VLNKRCEASTSSLNSCNKGKETIVNKFITDLT--------------------------------AGGVEV
Cel_Vit_6         544  ANAGLDLSVYPLEKIILNEQHETTIRTQAIES--------------------------------------
Cel_Vit_3         515  GNAGIDLSVYELVQLIQDPRQPLSIRTEAVDA--------------------------------------
Cel_Vit-1         518  GNAGLDISVNQLNEIIVDKRQLLPVRKEAIDA--------------------------------------
Cel_Apo_L3_Like   130  FHTKGQHKEVAAMI--------------------------------------------------------
```

```
HsApoA2         55   ----------------------------------------------------------------------
HsApoA4         221  ----------------------------------------------------------------------
HsApoA5         202  ----------------------------------------------------------------------
HsApoB48        850  SSGVIAPGAKAGVKLEVANMQAELVAKPSVSVEFVTNMGIIIPDFARSGVQMNTNFFHESGLEAHVALKA
HsApoB52        901  SAQQASWQVSARFNQYKYNQNFSAGNNENIMEAHVGINGEANLDFLNIPLTIPEMRLPYTIITTPPLKDF
HsApoC1         75   ----------------------------------------------------------------------
HsApoC2         65   ----------------------------------------------------------------------
HsApoC3         53   ----------------------------------------------------------------------
HsApoC4         91   ----------------------------------------------------------------------
HsApoD          131  ----------------------------------------------------------------------
HsApoE2         193  ----------------------------------------------------------------------
HsApoF          197  ----------------------------------------------------------------------
HsApoH          196  ----------------------------------------------------------------------
HsApoJ          292  ----------------------------------------------------------------------
HsApoL1         237  ----------------------------------------------------------------------
HsApoL2         173  ----------------------------------------------------------------------
HsApoL3         178  ----------------------------------------------------------------------
HsApoL4         198  ----------------------------------------------------------------------
HsApoOL(2)      194  ----------------------------------------------------------------------
HsApoL6         136  ----------------------------------------------------------------------
HsApoM          113  ----------------------------------------------------------------------
HsApoO          129  ----------------------------------------------------------------------
HsApoOL         131  ----------------------------------------------------------------------
HsMTTP          488  ----------------------------------------------------------------------
DmCG31659       134  ----------------------------------------------------------------------
DmMtp           485  ----------------------------------------------------------------------
DmCvD           524  -TLALADRRAERIYEVYWPIFESRNASLELRVAAVTLLLISNPTAA------------------------
DmGlaz          105  ----------------------------------------------------------------------
DmFabp          60   ----------------------------------------------------------------------
DmApoLTPII      416  ----------------------------------------------------------------------
DmApoLTPI       1675 LVESTEPEIARINFYGNLSPNSEGSLVGDLSLETPWKALGIDTVHLHSDVGFLNKGGHIVGEYKIGQYIG
DmNlaz          161  ----------------------------------------------------------------------
DmApoLII        363  ----------------------------------------------------------------------
DmApoLI         892  NSNLVYTAFNGEKSEISYQIKQQPNGDAKNIDFSLKAYGNPLPQPFEIAFALGDYSAQHAVVSITSKYGE
DmMICOS_A       101  ----------------------------------------------------------------------
Cel_MICOS       103  ----------------------------------------------------------------------
Cel_Vit_2       547  -LRLLKDTMPRKIQKVLLPIYKNRQYEPEIRMLALWRMMHTRPEES------------------------
Cel_Vit_4       546  -LRLLKDVMPRKIQKVLLPVYKNRQNKPELRMAALWRMMHTIPEEP------------------------
Cel_Vit_5       546  -LRLLKDVMPRKIQKVLLPVYKNRQNKPELRMAALWRMMHTIPEEP------------------------
Cel_ApoL_1      120  ----------------------------------------------------------------------
Cel_MTTP        475  RVLEVLENIPIFGSYTFAKKFICETESEDVQKAALNVILAASKNLYETQ---------------------
Cel_Vit_6       575  -FRRLRTQMPTKIQRVLMPVYLNRQQPQHIRMSALHQIIYTQPEWS------------------------
Cel_Vit_3       546  -LRLLKDVMPRKIQKVLLPVYKNRQNKPELRMAALWRMMHTIPEEP------------------------
Cel_Vit-1       549  -LRLLKDTMPRKIQKVLLPIYKNRQYEPEIRMLALWRMMHTRPEES------------------------
Cel_Apo_L3_Like 143  ----------------------------------------------------------------------

HsApoA1         72   ------------------------------------ALRTHLAPYSDELRQRLAARLEALKENGGARLAE
HsApoA2         55   ------------------------------------ELQAEAKSYFEKSKEQLTPLIKKAGTELVNFLSY
HsApoA4         221  -----SLAPYAQDTQEKLNHQLE-------------GLTFQMKKNAEELKARISASAEELRQRLAPLAED
HsApoA5         202  ------------------GRHVQ-------------ELHRSVAPHAPASPARLSRCVQVLSRKLTLKAKA
HsApoB48        920  GKLKFIIPSPKRPVKLLSGGNTL-------------HLVSTTKTEVIPPLIENRQSWSVCKQVFPGLNYC
HsApoB52        971  SLWEKTGLKEFLKTTKQSFDLSV-------------KAQYKKNKHRHSITNPLAVLCEFISQSIKSFDRH
HsApoC1         75   ------------------------------------GVCFWVEPWQMVQDEQIEKKTSPGEADNIPLVTQ
HsApoC2         65   ------------------------------------AVDEKLRDLYSKSTAAMSTYTGIFTDQVLSVLKG
HsApoC3         53   ------------------------------------QVAQQARGWVTDGFSSLKDYWSTVK---------
HsApoC4         91   ------------------------------------DLGPLTKAWFLESKDSLLKKTHSLCPRLVCGDKD
HsApoD          131  ------------------NPNLP-------------PETVDSLKNILTSNNIDVKKMTVTDQVNCPKLSQ
HsApoE2         193  ------------------------------------AIRERLGPLVEQGRVRAATVGSLAGQPLQERAQA
HsApoF          197  ------------------------------------ALYYATQNCLGKARERGRDGAIDLGYDLLMTMAG
HsApoH          196  ------------------LPECR-------------EVKCPFPSRPDNGFVNYPAKPTLYYKDKATFGCH
HsApoJ          292  ------------------------------------TGCLRMKDQCDKCREILSVDCSTNNPSQAKLRRE
HsApoL1         237  ------------------------------------AQAHDLVIKSLDKLKEVREFLGENISNFLSLAGN
HsApoL2         173  ------------------KLRAR-------------AQARNLDQSGTNVAKVMKEFVGGNTPNVLTLVDN
HsApoL3         178  ------------------------------------AEASRLTATSIDRLKVFKEVMRDITPNLLSLLNN
HsApoL4         198  ------------------------------------LTASRLTATSTDQLEALRDILHDITPNVLSFALD
HsApoOL(2)      194  ------------------NSAAR-------------DKASRLGPLTTSHEAFGGINWSEIEAAGFCVNKC
HsApoL6         136  --------------------EAQ-------------ARAEDILPTYDQEDREDEEEKADYVTAAGKIIYN
HsApoM          113  ------------------------------------EGRPDMKTELFSSSCPGGIMLNETGQGYQRFLLY
HsApoO          129  ------------------GSKIK-------------KLVYPPGFMGLAASLYYPQQAIVFAQVSGERLYD
HsApoOL         131  ------------------GSKFK-------------KITYPLGLATLGATVCYPVQSVIIAKVTAKKVYA
HsMTTP          488  ------LLKYAEAGEGPISHLATTALQRYDLPFITDEVKKTLNRIYHQNRKVHEKTVRTAAAIILNNNP
DmCG31659       134  ------------------------------------FMCFDASKIFSFHWAVIQTRKRLPSTQVIHMAQY
DmMtp           485  -----SVAALQALKAFPLGSFNS-------------SHRLQFESIFYQRKRRFDSSARTLALDIILSLRP
DmCvD           569  -----RLISIHRIIQSETDPHMI-------------NYYRTTVTSISETTYPCYQHLRRLLSYMHRHLPQ
DmGlaz          105  ------------------IGYAT-------------PENSRSSIMDFKFTTRFPDVIARLLPGSGKY---
DmFabp          60   ------------------------------------AISFKLGVEFDEETLDGRNVKSIITLDGNKLTQE
DmApoLTPII      416  ------------------------------------TASYKVMRDQIINEKLTKQMAHDWMTALSFITRP
DmApoLTPI       1745 RGSCLWSWILAEDMQLVLENYLERP-----------NAKPRIVHASAKYQNPGQTFTQLQAGGRLSVDSK
DmNlaz          161  ------------------SAEAV-------------DAARKILEDNDVSQAFLIDTVQKNCPRLDGNGTG
DmApoLII        363  ------------------------------------TSTAESARVILKQLSKFDEKEKLLAILSLNIVKS
DmApoLI         962  IFSVSANGNYNNNQALEYGLQANIEIPKSTLKSLEINSHGKVLKSLIGNENAAYNVEFFLDSKTSLGQYA
DmMICOS_A       101  ------------------------------------AHTQSTIDMLNEPQNSLHRSGAIVVGGLAGFIFA
Cel_MICOS       103  ------------------------------------KAATKCNAYLTEEWTALPKAAAITVGGMAGFVLG
Cel_Vit_2       592  -----LLVQVVSQMEKETNQQVA-------------ALTHQMIRHFAMSTNPCYQRVAIVCSKVLSFTRY
Cel_Vit_4       591  -----VLAHIVSQMENESNQHVA-------------AFTYNVLRQFSKSTNPCYQQLAVRCSKVLLFTRY
Cel_Vit_5       591  -----VLAHIVSQMENESNQHVA-------------AFTYNVLRQFYKSTNPCYQQLAVRCSKILLFTRY
Cel_ApoL_1      120  ------------------INRML-------------AEDAKFFEELLHSRNDLLNEVRRFVEDKQSSKIF
Cel_MTTP        523  -----LTHKLIKLFRNTCSQETP-------------TSHSQLAIDILLKCVPDHQNVATLILRTETLNPD
Cel_Vit_6       620  -----VLSQIGNQLRQERNQQVR-------------AFTLSLLRSYANNESPCEQTFSSRVQSLLNNIPF
Cel_Vit_3       591  -----VLAHIVSQMENESNQHVA-------------AFTYNVLRQFSKSTNPCYQQLAVRCSKVLLFTRY
Cel_Vit-1       594  -----LLVQVVSQMEKETNQQVA-------------ALTHQMIRHFAKSTNPCYQRVAIVCSKVLSFTRY
Cel_Apo_L3_Like 143  ------------------------------------AEDGVLFEELLKSREELMEAVRKIVEDEEFFKHF
```

```
HsApoA2         90   FVE-------------------------------------------------------------------
HsApoA4         274  VRGNLRGNTEGLQKSL------------------------------------------------------
HsApoA5         242  LHARIQQNL-------------------------------------------------------------
HsApoB48        977  TSGAYSNASSTDSASYYPLTGDTRLELELRPTGEIEQYSVSATYELQREDRALVDTLKFVTQAEGAKQTE
HsApoB52        1028 FEKNRNNALDFVTKSYNETKIKFDKYKAEKSHDELPRTFQIPGYTVPVVNVEV-----------------
HsApoC1         109  ----------------------------------------------------------------------
HsApoC2         100  EED-------------------------------------------------------------------
HsApoC3         78   ----------------------------------------------------------------------
HsApoC4         126  QGQ-------------------------------------------------------------------
HsApoD          171  AKE-------------------------------------------------------------------
HsApoE2         228  WGERLRARM-------------------------------------------------------------
HsApoF          232  MSG-------------------------------------------------------------------
HsApoH          236  DGYSLD----------------------------------------------------------------
HsApoJ          327  LDESLQVA--------------------------------------------------------------
HsApoL1         272  TYQLT-----------------------------------------------------------------
HsApoL2         213  WYQVT-----------------------------------------------------------------
HsApoL3         213  YYEAT-----------------------------------------------------------------
HsApoL4         233  FDEAT-----------------------------------------------------------------
HsApoOL(2)      234  VKA-------------------------------------------------------------------
HsApoL6         174  LRNTL-----------------------------------------------------------------
HsApoM          148  NRSP------------------------------------------------------------------
HsApoO          169  WGLRGY----------------------------------------------------------------
HsApoOL         171  TSQQIF----------------------------------------------------------------
HsMTTP          553  SYMDVKNILLSI----------------------------------------------------------
DmCG31659       169  FGKSA-----------------------------------------------------------------
DmMtp           538  TQEQLGNFLDYLASNDRQ----------------------------------------------------
DmCvD           622  KPESRYWVTGNYIFDYRDSK--------------------------------------------------
DmGlaz          141  ----------------------------------------------------------------------
DmFabp          95   QKG-------------------------------------------------------------------
DmApoLTPII      451  DEETLETFHSILEYAKNRLDAEYT----------------------------------------------
DmApoLTPI       1804 WNLDVNGSAEYKSVDDFKFRVITALPLPVGDRHQLSASYQGNVISQQFNNPDFVLEASYESFEAQNKLLS
DmNlaz          201  LA--------------------------------------------------------------------
DmApoLII        398  VDKETLNQAASQLLPNAPKELY------------------------------------------------
DmApoLI         1032 RVNTVWNGTANDGSYDFEAQTNNMESPLKFNGKYHRKQTGNIKDGDLTGKQTYVLNAQYGAQYVKMDASL
DmMICOS_A       136  ARG-------------------------------------------------------------------
Cel_MICOS       138  LKR-------------------------------------------------------------------
Cel_Vit_2       645  QPQE-QMIASSYAQLPLFLQNSFSGAQFDFAAIFEKNSFLPKDLHASLD---------------------
Cel_Vit_4       644  QPQE-QML-STYSQLPLFNSEWLSGVQFDFATIFEKNAFLPKEVQASFE---------------------
Cel_Vit_5       644  QPQE-QML-STYSQLPLFNSEWLSGVQFDFATIFEKNAFLPKEVQASFE---------------------
Cel_ApoL_1      160  KNF-------------------------------------------------------------------
Cel_MTTP        576  DQEKWHYLYKAIEA--------------------------------------------------------
Cel_Vit_6       673  SSQEIDRFESVYGKWSTYSRRHQSGFEANFASLFTTESVLPTEMMASIE---------------------
Cel_Vit_3       644  QPQE-QML-STYSQLPLFNSEWLSGVQFDFATIFEKNAFLPKEVQASFE---------------------
Cel_Vit-1       647  QPQE-QMIASSYAQLPLFLQNSFSGAQFDFAAIFEKNSFLLKDLHASLD---------------------
Cel_Apo_L3_Like 178  KND-------------------------------------------------------------------

HsApoA1         108  ----------------------------------------------------------------------
HsApoA2         92   ----------------------------------------------------------------------
HsApoA4         289  ----------------------------------------------------------------------
HsApoA5         250  ----------------------------------------------------------------------
HsApoB48        1047 ATMTFKYNRQSMTLSSEVQIPDFDVDLGTILRVNDESTEGKTSYRLTLDIQNKKITEVALMGHLSCDTKE
HsApoB52        1080 ----------------------------------------------------------------------
HsApoC1         109  ----------------------------------------------------------------------
HsApoC2         102  ----------------------------------------------------------------------
HsApoC3         78   ----------------------------------------------------------------------
HsApoC4         128  ----------------------------------------------------------------------
HsApoD          173  ----------------------------------------------------------------------
HsApoE2         236  ----------------------------------------------------------------------
HsApoF          234  ----------------------------------------------------------------------
HsApoH          241  ----------------------------------------------------------------------
HsApoJ          334  ----------------------------------------------------------------------
HsApoL1         276  ----------------------------------------------------------------------
HsApoL2         217  ----------------------------------------------------------------------
HsApoL3         217  ----------------------------------------------------------------------
HsApoL4         237  ----------------------------------------------------------------------
HsApoOL(2)      236  ----------------------------------------------------------------------
HsApoL6         178  ----------------------------------------------------------------------
HsApoM          151  ----------------------------------------------------------------------
HsApoO          174  ----------------------------------------------------------------------
HsApoOL         176  ----------------------------------------------------------------------
HsMTTP          564  ----------------------------------------------------------------------
DmCG31659       173  ----------------------------------------------------------------------
DmMtp           555  ----------------------------------------------------------------------
DmCvD           641  ----------------------------------------------------------------------
DmGlaz          141  ----------------------------------------------------------------------
DmFabp          97   ----------------------------------------------------------------------
DmApoLTPII      474  ----------------------------------------------------------------------
DmApoLTPI       1874 RISYKNATNNLK----------------------------------------------------------
DmNlaz          202  ----------------------------------------------------------------------
DmApoLII        419  ----------------------------------------------------------------------
DmApoLI         1102 GYGAEKVDIAYVIDSSFDSVKDIKVNIRTFKPLDDSTYVVTALFKQTDKSYGLDTTFYHSAHKKGVDIRL
DmMICOS_A       138  ----------------------------------------------------------------------
Cel_MICOS       140  ----------------------------------------------------------------------
Cel_Vit_2       692  ----------------------------------------------------------------------
Cel_Vit_4       690  ----------------------------------------------------------------------
Cel_Vit_5       690  ----------------------------------------------------------------------
Cel_ApoL_1      162  ----------------------------------------------------------------------
Cel_MTTP        589  ----------------------------------------------------------------------
Cel_Vit_6       721  ----------------------------------------------------------------------
Cel_Vit_3       690  ----------------------------------------------------------------------
Cel_Vit-1       694  ----------------------------------------------------------------------
Cel_Apo_L3_Like 180  ----------------------------------------------------------------------
```

```
HsApoA2         92   ----------------------------------------------------------------------
HsApoA4         289  ------------------AELGGHLDQQVE----------------------------------------
HsApoA5         250  ------------------DQLREELSRAFA----------------------------------------
HsApoB48        1117 ERKIKGVISIPRLQAEARSEILAHWSPAKLLL--------------------------------------
HsApoB52        1080 ------------------SPFTIEMSAF------------------------------------------
HsApoC1         109  ----------------------------------------------------------------------
HsApoC2         102  ----------------------------------------------------------------------
HsApoC3         78   ------------------DKFSEFWDLD------------------------------------------
HsApoC4         128  ----------------------------------------------------------------------
HsApoD          173  ------------------PCVESLVSQY------------------------------------------
HsApoE2         236  ------------------EEMGSRTRDRLD----------------------------------------
HsApoF          234  ------------------GPMGLAISAA------------------------------------------
HsApoH          241  ------------------GPEEIECTKL------------------------------------------
HsApoJ          334  ------------------ERLTRKYNELLK----------------------------------------
HsApoL1         276  ------------------RGIGKDIRAL------------------------------------------
HsApoL2         217  ------------------QGIGRNIRAI------------------------------------------
HsApoL3         217  ------------------QTIGSEIRAI------------------------------------------
HsApoL4         237  ------------------KMIANDVHTL------------------------------------------
HsApoOL(2)      236  ------------------IQGIKDLHAY------------------------------------------
HsApoL6         178  ------------------KYAKKNVRAFWK----------------------------------------
HsApoM          151  ------------------HPPEKCVEEF------------------------------------------
HsApoO          174  ------------------IVIEDLWKEN------------------------------------------
HsApoOL         176  ------------------GAVKSLWTKSSK----------------------------------------
HsMTTP          564  ------------------GELPQEMNKYML----------------------------------------
DmCG31659       173  ------------------GLVIGDMSKV------------------------------------------
DmMtp           555  ------------------FEIKTYVLQKLR----------------------------------------
DmCvD           641  ------------------FGIGAMLQVF------------------------------------------
DmGlaz          141  ------------------QVLYTDYENF------------------------------------------
DmFabp          97   ------------------DKPTTIVREF------------------------------------------
DmApoLTPII      474  ------------------LGATAVVHSFCK----------------------------------------
DmApoLTPI       1885 ------------------GLGHVEWGKIQNLSVVEGDFELLHKQGAQREFSAKIITPKFKNEHTFALTGS
DmNlaz          202  ------------------GEDGLDVDDF------------------------------------------
DmApoLII        419  ------------------IAVGNLVAKY------------------------------------------
DmApoLI         1172 DLLKEKPIIISSIAELLGDRKGKVLFEILNLADLDIKINSEASYVSIDEFYIIVNWSSKKLKLDGYELEA
DmMICOS_A       138  ------------------GFIKKVLYSGIG----------------------------------------
Cel_MICOS       140  ------------------GPVGRLLTTTIG----------------------------------------
Cel_Vit_2       692  ------------------AVFGGNWNKYFA----------------------------------------
Cel_Vit_4       690  ------------------TVFGGNWNKYFA----------------------------------------
Cel_Vit_5       690  ------------------TVFGGNWNKYFA----------------------------------------
Cel_ApoL_1      162  ------------------DDVQSHLKTLFG----------------------------------------
Cel_MTTP        589  ------------------SGNKDELKAEFW----------------------------------------
Cel_Vit_6       721  ------------------GVLSGEWNQYFA----------------------------------------
Cel_Vit_3       690  ------------------TVFGGNWNKYFA----------------------------------------
Cel_Vit-1       694  ------------------AVFGGNWNKYFA----------------------------------------
Cel_Apo_L3_Like 180  ------------------GDIENKLKTVFG----------------------------------------

HsApoA1         118  ----------------------------------------------------------------------
HsApoA2         92   ----------------------------------------------------------------------
HsApoA4         301  ----------------------------------------------------------------------
HsApoA5         262  ----------------------------------------------------------------------
HsApoB48        1148 ----------------------------------------------------------------------
HsApoB52        1090 --------------------------------------------GYVFPKAVSMPSFSILGSDVRVPSYT
HsApoC1         109  ----------------------------------------------------------------------
HsApoC2         102  ----------------------------------------------------------------------
HsApoC3         88   ----------------------------------------------------------------------
HsApoC4         128  ----------------------------------------------------------------------
HsApoD          183  ----------------------------------------------------------------------
HsApoE2         248  ----------------------------------------------------------------------
HsApoF          244  ----------------------------------------------------------------------
HsApoH          251  ----------------------------------------------------------------------
HsApoJ          346  ----------------------------------------------------------------------
HsApoL1         286  ----------------------------------------------------------------------
HsApoL2         227  ----------------------------------------------------------------------
HsApoL3         227  ----------------------------------------------------------------------
HsApoL4         247  ----------------------------------------------------------------------
HsApoOL(2)      246  ----------------------------------------------------------------------
HsApoL6         190  ----------------------------------------------------------------------
HsApoM          161  ----------------------------------------------------------------------
HsApoO          184  ----------------------------------------------------------------------
HsApoOL         188  ----------------------------------------------------------------------
HsMTTP          576  ----------------------------------------------------------------------
DmCG31659       183  ----------------------------------------------------------------------
DmMtp           567  ----------------------------------------------------------------------
DmCvD           651  ----------------------------------------------------------------------
DmGlaz          151  ----------------------------------------------------------------------
DmFabp          107  ----------------------------------------------------------------------
DmApoLTPII      486  ----------------------------------------------------------------------
DmApoLTPI       1938 YDLEKSGHHNVVGSLDYPASRRITDLDVSVSSLSNMHGIFNSTLPTFLNVSWLKTDFNFTTNNGKSYRYC
DmNlaz          212  ----------------------------------------------------------------------
DmApoLII        429  ----------------------------------------------------------------------
DmApoLI         1242 RAQSKNIKIQLKNENGIIFSGTATYALKKELNKTIIDGQGKVQYQGKALSGNFKLTRQHFDFGTDREVGF
DmMICOS_A       150  ----------------------------------------------------------------------
Cel_MICOS       152  ----------------------------------------------------------------------
Cel_Vit_2       704  ----------------------------------------------------------------------
Cel_Vit_4       702  ----------------------------------------------------------------------
Cel_Vit_5       702  ----------------------------------------------------------------------
Cel_ApoL_1      174  ----------------------------------------------------------------------
Cel_MTTP        601  ----------------------------------------------------------------------
Cel_Vit_6       733  ----------------------------------------------------------------------
Cel_Vit_3       702  ----------------------------------------------------------------------
Cel_Vit-1       706  ----------------------------------------------------------------------
Cel_Apo_L3_Like 192  ----------------------------------------------------------------------
```

```
HsApoA2           92   ----------------------------------------------------------------------
HsApoA4           301  ---------------EFRRRVEPYGENF------------------------------------------
HsApoA5           262  ---------------GTGTEEGAGPDPQ------------------------------------------
HsApoB48          1148 ---------------QMDSSATAYGSTV----------------------------------------SK
HsApoB52          1117 LILPSLELPVLHVPRNLKLSLPDFKELCTISHIFIPAMGNITYDFSFKSSVITLNTNAELFNQSDIVAHL
HsApoC1           109  ----------------------------------------------------------------------
HsApoC2           102  ----------------------------------------------------------------------
HsApoC3           88   ----------------------------------------------------------------------
HsApoC4           128  ----------------------------------------------------------------------
HsApoD            183  -----------------FQTVTDYGKDL------------------------------------------
HsApoE2           248  ---------------EVKEQVAEVRAKL------------------------------------------
HsApoF            244  ---------------LKPALRSGVQQLI------------------------------------------
HsApoH            251  ---------------GNWSAMPSCKASC------------------------------------------
HsApoJ            346  ---------------SYQWKMLNTSSLL------------------------------------------
HsApoL1           286  -----------------RRARANLQSVP------------------------------------------
HsApoL2           227  ---------------RRARANPQLGAYA------------------------------------------
HsApoL3           227  ---------------RQARARARLPVTT------------------------------------------
HsApoL4           247  -----------------RRSKATVGRPL------------------------------------------
HsApoOL(2)        246  ---------------QMAKSNSGFMAMV------------------------------------------
HsApoL6           190  -----------------LRANPRLANAT------------------------------------------
HsApoM            161  ----------------------------------------------------------------------
HsApoO            184  -----------------------FQKPG------------------------------------------
HsApoOL           188  ---------------EESLPKPKEKTKL------------------------------------------
HsMTTP            576  ---------------AIVQDILRFEMPA------------------------------------------
DmCG31659         183  ----------------------------------------------------------------------
DmMtp             567  ---------------MLAEKCPRFRALF------------------------------------------
DmCvD             651  ---------------LVGDPKSDMPVVA------------------------------------------
DmGlaz            151  ---------------AILWSCGSIGSLG------------------------------------------
DmFabp            107  -------------------TDNELITLI------------------------------------------
DmApoLTPII        486  ---------------HHEACEENLRVQQ------------------------------------------
DmApoLTPI         2008 RCFWPQDTAYFKLNSNYDSDSSNFNHNL------------------------------------------
DmNlaz            212  -----------------------VSTTV------------------------------------------
DmApoLII          429  ---------------CLKNYCQGPEIDA------------------------------------------
DmApoLI           1312 SYTFMGNLGSKNGLGTLKITNKEFNTKF-------------------------------------SVCEE
DmMICOS_A         150  ---------------AGAVASMCYPRQA------------------------------------------
Cel_MICOS         152  ---------------LATMAAFCYPIEA------------------------------------------
Cel_Vit_2         704  ---------------QIGFSQQHMDKYV------------------------------------------
Cel_Vit_4         702  ---------------QVGFSQQNFEQVI------------------------------------------
Cel_Vit_5         702  ---------------QVGFSQQNFEQVI------------------------------------------
Cel_ApoL_1        174  ---------------VSLAGITGFGIKM------------------------------------------
Cel_MTTP          601  ---------------SRMRKFKVFRPNF------------------------------------------
Cel_Vit_6         733  ---------------QIGFTQKNMEKII------------------------------------------
Cel_Vit_3         702  ---------------QVGFSQQNFEQVI------------------------------------------
Cel_Vit-1         706  ---------------QIGFSQQHMDKYV------------------------------------------
Cel_Apo_L3_Like   192  ---------------GSVTGITGIGTRF------------------------------------------

HsApoA1           130  ----------------------------------------------------------------------
HsApoA2           92   ----------------------------------------------------------------------
HsApoA4           314  ----------------------------------------------------------------------
HsApoA5           275  ----------------------------------------------------------------------
HsApoB48          1164 RVAWHYDEEKIEFEWNTGTNVDTKKMTSNFPVDLSDYPKSLHMYANRLLDHRVPQTDMTFRHVGSKLIVA
HsApoB52          1187 LSSSSSVIDALQYKLEGTTRLTRKRGLKLATALSLSNKFVEGSHNSTVSLTTKNMEVSVATTTKAQIPIL
HsApoC1           109  ----------------------------------------------------------------------
HsApoC2           102  ----------------------------------------------------------------------
HsApoC3           88   ----------------------------------------------------------------------
HsApoC4           128  ----------------------------------------------------------------------
HsApoD            194  ----------------------------------------------------------------------
HsApoE2           261  ----------------------------------------------------------------------
HsApoF            257  ----------------------------------------------------------------------
HsApoH            264  ----------------------------------------------------------------------
HsApoJ            359  ----------------------------------------------------------------------
HsApoL1           297  ----------------------------------------------------------------------
HsApoL2           240  ----------------------------------------------------------------------
HsApoL3           240  ----------------------------------------------------------------------
HsApoL4           258  ----------------------------------------------------------------------
HsApoOL(2)        259  ----------------------------------------------------------------------
HsApoL6           201  ----------------------------------------------------------------------
HsApoM            161  ----------------------------------------------------------------------
HsApoO            189  ----------------------------------------------------------------------
HsApoOL           201  ----------------------------------------------------------------------
HsMTTP            589  --------------------------SKIVRRVLKEMVAHNYDRFSRSGSSSAYTGYIERSPRSASTYSL
DmCG31659         183  ----------------------------------------------------------------------
DmMtp             580  -----------------------------KSELVKRRHVNNYNVLGQKGLTTVLTRQLSQAPAFNETLLS
DmCvD             664  -----------------------------FFKFDTEALGKFTGQLALYIKARGLPDTILNKMQSRNGSDP
DmGlaz            164  ----------------------------------------------------------------------
DmFabp            116  ----------------------------------------------------------------------
DmApoLTPII        499  ----------------------------------------------------------------------
DmApoLTPI         2035 --------NGNVEIEVPLATRHRADIVYGLQKRRNQDAGNVKVVYNEKQVLDGKYKRLEQAKAPIYKETT
DmNlaz            217  ----------------------------------------------------------------------
DmApoLII          442  ----------------------------------------------------------------------
DmApoLI           1345 KRQCTNLIVQSIVSIDEQKLDAVEHTTLIIVDLRDFGYPYEFELKSQNTRQGLKYQYHLDSFIITGNNFK
DmMICOS_A         163  ----------------------------------------------------------------------
Cel_MICOS         165  ----------------------------------------------------------------------
Cel_Vit_2         717  --------------------------QMALEKLESLEKESTTVVRGRRIQTGIK---LLKELAQKMNIRA
Cel_Vit_4         715  --------------------------LKTLEKLSLYGKQSDE-LRSRRVQSGIQ---MLQEIVKKMNIRP
Cel_Vit_5         715  --------------------------LKTLEKLSLYGKQSDE-LRSRRVQSGIQ---MLQEIVKKMNIRP
Cel_ApoL_1        187  ----------------------------------------------------------------------
Cel_MTTP          614  ----------------------------LHRALQADSHVHWQEIADASNFQLFSTANTEFLQKSFKRSIF
Cel_Vit_6         746  --------------------------KKLLSNVQEKGLEQIV-VRGKRASGSFQPTEFLSNLLEKLRITR
Cel_Vit_3         715  --------------------------LKTLEKLSLYGKQSDE-LRSRRVQSGIQ---MLQEIVKKMNIRP
Cel_Vit-1         719  --------------------------QMALEKLESIEKESTTVVRGRRIQTGIT---LLKELALKMNIRA
Cel_Apo_L3_Like   205  ----------------------------------------------------------------------
```

```
HsApoA2          92   ----------------------------------------------------------------------
HsApoA4          314  ----------------------------------------------------------------------
HsApoA5          275  ----------------------------------------------------------------------
HsApoB48         1234 MSSWLQKASGSLPYTQTLQDHLNSLKEFNLQNMGLPDFHIPENLFLKSDGRVKYTLNKNSLKIEIPLPFG
HsApoB52         1257 RMNFKQELNGNTKSKPTVSSSMEFKYDFNSSMLYSTAKGAVDHKLSLESLTSYFSIESSTKGDVKGSVLS
HsApoC1          109  ----------------------------------------------------------------------
HsApoC2          102  ----------------------------------------------------------------------
HsApoC3          88   ----------------------------------------------------------------------
HsApoC4          128  ----------------------------------------------------------------------
HsApoD           194  ----------------------------------------------------------------------
HsApoE2          261  ----------------------------------------------------------------------
HsApoF           257  ----------------------------------------------------------------------
HsApoH           264  ----------------------------------------------------------------------
HsApoJ           359  ----------------------------------------------------------------------
HsApoL1          297  ---------------------------------------------------------------------H
HsApoL2          240  ----------------------------------------------------------------------
HsApoL3          240  ----------------------------------------------------------------------
HsApoL4          258  ----------------------------------------------------------------------
HsApoOL(2)       259  ----------------------------------------------------------------------
HsApoL6          201  ----------------------------------------------------------------------
HsApoM           161  ----------------------------------------------------------------------
HsApoO           189  ----------------------------------------------------------------------
HsApoOL          201  ----------------------------------------------------------------------
HsMTTP           634  DILYSGS-------------------------------------GILRRSNLNIFQYIGKAGLHGSQVVI
DmCG31659        183  ----------------------------------------------------------------------
DmMtp            622  TQEVYQG-------------------------------------ILKRGSVEFLLHAGRSQASSFKLGIY
DmCvD            706  FTFKSIKALLAMLQAPIINSKDLHLEFILQMEGKTVLSYYLNQRMFRQLTYDNILERMQQIIRTDSHINM
DmGlaz           164  ----------------------------------------------------------------------
DmFabp           116  ----------------------------------------------------------------------
DmApoLTPII       499  ------------------------------------------------------IINLLETEFLNLYNLF
DmApoLTPI        2098 DISLENEVKPLGIHFVSTRDASDPAGSQDVKHIEIYELRNTQNFNLTGELHSRATLKAQDFKVVAIHPNR
DmNlaz           217  ----------------------------------------------------------------------
DmApoLII         442  --------------------------------------------------ISKKFSDGLKHCKPNTKREE
DmApoLI          1415 YQFTANVQPTSSTIKLALPKRQILFETTQKIPADGSLFGRYEQTASFFIDKLQKPDDVARFSAIVDVTGT
DmMICOS_A        163  ----------------------------------------------------------------------
Cel_MICOS        165  ----------------------------------------------------------------------
Cel_Vit_2        759  RPATYTEKDAFAMVYLRYKDMDYAFLPIDRQLVENLIEKFTSNGKVQFSEIRRLLNQELEFETHHAAYFY
Cel_Vit_4        756  RVQQTDSQNAHAVFYLRYKEMDYIVLPIDMETIDNVVEKYVRNGEFDIKSLLTFLTNDSKFELHRALFFY
Cel_Vit_5        756  RVQQTDSQNAHAVFYLRYKEMDYIVLPIDMETIDTLVEKYVRNGEFDIKSLLTFLTNDSKFELHRALFFY
Cel_ApoL_1       187  ----------------------------------------------------------------------
Cel_MTTP         657  ELSMKKG---------------------------------------RKEHNLFSLSIDTEHLEQFVTGSA
Cel_Vit_6        790  R--QSSEQDPHAFVYIRHRDMDYAFLPIDADSIPEVVRSMIQGGRLEIGDIERVLAQGIHFSASNAAFLY
Cel_Vit_3        756  RVQQTDSQNAHAVFYLRYKEMDYIVLPIDMETIDNVVEKYVRNGEFDIKSLLTFLTNDSKFELHRALFFY
Cel_Vit-1        761  RPANYNEKDAFAMVYLRYKDMDYAILPVDTQLIEKLIEKYISNGKVQFSEIRRLLNQEHEFETHHAAYFY
Cel_Apo_L3_Like  205  ----------------------------------------------------------------------

HsApoA1          130  ----------------------------------------------------------------------
HsApoA2          92   ----------------------------------------------------------------------
HsApoA4          314  ----------------------------------------------------------------------
HsApoA5          275  ----------------------------------------------------------------------
HsApoB48         1304 GKSSRDLKMLETVRTPALHFKSVGFHLPSREFQVPTFTIPKLYQLQVPLLGVLDLSTNVYSNLYNWSASY
HsApoB52         1327 REYSGTIASEANTYLNSKSTRSSVKLQGTSKIDDIWNLEVKENFAGEATLQRIYSLWEHSTKNHLQLEGL
HsApoC1          109  ----------------------------------------------------------------------
HsApoC2          102  ----------------------------------------------------------------------
HsApoC3          88   ----------------------------------------------------------------------
HsApoC4          128  ----------------------------------------------------------------------
HsApoD           194  ----------------------------------------------------------------------
HsApoE2          261  ----------------------------------------------------------------------
HsApoF           257  ----------------------------------------------------------------------
HsApoH           264  ----------------------------------------------------------------------
HsApoJ           359  ----------------------------------------------------------------------
HsApoL1          299  ASASRPRVTEPISAESGEQVE-------------------------------------------------
HsApoL2          240  -----------PPPHVIGRISAE-----------------------------------------------
HsApoL3          240  -----------WRISAGSGGQAE-----------------------------------------------
HsApoL4          258  ---------IAWRYVPINVVETL-----------------------------------------------
HsApoOL(2)       259  -----------KNFVAKRHIPFW-----------------------------------------------
HsApoL6          201  -----------KRLLTTGQVSSR-----------------------------------------------
HsApoM           161  ----------------------------------------------------------------------
HsApoO           189  ----------------------------------------------------------------------
HsApoOL          201  ----------------------------------------------------------------------
HsMTTP           667  EAQGLEALIAATPDEGEENLDSYAGMSA------------------------------------------
DmCG31659        183  ----------------------------------------------------------------------
DmMtp            655  TAGLGSLVGDGDSGDGNDAIPAD-----------------------------------------------
DmCvD            776  QTVRWPFMNRYTVPTVLGTSSDVLLQTTVLTSLRGNITEQRNSPITKHTLEIDARYSSYASVRSRSYNPF
DmGlaz           164  ----------------------------------------------------------------------
DmFabp           116  ----------------------------------------------------------------------
DmApoLTPII       516  KGERRTRERMVILLKGLGNIGVVS----------------------------------------------
DmApoLTPI        2168 AVVLSTKYEDVSPEVVRHHSKLELSETAWIGYNLELGNFSKVGNESQSFALEIFYPKRNLSSSGQYYMTD
DmNlaz           217  ----------------------------------------------------------------------
DmApoLII         463  ERIVYILKGLGNAKSLSGNTVAALSECA------------------------------------------
DmApoLI          1485 ERVAFNANGKLKFEHPTIRPLSISGQLNGDVNQQIASAEVIFDIFRLPEQKVVGNSELRNSRSQNGFNIA
DmMICOS_A        163  ----------------------------------------------------------------------
Cel_MICOS        165  ----------------------------------------------------------------------
Cel_Vit_2        829  EAIRKFPTTLGLPLTISGKIPTVISAEGQFSLELEGT------ELRLTVEARPSVAATHVYEMRMFTPLF
Cel_Vit_4        826  EAERRIPTTIGMPLTISGKMPTILSINGKVSIELEKL------GARLVLDIVPTVATTHVTEMRFWYPVI
Cel_Vit_5        826  EAERRIPTTIGMPLTISGKMPTILSINGKVSIELEKL------GARLVLDIVPTVATTHVTEMRFWYPVI
Cel_ApoL_1       187  ----------------------------------------------------------------------
Cel_MTTP         688  SSRSGAPQGSVRIGVAGHKLPTHHIFKG------------------------------------------
Cel_Vit_6        858  ETVRRVPTPMGLPVQFTSKMPTISSIRGQVTFELEPKNGKSFDGLRLRVQAGPRVASTHVLSLRVICPIA
Cel_Vit_3        826  EAERRIPTTIGMPLTISGKMPTILSINGKVSIELEKL------GARLVLDIVPTVATTHVTEMRFWYPVI
Cel_Vit-1        831  EAIRKFPTTLGLPLIVSGKIPTVFSAEGQFSLGLEET------ELRLTVEARPSVAATHVYEMRMFTPLF
Cel_Apo_L3_Like  205  ----------------------------------------------------------------------
```

```
HsApoA2         92   ----------------------------------------------------------------------
HsApoA4         314  ----------------------------------------------------------------------
HsApoA5         275  ----------------------------------------------------------------------
HsApoB48        1374 SGGNTSTDHFSLRARYHMKADSVVDLLSYNVQGSGETTYDHKNTFTLSCDGSLRHKFLDSNIKFSHVEKL
HsApoB52        1397 FFTNGEHTSKATLELSPWQMSALVQVHASQPSSFHDFPDLGQEVALNANTKNQKIRWKNEVRIHSGSFQS
HsApoC1         109  ----------------------------------------------------------------------
HsApoC2         102  ----------------------------------------------------------------------
HsApoC3         88   ----------------------------------------------------------------------
HsApoC4         128  ----------------------------------------------------------------------
HsApoD          194  ----------------------------------------------------------------------
HsApoE2         261  ----------------------------------------------------------------------
HsApoF          257  ----------------------------------------------------------------------
HsApoH          264  ----------------------------------------------------------------------
HsApoJ          359  ----------------------------------------------------------------------
HsApoL1         319  ----------------------------------------------------------------------
HsApoL2         252  ----------------------------------------------------------------------
HsApoL3         252  ----------------------------------------------------------------------
HsApoL4         272  ----------------------------------------------------------------------
HsApoOL(2)      271  ----------------------------------------------------------------------
HsApoL6         213  ----------------------------------------------------------------------
HsApoM          161  ----------------------------------------------------------------------
HsApoO          189  ----------------------------------------------------------------------
HsApoOL         201  ----------------------------------------------------------------------
HsMTTP          694  ----------------------------------------------------------------------
DmCG31659       183  ----------------------------------------------------------------------
DmMtp           677  ----------------------------------------------------------------------
DmCvD           846  LNLDHEINREQGFLIYIPFSSELHLNESGSKCRRYSFSRPQNLTSGLSFKSRAVTK--------------
DmGlaz          164  ----------------------------------------------------------------------
DmFabp          116  ----------------------------------------------------------------------
DmApoLTPII      539  ----------------------------------------------------------------------
DmApoLTPI       2238 TNFNSDLSFQWLGGNYDQQPKIIHSNLQWKAEPLHRGDREHRTIALTVAHPLLEKDINCKATYYRGLRDL
DmNlaz          217  ----------------------------------------------------------------------
DmApoLII        490  ----------------------------------------------------------------------
DmApoLI         1555 YITTVKSAGLQFQYQINSNAAVDIEAHEYNIGLELNNGEIDVKAISFLNKEKFEISLSESNKHIIYIVGD
DmMICOS_A       163  ----------------------------------------------------------------------
Cel_MICOS       165  ----------------------------------------------------------------------
Cel_Vit_2       893  EQGVKSVQSVRAYTPIKIQAVAGMKRN-FEIVYKVVVPENQKSIVSLTTRPVVFLR--------------
Cel_Vit_4       890  EQGVKSLQSARLHTPLRFESTVELKKNTLEITHKFVVPENKKTTVSVHTRPVAFIR--------------
Cel_Vit_5       890  EQGVKSLQSARLHTPLRFESTVELKKNTLEITHKFVVPENKKTTVSVHTRPVAFIR--------------
Cel_ApoL_1      187  ----------------------------------------------------------------------
Cel_MTTP        715  ----------------------------------------------------------------------
Cel_Vit_6       928  EVGTKFLHQAVLNTPVDTEIRMNWEDK-VVIRAIYNTPSEEKRIAMIQSRPVTFTR--------------
Cel_Vit_3       890  EQGVKSLQSARLHTPLRFESTVELKKNTLEITHKFVVPENKKTTVSVHTRPVAFIR--------------
Cel_Vit-1       895  EQGVKSVQSVRAYTPIKIQAVVGMKRN-FEIVYKVVVPENQKSIISLTTRPVVFLR--------------
Cel_Apo_L3_Like 205  ----------------------------------------------------------------------

HsApoA1         130  ----------------------------------------------------------------------
HsApoA2         92   ----------------------------------------------------------------------
HsApoA4         314  ----------------------------------------------------------------------
HsApoA5         275  ----------------------------------------------------------------------
HsApoB48        1444 GNNPVSKGLLIFDASSSWGPQMSASVHLDSKKKQHLFVKEVKIDGQFRVSSFYAKGTYGLSCQRDPNTGR
HsApoB52        1467 QVELSNDQEKAHLDIAGSLEGHLRFLKNIILPVYDKSLWDFLKLDVTTSIGRRQHLRVSTAFVYTKNPNG
HsApoC1         109  ----------------------------------------------------------------------
HsApoC2         102  ----------------------------------------------------------------------
HsApoC3         88   ----------------------------------------------------------------------
HsApoC4         128  ----------------------------------------------------------------------
HsApoD          194  ----------------------------------------------------------------------
HsApoE2         261  ----------------------------------------------------------------------
HsApoF          257  ----------------------------------------------------------------------
HsApoH          264  ----------------------------------------------------------------------
HsApoJ          359  ----------------------------------------------------------------------
HsApoL1         319  ----------------------------------------------------------------------
HsApoL2         252  ----------------------------------------------------------------------
HsApoL3         252  ----------------------------------------------------------------------
HsApoL4         272  ----------------------------------------------------------------------
HsApoOL(2)      271  ----------------------------------------------------------------------
HsApoL6         213  ----------------------------------------------------------------------
HsApoM          161  ----------------------------------------------------------------------
HsApoO          189  ----------------------------------------------------------------------
HsApoOL         201  ----------------------------------------------------------------------
HsMTTP          694  ----------------------------------------------------------------------
DmCG31659       183  ----------------------------------------------------------------------
DmMtp           677  ----------------------------------------------------------------------
DmCvD           902  TRGLITKTAAAPFEEIMVPEGRNDVVQLFSYPMTDLGVRLSMTTNLNELIKYRGMLLKSEFTENGFSGNM
DmGlaz          164  ----------------------------------------------------------------------
DmFabp          116  ----------------------------------------------------------------------
DmApoLTPII      539  ----------------------------------------------------------------------
DmApoLTPI       2308 LRTHLTIDYSEYPDQLIELGAQLTDRYSELGHTNYTFHVYGKHIASELDVQLNGTLAAMNSYYKTESTAH
DmNlaz          217  ----------------------------------------------------------------------
DmApoLII        490  ----------------------------------------------------------------------
DmApoLI         1625 FSKQNHYAKLNTKVQILDKNPIEITSEVQPNSAKIILKRQDFIDGTAEVKLGKEFKVDVIGSGKQLFNGR
DmMICOS_A       163  ----------------------------------------------------------------------
Cel_MICOS       165  ----------------------------------------------------------------------
Cel_Vit_2       948  FPGFSKFEYIEAEERTVVVPQWQQKTQEIEKVFNFLGLEVSTRGNILNQHTLENWLLAEQDFEVSVENKN
Cel_Vit_4       946  VPKNQDSEYVEAEEKTISHSQYQMSTEEIDRQYETFGLRINAQGNVLSQWTLPMVLMTEQDFEFTLENKN
Cel_Vit_5       946  VPKNQDSEYVEAEEKTISHSQYQMSTEEIDRQYETFGLRINAQGNVLSQWTLPMVLMTEQDFEYTLENKN
Cel_ApoL_1      187  ----------------------------------------------------------------------
Cel_MTTP        715  ----------------------------------------------------------------------
Cel_Vit_6       983  TVAPDARQYPEPIEMTYMLPAHKQLSQSLDREYPQ--IRVQGTLNRPTSVRIPQWIV---DSNVEVYYKP
Cel_Vit_3       946  VPKNQDSEYVETEEKTISHSQYQMSTEEIDRQYETFGLRINAQGNVLSQWTLPMVLMTEQDFEFTLENKN
Cel_Vit-1       950  FPGFSKFEYIEAEERTVVVPQWQQKTQEIEKVFNFLGLEVSTRGNILNQHTLENWLLAEQDFEVSVENKY
Cel_Apo_L3_Like 205  ----------------------------------------------------------------------
```

```
HsApoA2         92   ----------------------------------------------------------------------
HsApoA4         314  ----------------------------------------------------------------------
HsApoA5         275  ----------------------------------------------------------------------
HsApoB48        1514 LNGESNLRFNSSYLQGTNQITGRYEDGTLSLTSTSDLQSGIIKNTASLKYENYELTLKSDTNGKYKNFAT
HsApoB52        1537 YSFSIPVKVLADKFIIPGLKLNDLNSVLVMPTFHVPFTDLQVPSCKLDFREIQIYKKLRTSSFALNLPTL
HsApoC1         109  ----------------------------------------------------------------------
HsApoC2         102  ----------------------------------------------------------------------
HsApoC3         88   ----------------------------------------------------------------------
HsApoC4         128  ----------------------------------------------------------------------
HsApoD          194  -------------------------------------------MEKVKS---------------------
HsApoE2         261  -------------------------------------------EEQAQQ---------------------
HsApoF          257  ----------------------------------------------------------------------
HsApoH          264  -------------------------------------------KVPVKK---------------------
HsApoJ          359  ----------------------------------------------------------------------
HsApoL1         319  ----------------------------------------------------------------------
HsApoL2         252  -------------------------------------------GGEQVE---------------------
HsApoL3         252  ----------------------------------------------------------------------
HsApoL4         272  ----------------------------------------------------------------------
HsApoOL(2)      271  -------------------------------------------TARGVQ---------------------
HsApoL6         213  -------------------------------------------SRVQVQ---------------------
HsApoM          161  ----------------------------------------------------------------------
HsApoO          189  ----------------------------------------------------------------------
HsApoOL         201  ----------------------------------------------------------------------
HsMTTP          694  ----------------------------------------------------------------------
DmCG31659       183  ----------------------------------------------------------------------
DmMtp           677  -------------------------------------------DEFSED---------------------
DmCvD           972  VVNALMYIFGFTQLSSIHLGHDRNFTMLMYNEKNTRIEGNFCAEDVLKT---------------------
DmGlaz          164  ----------------------------------------------------------------------
DmFabp          116  ----------------------------------------------------------------------
DmApoLTPII      539  ----------------------------------------------------------------------
DmApoLTPI       2378 YKRDIFPARYGKFLALLDVNKRELEYERQSPFHAVRLHLLPTIRYPIYGLNATIWDTPDTNHSGYIYMDI
DmNlaz          217  ----------------------------------------------------------------------
DmApoLII        490  -------------------------------------------STGRSN---------------------
DmApoLI         1695 VALDATNFLQTNYFINEDHLNGFWHIVESEINKDSEYISENIKERLKKSRQVTDKIVKLAKEAGPDFSKL
DmMICOS_A       163  -------------------------------------------EENCRV---------------------
Cel_MICOS       165  ----------------------------------------------------------------------
Cel_Vit_2       1018 RPAEFTARLTVGQLEKTELSQIKYNKIFEKE-----FELEQE-NTESRR---------------------
Cel_Vit_4       1016 RPVEFTARVTIGNLEKTDLSEIKFDKIFEKE-----FDLENN-ESENRR---------------------
Cel_Vit_5       1016 RPVEFTARVTIGNLEKTDLSEIKFDKIFEKE-----FDLENN-ESENRR---------------------
Cel_ApoL_1      187  -------------------------------------------AASSMG---------------------
Cel_MTTP        715  -------------------------------------------STDLLS---------------------
Cel_Vit_6       1048 NVEQYEAIFELNLYNNYKMEK-NYEKVYKKHNGRRYLEAEPEYDEEEHR---------------------
Cel_Vit_3       1016 RPVEFTARVTIGNLEKTDLSEIKFDKIFEKE-----FDLENN-ESENRR---------------------
Cel_Vit-1       1020 RPAEFTARLTVGQLEKTELSQIKYNKIFEKE-----FELEQE-NTESRR---------------------
Cel_Apo_L3_Like 205  -------------------------------------------AITSMG---------------------

HsApoA1         130  ----------------------------------------------------------------------
HsApoA2         92   ----------------------------------------------------------------------
HsApoA4         314  ----NKALVQQMEQLRQKLGPHAGDVEGHLSFLE------------------------------------
HsApoA5         275  -----MLSEEVRQRLQAFRQDTYLQIAAFTRAID------------------------------------
HsApoB48        1584 SNKMDMTFSKQNALLRSEYQADYESLRFFSLLSGSLNSHGLELNADILGTDKINSGAHKATLRIGQDGIS
HsApoB52        1607 PEVKFPEVDVLTKYSQPEDSLIPFFEITVPESQLTVSQFTLPKSVSDGIAALDLNAVANKIADFELPTII
HsApoC1         109  ----------------------------------------------------------------------
HsApoC2         102  ----------------------------------------------------------------------
HsApoC3         88   ----------------------------------------------------------------------
HsApoC4         128  ----------------------------------------------------------------------
HsApoD          200  ----------------------------------------------------------------------
HsApoE2         267  -----IRLQAEAFQARLKSWFE------------------------------------------------
HsApoF          257  -----QYYQDQKDANISQPETTKEGLRAI-----------------------------------------
HsApoH          270  -----ATVVYQGERVKIQEKFKNGMLHGDKVS--------------------------------------
HsApoJ          359  -----EQLNEQFNWVSRLANLTQGEDQYYLRVTTVASHTSDSDVP-------------------------
HsApoL1         319  -----RVNEPSILEMSRGVKLTDVAPVSFFLV--------------------------------------
HsApoL2         258  -----RVVEGPAQAMSRGTMIVGAATGGILLL--------------------------------------
HsApoL3         252  -----RTIAGTTRAVSRGARILSATTSGIFLA--------------------------------------
HsApoL4         272  -----RTRGAPTRIVRKVARNLGKATSGVLVV--------------------------------------
HsApoOL(2)      277  -----RAFEGTTLAMTNGAWVMGAAGAGFLLM--------------------------------------
HsApoL6         219  -----KAFAGTTLAMTKNARVLGGVMSAFSLG--------------------------------------
HsApoM          161  ----------------------------------------------------------------------
HsApoO          189  ----------------------------------------------------------------------
HsApoOL         201  ---------GSSSEIEVPAKTTHVLKHSVPLP--------------------------------------
HsMTTP          694  -----ILFDVQLRPVTFFNGYSDLMSKMLSASGDPI----------------------------------
DmCG31659       183  ----------------------------------------------------------------------
DmMtp           683  -----EAVTAGMEISVQGAQLRPLVFFSGQTELMGHVWGGSASDST------------------------
DmCvD           1020 -----SDMKGKQIGLTLEHTDHMNENHAADALHRWNITLDVLASTKSNWFKLTGQVQRNSKDDEDDW---
DmGlaz          164  --------------HSDQIWILGRD---------------------------------------------
DmFabp          116  ----------------------------------------------------------------------
DmApoLTPII      539  -----SAFAEQLQWIIREDEAPVDIRLHGILAFRRVDCARHRSYFLDNYGNYTLNSELRIYSYLQAMRC-
DmApoLTPI       2448 LERYARMDFNLTEDASQNLQMVGYIPDTRSAFLDIWRNYEEIRVIDVSSYLKMNHSRLITGRFHWRPSIR
DmNlaz          217  ----------------------------------------------------------------------
DmApoLII        496  -----RIRVAALHAFSKVKCEETLQSKSLELLKNRNEDSELRIEAYLSAISC------------------
DmApoLI         1765 QGKLLDYKNDIVQELEADQSIAPIIDGIRTLFKKIAGIVDDINKAISEILEKAQKSIVDIYDKLQALWKD
DmMICOS_A       169  -----VLYEGRKIFAVAYNFIKGVKPGEDVPV--------------------------------------
Cel_MICOS       165  ----------------------------------------------------------------------
Cel_Vit_2       1060 -----EYFNKMVKNIQKEQGYKSVISLKLEAPRDYTMNTELTTVCDKQVRMCQWEVEIRRSPILEETKEW
Cel_Vit_4       1058 -----QYFHKMIREIQSEQGFKNLITLKLEAPQQMYWNTELRTVCDKWIRMCKVEMDARRSPIEHENKEW
Cel_Vit_5       1058 -----QYFHKMIREIQSEQGFKNLITLKLEAPQQMYWNTELRTVCDKWIRMCKVEMDARRSPMEHENKEW
Cel_ApoL_1      193  -----HLTNALMKGMLQSVAAIGIV---------------------------------------------
Cel_MTTP        721  -----TVWEADGRTHKAFEGHVPVRDVRLSVPLL------------------------------------
Cel_Vit_6       1095 -----EQITKKFEWLQNEKVYQHVAKFEIKPE---VVKMEVEAVCNNDFHFCKTQI-----------RGE
Cel_Vit_3       1058 -----QYFHKMIREIQSEQGFKNLITLKLEAPQQMYWNTELRTVCDKWIRMCKVEMDARRSPMEHENKEW
Cel_Vit-1       1062 -----EYFTKMVKSIQKEQGYKSVVSLRLEAPRDYTMNTEVTTVCDKQVRMCQWEVEIRRSPILEETKEW
Cel_Apo_L3_Like 211  -----RLSSSLVKGVLHSVAVIGIV---------------------------------------------
```

```
HsApoA2         92   ----------------------------------------------------------------------
HsApoA4         344  -------------------------------------------------KDLRDKVNSFFSTFKEKESQD
HsApoA5         304  -------------------------------------------------QETEEVQQQLAPPPPGHSAFA
HsApoB48        1654 TSATTNLKCSLLVLENELNAELGLSG-----------------------ASMKLTTNGRFREHNAKFSLD
HsApoB52        1677 VPEQTIEIPSIKFSVPAGIVIPSFQALTARFEVDSPVYNATWSASLKNKADYVETVLDSTCSSTVQFLEY
HsApoC1         109  -------------------------------------------------LDLKGVVFRDISESEGETQD-
HsApoC2         102  ----------------------------------------------------------------------
HsApoC3         88   -------------------------------------------------PEVRPTSAVAAE---------
HsApoC4         128  ----------------------------------------------------------------------
HsApoD          200  -------------------------------------------------PELQAEAKSYFEKSKEQLTPL
HsApoE2         284  -------------------------------------------------PLVEDMQRQWAGLVEKVQAAV
HsApoF          281  -------------------------------------------------SDVSDLEETTTLASFISEVVS
HsApoH          297  -------------------------------------------------FFCKNKEKKCSYTEDAQCIDG
HsApoJ          399  -------------------------------------------------SGVTEVVVKLFDSDPITVTVP
HsApoL1         346  -------------------------------------------------LDVVYLVYESKHLHEGAKSET
HsApoL2         285  -------------------------------------------------LDVVSLAYESKHLLEGAKSES
HsApoL3         279  -------------------------------------------------LDVVNLVYESKHLHEGAKSAS
HsApoL4         299  -------------------------------------------------LDVVNLVQDSLDLHKGEKSES
HsApoOL(2)      304  -------------------------------------------------KDMSSFLQSWKHLEDGARTET
HsApoL6         246  -------------------------------------------------YDLATLSKEWKHLKEGARTKF
HsApoM          161  -------------------------------------------------KSLTSCLDSKAFLLTPRNQEA
HsApoO          189  ----------------------------------------------------------------------
HsApoOL         224  -------------------------------------------------TELSSEAKTKSESTSGATQFM
HsMTTP          725  -------------------------------------------------SVVKGLILLIDHSQELQLQSG
DmCG31659       183  ----------------------------------------------------------------------
DmMtp           724  -------------------------------------------------PAYQATTLSQDNEHYIILTSG
DmCvD           1082 -------------------------------------------------KACTKLTYEPLVFTKRPHTLN
DmGlaz          175  -------------------------------------------------RDFEVDIRSKVYDVLKRLSLD
DmFabp          116  -------------------------------------------------PHLTPSQHGSLRLRVPCQPVP
DmApoLTPII      603  -------------------------------------------------PDYISVGVIKSILEHEEINQV
DmApoLTPI       2518 QEVREKIQAVGKSVY----------------------------------SSFSEGIDFWIKSIYTETTES
DmNlaz          217  -------------------------------------------------PNAIEKA-EWLRR--------
DmApoLII        543  -------------------------------------------------PNAEVANQISEIVNSETVNQV
DmApoLI         1835 SLLKAWEDFIITVQKLISTLKTEFIKICTQSFKDLL-------------SALEKYGPALKNYGKAIGEIV
DmMICOS_A       196  -------------------------------------------------VPFPTSLEDLKYMASD-----
Cel_MICOS       165  -------------------------------------------------VDVAKTGRAHAEQTWYSFQES
Cel_Vit_2       1126 TLRSQLLVVR---------------------------------------PEMPSSLRQLRDQPHREVQLS
Cel_Vit_4       1124 TLRTELLAAR---------------------------------------PQMPSSLRQLREQPHREVQLA
Cel_Vit_5       1124 TLRTELLAAR---------------------------------------PQMPSSLRQLREQPHREVQLA
Cel_ApoL_1      213  -------------------------------------------------LDGVTLAMSANTLKNGSSSEL
Cel_MTTP        750  -------------------------------------------------SGLTLDVDSVGAISMRVLASA
Cel_Vit_6       1147 ELKATIQYVY---------------------------------------PQTPRTVEELKEQKYRQLVVM
Cel_Vit_3       1124 TLRTELLAAR---------------------------------------PQMPSSLRQLREQPHREVQLA
Cel_Vit-1       1128 TLRSQLLVVR---------------------------------------PEMPSSLRQLHDQPHREVQLS
Cel_Apo_L3_Like 231  -------------------------------------------------LDSVTLALSAKTLGEGSVSEL

HsApoA1         150  ----------------------------------------------------------------------
HsApoA2         92   ----------------------------------------------------------------------
HsApoA4         366  KTLSLPELEQQQEQQQEQQQE-------------------------------------------------
HsApoA5         326  PEFQQTDSGKVLSKLQARLDD-------------------------------------------------
HsApoB48        1701 GKAALTELSLGSAYQAMILGVDSKNIFNFKVSQEGLKLSNDMMGSYAEMKFDHTNSLNIAGLSLDFSSKL
HsApoB52        1747 ELNVLGTHKIEDGTLASKTKGTFAHRDFSAEYEEDGKYEGLQEWEGKAHLNIKSPAFTDLHLRYQKDKKG
HsApoC1         129  ----------------------------------------------------------------------
HsApoC2         102  ----------------------------------------------------------------------
HsApoC3         100  ----------------------------------------------------------------------
HsApoC4         128  ----------------------------------------------------------------------
HsApoD          222  IKKAGTELVNFLSYF-------------------------------------------------------
HsApoE2         306  GT--------------------------------------------------------------------
HsApoF          303  SAPYWGWAIIKSYD--------------------------------------------------------
HsApoH          319  TIEVPKCFKEHSSLA-------------------------------------------------------
HsApoJ          421  VEVSRKNPKFMETVAEKAL---------------------------------------------------
HsApoL1         368  AEELKKVAQE------------------------------------------------------------
HsApoL2         307  AEELKKRAQE------------------------------------------------------------
HsApoL3         301  AEELRRQAQE------------------------------------------------------------
HsApoL4         321  AELLRQWAQE------------------------------------------------------------
HsApoOL(2)      326  AEELRALAKK------------------------------------------------------------
HsApoL6         268  AEELRAKA--------------------------------------------------------------
HsApoM          183  CELSNNW---------------------------------------------------------------
HsApoO          189  ----------------------------------------------------------------------
HsApoOL         246  PDPKLMDHGQSHPE--------------------------------------------------------
HsMTTP          747  LKANIEVQGGLAIDISGA----------------------------------------------------
DmCG31659       183  ----------------------------------------------------------------------
DmMtp           746  ATLHWRVLGARSVDLNGKVGFS------------------------------------------------
DmCvD           1104 GDVVFGLATEESECPEKGSTVQFAARAGPSEHARAFLRSDKISLTDTDFCPKEVLKFSPIPTSRYCKRSN
DmGlaz          197  PERLIIS---------------------------------------------------------------
DmFabp          138  PTSVDDGARSGG----------------------------------------------------------
DmApoLTPII      625  GSFVWSHLTNLAKSNSPVRIEAQGLL--------------------------------------------
DmApoLTPI       2554 MGVVWNTAKEYNRDFIDDIGQLSVLEEDLADLRLFVNQSYEANDFYIKNVVNFTLTILDELAIRDHIESL
DmNlaz          229  ----------------------------------------------------------------------
DmApoLII        565  GGFISSNLKAIRDSTDVS----------------------------------------------------
DmApoLI         1892 KPINDAAQEVIKIVVNAAEGVTHEFKQYVASLPSFESIRNEFNDKVKVLKLFEKATELTNSLFDQINILP
DmMICOS_A       212  ----------------------------------------------------------------------
Cel_MICOS       187  PTPSAI----------------------------------------------------------------
Cel_Vit_2       1157 LTSTWGSQKKSEVTVNAQLQQSKEQKKYERNMDRQFNGMPEYELLIKAARLNQINAVAEYKLTRETEQVL
Cel_Vit_4       1155 LNAKWGSSKKSEITFNAQLEQSTEQKKFLRNIEREYKGIPEYELLIKAARLNQVNVVSEYKLTPESEYTF
Cel_Vit_5       1155 FNAKWGSSKKSEITVNAQLEQSTEQKKFIRNIEREYKGIPEYELLIKAARLNQVNVVSEYKLTPQSEYTF
Cel_ApoL_1      235  AGKIREASGKMERMRHNVVTNF------------------------------------------------
Cel_MTTP        772  EVSLWNQRSNAKAEAYTSGSLH------------------------------------------------
Cel_Vit_6       1178 GEMNYG---ENTIHININGQQSQEQKKFVKQIEQA----PEHETLLEASRLDQYQTVVEYEFEPKPAQYF
Cel_Vit_3       1155 LNAKWGSSKKSEITFNAQLEQSTEQKKFLRNIEREYKGIPEYELLIKAARLNQVNVVSEYKLTPESEYTF
Cel_Vit-1       1159 LTSTWGSQKKSEVTVNAQLQQSKEQKKYERNMDRHFNGMPEYELLIKAARLNQINAVAEYKLTRETEQVL
Cel_Apo_L3_Like 253  GSSILEASSKMEMMRQKVVKHF------------------------------------------------
```

```
HsApoA2         92    ----------------------------------------------------------------------
HsApoA4         386   ----------------------------------------------------------------------
HsApoA5         346   ----------------------------------------------------------------------
HsApoB48        1771  DNIYSSDKFYKQTVNLQLQPYSLVTTLNSDLKYNALDLTNNGKLRLEPLKLHVAGNLKGAYQNNEIKHIY
HsApoB52        1817  ISTSAASPAVGTVGMDMDEDDDFSKWNFYYSPQSSPDKKLTIFKTELRVRESDEETQIKVNWEEEAASGL
HsApoC1         129   ----------------------------------------------------------------------
HsApoC2         102   ----------------------------------------------------------------------
HsApoC3         100   ----------------------------------------------------------------------
HsApoC4         128   ----------------------------------------------------------------------
HsApoD          236   ----------------------------------------------------------------------
HsApoE2         307   ----------------------------------------------------------------------
HsApoF          316   ----------------------------------------------------------------------
HsApoH          333   ----------------------------------------------------------------------
HsApoJ          439   ----------------------------------------------------------------------
HsApoL1         377   ----------------------------------------------------------------------
HsApoL2         316   ----------------------------------------------------------------------
HsApoL3         310   ----------------------------------------------------------------------
HsApoL4         330   ----------------------------------------------------------------------
HsApoOL(2)      335   ----------------------------------------------------------------------
HsApoL6         275   ----------------------------------------------------------------------
HsApoM          189   ----------------------------------------------------------------------
HsApoO          189   ----------------------------------------------------------------------
HsApoOL         259   ----------------------------------------------------------------------
HsMTTP          764   ----------------------------------------------------------------------
DmCG31659       183   ----------------------------------------------------------------------
DmMtp           767   ----------------------------------------------------------------------
DmCvD           1174  FENFTSITQYDMDLKFDNMPAWFELWSNRLDHLVSALSADKVDSLHMSQEINISMQTPQDQFRLAVEVNG
DmGlaz          203   ----------------------------------------------------------------------
DmFabp          149   ----------------------------------------------------------------------
DmApoLTPII      650   ----------------------------------------------------------------------
DmApoLTPI       2624  PKIFSELWQAMGDSGKALRNSIVWLIETIKTTYNNLLDAVARFFHGESLVYISGLLEKGIAKYDSFIKDL
DmNlaz          229   ----------------------------------------------------------------------
DmApoLII        582   ----------------------------------------------------------------------
DmApoLI         1962  QTPETSEFLQKLHDYLIAKLKQEHIDNEKYIEELGQLLIKAVRSIWVSIRSTYPGSSDHVIDFQSWIGSL
DmMICOS_A       212   ----------------------------------------------------------------------
Cel_MICOS       192   ----------------------------------------------------------------------
Cel_Vit_2       1227  ARYFDLVKTY---NY-WT--VSSRPENNENDRVVVQLTVEPMSRQYVNITMQSPMERIELKNVQVPRVYL
Cel_Vit_4       1225  SRIFDLIKAY---NF-WT--VSEKRVQNEDRRVVLQLSVEPLSRQYMNMTIQTPEQEVELKNVRIPRVVL
Cel_Vit_5       1225  SRIFDLIKAY---NF-WT--VSEKRVQNENRRVVLQLSVEPLSRQYMNMTIQTPEQEVELKNVRIPRVVL
Cel_ApoL_1      256   ----------------------------------------------------------------------
Cel_MTTP        793   ----------------------------------------------------------------------
Cel_Vit_6       1241  ARYWNMVQAYLRTQYPWTSRIETREEPSRKNMIRATINVEPRQRLTVNMTIETPMETTVLERVELP-FRL
Cel_Vit_3       1225  SRIFDLIKAY---NF-WT--VSEKRVQNEDRRVVLQLSVEPLSRQYMNMTIQTPEQEVELKNVRIPRVVL
Cel_Vit-1       1229  ARYFDLVKAY---NY-WT--VSSRPENNENDRVVVQLTVEPMSRQYVNITMQSPIERVELKNVQVPRVYL
Cel_Apo_L3_Like 274   ----------------------------------------------------------------------

HsApoA1         150   -----------------------YTKKLNTQ---------------------------------------
HsApoA2         92    -----------------------LGTQPATQG--------------------------------------
HsApoA4         386   -----------------------QVQMLAPLES-------------------------------------
HsApoA5         346   -----------------------LWEDITHSLH-------------------------------------
HsApoB48        1841  AISSAALSASYKADTVAKVQGVEFSHRLNTDIA-------------------------------------
HsApoB52        1887  LTSLKDNVPKATGVLYDY-----VNKYHWEHTG-------------------------------------
HsApoC1         129   ----------------------------------------------------------------------
HsApoC2         102   ----------------------------------------------------------------------
HsApoC3         100   ----------------------------------------------------------------------
HsApoC4         128   ----------------------------------------------------------------------
HsApoD          236   -----------------------VELGTQPATQ-------------------------------------
HsApoE2         307   -----------------------SAAPVPSDNH-------------------------------------
HsApoF          316   -----------------------LDPGAGSLEI-------------------------------------
HsApoH          333   -----------------------FWKTDASDVK-------------------------------------
HsApoJ          439   -----------------------QEYRKKHREE-------------------------------------
HsApoL1         377   -----------------------LEEKLNILNN-------------------------------------
HsApoL2         316   -----------------------LEGKLNFLTK-------------------------------------
HsApoL3         310   -----------------------LEENLMELTQ-------------------------------------
HsApoL4         330   -----------------------LEENLNELTH-------------------------------------
HsApoOL(2)      335   -----------------------LEQELDRLTQ-------------------------------------
HsApoL6         275   ----------------LE-----LERKLTELTQ-------------------------------------
HsApoM          189   ----------------------------------------------------------------------
HsApoO          189   -----------------------NVKNSPGTKN-------------------------------------
HsApoOL         259   -----------------------DIDMYSTRSE-------------------------------------
HsMTTP          764   -----------------------MEFSLWYRES-------------------------------------
DmCG31659       183   -----------------------PQESCPYDTS-------------------------------------
DmMtp           767   -----------------------LWNRNAQTEI-------------------------------------
DmCvD           1244  VKWRFHQIPFFYKLDSKFD----ASHELTFDSG-------------------------------------
DmGlaz          203   -----------------------KNKQCPEALI-------------------------------------
DmFabp          149   -----------------------CGQRPGGPGS-------------------------------------
DmApoLTPII      650   -----------------------LNDELSERFK-------------------------------------
DmApoLTPI       2694  HIKFIKYIEN-------------LWHKTWTLAENHWKAVLKRFEPHLFKMISFIETTAWNLSKEVFDFIY
DmNlaz          229   -----------------------LYERLYDIFM-------------------------------------
DmApoLII        582   -----------------------RDQQKYHLAN-------------------------------------
DmApoLI         2032  THSFDSLAVLPSILSFRSSILNCLLNENWDVVFNKKLLYSWIFFNDFELRGHVVDGKHIFTFDGLNFAYP
DmMICOS_A       212   -----------------------LYDEAKDLIF-------------------------------------
Cel_MICOS       192   -----------------------VKTNLSPPKS-------------------------------------
Cel_Vit_2       1291  PSI-----------AQRS-----VKHQLTEASG-------------------------------------
Cel_Vit_4       1289  PTI-----------ARRA-----MFQQTWEKTG-------------------------------------
Cel_Vit_5       1289  PTI-----------ARSA-----MFQQTWEKTG-------------------------------------
Cel_ApoL_1      256   -----------------------LNYDAWNSDV-------------------------------------
Cel_MTTP        793   -----------------------LTASLYHHSE-------------------------------------
Cel_Vit_6       1310  PTAQIHYQPRNSRYEQKP-----VMEKIAHHAS-------------------------------------
Cel_Vit_3       1289  PTI-----------ARRA-----MFQQTWEKTG-------------------------------------
Cel_Vit-1       1293  PSI-----------AQRS-----VKHLLNEASG-------------------------------------
Cel_Apo_L3_Like 274   -----------------------LNEDVWKDDD-------------------------------------
```

```
HsApoA2          101  ----------------------------------------------------------------------
HsApoA4          396  ----------------------------------------------------------------------
HsApoA5          356  ----------------------------------------------------------------------
HsApoB48         1873 ----------------------------------------------------------------------
HsApoB52         1914 ----------------------------------------------------------------------
HsApoC1          129  ----------------------------------------------------------------------
HsApoC2          102  ----------------------------------------------------------------------
HsApoC3          100  ----------------------------------------------------------------------
HsApoC4          128  ----------------------------------------------------------------------
HsApoD           246  ----------------------------------------------------------------------
HsApoE2          317  ----------------------------------------------------------------------
HsApoF           326  ----------------------------------------------------------------------
HsApoH           343  ----------------------------------------------------------------------
HsApoJ           449  ----------------------------------------------------------------------
HsApoL1          387  ----------------------------------------------------------------------
HsApoL2          326  ----------------------------------------------------------------------
HsApoL3          320  ----------------------------------------------------------------------
HsApoL4          340  ----------------------------------------------------------------------
HsApoOL(2)       345  ----------------------------------------------------------------------
HsApoL6          287  ----------------------------------------------------------------------
HsApoM           189  ----------------------------------------------------------------------
HsApoO           199  ----------------------------------------------------------------------
HsApoOL          269  ----------------------------------------------------------------------
HsMTTP           774  ----------------------------------------------------------------------
DmCG31659        193  ----------------------------------------------------------------------
DmMtp            777  ----------------------------------------------------------------------
DmCvD            1272 ----------------------------------------------------------------------
DmGlaz           213  ----------------------------------------------------------------------
DmFabp           159  ----------------------------------------------------------------------
DmApoLTPII       660  ----------------------------------------------------------------------
DmApoLTPI        2751 KRTNELAESPYFNKVSSFTADAERLYRDFKANDAITNIKKYSTIAWNFVKEKYFKLVPFGAELNEVLTEI
DmNlaz           239  ----------------------------------------------------------------------
DmApoLII         592  ----------------------------------------------------------------------
DmApoLI          2102 GNCKYILAQDSVDNNFTIIGQ-------------------------------------------------
DmMICOS_A        222  ----------------------------------------------------------------------
Cel_MICOS        202  ----------------------------------------------------------------------
Cel_Vit_2        1307 ----------------------------------------------------------------------
Cel_Vit_4        1305 ----------------------------------------------------------------------
Cel_Vit_5        1305 ----------------------------------------------------------------------
Cel_ApoL_1       266  ----------------------------------------------------------------------
Cel_MTTP         803  ----------------------------------------------------------------------
Cel_Vit_6        1337 ----------------------------------------------------------------------
Cel_Vit_3        1305 ----------------------------------------------------------------------
Cel_Vit-1        1309 ----------------------------------------------------------------------
Cel_Apo_L3_Like  284  ----------------------------------------------------------------------

HsApoA1          158  ----------------------------------------------------------------------
HsApoA2          101  ----------------------------------------------------------------------
HsApoA4          396  ----------------------------------------------------------------------
HsApoA5          356  ----------------------------------------------------------------------
HsApoB48         1873 ----------------------------------------------------------------------
HsApoB52         1914 ----------------------------------------------------------------------
HsApoC1          129  ----------------------------------------------------------------------
HsApoC2          102  ----------------------------------------------------------------------
HsApoC3          100  ----------------------------------------------------------------------
HsApoC4          128  ----------------------------------------------------------------------
HsApoD           246  ----------------------------------------------------------------------
HsApoE2          317  ----------------------------------------------------------------------
HsApoF           326  ----------------------------------------------------------------------
HsApoH           343  ----------------------------------------------------------------------
HsApoJ           449  ----------------------------------------------------------------------
HsApoL1          387  ----------------------------------------------------------------------
HsApoL2          326  ----------------------------------------------------------------------
HsApoL3          320  ----------------------------------------------------------------------
HsApoL4          340  ----------------------------------------------------------------------
HsApoOL(2)       345  ----------------------------------------------------------------------
HsApoL6          287  ----------------------------------------------------------------------
HsApoM           189  ----------------------------------------------------------------------
HsApoO           199  ----------------------------------------------------------------------
HsApoOL          269  ----------------------------------------------------------------------
HsMTTP           774  ----------------------------------------------------------------------
DmCG31659        193  ----------------------------------------------------------------------
DmMtp            777  ----------------------------------------------------------------------
DmCvD            1272 ----------------------------------------------------------------------
DmGlaz           213  ----------------------------------------------------------------------
DmFabp           159  ----------------------------------------------------------------------
DmApoLTPII       660  ----------------------------------------------------------------------
DmApoLTPI        2821 WQEIKELEKIDQVQIMVQKYYEVMAKVDWVADELQLEHRLHQVYGLVRNKFRNYAMNALETADMYREAKT
DmNlaz           239  ----------------------------------------------------------------------
DmApoLII         592  ----------------------------------------------------------------------
DmApoLI          2122 ----------------------------------------------------------------------
DmMICOS_A        222  ----------------------------------------------------------------------
Cel_MICOS        202  ----------------------------------------------------------------------
Cel_Vit_2        1307 ----------------------------------------------------------------------
Cel_Vit_4        1305 ----------------------------------------------------------------------
Cel_Vit_5        1305 ----------------------------------------------------------------------
Cel_ApoL_1       266  ----------------------------------------------------------------------
Cel_MTTP         803  ----------------------------------------------------------------------
Cel_Vit_6        1337 ----------------------------------------------------------------------
Cel_Vit_3        1305 ----------------------------------------------------------------------
Cel_Vit-1        1309 ----------------------------------------------------------------------
Cel_Apo_L3_Like  284  ----------------------------------------------------------------------
```

```
HsApoA2         101  ----------------------------------------------------------------------
HsApoA4         396  ----------------------------------------------------------------------
HsApoA5         356  ----------------------------------------------------------------------
HsApoB48        1873 ----------------------------------------------------------------------
HsApoB52        1914 ----------------------------------------------------------------------
HsApoC1         129  ----------------------------------------------------------------------
HsApoC2         102  ----------------------------------------------------------------------
HsApoC3         100  ----------------------------------------------------------------------
HsApoC4         128  ----------------------------------------------------------------------
HsApoD          246  ----------------------------------------------------------------------
HsApoE2         317  ----------------------------------------------------------------------
HsApoF          326  ----------------------------------------------------------------------
HsApoH          343  ----------------------------------------------------------------------
HsApoJ          449  ----------------------------------------------------------------------
HsApoL1         387  ----------------------------------------------------------------------
HsApoL2         326  ----------------------------------------------------------------------
HsApoL3         320  ----------------------------------------------------------------------
HsApoL4         340  ----------------------------------------------------------------------
HsApoOL(2)      345  ----------------------------------------------------------------------
HsApoL6         287  ----------------------------------------------------------------------
HsApoM          189  ----------------------------------------------------------------------
HsApoO          199  ----------------------------------------------------------------------
HsApoOL         269  ----------------------------------------------------------------------
HsMTTP          774  ----------------------------------------------------------------------
DmCG31659       193  ----------------------------------------------------------------------
DmMtp           777  ----------------------------------------------------------------------
DmCvD           1272 ----------------------------------------------------------------------
DmGlaz          213  ----------------------------------------------------------------------
DmFabp          159  ----------------------------------------------------------------------
DmApoLTPII      660  ----------------------------------------------------------------------
DmApoLTPI       2891 KFVFDPEVGIIDLEQKLPMSWHAFNETPRFEEIPEYQVLAKAQSFFSETNSSIVMKLYNMRTHLDPKTWL
DmNlaz          239  ----------------------------------------------------------------------
DmApoLII        592  ----------------------------------------------------------------------
DmApoLI         2122 ----------------------------------------------------------------------
DmMICOS_A       222  ----------------------------------------------------------------------
Cel_MICOS       202  ----------------------------------------------------------------------
Cel_Vit_2       1307 ----------------------------------------------------------------------
Cel_Vit_4       1305 ----------------------------------------------------------------------
Cel_Vit_5       1305 ----------------------------------------------------------------------
Cel_ApoL_1      266  ----------------------------------------------------------------------
Cel_MTTP        803  ----------------------------------------------------------------------
Cel_Vit_6       1337 ----------------------------------------------------------------------
Cel_Vit_3       1305 ----------------------------------------------------------------------
Cel_Vit-1       1309 ----------------------------------------------------------------------
Cel_Apo_L3_Like 284  ----------------------------------------------------------------------

HsApoA1         158  ----------------------------------------------------------------------
HsApoA2         101  ----------------------------------------------------------------------
HsApoA4         396  ----------------------------------------------------------------------
HsApoA5         356  ----------------------------------------------------------------------
HsApoB48        1873 ----------------------------------------------------------------------
HsApoB52        1914 --------------------------------------------------------------------LT
HsApoC1         129  ----------------------------------------------------------------------
HsApoC2         102  ----------------------------------------------------------------------
HsApoC3         100  ----------------------------------------------------------------------
HsApoC4         128  ----------------------------------------------------------------------
HsApoD          246  ----------------------------------------------------------------------
HsApoE2         317  ----------------------------------------------------------------------
HsApoF          326  ----------------------------------------------------------------------
HsApoH          343  ----------------------------------------------------------------------
HsApoJ          449  ----------------------------------------------------------------------
HsApoL1         387  ----------------------------------------------------------------------
HsApoL2         326  ----------------------------------------------------------------------
HsApoL3         320  ----------------------------------------------------------------------
HsApoL4         340  ----------------------------------------------------------------------
HsApoOL(2)      345  ----------------------------------------------------------------------
HsApoL6         287  ----------------------------------------------------------------------
HsApoM          189  ----------------------------------------------------------------------
HsApoO          199  ----------------------------------------------------------------------
HsApoOL         269  ----------------------------------------------------------------------
HsMTTP          774  ----------------------------------------------------------------------
DmCG31659       193  ----------------------------------------------------------------------
DmMtp           777  ----------------------------------------------------------------------
DmCvD           1272 ----------------------------------------------------------------------
DmGlaz          213  ----------------------------------------------------------------------
DmFabp          159  ----------------------------------------------------------------------
DmApoLTPII      660  ----------------------------------------------------------------------
DmApoLTPI       2961 PPYYSRALLIDSRHYMTFDQRYVGLNLNFDELGNGRSTSQCSYLLAHDFFKRNFTLLLEPASKSLAGQGL
DmNlaz          239  ----------------------------------------------------------------------
DmApoLII        592  ----------------------------------------------------------------------
DmApoLI         2122 ------------------------LTNGKLKSITLIDREGSYFEVADNLALKLNGNLVEYPQHLSGLHAW
DmMICOS_A       222  ----------------------------------------------------------------------
Cel_MICOS       202  ----------------------------------------------------------------------
Cel_Vit_2       1307 ----------------------------------------------------------------------
Cel_Vit_4       1305 ----------------------------------------------------------------------
Cel_Vit_5       1305 ----------------------------------------------------------------------
Cel_ApoL_1      266  ----------------------------------------------------------------------
Cel_MTTP        803  ----------------------------------------------------------------------
Cel_Vit_6       1337 ----------------------------------------------------------------------
Cel_Vit_3       1305 ----------------------------------------------------------------------
Cel_Vit-1       1309 ----------------------------------------------------------------------
Cel_Apo_L3_Like 284  ----------------------------------------------------------------------
```

```
HsApoA2          101  ----------------------------------------------------------------------
HsApoA4          396  ----------------------------------------------------------------------
HsApoA5          356  ----------------------------------------------------------------------
HsApoB48         1873 ----------------------------------------------------------------------
HsApoB52         1917 LREVSSKLRRNLQNNAEWVYQGAIRQIDDIDVRFQKAASGTTGTYQEWKDKAQNLYQELLTQEGQASFQG
HsApoC1          129  ----------------------------------------------------------------------
HsApoC2          102  ----------------------------------------------------------------------
HsApoC3          100  ----------------------------------------------------------------------
HsApoC4          128  ----------------------------------------------------------------------
HsApoD           246  ----------------------------------------------------------------------
HsApoE2          317  ----------------------------------------------------------------------
HsApoF           326  ----------------------------------------------------------------------
HsApoH           343  --------------------PC------------------------------------------------
HsApoJ           449  ----------------------------------------------------------------------
HsApoL1          387  ----------------------------------------------------------------------
HsApoL2          326  -----------------IHEMLQ-----------------------------------------------
HsApoL3          320  ----------------------------------------------------------------------
HsApoL4          340  ----------------------------------------------------------------------
HsApoOL(2)       345  -----------------HHRHLP-----------------------------------------------
HsApoL6          287  -----------------LYKSLQ-----------------------------------------------
HsApoM           189  ----------------------------------------------------------------------
HsApoO           199  ----------------------------------------------------------------------
HsApoOL          269  ----------------------------------------------------------------------
HsMTTP           774  ----------------------------------------------------------------------
DmCG31659        193  ----------------------------------------------------------------------
DmMtp            777  -----------------QQNTGS-----------------------------------------------
DmCvD            1272 -----------------LKRSCS-----------------------------------------------
DmGlaz           213  ----------------------------------------------------------------------
DmFabp           159  ----------------------------------------------------------------------
DmApoLTPII       660  ----------------------------------------------------------------------
DmApoLTPI        3031 TRKLSFIANGQLIEIDLETDHISINGNPQPILPLKLGDVNIHRDLDVLSITSDTEFSLHCNVQFDLCWFE
DmNlaz           239  -----------------NFLSY------------------------------------------------
DmApoLII         592  ----------------------------------------------------------------------
DmApoLI          2169 RRFYTIHLYSEYGVGIVCTSDLKVCHININGFYTSKTRGLLGNGNAEPYDDFLLIDGTLAENSAALGNDY
DmMICOS_A        222  ------------------PKKKP-----------------------------------------------
Cel_MICOS        202  ----------------------------------------------------------------------
Cel_Vit_2        1307 -------------------SVCK-----------------------------------------------
Cel_Vit_4        1305 -------------------ATCK-----------------------------------------------
Cel_Vit_5        1305 -------------------ATCK-----------------------------------------------
Cel_ApoL_1       266  ----------------------------------------------------------------------
Cel_MTTP         803  -----------------PVRHVE-----------------------------------------------
Cel_Vit_6        1337 -----------------KQANCV-----------------------------------------------
Cel_Vit_3        1305 -------------------ATCK-----------------------------------------------
Cel_Vit-1        1309 -------------------SVCK-----------------------------------------------
Cel_Apo_L3_Like  284  ----------------------------------------------------------------------

HsApoA1          158  ----------------------------------------------------------------------
HsApoA2          101  ----------------------------------------------------------------------
HsApoA4          396  ----------------------------------------------------------------------
HsApoA5          356  --------------------------------------------DQGHSHLGDPT---------------
HsApoB48         1873 -------------------------------GLASAIDMSTNYNSDSLHFSNVFRSVMAPFTMTIDAHTN
HsApoB52         1987 LKDNVFDGLVRVTQEFHMKVKHLIDSLIDFLNFPRFQFPGKPGIYTREELCTMFIREVGTVLSQVYSKVH
HsApoC1          129  ----------------------------------------------------------------------
HsApoC2          102  ----------------------------------------------------------------------
HsApoC3          100  ----------------------------------------------------------------------
HsApoC4          128  ----------------------------------------------------------------------
HsApoD           246  ----------------------------------------------------------------------
HsApoE2          317  ----------------------------------------------------------------------
HsApoF           326  ----------------------------------------------------------------------
HsApoH           345  ----------------------------------------------------------------------
HsApoJ           449  ----------------------------------------------------------------------
HsApoL1          387  -----------------------------------------------NYKILQADQELP-----------
HsApoL2          332  ---------------------------------------------PGQDQV-------------------
HsApoL3          320  ---------------------------------------------------------IYQRLNPCHTHQ-
HsApoL4          340  ---------------------------------------------IHQSLKAG-----------------
HsApoOL(2)       351  -------------------------------QKASQTCSSSRGRAVRGSRVVKPEGSRSPLPWPVVEHQP
HsApoL6          293  ------------------------------------QKVRSRARGVGKDLTGTCETEAYWKELREHVWMW
HsApoM           189  ----------------------------------------------------------------------
HsApoO           199  ----------------------------------------------------------------------
HsApoOL          269  ----------------------------------------------------------------------
HsMTTP           774  -----------------------------------------------KTRVKNRVTVVITTDITVDSSFV
DmCG31659        193  ----------------------------------------------------------------------
DmMtp            783  ---------------------------------------------AVLGHLAVGFTYAKLVQDFSITHEP
DmCvD            1278 -------------------------VINGIVNTFDDYLINLREIAVRPDCLTLLVADCSPLPQIAVFVTP
DmGlaz           213  ----------------------------------------------------------------------
DmFabp           159  ----------------------------------------------------------------------
DmApoLTPII       660  --------------------------------------MDIRKFSRNYEHSLFFDEYNFGTTTDANVIFG
DmApoLTPI        3101 VSGWYFGRTAGLLGTLNNEPYDEYTMSSGVISNETQLFTDSWSLKQCRQNKLAQTQEVSQEVSDACTSFF
DmNlaz           244  ----------------------------------------------------------------------
DmApoLII         592  ---------------------------------------------IRVTKTFPVDYRRYSFNNEVSYKLE
DmApoLI          2239 GVGKCTAIEFDNNQFKSSKRQEMCSELFGIESTLAFNFITLDSRPYRKACDIALAKVAEKEKEATACTFA
DmMICOS_A        227  ----------------------------------------------------------------------
Cel_MICOS        202  ----------------------------------------------------------------------
Cel_Vit_2        1311 ---------------------------------------------VQKNQIRTFDDVLYNTPLTTCYSLI
Cel_Vit_4        1309 ---------------------------------------------VDQSEVSTFDNVIYRAPLTTCYSLV
Cel_Vit_5        1309 ---------------------------------------------VDQSEVSTFDNVIYRAPLTTCYSLV
Cel_ApoL_1       266  ----------------------------------------------------------------------
Cel_MTTP         809  ---------------------------------------------STISALSTFTTDTRAIFETLPYDFC
Cel_Vit_6        1343 ---------------------------------------------VKSTKINTFDQVAYRNQFTPCYSVL
Cel_Vit_3        1309 ---------------------------------------------VGQSEVSTFDNVIYRAPLTTCYSLV
Cel_Vit-1        1313 ---------------------------------------------VQKNQIRTFDDVLYNTPLTTCYSLI
Cel_Apo_L3_Like  284  ---------------------------------------------DVLSDVGSIEMVQPESPNQSLIMDP
```

```
HsApoA2          101  ----------------------------------------------------------------------
HsApoA4          396  ----------------------------------------------------------------------
HsApoA5          367  ----------------------------------------------------------------------
HsApoB48         1913 GNGKLALW--------------------------------------------------------------
HsApoB52         2057 NGSEILFS--------------------------------------------------------------
HsApoC1          129  ----------------------------------------------------------------------
HsApoC2          102  ----------------------------------------------------------------------
HsApoC3          100  ----------------------------------------------------------------------
HsApoC4          128  ----------------------------------------------------------------------
HsApoD           246  ----------------------------------------------------------------------
HsApoE2          317  ----------------------------------------------------------------------
HsApoF           326  ----------------------------------------------------------------------
HsApoH           345  ----------------------------------------------------------------------
HsApoJ           449  ----------------------------------------------------------------------
HsApoL1          399  ----------------------------------------------------------------------
HsApoL2          338  ----------------------------------------------------------------------
HsApoL3          332  ----------------------------------------------------------------------
HsApoL4          348  ----------------------------------------------------------------------
HsApoOL(2)       391  RLGPGVAL--------------------------------------------------------------
HsApoL6          328  LWLCVCLC--------------------------------------------------------------
HsApoM           189  ----------------------------------------------------------------------
HsApoO           199  ----------------------------------------------------------------------
HsApoOL          269  ----------------------------------------------------------------------
HsMTTP           798  KAGLETST--------------------------------------------------------------
DmCG31659        193  ----------------------------------------------------------------------
DmMtp            809  KLSLNADL--------------------------------------------------------------
DmCvD            1324 SPVQGLST--------------------------------------------------------------
DmGlaz           213  ----------------------------------------------------------------------
DmFabp           159  ----------------------------------------------------------------------
DmApoLTPII       693  TDSYL-----------------------------------------------------------------
DmApoLTPI        3171 RTGILATCSAVLDPTPFYEMCMDLGMKSPPIRKGHPAVKGACAAALAYIEACTALKVPMRVPSQCVFCQL
DmNlaz           244  ----------------------------------------------------------------------
DmApoLII         618  SLGVGAST--------------------------------------------------------------
DmApoLI          2309 LAYGSAVK--------------------------------------------------------------
DmMICOS_A        227  ----------------------------------------------------------------------
Cel_MICOS        202  ----------------------------------------------------------------------
Cel_Vit_2        1337 AKDCS-----------------------------------------------------------------
Cel_Vit_4        1335 AKDCS-----------------------------------------------------------------
Cel_Vit_5        1335 AKDCS-----------------------------------------------------------------
Cel_ApoL_1       266  ----------------------------------------------------------------------
Cel_MTTP         835  LRTSNSNV--------------------------------------------------------------
Cel_Vit_6        1369 AKDCGSEK--------------------------------------------------------------
Cel_Vit_3        1335 AKDCS-----------------------------------------------------------------
Cel_Vit-1        1339 AKDCS-----------------------------------------------------------------
Cel_Apo_L3_Like  310  HQQESSVQH-------------------------------------------------------------

HsApoA1          158  ----------------------------------------------------------------------
HsApoA2          101  ----------------------------------------------------------------------
HsApoA4          396  ----------------------------------------------------------------------
HsApoA5          367  ----------------------------------------------------------------------
HsApoB48         1920 -----------------GEHTGQLYSKFLLKAEPLAFTFSHDYKGSTSHHLVSRKSISAALEHKVSALLT
HsApoB52         2064 -----------------YFQDLVITLPFELRKHKLIDVISMYRELLKDLSKEAQEVFKAIQSLKTTEVLR
HsApoC1          129  ----------------------------------------------------------------------
HsApoC2          102  ----------------------------------------------------------------------
HsApoC3          100  ----------------------------------------------------------------------
HsApoC4          128  ----------------------------------------------------------------------
HsApoD           246  ----------------------------------------------------------------------
HsApoE2          317  ----------------------------------------------------------------------
HsApoF           326  ----------------------------------------------------------------------
HsApoH           345  ----------------------------------------------------------------------
HsApoJ           449  ----------------------------------------------------------------------
HsApoL1          399  ----------------------------------------------------------------------
HsApoL2          338  ----------------------------------------------------------------------
HsApoL3          332  ----------------------------------------------------------------------
HsApoL4          348  ----------------------------------------------------------------------
HsApoOL(2)       398  -----------------RTPKRTVSAPRMLGHQPAPPAPARKGRQAPGRHRQ------------------
HsApoL6          335  -----------------VCVYVQFTT--------------------------------------------
HsApoM           189  ----------------------------------------------------------------------
HsApoO           199  ----------------------------------------------------------------------
HsApoOL          269  ----------------------------------------------------------------------
HsMTTP           805  -----------------ETEAGLEFISTVQFSQYPFLVCMQMDKDEAPFRQFEKKYERLSTGRGYVSQKR
DmCG31659        193  ----------------------------------------------------------------------
DmMtp            816  -----------------DFYSGIKLCMQLQRPEQLLKQTNVRSVFLQSVDRPYAKHVRSTLSHKTAGCTF
DmCvD            1331 -----------------NYGLRVHIGQNYFNFRARTDNSSLPTDEPVLIYLNQDQTPHNVRKKPYQWPIE
DmGlaz           213  ----------------------------------------------------------------------
DmFabp           159  ----------------------------------------------------------------------
DmApoLTPII       697  -----------------PRIASVNFTADLFGQSVNFFEFTARAEGLEELAANAFGPKGPLSGQLLRKKLS
DmApoLTPI        3241 SNGSYVPEGTFMELSGPEIPKSSDVVFIVEAKECNANLKTSKNIMTVVSSIEEQLQAAKITNNRYAVVAF
DmNlaz           244  ----------------------------------------------------------------------
DmApoLII         625  -----------------DYQIIYSQHGFLPRSSRINVTTEFFGTNYNVFEASVRQENVEDVLEYYLGPKG
DmApoLI          2316 -----------------QINKWVLLPPRCIKCAGPAGQHDFGDEFTVKLPNNKVDVVFVVDINVTPGVLS
DmMICOS_A        227  ----------------------------------------------------------------------
Cel_MICOS        202  ----------------------------------------------------------------------
Cel_Vit_2        1341 -----------------EEPTFAVLSKKTEKNSEEMIIKVIRGEQEIVAQLQNEEIRVKVDGKKIQSE--
Cel_Vit_4        1339 -----------------EQPRFAVLAKKINKNSEELLVKVVRREEEIVVKKSDDKFLVKVDGKKVNPT--
Cel_Vit_5        1339 -----------------EQPRFAVLAKKINKNSEELLVKVVRREEEIVVKKSDDKFLVKVDGKKVNPT--
Cel_ApoL_1       266  ----------------------------------------------------------------------
Cel_MTTP         842  -----------------DINQKTVVQDQIGKHKKKTLNRKRVHPGVTYRLDDSTIRQCNSYLEQFRLV--
Cel_Vit_6        1376 -----------------SEPRFVVLMKKINEKKEWKNVKVVYGENEIEMYKTEEGLICRVNGEEIEYQPE
Cel_Vit_3        1339 -----------------EQPRFAVLAKKINKNSEELLVKVVRREEEIVVKKSDDKFLVKVDEKKVNPT--
Cel_Vit-1        1343 -----------------EEPTFAVLSKKTEKNSEEMIIKVIRGEQEIVAQLQNEEIRVKVDGKKILSE--
Cel_Apo_L3_Like  318  ----------------------------------------------------------------------
```

```
HsApoA2         101  ----------------------------------------------------------------------
HsApoA4         396  ----------------------------------------------------------------------
HsApoA5         367  ----------------------------------------------------------------------
HsApoB48        1974 PAEQTGTWKLKTQFNNNEYSQDLDAYNTKDKIGVELTGRTLADLTLLDSPIKVPLLLSEPINIIDALEMR
HsApoB52        2118 NLQDLLQFIFQLIEDNIKQLKEMKFTYLINYIQDEINTIFSDYIPYVFKLLKENLCLNLHKFNEFIQNEL
HsApoC1         129  ----------------------------------------------------------------------
HsApoC2         102  ----------------------------------------------------------------------
HsApoC3         100  ----------------------------------------------------------------------
HsApoC4         128  ----------------------------------------------------------------------
HsApoD          246  ----------------------------------------------------------------------
HsApoE2         317  ----------------------------------------------------------------------
HsApoF          326  ----------------------------------------------------------------------
HsApoH          345  ----------------------------------------------------------------------
HsApoJ          449  ----------------------------------------------------------------------
HsApoL1         399  ----------------------------------------------------------------------
HsApoL2         338  ----------------------------------------------------------------------
HsApoL3         332  ----------------------------------------------------------------------
HsApoL4         348  ----------------------------------------------------------------------
HsApoOL(2)      433  ----------------------------------------------------------------------
HsApoL6         344  ----------------------------------------------------------------------
HsApoM          189  ----------------------------------------------------------------------
HsApoO          199  ----------------------------------------------------------------------
HsApoOL         269  ----------------------------------------------------------------------
HsMTTP          859  KESVLAGCEFPLHQENSEMCKVVFAPQPDSTSSGWF----------------------------------
DmCG31659       193  ----------------------------------------------------------------------
DmMtp           870  ALNQKNNEMCNLIFRDLD----------------------------------------------------
DmCvD           1385 TSDYDFRVELNEQNILIVECTQLSSTIQFDLYNILNFEIYGVYKHQMCGLCSKPLNRMQNYTICELEANT
DmGlaz          213  ----------------------------------------------------------------------
DmFabp          159  ----------------------------------------------------------------------
DmApoLTPII      751  FLNRWLGNESAEEDDTLENLLSLDNLRLKW----------------------------------------
DmApoLTPI       3311 GGVSPYDKARSVIYEHNEFTSKPEQLADYFGHINTGNGSSNDILMAISAAAKLNFRPGVSKTFILLSCSK
DmNlaz          244  ----------------------------------------------------------------------
DmApoLII        679  LVNKDFDEIVKLIEVGNNGVAAGGRARR------------------------------------------
DmApoLI         2370 NLIAPAINDIRESLRSRGFSDVQVGVIVFEETKRYPALLTSDGGKINYKGNVADVKLAGIKSFCDNCVEQ
DmMICOS_A       227  ----------------------------------------------------------------------
Cel_MICOS       202  ----------------------------------------------------------------------
Cel_Vit_2       1392 ---DYSAYQIERLGESAIVIELPEGEVRFDGYTIKTQLPSYSRKNQLCGLCGNNDDESTNEFYTSDNTET
Cel_Vit_4       1390 ---ELEQYNIEILGDNLIVIRLPHGEVRFDGYTVKTNMPSVASQNQLCGLCGNNDGERDNEFMTADNYET
Cel_Vit_5       1390 ---ELEQYNIEILGDNLIVIRLPQGEVRFDGYTVKTNMPSVASQNQLCGLCGNNDGERDNEFMTADNYET
Cel_ApoL_1      266  ----------------------------------------------------------------------
Cel_MTTP        893  ----------------------------------------------------------------------
Cel_Vit_6       1430 SEIEKKQYNIIWLNKNTLKFDSDDVTVQFDGVNARIHLSALYR-NQQCGLCGHYDNEKETEFYDAENQE-
Cel_Vit_3       1390 ---ELEQYNIEILGDNLIVIRLPHGEVRFDGYTVKTNMPSVASQNQLCGLCGNNDGERDNEFMTADNYET
Cel_Vit-1       1394 ---DYSAHQIERLGESDIVIELPEGEVRFDGYTIKTQLPSYSRKNQLCGLCGNNDDESTNEFYTSDNTET
Cel_Apo_L3_Like 318  ----------------------------------------------------------------------

HsApoA1         158  ----------------------------------------------------------------------
HsApoA2         101  ----------------------------------------------------------------------
HsApoA4         396  ----------------------------------------------------------------------
HsApoA5         367  ----------------------------------------------------------------------
HsApoB48        2044 DAVEKPQEFTIVAFVKYDKNQDVHSINLPFFETLQEYFERNRQTIIVVLENVQRNLKHINIDQFVRKYRA
HsApoB52        2188 QEASQELQQIHQYIMALREEYFDPSIVGWTVKYYELEEKIVSLIKNLLVALKDFHSEYIVSASNFTSQLS
HsApoC1         129  ----------------------------------------------------------------------
HsApoC2         102  ----------------------------------------------------------------------
HsApoC3         100  ----------------------------------------------------------------------
HsApoC4         128  ----------------------------------------------------------------------
HsApoD          246  ----------------------------------------------------------------------
HsApoE2         317  ----------------------------------------------------------------------
HsApoF          326  ----------------------------------------------------------------------
HsApoH          345  ----------------------------------------------------------------------
HsApoJ          449  ----------------------------------------------------------------------
HsApoL1         399  ----------------------------------------------------------------------
HsApoL2         338  ----------------------------------------------------------------------
HsApoL3         332  ----------------------------------------------------------------------
HsApoL4         348  ----------------------------------------------------------------------
HsApoOL(2)      433  ----------------------------------------------------------------------
HsApoL6         344  ----------------------------------------------------------------------
HsApoM          189  ----------------------------------------------------------------------
HsApoO          199  ----------------------------------------------------------------------
HsApoOL         269  ----------------------------------------------------------------------
HsMTTP          894  ----------------------------------------------------------------------
DmCG31659       193  ----------------------------------------------------------------------
DmMtp           887  ----------------------------------------------------------------------
DmCvD           1455 PTPVPLQNSSDVVVVA------------------------------------------------------
DmGlaz          213  ----------------------------------------------------------------------
DmFabp          159  ----------------------------------------------------------------------
DmApoLTPII      780  ----------------------------------------------------------------------
DmApoLTPI       3381 CAARDMRFDYTSILQYLLEEGVNLHILADTEFDFERNKKLRHFFGLDSKLVYSKRFPEGDAETRNTTHIP
DmNlaz          244  ----------------------------------------------------------------------
DmApoLII        706  ----------------------------------------------------------------------
DmApoLI         2440 IITEKRILDIYNSLKEIVKGIAPQADEKAFQLALDYPFRAGAAKSIIGVRSDSLEYKNWWKFVRAQLTGS
DmMICOS_A       227  ----------------------------------------------------------------------
Cel_MICOS       202  ----------------------------------------------------------------------
Cel_Vit_2       1460 EDIEEFHRSYLLKNEECEAEEERLSEKKNYR-KYERDEEQSDEYSSEETYDYEQENTKKSQKNQRSQKKS
Cel_Vit_4       1458 EDVEEFHRSYLLKNEECEVEKDRISEKKNYKNKWNREEKKSD---YESSSDYESNYDEKETEKE------
Cel_Vit_5       1458 EDVEEFHRSYLLKNEECEVENDRISEKKNYRNKWNREEKKSD---YESSSDYESNYDEKETEKE------
Cel_ApoL_1      266  ----------------------------------------------------------------------
Cel_MTTP        893  ----------------------------------------------------------------------
Cel_Vit_6       1498 NTIPKFAKSYLYKDSKCNYEREMFEKEENFQ-RIEKNQEEEK----DQEMNYEESRREQDDEPT------
Cel_Vit_3       1458 EDVEEFHRSYLLKNEECEVENDRISEKKNYRNKWNREEKKSD---YVSSSDYENNYDEKETENQ------
Cel_Vit-1       1462 KDIEEFHRSYLLKNEECAEEERLSEKKNYR-KYDERKYESEEYSFEETYDYEQENTNKKQKNQRSQKKS
Cel_Apo_L3_Like 318  ----------------------------------------------------------------------
```

```
HsApoA2         101  ----------------------------------------------------------------------
HsApoA4         396  ----------------------------------------------------------------------
HsApoA5         367  ----------------------------------------------------------------------
HsApoB48        2114 ALGKLPQQANDYLNSFNWERQVSHAKEKLTALTKKYRITENDIQIALDDAKINFNEKLSQLQTYMIQ---
HsApoB52        2258 SQVEQFLHRNIQEYLSILTDPDGKGKEKIAELSATAQEIIKSQAIATKKIISDYHQQFRYKLQDFSDQLS
HsApoC1         129  ----------------------------------------------------------------------
HsApoC2         102  ----------------------------------------------------------------------
HsApoC3         100  ----------------------------------------------------------------------
HsApoC4         128  ----------------------------------------------------------------------
HsApoD          246  ----------------------------------------------------------------------
HsApoE2         317  ----------------------------------------------------------------------
HsApoF          326  ----------------------------------------------------------------------
HsApoH          345  ----------------------------------------------------------------------
HsApoJ          449  ----------------------------------------------------------------------
HsApoL1         399  ----------------------------------------------------------------------
HsApoL2         338  ----------------------------------------------------------------------
HsApoL3         332  ----------------------------------------------------------------------
HsApoL4         348  ----------------------------------------------------------------------
HsApoOL(2)      433  ----------------------------------------------------------------------
HsApoL6         344  ----------------------------------------------------------------------
HsApoM          189  ----------------------------------------------------------------------
HsApoO          199  ----------------------------------------------------------------------
HsApoOL         269  ----------------------------------------------------------------------
HsMTTP          894  ----------------------------------------------------------------------
DmCG31659       193  ----------------------------------------------------------------------
DmMtp           887  ----------------------------------------------------------------------
DmCvD           1470 ----------------------------------------------------------------------
DmGlaz          213  ----------------------------------------------------------------------
DmFabp          159  ----------------------------------------------------------------------
DmApoLTPII      780  ----------------------------------------------------------------------
DmApoLTPI       3451 KSNLGICTTLAVETQGSVFSARKLQPERKYPIKRFATIFAKRVALSATPIQSQTCECSAHNTGVSYMACS
DmNlaz          244  ----------------------------------------------------------------------
DmApoLII        706  ----------------------------------------------------------------------
DmApoLI         2510 ITKFDGALIHLIAPVKGLSLEGVLSEKLIGFNSRLVATVDGKDSKKRTKLQFDNDMGIDFVLNNGGWVFA
DmMICOS_A       227  ----------------------------------------------------------------------
Cel_MICOS       202  ----------------------------------------------------------------------
Cel_Vit_2       1529 DLVEKTQIKEFSHRICFSVEPVAEC-RRGYEVEQQQQRKIRFTCLQRHNRDASRLLKESRQQPL-QLDDY
Cel_Vit_4       1518 -LVKKTLIKEFSNRVCFSIEPVSEC-RRGLESEKTSNKKIRFTCMPRHSKNARRFLKEAREQTVADLVDF
Cel_Vit_5       1518 -LVKKTLIKEFSNRVCFSIEPVSEC-RRGLESEKTSNKKIRFTCMPRHSKNARRFLKEAREQTVAELVDF
Cel_ApoL_1      266  ----------------------------------------------------------------------
Cel_MTTP        893  ----------------------------------------------------------------------
Cel_Vit_6       1556 ---EQVAIVERQHEICFTQKPVLRC-QNG-KSQESKKQKTSVYCLPSSNSWARRQMREIRREPLAQWPEH
Cel_Vit_3       1518 -LFKKTLIKEFSNRVCFSIEPVSEC-RRGLESEKTSNEKIRFTCMPRHSKNARRFLKEAREQTVADLVDF
Cel_Vit-1       1531 DLVEKTQIKEFSHRICFSVEPVAECRRRGYEAVEQQQRKVRFTCLPRHSSEARRLVKEARQGTV-QLDDH
Cel_Apo_L3_Like 318  ----------------------------------------------------------------------

HsApoA1         158  ------------------------------------------------------------------
HsApoA2         101  ------------------------------------------------------------------
HsApoA4         396  ------------------------------------------------------------------
HsApoA5         367  ------------------------------------------------------------------
HsApoB48        2180 ------------------------------------------------------------------
HsApoB52        2328 DYYEKFIAESKRLIDLSIQNYHTFLIYITELLKKLQSTTVMNPYMKLAPGELTIIL----------
HsApoC1         129  ------------------------------------------------------------------
HsApoC2         102  ------------------------------------------------------------------
HsApoC3         100  ------------------------------------------------------------------
HsApoC4         128  ------------------------------------------------------------------
HsApoD          246  ------------------------------------------------------------------
HsApoE2         317  ------------------------------------------------------------------
HsApoF          326  ------------------------------------------------------------------
HsApoH          345  ------------------------------------------------------------------
HsApoJ          449  ------------------------------------------------------------------
HsApoL1         399  ------------------------------------------------------------------
HsApoL2         338  ------------------------------------------------------------------
HsApoL3         332  ------------------------------------------------------------------
HsApoL4         348  ------------------------------------------------------------------
HsApoOL(2)      433  ------------------------------------------------------------------
HsApoL6         344  ------------------------------------------------------------------
HsApoM          189  ------------------------------------------------------------------
HsApoO          199  ------------------------------------------------------------------
HsApoOL         269  ------------------------------------------------------------------
HsMTTP          894  ------------------------------------------------------------------
DmCG31659       193  ------------------------------------------------------------------
DmMtp           887  ------------------------------------------------------------------
DmCvD           1470 ------------------------------------------------------------------
DmGlaz          213  ------------------------------------------------------------------
DmFabp          159  ------------------------------------------------------------------
DmApoLTPII      780  ------------------------------------------------------------------
DmApoLTPI       3521 PQALPEEKYDLDDYDSFNNWDWGDEPESETNVMS--------------------------------
DmNlaz          244  ------------------------------------------------------------------
DmApoLII        706  ------------------------------------------------------------------
DmApoLI         2580 TQNFEKLKASDQKKMLNQITSSLADTLFKTEIVSDCRCLPIHGLHGQHKCVIKSSTFVANKKAKSA
DmMICOS_A       227  ------------------------------------------------------------------
Cel_MICOS       202  ------------------------------------------------------------------
Cel_Vit_2       1597 PVSFVES----------VKVPTAC--VAYS------------------------------------
Cel_Vit_4       1587 PVSFVES----------VKIPTAC--VAYV------------------------------------
Cel_Vit_5       1587 PVSFVES----------VKIPTAC--VAYV------------------------------------
Cel_ApoL_1      266  ------------------------------------------------------------------
Cel_MTTP        893  ------------------------------------------------------------------
Cel_Vit_6       1622 KLRNLRDQPQMEERTVRVAVDQKCDKFDY-------------------------------------
Cel_Vit_3       1587 PVSFVES----------VKIPTAC--VAYR------------------------------------
Cel_Vit-1       1600 KISFVHS----------VQVPVAC--VAY-------------------------------------
Cel_Apo_L3_Like 318  ------------------------------------------------------------------
```
